# Decoding the immune landscape following hip fracture in elderly patients: unveiling temporal dynamics through single-cell RNA sequencing

**DOI:** 10.1186/s12979-023-00380-6

**Published:** 2023-10-17

**Authors:** Yining Lu, Yang Luo, Qi Zhang, Wei Chen, Ning Zhang, Ling Wang, Yingze Zhang

**Affiliations:** 1 Department of Orthopaedic Surgery, The Third Hospital of Hebei Medical University, Shijiazhuang, Hebei People’s Republic of China; 2 Department of Orthopedic Research Center, The Third Hospital of Hebei Medical University, Shijiazhuang, Hebei P.R. China; 3 Department of Orthopedic Oncology, The Third Hospital of Hebei Medical University, Shijiazhuang, Hebei P.R. China; 4 Chinese Academy of Engineering, Beijing, 100088 People’s Republic of China

**Keywords:** Hip fracture, Aging, Immune, Inflammation, Bone, Single-cell RNA sequencing

## Abstract

**Background:**

Hip fractures in the elderly have significant consequences, stemming from the initial trauma and subsequent surgeries. Hidden blood loss and stress due to concealed injury sites could impact the whole osteoimmune microenvironment. This study employs scRNA-seq technique to map immune profiles in elderly hip fracture patients from post-trauma to the recovery period, investigating the dynamic changes of immune inflammation regulation subgroups.

**Methods:**

We collected peripheral blood samples from four elderly hip fracture patients (two males and two females, all > 75 years of age) at three different time points (24 h post-trauma, 24 h post-operation, and day 7 post-operation) and applied scRNA-seq technique to analyze the cellular heterogeneity and identify differentially expressed genes in peripheral blood individual immune cells from elderly hip fracture patients.

**Results:**

In this study, we analyzed the composition and gene expression profiles of peripheral blood mononuclear cells (PBMCs) from elderly hip fracture patients by scRNA-seq and further identified new CD14 monocyte subpopulations based on marker genes and transcriptional profiles. Distinct gene expression changes were observed in various cell subpopulations at different time points. C-Mono2 monocyte mitochondria-related genes were up-regulated and interferon-related and chemokine-related genes were down-regulated within 24 h post-operation. Further analysis of gene expression profiles at day 7 post-operation showed that C-Mono2 monocytes showed downregulation of inflammation-related genes and osteoblast differentiation-related genes. However, the expression of these genes in cytotoxic T cells, Treg cells, and B cell subsets exhibited a contrasting trend. GZMK^+^CD8^+^ cytotoxic T cells showed downregulation of chemokine-related genes, and Treg cells showed upregulation of genes related to the JAK/STAT signaling pathway. Furthermore, we examined interactions among diverse immune cell subsets, pinpointing specific ligand-receptor pairs. These findings imply cross-talk and communication between various cell types in the post-traumatic immune response.

**Conclusions:**

Our study elucidates the notable alterations in immune cell subpopulations during different stages of hip fracture in elderly patients, both in terms of proportions and differential gene expressions. These changes provide significant clinical implications for tissue repair, infection prevention, and fracture healing in clinic.

**Supplementary Information:**

The online version contains supplementary material available at 10.1186/s12979-023-00380-6.

## Introduction

Osteoporosis symptoms, such as bone loss, decreased bone density, and increased bone fragility, occur in middle and old age, and hip fracture is one of the most common fractures in the elderly [1, 2]. The number of hip fractures is expected to almost double from 2018 to 2050 due to global population aging, particularly in developing countries [3], around 30% of these fractures will occur in Asia, especially China [4]. Hip fractures have a significantly higher mortality rate in the elderly, with reported 1- and 2-year mortality rates of 29% and 38%, respectively, which are much higher than the mortality rates associated with other types of fractures in elderly patients, and have been described as the “last fracture of life” [5]. Studies have shown that even if the patient survives, there is a high risk of disability, and 80% of patients need to use a walker within 1 year after fracture, resulting in a serious decline in quality of life and a huge social burden [6].

The elevated mortality and disability rates observed in elderly hip fracture patients are intricately linked to immune system disorders that occur post-trauma [7], with a pivotal role played by the inflammatory response [8]. After fracture, the immune system initiates a cascade of responses related to the recognition of damage-associated molecular patterns (DAMPs) [9, 10], which stimulate a large number of immune cells to release a variety of inflammatory factors and cause inflammatory reactions [11]. Neutrophils are the initial inflammatory cells to arrive at the fracture site and subsequently attract monocytes/macrophages through the secretion of inflammatory and chemotactic mediators to participate the process of bone healing [12]. During the fracture healing phase, the synergistic effects between immune cells and cytokines initiate the regeneration of cartilage, bone and angiogenesis, and ultimately promoting fracture repair [13]. Under normal conditions, moderate inflammatory response is an important defense that helps the body repair tissues, kill pathogens, etc. With aging proceeds, the human immune system undergoes great changes during this process, which results in immune dysregulation [14]. This makes them more susceptible to a state of post-immune suppression, leading to a slower recovery process after surgery. In addition, as a traumatic intervention, surgery exacerbates the inflammatory response and increases the release of inflammatory mediators to the peripheral circulation system [15]. Therefore, they are more vulnerable to infectious complications, leading to a higher mortality rate among elderly people [16–19]. It has been found that patients who develop systemic inflammatory response syndrome (SIRS) are often elderly patients with immune imbalance, and because they cannot regulate the body’s immune response well, they activate more immune cells and release more inflammatory factors, forming a vicious cycle that progresses to multi-organ failure and eventually leads to patient’s death [20]. For some patients, even after the acute phase of inflammation, the chronic immune inflammatory disorder causing by aging could also increase the risk of heart, lung, brain and other organ’s complications, slows down the fracture healing process, affects limb’s functions and finally leads to disability [21–24]. Therefore, rebalancing the immune inflammation regulation ability and correcting the disordered immune inflammation state is an urgent clinical problem to be solved while actively performing surgical treatment.

Single-cell RNA sequencing (scRNA-seq) techniques are useful for exploring the dynamic development of disease in a variety of different types of immune cells proportions and functions, compensating for the information overwhelm caused in traditional population cell sequencing, and showing unprecedented unprecedented advantages. To investigate the impact of the immune system at various stages of post-trauma in elderly hip fracture patients, we applied scRNA-seq to explore the heterogeneity, gene expression patterns, and cellular interactions among peripheral blood mononuclear cells (PBMCs) in these patients. Our results show that each cell type has its own unique global and dynamic immune response in the pathogenesis and treatment of geriatric hip fractures, which might be used as potential translational treatment strategies in the near future for clinic.

## Methods

### Patient recruitment and ethics

We collected peripheral blood samples from four elderly patients with hip fracture (Age > 75 years), including two male patients and two female patients, at 24 h post-trauma, 24 h post-operation and on day 7 post-operation, respectively. Neither of the four patients had any comorbidities. We summarized the information for all patients in Table 1. All patients were admitted to the hospital within 24 h of trauma and the procedure was completed within 72 h of admission. Bone mineral density of the robust femoral trochanter was determined by dual-energy X-ray absorptiometry (DXA). We obtained informed consent from four human subjects and our study was approved by the Ethics Committee of the Third Hospital of Hebei Medical University (Ke-2023-051-1).

**Table 1:** Clinical information for all patients

Characteristic	Patient 1	Patient 2	Patient 3	Patient 4
**Age**	77	75	79	76
**Gender**	Male	Male	Female	Female
**BMI**	21.7	26.2	23.2	19.1
**Fracture type**	Right femur intertrochanteric fracture	Left femur intertrochanteric fracture	Right femur intertrochanteric fracture	Right femur intertrochanteric fracture
**Cause of fracture**	Fall	Fall	Traffic accident	Fall
**BMD(T-score)**	-2.6	-2.6	-2.7	-2.5
**Intraoperative bleeding**	300	100	300	200
**Postoperative infection**	no	no	no	no
**Postoperative DVT**	no	no	no	no
**HSS score (6 months postoperatively)**	85	83	87	81

### Single-cell RNA-seq processing

PBMC preparation and cell suspensions were performed as described previously [25], according to the manufacturer’s instructions for the 10X Genomics Chromium Single-Cell 3’ Kit (V3), single-cell suspensions were placed into 10x Chromium to capture 8000 single cells. The processes for cDNA amplification and library creation were carried out according to the conventional procedure. LC-Bio Technology Co., Ltd. (Hangzhou, China) sequenced libraries using an Illumina NovaSeq 6000 sequencing system (paired-end multiplexing run, 150 bp) at a minimum depth of 20,000 reads per cell.

### Data processing and data visualization

Illumina bcl2fastq software (version 2.20) was applied to demultiplex the sequencing data and converted it to FASTQ format. The Cell Ranger pipeline (version 6.1.1) was used to demultiplex samples, process barcodes, and count single-cell 3’ genes, and scRNA-seq data were aligned to the GRCh38 reference genome in Ensembl. A total of 119,294 single cells were processed using 10X Genomics Chromium Single Cell 3’ Solution from four elderly hip fracture patients. The Cell Ranger output was loaded into Seurat (version 3.1.1) and used for dimensionality reduction, clustering, and analysis of scRNA-seq data. Then, to filter the environmental RNA contamination caused by these techniques, we used CellBender (version 0.2.0) to assess and adjust the results. To eliminate the influence of other factors on the results, we screened the data according to the following criteria: (1) The number of genes identified in a single cell was between 500 and Inf. (2) The total number of UMIs in a single cell was less than Inf. (3) The percentage of mitochondrial gene expression in a single cell was less than 25%. (4) Multiple cells were removed using the DoubletFinder package. Ultimately, 91,866 cells passed the quality control threshold. We utilized Seurat to reduce the dimensionality of all cells and tSNE to project the cells into 2D space to display the data. The procedure is as follows: First, we calculated the expression value of genes using the LogNormalize technique of the Seurat software’s “Normalization” function; second, the normalized expression value was used to perform principal component analysis (R harmony), and the top 20 PCs were utilized for clustering and tSNE analysis; third, to locate clusters, the weighted Shared Nearest Neighbor graph-based clustering approach was applied. “bimod”: A likelihood-ratio test was used to find marker genes for each cluster using the “FindAllMarkers” function in Seurat. This method chooses marker genes that are expressed in more than 10% of the cells in a cluster and have an average log (fold change) larger than 0.26.

### Dimensionality reduction and annotation of major cell types

The number of unique molecular identifiers, percentage of mitochondrial genes, and genes were scaled to unit variance. Principal component analysis was performed. Clusters were then identified using tSNE. Cell identity was assigned using known markers.

### Detection of differentially expressed genes and pathway analysis

The differentially expressed genes (DEGs) between two groups comparing the same cell types were identified using the defalut parameters via the FindMarkers function in Seurat. These genes were categorized by average log2 (fold change) after being processed with a minimum log2 (fold change) of 0.26 and a maximum adjusted p value of 0.01. Gene Ontology (GO) terms and Kyoto Encyclopedia of Genes and Genomes (KEGG) pathways were used to create the gene sets.

### Pseudotime analysis

Single-cell pseudotime trajectory was constructed using R package ‘monocle2’ (http://cole-trapnell-lab.github.io/monocle-release/docs/;), UMAP method was applied to reduce dimensions, and function of ‘plot_cells’ was used for visualization. The ‘graph_test’ function was used to screen for DEGs. The threshold of Morans index was set at > 0.3 and the q-value (corrected p value) threshold was set at < 0.001 [26].

### Cell-cell interaction analysis

CellPhone DB (v3) was utilized to investigate cell-cell interactions between cells in depth. According to the concept of receptor expression by one cell subpopulation and ligand expression by another, we calculated possible ligand-receptor interactions. The normalized counts of cells in the two groups were independently downloaded and used as input for the CellPhone DB method.

## Results

### The landscape of various cells in PBMCs

**Fig. 1:**
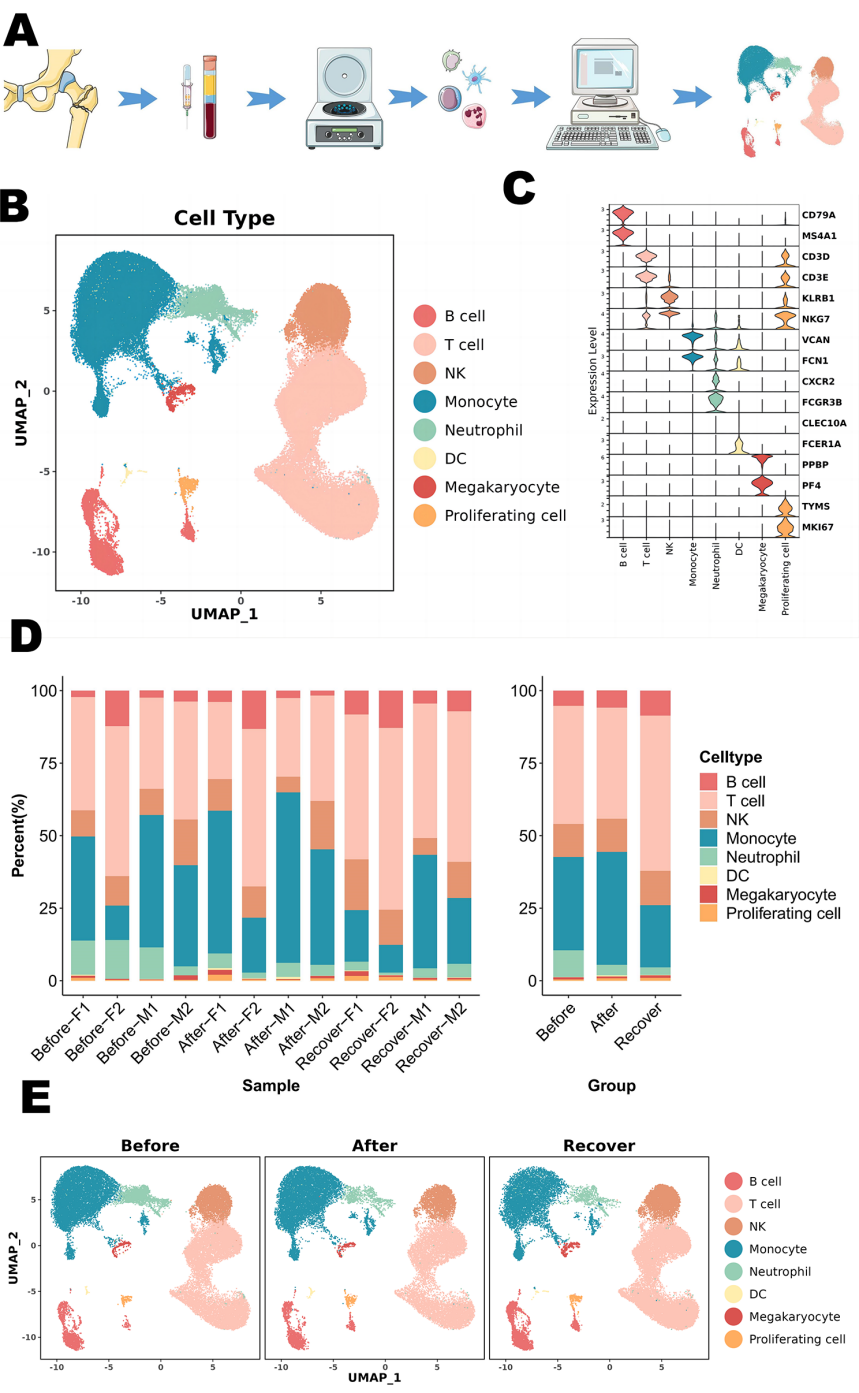
Clustering and categorization of cellular profiles of PBMCs from elderly hip fracture patients. (**A**) Overview of the experimental workflow. (**B**) Visualization of tSNE of PBMCs cell clusters in elderly hip fracture patients. (**C**) Vilin plot showing marker genes for each cell cluster. (**D**) Bar graph showing the relative percentage of cell clusters for each sample. (**E**) Visualization of tSNE of PBMCs cell clusters in elderly hip fracture patients at different time points

In this work, we applied scRNA-seq to analyze PBMCs from elderly hip fracture patients. We identify 8 clusters, which mainly consisted of T cells (CD3D, CD3E), B cells (CD79A, MS4A1), monocytes (VCAN, FCN1), dendritic cells (CLEC10A, FCER1A), natural killer (NK) cells (CLEC10A, FCER1A), megakaryocytes (PPBP, PF4), proliferating cells (TYMS, MKI67), and neutrophils (CXCR2, FCGR3B) (Fig. 1C).

**Fig. 2:**
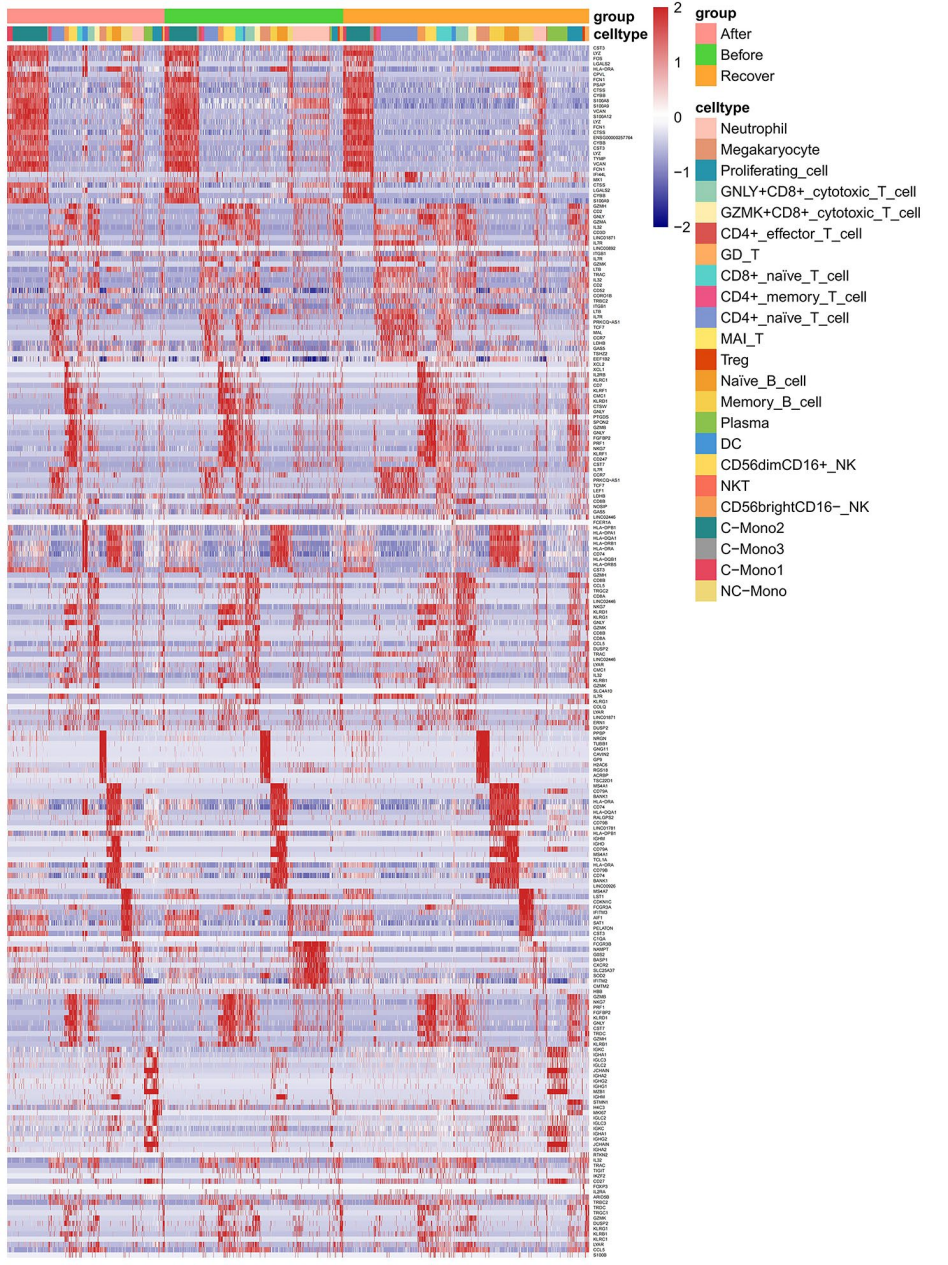
Genetic heat map of various subgroups of elderly hip fracture patients at different time points

**Fig. 3:**
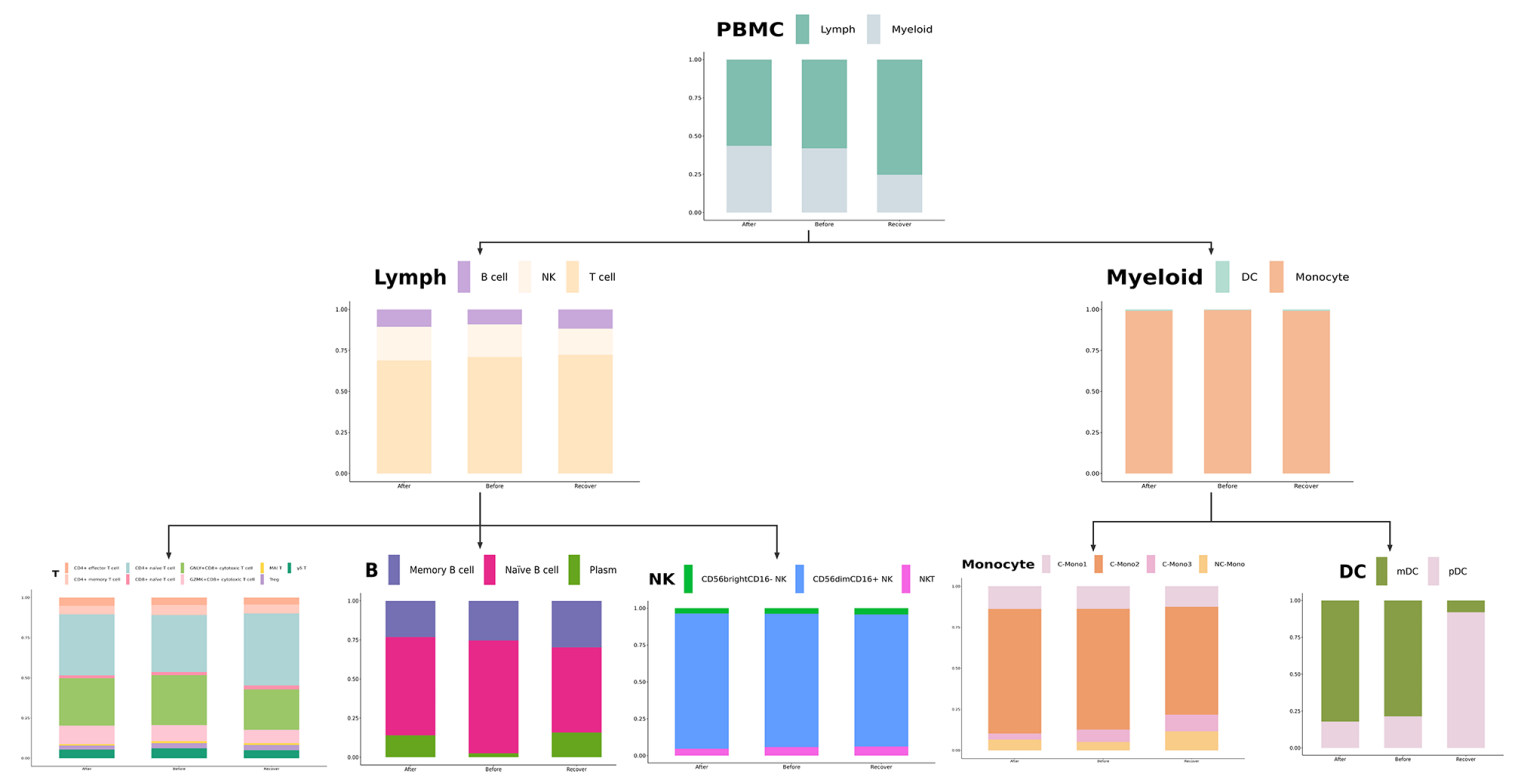
Bar plot of changes in the proportion of each cell subpopulation

Among lymphocyte subsets, T cells were further categorized into CD4^+^ T cells (CD4), CD8^+^ T cells (CD8A), γδ T cells (TRDC, TRGC2), mucosal-associated invariant T (MAIT) cells (NCR3, KLRB1, SLC4A10), and regulatory T (Treg) cells (FOXP3). CD4^+^ T cells were identified as Naive T cells (CCR7, SELL, S100A4), Memory T cells (GPR183, CD44), and Effector T cells (Teffc) (GNLY, GZMK), whereas CD8^+^ T cells were identified as Naive T cells (CCR7, SELL, S100A4) and Cytotoxic T cells (KLRD1, NKG7). We further characterized CD8^+^ cytotoxic T cells as GNLY^+^CD8^+^ cytotoxic T cells (GNLY) and GZMK^+^CD8^+^ cytotoxic T cells (GZMK). NK cells were also categorized as CD56^bright^CD16^−^NK cells (XCL1, TRDC, XCL2, FCER1G, TYROBP), CD56^dim^CD16^+^ NK cells (NCAM1, FCGR3A, FGFBP2, NKG7, CX3CR1), and NKT cells (CD3D).

Within the myeloid cell subsets, monocytes were categorized into two subclasses: classical CD14^+^ (CD14) and non-classical CD16^+^ (FCGR3A) groups. We conducted a further analyze of the CD14^+^ monocyte population and classified it into three subpopulations based on differences in their transcriptional profiles: C-Mono1, C-Mono2, and C-Mono3. C-Mono1 expresses high levels of MHC-II molecules (HLA-DPA1, HLA-DPB1, HLA-DQA1, HLA-DQB1, and HLA-DRA), C-Mono2 expresses high levels of S100 family genes, including high levels of S100A8, S100A9, and S100A12, while C-Mono3 expresses high levels of interferon-inducible protein-related genes (IFI44L, IFI6, IFIT2, IFIT3). Finally, dendritic cells (DC) were categorized into two subpopulations: myeloid dendritic cells (mDC) (ITGAX, ANPEP, CD33, ITGAM) and plasmacytoid dendritic cells (pDC) (IL3RA, CLEC4C, NRP1) (Fig. 2, Figure S1-S5).

### Dynamics of peripheral blood immune cells in elderly hip fracture patients at different time points

Our analysis revealed significant changes in immune cell composition at different stages of hip fracture (Fig. 3). The proportion of neutrophils continued to decrease over time (9.20%, 3.63%, 2.57%), possibly indicating the progression of the immune-inflammatory response. In myeloid cells, we observed a slight decrease in the proportion of monocytes and an increase in the proportion of dendritic cells in elderly hip fracture patients during the postoperative recovery period compared to 24 h post-trauma (32.14–21.48% and 0.12–0.82%, respectively). In addition, the percentage of monocytes in the peripheral blood increased significantly (38.98%) within 24 h post-operation and gradually decreased to the normal range (21.48%) on the day 7 post-operation. As previously mentioned, we identified four subpopulations of monocytes, with C-Mono2 being the most predominant. Its proportion significantly decreased during the recovery period to 65.66%, in contrast to 24 h post-trauma (73.53%) and 24 h post-operation (75.90%). In contrast, CD16^+^ monocytes showed a gradual increase throughout the post-traumatic immune-inflammatory response, which accounts for 6.59% and 11.64% at 24 h and day 7 post-operation compared to 24 h post-trauma (5.19%).

In terms of lymphocytes, we observed a significant decrease in the percentage of cytotoxic T cells (GNLY^+^CD8^+^ cytotoxic T cells, GZMK^+^CD8^+^ cytotoxic T cells) during the recovery period. The percentages on day 7 post-operation were 25.11% and 8.32%, respectively, compared to 24 h post-trauma (31.09%, 10.07%) and 24 h post-operation (29.61%, 11.47%). In contrast to cytotoxic T cells, the proportion of CD4^+^ naïve T cells progressively increased over time, with proportions of 35.47% at 24 h post-trauma, 37.99% at 24 h post-operation, and 44.79% at day 7 post-operation. In our study, the proportion of CD56^dim^CD16^+^ NK in NK cells varied over time (88.41%, 89.94%, 86.93%), accounting for the majority of NK cells.

B cell subsets were categorized as naïve B cells, memory B cells, and plasma cells. The proportion of naïve B cells in the total number of B cells during the recovery period was significantly lower (54.44%) compared with the preoperative period (72.16%). Additionally, the proportion of memory B cells initially decreased and then increased over time (25.34%, 23.18%, 29.75%). In our study, we found that the composition of PBMCs in elderly hip fracture patients underwent significant changes at different stages post-trauma.

### Dynamic differential expression of PBMCs in elderly hip fracture patients: comparing 24 h post-operation to 24 h post-Trauma

**Fig. 4:**
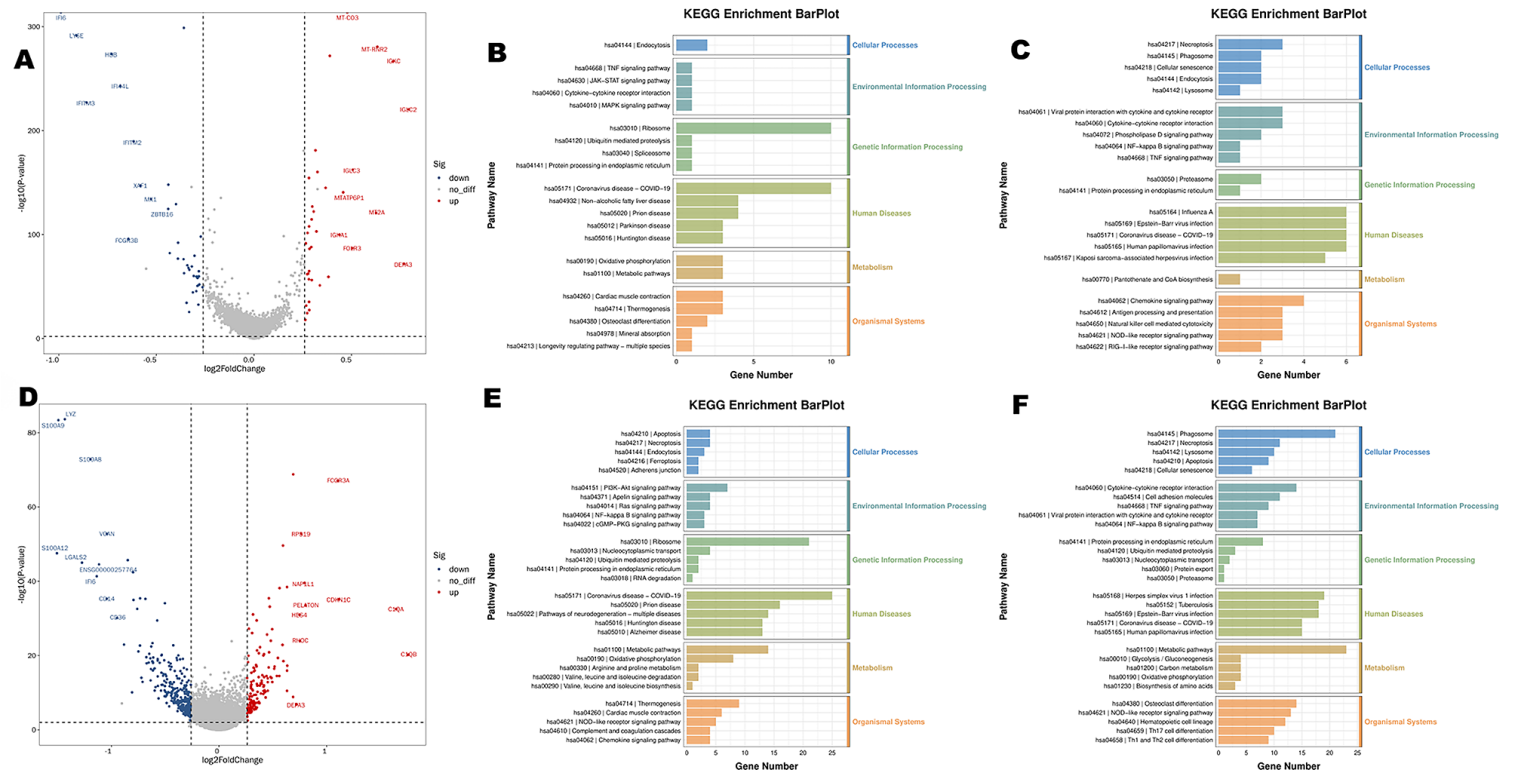
DEGs for C-Mono2 and NC-Mono at 24 h post-operation vs. 24 h post-trauma (**A**) Top 10 DEGs for C-Mono2 at 24 h post-operation vs. 24 h post-trauma (**B**) KEGG pathway enrichment analysis of DEGs upregulated by C-Mono2 (**C**) KEGG pathway enrichment analysis of DEGs downregulated by C-Mono2 (**D**) Top 10 DEGs for NC-Mono at 24 h post-operation vs. 24 h post-trauma (**E**) KEGG pathway enrichment analysis of DEGs upregulated by NC-Mono (**F**) KEGG pathway enrichment analysis of DEGs downregulated by NC-Mono.

In our study, we observed an up-regulation of mitochondria-encoded cytochrome genes (MT-CO3, MT-CO1, MT-CYB) in the C-Mono2 subpopulation during the first 24 h post-operation. Additionally, there was a down-regulation of interferon-related genes (IFI6, IFI44L, IFI27) and chemokine-related genes (CXCL-8, CXCL-2) in the same subpopulation during this time (Fig. 4A, Table S1). Pathway analysis indicated that the upregulated DEGs in the C-Mono2 were primarily enriched in the Oxidative phosphorylation pathway, while the downregulated DEGs were mainly associated with the Chemokine signaling pathway when compared to the status at 24 h post-trauma (Fig. 4B, C).

Furthermore, we observed the up-regulation of DEFA3 and SOCS3 in several subpopulations (Fig. 4A and Table S1). In contrast, we found a down-regulation of S100 family genes in the CD16^+^ monocyte subpopulation (Fig. 4D, Table S2). Enrichment analysis revealed that the genes downregulated in CD16^+^monocytes were significantly enriched in pathways such as osteoblast differentiation, hematopoietic cell lineage, cytokine-cytokine receptor interactions, NOD-like receptor signaling pathway, and IL-17 signaling pathway, (Fig. 4E, F). Additionally, we observed significant upregulation of C1Q A and C1Q B in CD16^+^ monocytes at 24 h post-operation (Fig. 4D & Table S2).

**Fig. 5:**
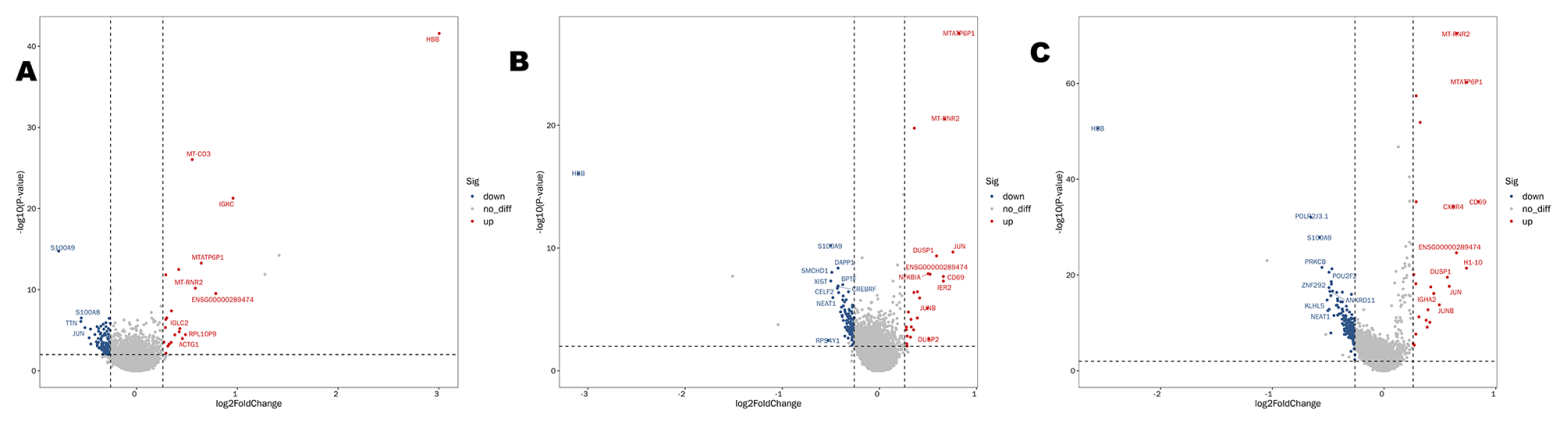
TOP 10 DEGs for Treg cell(**A**), Memory B cell(**B**), and Naive B cells(**C**) in day 7 post-operation vs. 24 h post-operation

In addition to monocyte subpopulations, other cell types also displayed interesting differential expression patterns. Treg cells exhibited significant upregulation of hemoglobin subunit-related genes (HBB, HBA1, HBA2) at 24 h post-operation, whereas memory B cells and naïve B cells showed the opposite trend (Fig. 5, Table S3-S5). Naïve B cells exhibited high expression of inflammation-related genes such as S100A8, JUN, and NFKBIA at 24 h post-operation. DEGs indicated that they were highly enriched in pathways related to osteoclast differentiation and the IL-17 signaling pathway. At 24 h post-operation, we also found high expression of DUSP1 in naïve B cells.

### Meaningful changes in the expression profiles of various cells subpopulations were observed within 7 days post-operation

**Fig. 6:**
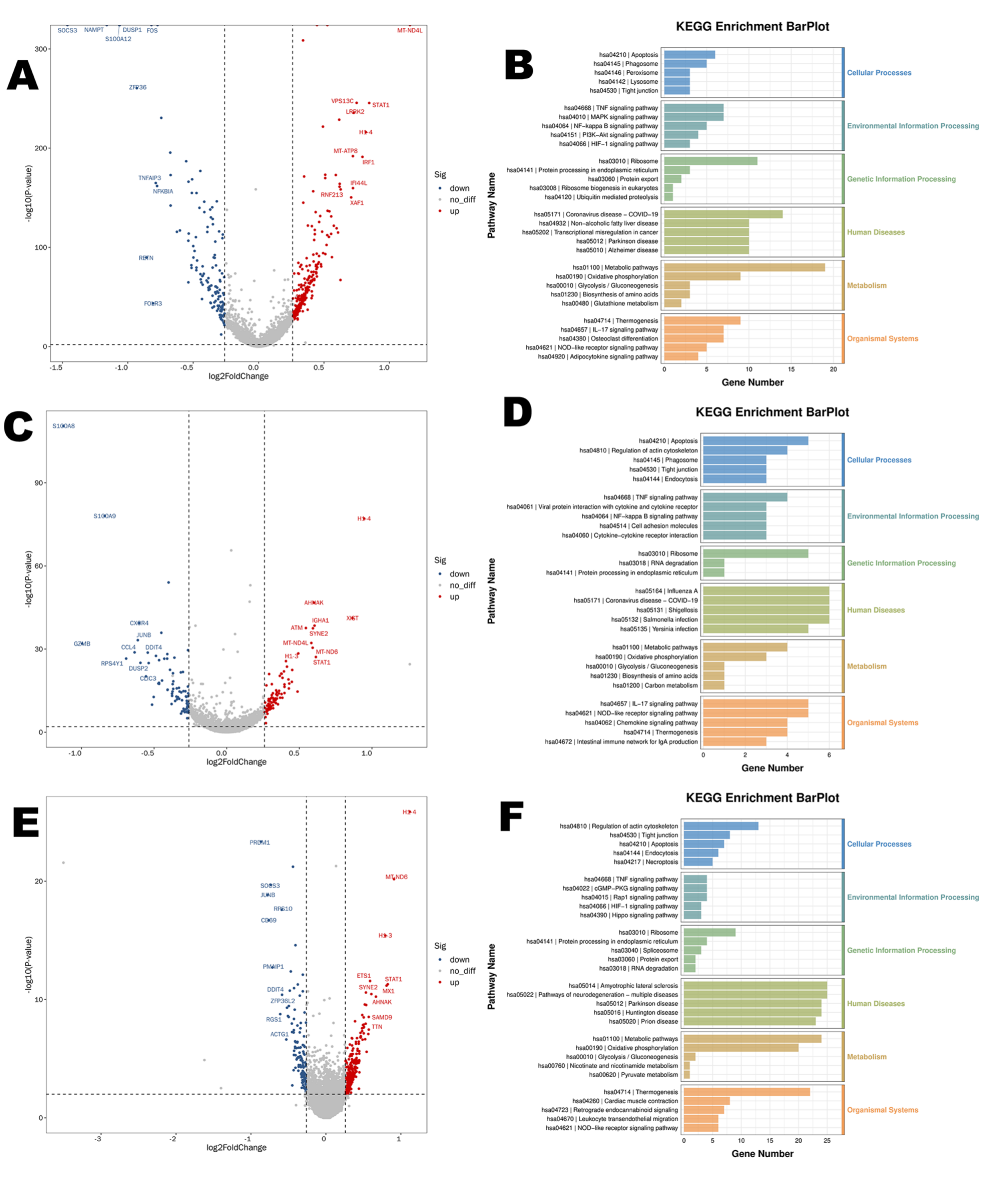
DEGs of monocyte subpopulations and T cell subpopulations at day 7 post-operation versus 24 h post-operation (**A**) Top 10 DEGs of C-Mono2 at day 7 post-operation VS 24 post-operation (**B**) KEGG pathway enrichment analysis of KEGG pathways of DEGs down-regulated by C-Mono2 (**C**) Top 10 DEGs of GZMK + CD8 + cytotoxic T cell at day 7 post-operation VS 24 post-operation DEGs compared (**D**) KEGG pathway enrichment analysis of DEGs down-regulated by GZMK + CD8 + cytotoxic T cel (**E**) Top 10 DEGs at day 7 post-operation VS 24 post-operation in Treg cell (**F**) KEGG pathway enrichment analysis of DEGs down-regulated by Treg cell

In C-Mono2, we observed an upregulation of DEFA3 expression at 24 h post-operation compared to preoperatively, followed by a decreasing trend at day 7 post-operation. Additionally, we noted a significant down-regulation of genes associated with inflammation and osteoclast differentiation (e.g., FOS, SOCS3, NAMPT, and TNFAIP3) in the C-Mono2 subpopulation at day 7 post-operation compared to 24 h post-operation [27–30] (Fig. 6A, Table S6). Pathway enrichment analysis showed that compared with 24 h post-operation, genes downregulated at day 7 post-operation were mainly enriched in oxidative phosphorylation, IL-17 signaling pathway, TNF signaling pathway, and osteoclast differentiation. This suggests a transition from the initial inflammatory response phase to the tissue healing phase [7] (Fig. 6B).

**Fig. 7:**
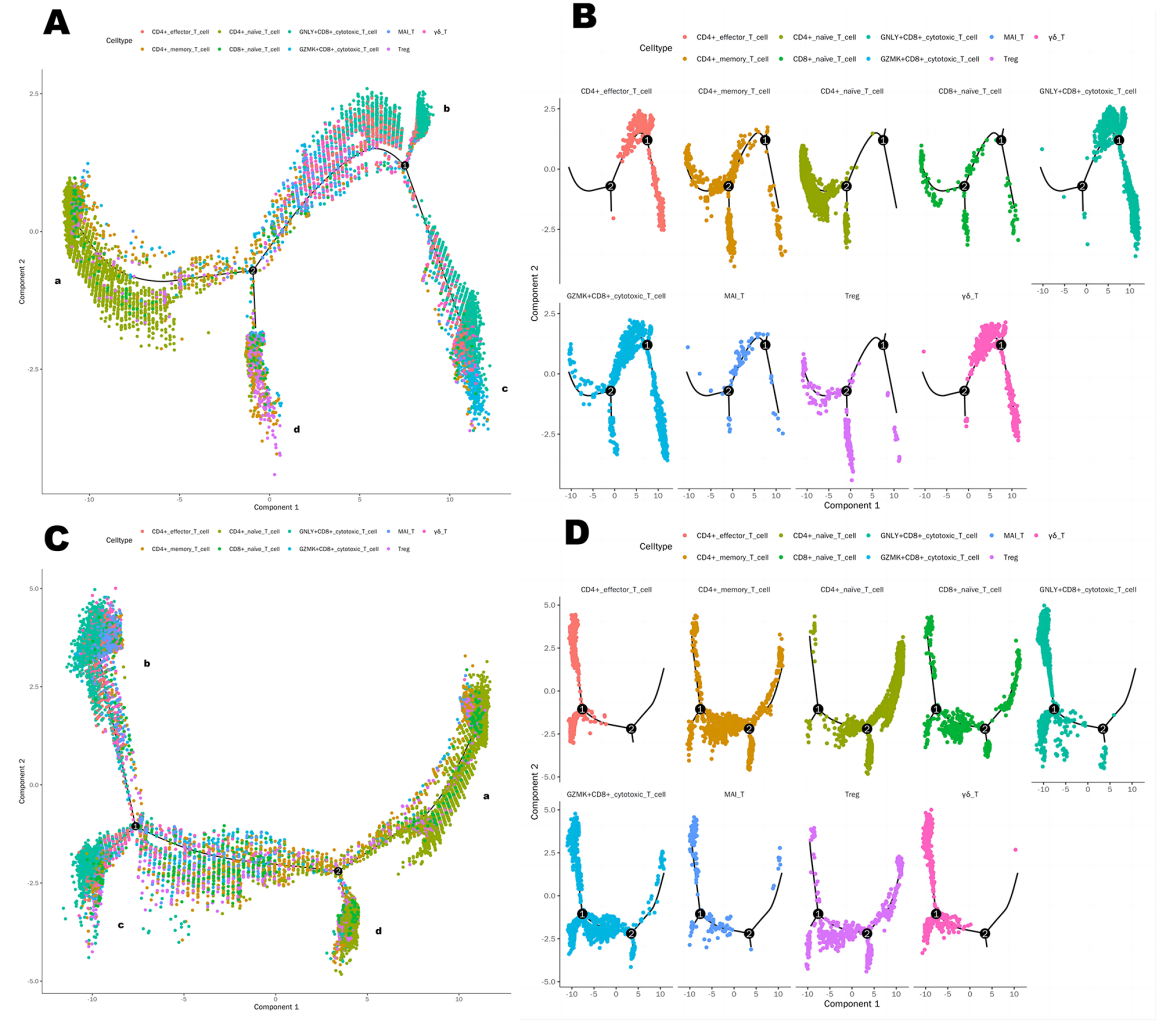
Pseudotime series analysis of CD4naive T cells at 24 h and day 7 post-operation

In the GZMK^+^CD8^+^ cytotoxic T-cell subset, we observed significant downregulation of chemokine-associated DEGs (including CCL3, CCL4, CCL5, and CXCR4) within 7 days post-operation. Previous studies have indicated that the downregulation of these DEGs may have a positive impact on fracture healing day 7 post-operation [31–33] (Fig. 6C, D, Table S7). Additionally, GZMB gene was upregulated at 24 h post-operation and displayed a downward trend at day 7 post-operation. Pathway enrichment analysis showed that the upregulated DEGs in the Treg cell subpopulation were enriched in the JAK/STAT signaling pathway at day 7 post-operation (Fig. 6E, F, Table S8), including JAK1, STAT1, etc. The CD4^+^Naive T cell subpopulation, the largest among T cells, exhibited significant downregulation of many DEGs associated with inflammation and positive regulation of osteoclasts, including S100 genes, chemokine-associated genes, SOCS3, TNFAIP3, FOS, JUN, among others. Pathway enrichment analysis indicated that these down-regulated genes were highly enriched in osteoclast differentiation and the IL-17 signaling pathway (Table S9). To better investigate the differentiation trajectory of T cell subsets, we performed a proposed time series analysis for T cell subsets at different time points. CD4^+^naive T cells were mainly located at the beginning of the predicted timeline trajectory GNLY^+^CD8^+^cytotoxic T cells were positioned immediately after CD4^+^naive T cells in (a)-(b), GZMK^+^CD8^+^cytotoxic T cells were localized at (a)-(c) while Treg cells were localized at another trend of pseudo-temporal endpoints (a)-(d) (Fig. 7A, B). In contrast to the postoperative period, the recovery Fate was divided into three branches, with CD4^+^naive T cells located mainly at the beginning of the predicted timeline trajectory, GNLY^+^CD8^+^cytotoxic T cells and GZMK^+^CD8^+^cytotoxic T cells positioned immediately after CD4^+ ^naive T cells at (a)-(b) and (a)-(c). While Treg cells, MAIT cells, and γδ T cell were localized in the other trend of the proposed chronological endpoints (a)-(d), while the other groups were in the figure throughout the development. Throughout the present study, this is the general transcriptional generalization state of each immune cell subpopulation in elderly hip fracture patients (Fig. 7C, D).

In summary, immune senescence, inflammation, and osteoimmunology are the three protagonists in the process of hip fracture in elderly patients. Further studies are crucial to improve our understanding of this complex process.

### Cellular interactions between PBMCs in different trauma immune states

**Fig. 8:**
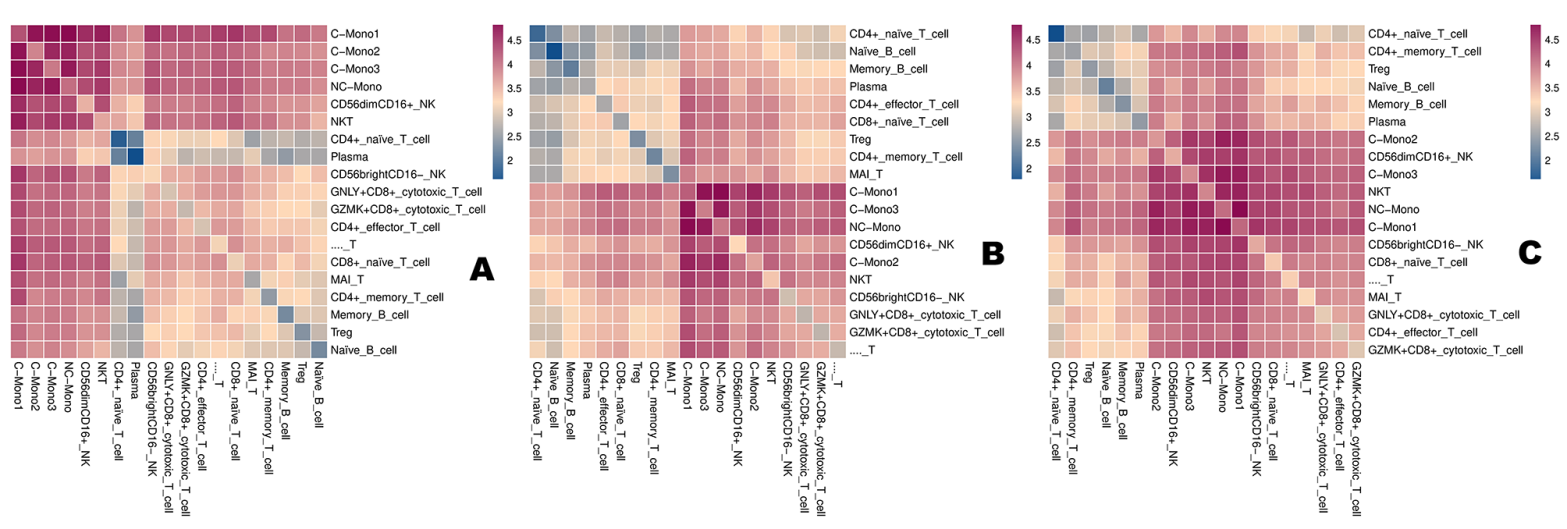
Crosstalk between monocytes, T cells, NK cells, and B cells. (**A-C**) Heat map showing key cellular interactions between the four cell types

To investigate the interaction of immune cells in different immune states post-trauma in elderly patients, we used CellPhone DB to assess the interaction between adult T cell subpopulations, B cell subpopulations, monocyte subpopulations, and NK cell subpopulations. We found that each subpopulation exhibited interactions with other subpopulations, and then applied a series of immune-related ligand-receptor pairs to investigate the L-R pairs involved in these cell-cell interactions (Fig. 8).

Within 24 h post-trauma, the C-Mono2 cell subpopulation interacted primarily within the monocyte subpopulation via TYROBP_CD44 and C5AR1_RPS19. The GZMK^+^CD8^+^ cytotoxic T cell subpopulation interacted with C-Mono2 via CCL5_CCR1, HLA-B_KIR3DL2, and HLA-C_FAM3C. Mono2 and NKT cells interacted with C-Mono1 via TYROBP_CD44, CCL5_CCR1, and HLA-C_FAM3C. Within 24 h post-operation, the GZMK^+^CD8^+^ cytotoxic T cell subpopulation interacted with C-Mono2 mainly through CCL5_CCR1, CCL5_CCR5, HLA-B_KIR3DL2, and with NC-Mono monocytes through C5AR1_RPS19, CCL5_CCR1, and CCL5_CCR5 interactions (Table S10-S13).

In the postoperative recovery phase, we observed significant interaction of CD56^dim^CD16^+^ NK cells with NC-Mono via C5AR1_RPS19, CCL5_CCR1, and HLA-C_FAM3C. Overall, these findings suggest that different immune cell subpopulations interact with each other during the immune response after trauma in elderly patients, which could provide a potential basis for novel therapeutic strategies in the future (Table S14-S15).

## Discussions

In the present study, we investigated the chronological changes in immune cells in the peripheral blood microenvironment of elderly hip fracture patients from 24 h post-trauma to day 7 post-operation at the single-cell transcriptional level. Our study suggests that variations in the expression of immune cells at different time points following trauma may, to some extent, serve as indicators of the immune status and disease progression in elderly hip fracture patients.

Several studies have shown that a massive immune-inflammatory response is induced within a short period of time after fracture, which may adversely affect the prognosis of elderly hip fracture patients [7, 21]. To validate the above hypothesis, we analyzed the differential gene expression of each immune cell subpopulation at different time points. Among them, monocytes are of particular interest because they express inflammatory genes mainly in PBMCs [34, 35]. In elderly hip fracture patients, the frequencies of postoperative monocyte increased significantly within 24 h, gradually returning to normal level by day 7, which is consistent with other findings [36]. C-Mono2 subsets exhibit high expression levels of S100 family genes, which are predominantly expressed by neutrophils and monocytes and play a crucial role in inflammation development [37]. The expression of these inflammatory genes triggers monocytes to release inflammatory mediators, further contributing to the initiation and progression of the inflammatory response [37, 38]. Within 24 h post-operation, the C-Mono2 subpopulation of monocytes exhibited upregulation of genes associated with mitochondrial function and oxidative phosphorylation pathways, while interferon-related and chemokine-related genes were downregulated. These findings suggest that the impact on the C-Mono2 subpopulation at 24 h post-operation may involve the regulation of cellular energy metabolism and anti-inflammatory responses. Additionally, the downregulation of S100 family genes in the CD16^+^ monocyte subpopulation is associated with enrichment in signaling pathways related to bone resorption and inflammation. This suggests that CD16^+^monocytes may play an important role in bone resorption and inflammatory processes and are associated with disorders of skeletal metabolism and immune responses. Furthermore, various subpopulations of T cells displayed distinct patterns of differential expression. The upregulation of hemoglobin subunit-related genes in Treg cells suggests a potential immunomodulatory response to the fracture healing process. Conversely, the trend of downregulation of related genes in CD8 T cell subpopulations may reflect their functional adjustments within the 24-hour postoperative period. It has been shown that GZMK^+^CD8^+^ cytotoxic T cells, which are considered to be the hallmark cells of immune senescence, are highly correlated with the inflammatory response, which is consistent with our findings [39]. Among the B-cell subpopulations, the proportion of naïve B-cells decreased during the postoperative recovery period, whereas the proportion of memory B-cells initially decreased and then increased, in line with the trend reported by Zhang et al [40]. As the largest subpopulation of NK cells in terms. In our study, CD56^dim^CD16^+^ NK cells showed a relatively small magnitude of variation in percentage at different stages, but were actively involved in cellular interactions [41]. These findings emphasize the significance of the immune system in fracture healing and the involvement of different cell types in regulating inflammatory and immune responses.

Of interest, the expression level of DEFA3 in the C-Mono2 subpopulation was upregulated at 24 h post-operation and gradually downregulated at day 7 post-operation. Studies have demonstrated that DEFA3 is a novel gene related to bone metabolism, but its exact mechanism of action is still awaiting further investigation [42]. In addition, at day 7 post-operation, inflammation-regulated genes and osteoclast differentiation genes showed a significant downregulation trend, indicating that the initial inflammatory response phase was gradually weakening and the tissue healing and repair phase began to gradual intensification. Pathway enrichment analysis showed that the down-regulated DEGs were mainly enriched in oxidative phosphorylation, IL-17 signaling pathway, TNF signaling pathway, and osteoclast differentiation pathway, suggesting that these biological pathways have important roles in the healing and repair process of elderly fracture patients [43, 44]. In both monocyte and T-cell subsets, a large number of genes characterizing inflammation-related and positive regulation of osteoclasts were downregulated, including S100 family genes, chemokine-related genes, SOCS3, TNFAIP3, FOS, JUN, etc. Pathway enrichment analysis showed that these downregulated genes were highly enriched in IL-17 signaling pathway, TNF signaling pathway, and osteoclast differentiation. This suggests that the inflammatory response of immune cell subsets is gradually diminished and osteoclast differentiation is regulated during the healing and repair phase in elderly fracture patients. Meanwhile, the upregulation of C1Q A and C1Q B expression in CD16^+^ monocytes suggested the influence of the somatic component C1q in elderly fracture patients, but the role it plays needs further investigation [45].

Above all, this study represents the first exploration of immune status changes in elderly hip fracture patients at various stages post-injury using scRNA-seq technology. Our investigation delved into the immune profiles of elderly hip fracture patients at multiple time points, providing a comprehensive understanding of cellular heterogeneity and alterations in gene expression. Our study underscored the intricate nature of the post-traumatic immune response by examining interactions among different immune cell subpopulations, which could show significant clinical relevance for tailoring interventions among HF elderly people.

However, it is crucial to acknowledge certain limitations in our research. Firstly, in an effort to eliminate the influence of underlying diseases, we specifically included elderly individuals (age > 75) devoid of any comorbidities as our study group. Such a population is quite rare, however, and thus we had only four elderly hip fracture patients in our dataset. Consequently, the limited sample size may not fully capture the heterogeneity present within the broader elderly population with hip fractures. Secondly, our study primarily focused on peripheral blood samples, and as such, our findings may not provide a complete representation of the immune microenvironment at the actual fracture site. Additionally, Third, we failed to sort out subclusters of major cells, such as megakaryocytes, proliferating cells, and neutrophils according to biomarkers from published studies.

## Conclusion

Our study provides the first landscape of immune cellular subclusters from the different stages of post-traumatic hip fracture in the elderly at the single-cell level. Our results suggest that each cell type has its own unique holistic and dynamic immune response in the pathogenesis and treatment of hip fractures in the elderly. Changes in DSGs expression of immune cell subsets at 24 h post-operation reflect an early response to injury and contribute to tissue repair and prevention of infection. The changes in DEGs expression of immune cell subpopulations at day 7 post-operation reflect an immune status that favors fracture healing and local tissue recovery.

Understanding the changes in immune system status and cell subpopulations in elderly hip fracture patients can provide a scientific basis for understanding the changes in immune system status and cell subpopulations in elderly hip fracture patients, which can be used for clinical diagnosis, prognostic assessment, and the development of personalized treatment plans. By monitoring the expression levels of inflammation- and osteogenesis/bone resorption-related genes in immune cell subpopulations at different time points, the degree of fracture healing and prognosis can be better assessed, which can be used as a reference for formulating personalized treatment plans. In addition, by targeting the expression of different genes in immune cell subpopulations at different time points, new therapeutic targets for promoting fracture healing and preventing complications can also be identified, thereby improving the effectiveness and safety of treatment.

### Electronic supplementary material

Below is the link to the electronic supplementary material.

Supplementary Material 1

Supplementary Material 2

Supplementary Material 3

Supplementary Material 4

Supplementary Material 5

Supplementary Material 6

Supplementary Material 7

Supplementary Material 8

Supplementary Material 9

Supplementary Material 10

Supplementary Material 11

Supplementary Material 12

Supplementary Material 13

Supplementary Material 14

Supplementary Material 15

Supplementary Material 16

## Data Availability

The datasets used and/or analysed during the current study are available from the corresponding author on reasonable request.

## Declarations

### Submission declaration and verification

This study was not considered for publication elsewhere and its publication was approved by all authors, with the implicit or explicit approval of the competent authorities.

## Abbreviations

DXA: Dual-energy X-ray absorptiometry
DAMPs: Damage-associated molecular patterns
PBMCs: Peripheral blood mononuclear cells
DEGs: Differentially expressed genes
GO: Gene Ontology
KEGG: Kyoto Encyclopedia of Genes and Genomes
Mono: Monocyte
NK: Natural killer
DC: Dendritic cells
mDC: Myeloid dendritic cells
pDC: Plasmacytoid dendritic cells
