# Supplementary material for: Decoding the immune landscape following hip fracture in elderly patients: unveiling temporal dynamics through single-cell RNA sequencing
Source: Immun Ageing. 2023 Oct 17;20:54. doi: 10.1186/s12979-023-00380-6 (PMC10580557; doi:10.1186/s12979-023-00380-6)
Supplement: Supplementary file 1 — Supplementary Material 1 [file 12979_2023_380_MOESM1_ESM.docx]

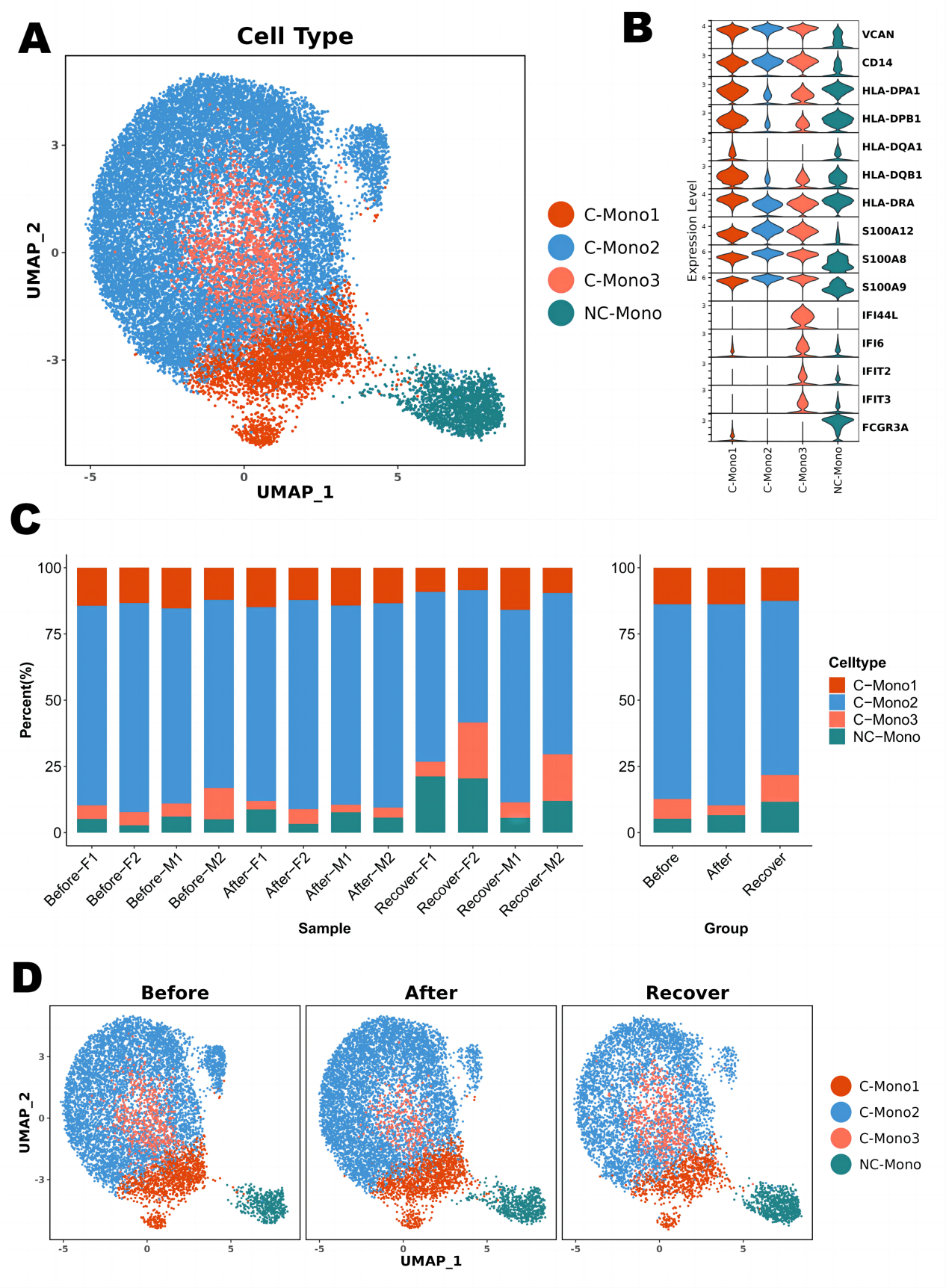


**Figure S1.** Clustering and categorization of cellular profiles in elderly hip fracture patients. (A) tSNE visualization of monocyte clusters in elderly hip fracture patients. (B) Vilin plot showing marker genes for each cell cluster. (C) Bar graph showing the relative percentage of cell clusters for each sample. (D) Visualization of tSNE of monocyte clusters in aging hip fracture patients at different time points.


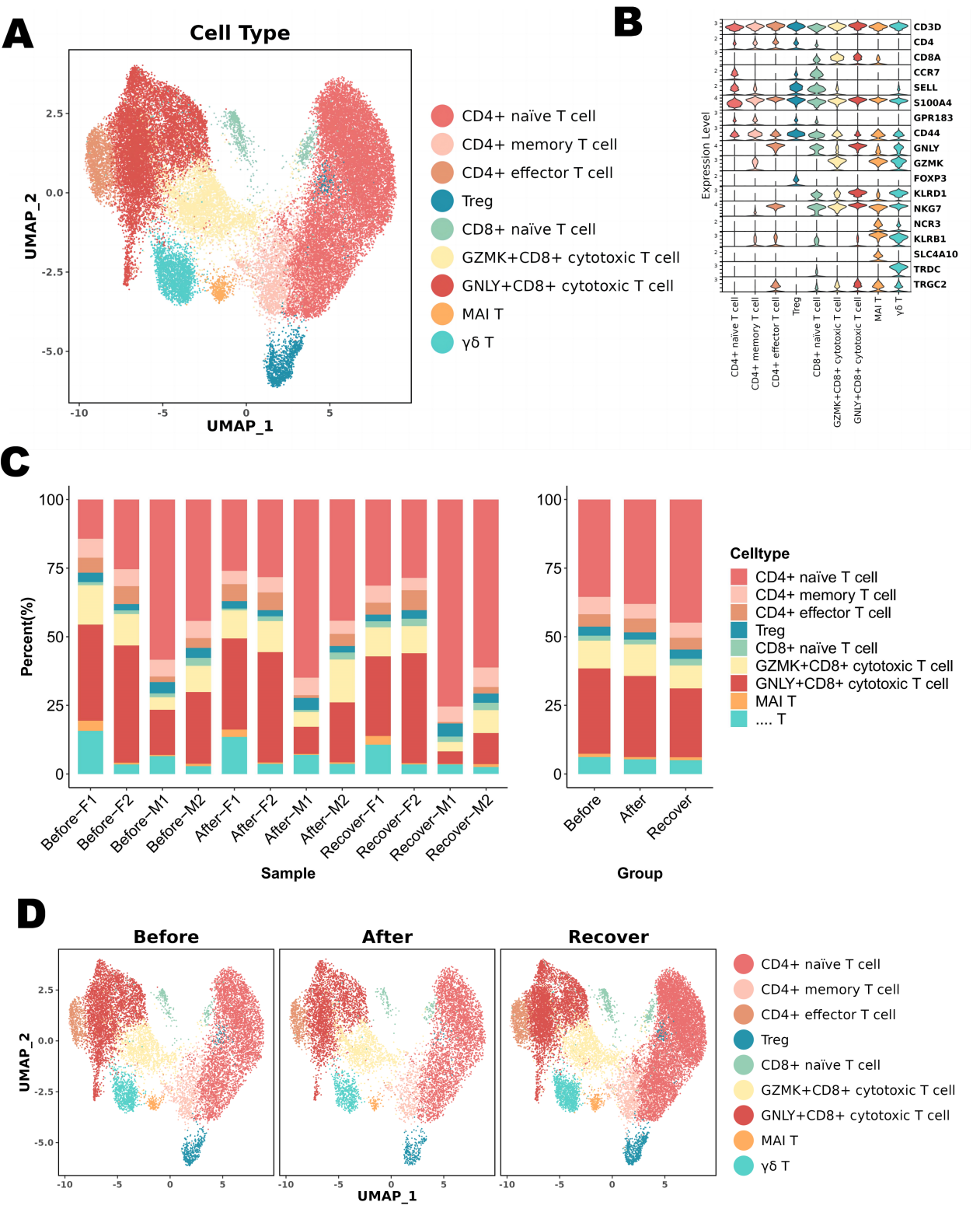


**Figure S2.** Clustering and categorization of cellular profiles in elderly hip fracture patients. (A) tSNE visualization of T cell clusters from elderly hip fracture patients. (B) Vilin plot showing marker genes for each cell cluster. (C) Bar graph showing the relative percentage of cell clusters for each sample. (D) Visualization of tSNE of T cell clusters in aging hip fracture patients at different time points.


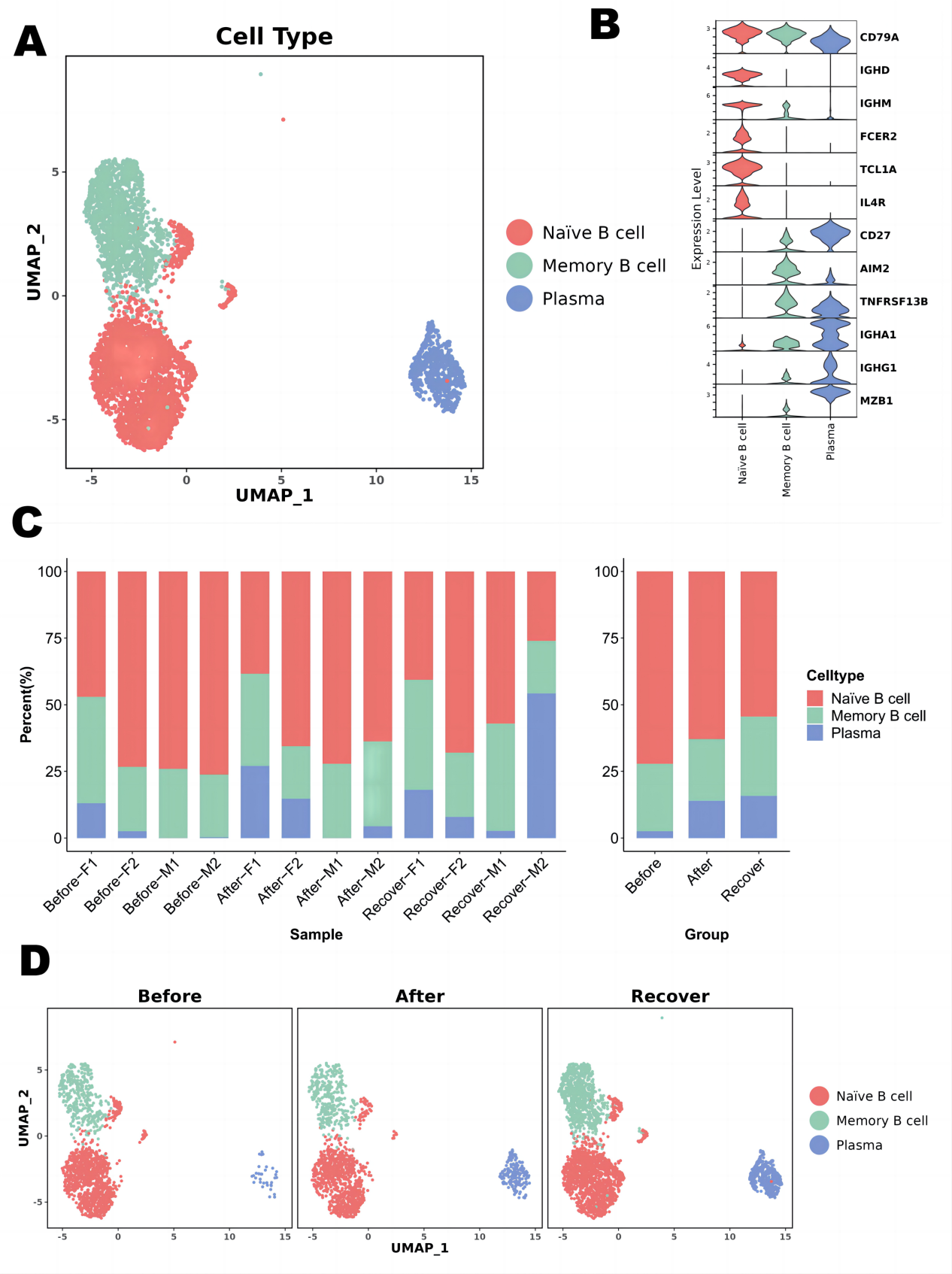


**Figure S3.** Clustering and categorization of cellular profiles in elderly hip fracture patients. (A) Visualization of tSNE of B-cell clusters from elderly hip fracture patients. (B) Vilin plot showing marker genes for each cell cluster. (C) Bar graph showing the relative percentage of cell clusters for each sample. (D) Visualization of tSNE of B-cell clusters in aging hip fracture patients at different time points.


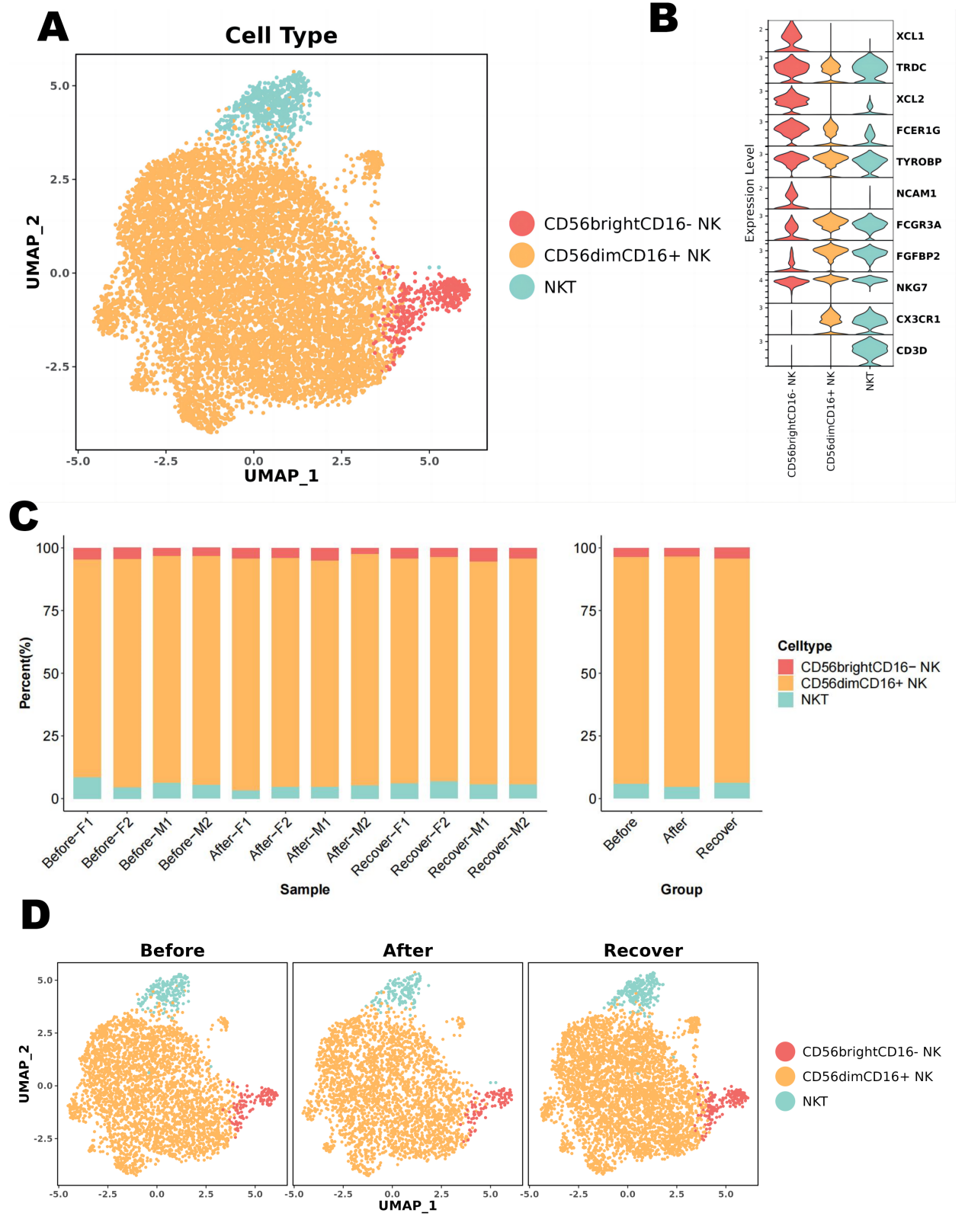


**Figure S4.** Clustering and categorization of cellular profiles in elderly hip fracture patients. (A) tSNE visualization of NK cell clusters in elderly hip fracture patients. (B) Vilin plot showing marker genes for each cell cluster. (C) Bar graph showing the relative percentage of cell clusters for each sample. (D) Visualization of tSNE of N cell clusters in aging hip fracture patients at different time points.


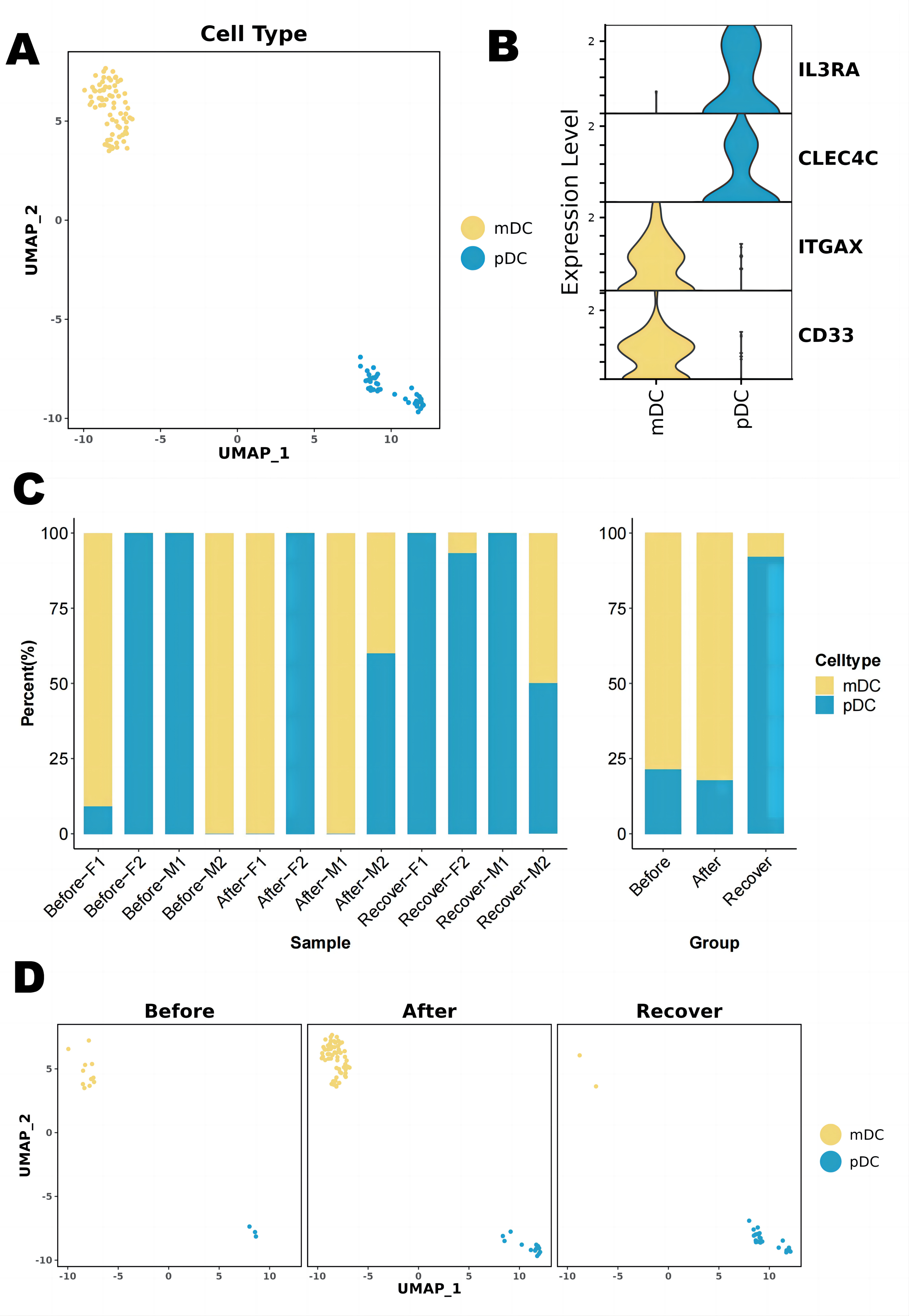


**Figure S5.** Clustering and categorization of cellular profiles in elderly hip fracture patients. (A) tSNE visualization of DC cell clusters in elderly hip fracture patients. (B) Vilin plot showing marker genes for each cell cluster. (C) Bar graph showing the relative percentage of cell clusters for each sample. (D) Visualization of tSNE of DC cell clusters in aging hip fracture patients at different time points.
