# Supplementary material for: Decoding the immune landscape following hip fracture in elderly patients: unveiling temporal dynamics through single-cell RNA sequencing
Source: Immun Ageing. 2023 Oct 17;20:54. doi: 10.1186/s12979-023-00380-6 (PMC10580557; doi:10.1186/s12979-023-00380-6)
Supplement: Supplementary file 2 — Supplementary Material 2 [file 12979_2023_380_MOESM2_ESM.docx]

**Supplementary Table 1.** TOP 100 DEGs in C-Mono2 (24h post-surgery vs. 24h post-trauma)

| **GeneName** | **log2FC** | **Pvlaue** | **Qvalue** |
| --- | --- | --- | --- |
| IGLC2 | 0.792985143 | 3.1936E-221 | 1.3122E-216 |
| DEFA3 | 0.770400189 | 1.52326E-72 | 6.25894E-68 |
| IGKC | 0.714808441 | 1.9244E-267 | 7.9072E-263 |
| MT-RNR2 | 0.632749704 | 1.9548E-281 | 8.0319E-277 |
| MT2A | 0.626137666 | 1.5636E-121 | 6.4246E-117 |
| IGLC3 | 0.506700447 | 3.5164E-163 | 1.4448E-158 |
| FOLR3 | 0.504270399 | 1.07874E-87 | 4.43245E-83 |
| MT-CO3 | 0.478951638 | 0 | 0 |
| MTATP6P1 | 0.457677904 | 2.1423E-141 | 8.8023E-137 |
| IGHA1 | 0.438039976 | 1.408E-100 | 5.78534E-96 |
| MT-CO1 | 0.389074019 | 1.0122E-272 | 4.1591E-268 |
| MIR23AHG | 0.382375484 | 4.55584E-60 | 1.87195E-55 |
| MT-CYB | 0.36756116 | 1.0277E-145 | 4.2226E-141 |
| SOCS3 | 0.33793285 | 7.28054E-52 | 2.9915E-47 |
| IGHG2 | 0.325921924 | 2.0968E-144 | 8.6157E-140 |
| RPS24 | 0.325672899 | 5.6468E-161 | 2.3202E-156 |
| IGHA2 | 0.320521645 | 8.2031E-104 | 3.37056E-99 |
| RPL39 | 0.315896822 | 9.1179E-182 | 3.7465E-177 |
| RPS4X | 0.306206206 | 6.3452E-123 | 2.6072E-118 |
| RPL32 | 0.299068471 | 9.9471E-128 | 4.0871E-123 |
| ZFP36 | 0.296300074 | 5.45631E-57 | 2.24194E-52 |
| MT-RNR1 | 0.295727269 | 2.2045E-115 | 9.058E-111 |
| RPL12 | 0.293961888 | 9.41086E-89 | 3.86683E-84 |
| RPL22 | 0.285107238 | 5.01108E-87 | 2.059E-82 |
| IL1R2 | 0.284088227 | 3.56908E-28 | 1.4665E-23 |
| HSPA8 | 0.283283885 | 1.32808E-65 | 5.45694E-61 |
| RPS12 | 0.282985493 | 1.4492E-108 | 5.9546E-104 |
| H1-10 | 0.282928622 | 5.3777E-36 | 2.20964E-31 |
| RPL34 | 0.282415417 | 3.4845E-155 | 1.4317E-150 |
| SNHG29 | 0.282088578 | 9.49586E-58 | 3.90176E-53 |
| RBM3 | 0.278144548 | 4.37763E-63 | 1.79872E-58 |
| RPS8 | 0.275018664 | 2.4346E-102 | 1.00035E-97 |
| EEF1B2 | 0.273006537 | 1.13338E-62 | 4.65694E-58 |
| ENSG00000289474 | 0.272919595 | 4.44349E-25 | 1.82578E-20 |
| FOSB | 0.268238199 | 2.28791E-32 | 9.40078E-28 |
| RPS3A | 0.26616377 | 2.8101E-92 | 1.15464E-87 |
| XIST | 0.263274831 | 1.09375E-18 | 4.4941E-14 |
| RPL10 | 0.257139061 | 5.82317E-72 | 2.39268E-67 |
| MT-CO2 | 0.255109736 | 2.2863E-181 | 9.3941E-177 |
| RPS13 | 0.24860795 | 2.10584E-80 | 8.65267E-76 |
| RPS6 | 0.246063422 | 1.90313E-66 | 7.81975E-62 |
| RPS23 | 0.245795043 | 1.21044E-77 | 4.97357E-73 |
| RPL6 | 0.244046452 | 2.15454E-63 | 8.8528E-59 |
| RPS7 | 0.240468158 | 6.67408E-72 | 2.74231E-67 |
| RPL8 | 0.239189124 | 1.56632E-73 | 6.43587E-69 |
| SNHG5 | 0.233598695 | 1.03502E-56 | 4.2528E-52 |
| MT-ND5 | 0.233496556 | 1.93221E-47 | 7.93927E-43 |
| ADAMTS2 | 0.233433848 | 2.2389E-46 | 9.1994E-42 |
| JCHAIN | 0.23275797 | 2.30554E-61 | 9.47324E-57 |
| RPL13 | 0.232192401 | 8.45794E-93 | 3.47528E-88 |
| RPLP0 | 0.230508037 | 5.56468E-42 | 2.28647E-37 |
| RPLP1 | 0.222236559 | 1.57638E-74 | 6.47718E-70 |
| EREG | 0.221419468 | 1.78599E-17 | 7.33844E-13 |
| RPL7 | 0.221233351 | 2.82344E-51 | 1.16012E-46 |
| RPS28 | 0.221022771 | 3.87459E-87 | 1.59203E-82 |
| RPL5 | 0.219157483 | 1.42944E-47 | 5.87342E-43 |
| RPS27A | 0.210339922 | 5.20149E-64 | 2.13724E-59 |
| RPL29 | 0.209600335 | 5.43143E-54 | 2.23172E-49 |
| HSP90AB1 | 0.208232421 | 4.20084E-43 | 1.72608E-38 |
| RPL21 | 0.200199015 | 5.99894E-61 | 2.4649E-56 |
| H1-4 | 0.195016982 | 1.91922E-26 | 7.88588E-22 |
| IGHG1 | 0.190875863 | 1.77696E-61 | 7.30134E-57 |
| ENSG00000288943 | 0.189683351 | 2.25439E-20 | 9.26306E-16 |
| DEFA1 | 0.188769633 | 5.77728E-23 | 2.37383E-18 |
| RPL26 | 0.186018382 | 1.64436E-43 | 6.75652E-39 |
| JUND | 0.185170944 | 6.76669E-25 | 2.78037E-20 |
| RPL24 | 0.18437657 | 6.72958E-43 | 2.76512E-38 |
| SLC2A3 | 0.183880125 | 4.84178E-20 | 1.98944E-15 |
| NFIL3 | 0.179864279 | 2.91798E-30 | 1.19897E-25 |
| CD163 | 0.1783865 | 6.4057E-30 | 2.63204E-25 |
| RACK1 | 0.176864704 | 4.1911E-35 | 1.72208E-30 |
| SLC25A6 | 0.176571986 | 4.41852E-33 | 1.81553E-28 |
| EIF3L | 0.175226559 | 5.79634E-25 | 2.38166E-20 |
| RPS3 | 0.174272145 | 2.99801E-43 | 1.23185E-38 |
| HMGN2 | 0.174238347 | 8.21959E-34 | 3.37735E-29 |
| PIM1 | 0.171926396 | 8.39252E-23 | 3.4484E-18 |
| RPS5 | 0.170933147 | 2.86783E-29 | 1.17836E-24 |
| RPL17 | 0.170479759 | 2.26704E-30 | 9.31505E-26 |
| RPL35A | 0.16944222 | 5.70643E-43 | 2.34472E-38 |
| HSP90B1 | 0.169061844 | 5.00189E-27 | 2.05523E-22 |
| RPS15A | 0.168381225 | 9.3437E-45 | 3.83923E-40 |
| MALAT1 | 0.168134654 | 1.93296E-36 | 7.94235E-32 |
| C4orf48 | 0.166724944 | 6.1204E-29 | 2.51481E-24 |
| RPL18 | 0.166630071 | 1.02547E-37 | 4.21357E-33 |
| RPL30 | 0.166185274 | 1.98942E-51 | 8.17432E-47 |
| RPL14 | 0.160760484 | 6.88857E-31 | 2.83045E-26 |
| ENSG00000279602 | 0.160517545 | 3.12877E-14 | 1.28558E-09 |
| EEF1D | 0.160497289 | 2.04359E-25 | 8.39693E-21 |
| ATP5F1D | 0.16016103 | 7.02203E-24 | 2.88528E-19 |
| RPL19 | 0.156760638 | 4.88388E-33 | 2.00674E-28 |
| DUSP1 | 0.156433382 | 7.99769E-17 | 3.28617E-12 |
| MT1E | 0.154333719 | 9.80672E-32 | 4.02948E-27 |
| RPL37 | 0.154307761 | 1.35631E-36 | 5.57292E-32 |
| MT-ND4 | 0.152684877 | 3.47143E-99 | 1.42638E-94 |
| MS4A6A | 0.152683011 | 8.18336E-22 | 3.36246E-17 |
| RPL3 | 0.152358888 | 3.14809E-27 | 1.29352E-22 |
| H1-3 | 0.150716423 | 1.47281E-19 | 6.05161E-15 |
| FDFT1 | 0.150443901 | 7.61629E-30 | 3.12946E-25 |
| PABPC4 | 0.148107257 | 7.72442E-25 | 3.17389E-20 |
| SNHG25 | 0.147205972 | 1.06226E-28 | 4.36472E-24 |
| TMEM123 | -0.194018255 | 1.89841E-32 | 7.80037E-28 |
| USP15 | -0.194854722 | 3.17824E-25 | 1.30591E-20 |
| CREBRF | -0.195830632 | 4.20052E-40 | 1.72595E-35 |
| ADAR | -0.195874797 | 7.7389E-33 | 3.17984E-28 |
| CD48 | -0.196109856 | 3.91038E-30 | 1.60673E-25 |
| RAC1 | -0.196455651 | 1.16953E-38 | 4.80549E-34 |
| RGS18 | -0.197713183 | 1.19324E-34 | 4.90289E-30 |
| YPEL3 | -0.197831402 | 1.83219E-32 | 7.5283E-28 |
| BLVRA | -0.197963319 | 2.36832E-31 | 9.73119E-27 |
| UBE2L6 | -0.198320194 | 3.1144E-32 | 1.27967E-27 |
| LUCAT1 | -0.198625519 | 1.51921E-24 | 6.24229E-20 |
| CCL5 | -0.200245688 | 4.52366E-32 | 1.85873E-27 |
| CYP1B1 | -0.201569079 | 8.2907E-18 | 3.40657E-13 |
| S100A11 | -0.201596838 | 5.425E-103 | 2.22908E-98 |
| TYROBP | -0.202957242 | 8.4137E-125 | 3.4571E-120 |
| CST3 | -0.20356444 | 3.8983E-56 | 1.60177E-51 |
| IFIT2 | -0.204762127 | 3.07348E-45 | 1.26286E-40 |
| PILRA | -0.205006474 | 1.26584E-43 | 5.20119E-39 |
| MS4A7 | -0.205432171 | 2.63751E-22 | 1.08373E-17 |
| JUN | -0.205522403 | 7.32825E-22 | 3.0111E-17 |
| HSPA6 | -0.20571983 | 5.61536E-42 | 2.3073E-37 |
| TMEM176A | -0.206711449 | 1.20925E-22 | 4.96869E-18 |
| ATP6V0B | -0.206879992 | 2.79804E-41 | 1.14969E-36 |
| SHISA5 | -0.209693894 | 2.63569E-39 | 1.08298E-34 |
| S100A4 | -0.212498858 | 4.54399E-93 | 1.86708E-88 |
| EGR1 | -0.212499801 | 2.27811E-35 | 9.36055E-31 |
| S100P | -0.215897558 | 1.12055E-43 | 4.60421E-39 |
| MYL12A | -0.217818831 | 4.00751E-40 | 1.64664E-35 |
| MX2 | -0.21922069 | 2.3976E-42 | 9.8515E-38 |
| ATP6V0E1 | -0.21969788 | 6.89223E-43 | 2.83195E-38 |
| CELF2 | -0.220974906 | 2.01332E-38 | 8.27254E-34 |
| KIAA0319L | -0.222688672 | 1.87302E-49 | 7.69606E-45 |
| ANKRD34B | -0.224079059 | 7.1557E-50 | 2.9402E-45 |
| HLA-E | -0.224293295 | 4.1296E-53 | 1.69681E-48 |
| OAS1 | -0.225006331 | 2.87365E-40 | 1.18075E-35 |
| WSB1 | -0.226426091 | 8.74144E-37 | 3.59177E-32 |
| HLA-B | -0.227162272 | 3.9089E-105 | 1.60612E-100 |
| NFKBIZ | -0.227370194 | 6.0061E-30 | 2.46785E-25 |
| CFD | -0.229602767 | 2.15159E-32 | 8.84066E-28 |
| TNFSF13B | -0.229860982 | 3.16547E-40 | 1.30066E-35 |
| CALM1 | -0.230023903 | 1.76737E-45 | 7.26196E-41 |
| SP110 | -0.231984725 | 4.9601E-42 | 2.03806E-37 |
| APOBEC3A | -0.232642658 | 3.51811E-32 | 1.44556E-27 |
| SLC38A2 | -0.232733226 | 8.12034E-45 | 3.33657E-40 |
| H3-3A | -0.233451092 | 4.8868E-116 | 2.0079E-111 |
| FCER1G | -0.233561105 | 1.59139E-39 | 6.53885E-35 |
| CD36 | -0.234791285 | 3.97952E-37 | 1.63515E-32 |
| HLA-A | -0.235704998 | 3.88237E-58 | 1.59523E-53 |
| EVI2A | -0.236372286 | 4.52765E-35 | 1.86037E-30 |
| LEPROTL1 | -0.236635184 | 9.19087E-60 | 3.77644E-55 |
| SMCHD1 | -0.23818182 | 1.47766E-36 | 6.07156E-32 |
| MT-TM | -0.24039432 | 2.92726E-68 | 1.20278E-63 |
| IFIT3 | -0.243371423 | 1.21227E-62 | 4.98108E-58 |
| CARD16 | -0.244472378 | 2.76281E-46 | 1.13521E-41 |
| LILRB1 | -0.244705684 | 8.98076E-42 | 3.69011E-37 |
| TMEM154 | -0.246214353 | 9.77207E-45 | 4.01525E-40 |
| HBA1 | -0.247379315 | 1.11632E-56 | 4.58684E-52 |
| GIMAP1 | -0.256653378 | 1.40972E-51 | 5.79241E-47 |
| IER2 | -0.259568688 | 4.44761E-38 | 1.82748E-33 |
| PREX1 | -0.263461835 | 4.67776E-50 | 1.92205E-45 |
| HLA-C | -0.27266626 | 8.62846E-99 | 3.54535E-94 |
| CXCR2 | -0.279746303 | 9.82232E-53 | 4.03589E-48 |
| OAS2 | -0.282163022 | 1.50982E-60 | 6.20368E-56 |
| CLEC12A | -0.282433357 | 1.60865E-65 | 6.60978E-61 |
| LGALS2 | -0.284628286 | 2.5539E-33 | 1.04937E-28 |
| TNFSF10 | -0.285159984 | 6.42116E-52 | 2.63839E-47 |
| EIF2AK2 | -0.288985884 | 6.28975E-59 | 2.58439E-54 |
| GIMAP7 | -0.289696336 | 2.20179E-51 | 9.04692E-47 |
| VNN2 | -0.291019683 | 2.23248E-60 | 9.17305E-56 |
| STAT1 | -0.29493097 | 2.28458E-60 | 9.38709E-56 |
| CLU | -0.295423042 | 2.77685E-46 | 1.14098E-41 |
| ITM2B | -0.305015401 | 2.29629E-80 | 9.43522E-76 |
| IFI44 | -0.308741604 | 6.51272E-61 | 2.67601E-56 |
| PLAC8 | -0.313035481 | 3.8621E-45 | 1.5869E-40 |
| CD63 | -0.318254813 | 4.91131E-70 | 2.01801E-65 |
| SIGLEC1 | -0.320039363 | 1.5722E-146 | 6.46E-142 |
| PSME2 | -0.330994026 | 7.59062E-67 | 3.11891E-62 |
| G0S2 | -0.332138327 | 3.06927E-26 | 1.26113E-21 |
| PSMB9 | -0.335705911 | 1.12711E-68 | 4.63116E-64 |
| RNF213 | -0.341630822 | 3.62021E-71 | 1.48751E-66 |
| CXCL8 | -0.34530285 | 4.39311E-35 | 1.80509E-30 |
| IRF1 | -0.35795208 | 1.41878E-63 | 5.82962E-59 |
| B2M | -0.35913581 | 1.4589E-299 | 5.9943E-295 |
| GIMAP4 | -0.360761531 | 5.02013E-77 | 2.06272E-72 |
| ISG15 | -0.388625817 | 6.6103E-93 | 2.71611E-88 |
| RNASE2 | -0.389439093 | 1.37058E-77 | 5.63157E-73 |
| EPSTI1 | -0.399587535 | 5.3091E-130 | 2.1815E-125 |
| RHOB | -0.431721944 | 6.60164E-83 | 2.71255E-78 |
| HBA2 | -0.438893729 | 9.2215E-149 | 3.789E-144 |
| ZBTB16 | -0.439630988 | 2.2344E-125 | 9.1811E-121 |
| MX1 | -0.527824919 | 6.1149E-135 | 2.5126E-130 |
| IFI27 | -0.552640887 | 7.27004E-68 | 2.98719E-63 |
| XAF1 | -0.582076753 | 7.9816E-148 | 3.2795E-143 |
| IFITM2 | -0.616532986 | 1.8489E-190 | 7.5971E-186 |
| FCGR3B | -0.643539729 | 1.26214E-96 | 5.18601E-92 |
| IFI44L | -0.685005558 | 1.1264E-243 | 4.6282E-239 |
| HBB | -0.728977097 | 2.5253E-274 | 1.0376E-269 |
| IFITM3 | -0.858134866 | 7.0667E-228 | 2.9036E-223 |
| LY6E | -0.909644196 | 3.1355E-292 | 1.2884E-287 |
| IFI6 | -0.989624058 | 0 | 0 |
