# Supplementary material for: Decoding the immune landscape following hip fracture in elderly patients: unveiling temporal dynamics through single-cell RNA sequencing
Source: Immun Ageing. 2023 Oct 17;20:54. doi: 10.1186/s12979-023-00380-6 (PMC10580557; doi:10.1186/s12979-023-00380-6)
Supplement: Supplementary file 3 — Supplementary Material 3 [file 12979_2023_380_MOESM3_ESM.docx]

**Supplementary Table 2.** Top 100 DEGs in CD16^+^ Monocyte (24h post-surgery vs. 24h post-trauma)

| **GeneName** | **log2FC** | **Pvlaue** | **Qvalue** |
| --- | --- | --- | --- |
| C1QB | 1.759796518 | 5.72766E-21 | 2.35344E-16 |
| C1QA | 1.645784053 | 2.92256E-33 | 1.20085E-28 |
| CDKN1C | 1.114932091 | 7.30788E-36 | 3.00274E-31 |
| FCGR3A | 1.102832712 | 9.18503E-68 | 3.77404E-63 |
| PELATON | 0.803724216 | 3.25053E-34 | 1.33561E-29 |
| NAP1L1 | 0.794275361 | 2.7232E-40 | 1.11893E-35 |
| RPS19 | 0.763038585 | 1.72911E-53 | 7.10476E-49 |
| RHOC | 0.750607353 | 1.17052E-24 | 4.80956E-20 |
| HES4 | 0.747751165 | 1.08944E-31 | 4.47639E-27 |
| DEFA3 | 0.719954636 | 1.53614E-07 | 0.00631183 |
| MT-CO3 | 0.689130846 | 1.62349E-69 | 6.67075E-65 |
| MT2A | 0.688023099 | 1.41963E-09 | 5.83312E-05 |
| MT-RNR2 | 0.633689375 | 5.29994E-18 | 2.17769E-13 |
| IGLC2 | 0.631755492 | 1.22358E-16 | 5.02757E-12 |
| LST1 | 0.631591974 | 3.55118E-39 | 1.45914E-34 |
| C1QC | 0.63143575 | 3.14491E-11 | 1.29221E-06 |
| MS4A7 | 0.59793411 | 3.12669E-19 | 1.28473E-14 |
| MT-CO1 | 0.59438378 | 2.45928E-50 | 1.01049E-45 |
| MTATP6P1 | 0.590166884 | 1.3732E-23 | 5.64233E-19 |
| MT-CO2 | 0.562067234 | 6.86489E-39 | 2.82071E-34 |
| TCF7L2 | 0.55953295 | 3.15125E-15 | 1.29482E-10 |
| CHST2 | 0.546650529 | 4.50811E-20 | 1.85234E-15 |
| MTSS1 | 0.537356972 | 3.12772E-15 | 1.28515E-10 |
| IGKC | 0.515425765 | 9.00889E-18 | 3.70166E-13 |
| TMPO | 0.512443868 | 3.51652E-17 | 1.4449E-12 |
| PECAM1 | 0.494126661 | 1.34238E-13 | 5.5157E-09 |
| MT-CYB | 0.493175123 | 7.89513E-28 | 3.24403E-23 |
| HMOX1 | 0.486744515 | 1.05434E-14 | 4.33216E-10 |
| MT-ATP6 | 0.480667338 | 1.02709E-24 | 4.22023E-20 |
| XIST | 0.478427673 | 6.36095E-15 | 2.61365E-10 |
| RPL8 | 0.471424434 | 7.05686E-34 | 2.89959E-29 |
| IGLC3 | 0.46521116 | 1.34403E-21 | 5.52248E-17 |
| RPS4X | 0.462975068 | 3.79713E-36 | 1.5602E-31 |
| VMO1 | 0.461085001 | 1.55215E-17 | 6.37763E-13 |
| EVL | 0.452472803 | 1.43382E-14 | 5.8914E-10 |
| SOD1 | 0.452450496 | 3.01279E-11 | 1.23792E-06 |
| PPDPF | 0.452182956 | 1.442E-13 | 5.92502E-09 |
| COTL1 | 0.445867534 | 2.17085E-26 | 8.91979E-22 |
| PABPC4 | 0.441037891 | 3.26331E-15 | 1.34086E-10 |
| TUBA1B | 0.432515669 | 1.97295E-09 | 8.10667E-05 |
| ARL4A | 0.429216155 | 5.4215E-11 | 2.22764E-06 |
| CTSC | 0.426219536 | 7.01974E-11 | 2.88434E-06 |
| CHCHD10 | 0.423206781 | 2.3908E-11 | 9.82357E-07 |
| LPL | 0.418760474 | 6.45306E-15 | 2.6515E-10 |
| UQCRB | 0.417579014 | 4.13619E-21 | 1.69952E-16 |
| NOP53 | 0.416886027 | 1.10755E-12 | 4.55082E-08 |
| DRAP1 | 0.413622851 | 3.47466E-11 | 1.4277E-06 |
| SLC25A6 | 0.413591985 | 2.43016E-12 | 9.9853E-08 |
| NACA | 0.412204743 | 9.63602E-21 | 3.95934E-16 |
| LTB | 0.411365941 | 2.09631E-10 | 8.61353E-06 |
| PTPN6 | 0.4018675 | 1.89504E-15 | 7.78653E-11 |
| CDH23 | 0.396537503 | 4.05359E-13 | 1.66558E-08 |
| EEF1B2 | 0.396462627 | 2.40692E-13 | 9.8898E-09 |
| LINC02432 | 0.395620904 | 5.80718E-14 | 2.38611E-09 |
| MALAT1 | 0.39448707 | 3.65378E-13 | 1.5013E-08 |
| CSF1R | 0.388641758 | 1.60485E-10 | 6.59419E-06 |
| HMGN2 | 0.386091295 | 3.04168E-13 | 1.2498E-08 |
| RPL10 | 0.380560053 | 5.51164E-23 | 2.26468E-18 |
| RPL10P9 | 0.377501723 | 1.14407E-12 | 4.70085E-08 |
| BID | 0.374900174 | 6.40278E-08 | 0.002630837 |
| FMNL2 | 0.369428537 | 2.34019E-21 | 9.6156E-17 |
| PAG1 | 0.368360617 | 2.72505E-08 | 0.001119697 |
| SAT1 | 0.367358195 | 6.47128E-15 | 2.65898E-10 |
| GNG2 | 0.3618301 | 5.17908E-08 | 0.002128033 |
| SPN | 0.357709914 | 5.96676E-11 | 2.45168E-06 |
| SIDT2 | 0.355634189 | 1.22897E-12 | 5.04973E-08 |
| SNHG8 | 0.354665343 | 3.90805E-09 | 0.000160578 |
| AIF1 | 0.352910167 | 4.29714E-22 | 1.76565E-17 |
| ADGRE1 | 0.351926 | 4.5914E-13 | 1.88656E-08 |
| HSPA8 | 0.35132033 | 1.00593E-06 | 0.041332736 |
| EEF1A1 | 0.349824725 | 3.43311E-30 | 1.41063E-25 |
| RPL10A | 0.348611669 | 8.18003E-18 | 3.36109E-13 |
| BCL2A1 | 0.343844525 | 9.35778E-07 | 0.038450172 |
| RPL6 | 0.343718297 | 3.5575E-18 | 1.46174E-13 |
| SLC25A5 | 0.343001313 | 5.55577E-08 | 0.002282811 |
| SIGLEC10 | 0.342877534 | 2.47342E-06 | 0.101630405 |
| BCAT1 | 0.34261911 | 3.64998E-10 | 1.49974E-05 |
| ETS2 | 0.335035559 | 1.88295E-06 | 0.077368703 |
| CKB | 0.331425993 | 1.17142E-16 | 4.81323E-12 |
| VASP | 0.33098913 | 8.51704E-08 | 0.003499566 |
| RPL22L1 | 0.330565999 | 9.10943E-08 | 0.003742973 |
| CXCL16 | 0.328788712 | 2.93479E-07 | 0.012058768 |
| NKTR | 0.327071382 | 3.40332E-06 | 0.139839009 |
| RPL14 | 0.326150889 | 2.37728E-17 | 9.76799E-13 |
| RPL17 | 0.325719862 | 1.08314E-07 | 0.00445051 |
| RPL36A | 0.325650492 | 4.03805E-10 | 1.65919E-05 |
| HSP90AB1 | 0.32331453 | 5.13414E-08 | 0.002109567 |
| RPL12 | 0.323156131 | 1.82482E-16 | 7.49799E-12 |
| UBC | 0.321863477 | 2.72917E-08 | 0.001121388 |
| CNIH4 | 0.321737452 | 1.72184E-08 | 0.000707488 |
| RPS6 | 0.320598544 | 1.88129E-17 | 7.73005E-13 |
| UICLM | 0.319690341 | 6.15507E-14 | 2.52906E-09 |
| GPI | 0.317052735 | 4.59842E-07 | 0.018894446 |
| TAGLN | 0.315737523 | 6.71226E-08 | 0.002758001 |
| SVIL | 0.315286622 | 1.10491E-07 | 0.004539944 |
| RPS23 | 0.314819681 | 8.305E-32 | 3.41244E-27 |
| RPL3 | 0.312512637 | 2.41701E-11 | 9.93124E-07 |
| KLF2 | 0.310182682 | 4.42638E-08 | 0.001818755 |
| ERICH1 | 0.308579643 | 1.65153E-06 | 0.067859578 |
| UNC119 | 0.30729243 | 1.23972E-11 | 5.09388E-07 |
| NAIP | -0.417518596 | 2.37758E-09 | 9.76924E-05 |
| AQP9 | -0.420454638 | 6.44013E-08 | 0.002646184 |
| TPP1 | -0.420802768 | 1.52689E-13 | 6.27382E-09 |
| METTL9 | -0.425479758 | 6.86885E-12 | 2.82234E-07 |
| SRGN | -0.425959406 | 1.75903E-23 | 7.22767E-19 |
| GCA | -0.426162652 | 1.82865E-11 | 7.51375E-07 |
| H2AC6 | -0.42687546 | 2.2977E-08 | 0.000944104 |
| AHR | -0.435831291 | 2.91254E-09 | 0.000119673 |
| OAS2 | -0.436896807 | 5.55763E-11 | 2.28358E-06 |
| MGST1 | -0.436975265 | 5.24191E-13 | 2.15385E-08 |
| NCF4 | -0.437738639 | 3.69406E-12 | 1.51785E-07 |
| JAML | -0.438511632 | 4.98999E-11 | 2.05034E-06 |
| IL6R | -0.43916637 | 2.05987E-10 | 8.46382E-06 |
| PTPRE | -0.440651639 | 1.2068E-10 | 4.95862E-06 |
| JUN | -0.442249008 | 1.52393E-08 | 0.000626168 |
| SIGLEC1 | -0.442787922 | 1.04499E-17 | 4.29376E-13 |
| CREB5 | -0.445182597 | 6.78808E-10 | 2.78915E-05 |
| CKLF | -0.44725782 | 1.09916E-10 | 4.51635E-06 |
| APLP2 | -0.45004967 | 1.56033E-18 | 6.41124E-14 |
| CD163 | -0.451242923 | 1.42144E-13 | 5.84055E-09 |
| TALDO1 | -0.452376573 | 5.4454E-17 | 2.23746E-12 |
| MLXIP | -0.454189307 | 3.86862E-13 | 1.58958E-08 |
| PLP2 | -0.459831179 | 1.95807E-13 | 8.0455E-09 |
| H4C3 | -0.460194163 | 1.79814E-12 | 7.38839E-08 |
| GIMAP4 | -0.463255654 | 6.50691E-18 | 2.67363E-13 |
| CYP1B1 | -0.463653424 | 4.76376E-07 | 0.01957382 |
| HLA-A | -0.468864572 | 6.78677E-19 | 2.78861E-14 |
| PLSCR1 | -0.469681305 | 8.15587E-11 | 3.35117E-06 |
| STAT1 | -0.473653212 | 7.26072E-11 | 2.98336E-06 |
| SORL1 | -0.483044985 | 1.54612E-10 | 6.35284E-06 |
| TMEM176B | -0.484298454 | 2.41552E-14 | 9.92512E-10 |
| NIBAN1 | -0.487834723 | 4.67758E-10 | 1.92197E-05 |
| PPBP | -0.487935943 | 6.01926E-08 | 0.002473256 |
| RAB32 | -0.491212231 | 3.67151E-15 | 1.50859E-10 |
| BNIP3L | -0.491554917 | 1.80321E-13 | 7.4092E-09 |
| NFKBIA | -0.495667811 | 1.05449E-09 | 4.3328E-05 |
| RNASE6 | -0.496269358 | 6.57278E-11 | 2.70069E-06 |
| PTAFR | -0.497589808 | 1.22206E-15 | 5.02134E-11 |
| TNFSF10 | -0.502749785 | 1.63256E-12 | 6.70802E-08 |
| NEAT1 | -0.503258311 | 8.31206E-35 | 3.41534E-30 |
| BTG2 | -0.505871138 | 9.35174E-10 | 3.84254E-05 |
| HLA-DMB | -0.506089684 | 2.45315E-11 | 1.00797E-06 |
| GNLY | -0.509065665 | 6.71342E-14 | 2.75848E-09 |
| CTSD | -0.509945143 | 1.8216E-19 | 7.48478E-15 |
| ZFP36L1 | -0.516591758 | 8.99391E-16 | 3.69551E-11 |
| CLU | -0.520277647 | 2.74424E-15 | 1.12758E-10 |
| EPSTI1 | -0.524364703 | 7.51231E-13 | 3.08673E-08 |
| PPT1 | -0.533361228 | 6.21423E-21 | 2.55336E-16 |
| SLC38A2 | -0.534566189 | 7.71231E-13 | 3.16891E-08 |
| TNFSF13B | -0.536207603 | 4.02821E-16 | 1.65515E-11 |
| FPR1 | -0.53625742 | 9.13012E-21 | 3.75147E-16 |
| SELL | -0.54020599 | 2.76099E-11 | 1.13446E-06 |
| CTSA | -0.552114082 | 8.78814E-19 | 3.61096E-14 |
| PLAC8 | -0.562251386 | 3.45347E-09 | 0.000141899 |
| MX1 | -0.568672873 | 1.54912E-12 | 6.36518E-08 |
| ACTN1 | -0.570696723 | 4.15627E-15 | 1.70777E-10 |
| BLVRB | -0.571190793 | 8.87989E-20 | 3.64866E-15 |
| KCTD12 | -0.571278545 | 2.13296E-19 | 8.76413E-15 |
| FOS | -0.57522047 | 3.54206E-30 | 1.4554E-25 |
| PLBD1 | -0.591298457 | 3.04585E-12 | 1.25151E-07 |
| RNASE2 | -0.599361716 | 2.70648E-11 | 1.11207E-06 |
| CCR2 | -0.59941142 | 2.94656E-17 | 1.21071E-12 |
| KLF10 | -0.603494606 | 3.37186E-17 | 1.38546E-12 |
| ZFP36L2 | -0.612007119 | 3.39549E-20 | 1.39517E-15 |
| ISG15 | -0.61409477 | 9.68023E-19 | 3.97751E-14 |
| TAGLN2 | -0.614988759 | 1.07047E-16 | 4.39844E-12 |
| MEGF9 | -0.616497495 | 4.55951E-19 | 1.87346E-14 |
| LY6E | -0.61866343 | 4.48257E-24 | 1.84184E-19 |
| CLEC4E | -0.62099393 | 3.60624E-19 | 1.48177E-14 |
| SYK | -0.630443471 | 3.68665E-23 | 1.51481E-18 |
| CD93 | -0.633528732 | 4.23538E-17 | 1.74028E-12 |
| TNFAIP3 | -0.636714303 | 4.96081E-14 | 2.03835E-09 |
| EVI2A | -0.638514348 | 5.7326E-20 | 2.35547E-15 |
| IER2 | -0.64924563 | 3.23988E-21 | 1.33123E-16 |
| XAF1 | -0.654720499 | 7.74882E-19 | 3.18391E-14 |
| CSF3R | -0.673109796 | 1.2148E-18 | 4.99149E-14 |
| CD99 | -0.676241907 | 2.79321E-20 | 1.1477E-15 |
| FCN1 | -0.684753549 | 5.68342E-36 | 2.33526E-31 |
| EGR1 | -0.711811781 | 1.22817E-13 | 5.04643E-09 |
| APOBEC3A | -0.722318468 | 9.7057E-15 | 3.98798E-10 |
| HBB | -0.734438492 | 3.99756E-36 | 1.64256E-31 |
| CAPG | -0.735466433 | 2.05217E-23 | 8.43216E-19 |
| CD63 | -0.749934927 | 8.0417E-22 | 3.30426E-17 |
| GPX1 | -0.760998418 | 2.70088E-33 | 1.10977E-28 |
| MS4A6A | -0.795668052 | 1.08397E-35 | 4.45392E-31 |
| GRN | -0.79956088 | 3.78808E-43 | 1.55649E-38 |
| FCGR3B | -0.808581632 | 8.11223E-11 | 3.33323E-06 |
| MNDA | -0.849014175 | 2.10636E-46 | 8.65481E-42 |
| IFI44L | -0.88390836 | 1.14113E-23 | 4.68878E-19 |
| IFI27 | -0.904180238 | 7.85329E-08 | 0.003226837 |
| CD36 | -0.948836196 | 7.57471E-31 | 3.11237E-26 |
| VCAN | -1.042719563 | 1.7111E-53 | 7.03074E-49 |
| CD14 | -1.04384328 | 5.32747E-36 | 2.189E-31 |
| ENSG00000257764 | -1.115913863 | 2.80191E-45 | 1.15128E-40 |
| IFI6 | -1.137795697 | 4.44616E-42 | 1.82688E-37 |
| S100A8 | -1.196239304 | 1.36074E-73 | 5.59113E-69 |
| LGALS2 | -1.274629666 | 9.59172E-46 | 3.94114E-41 |
| LYZ | -1.434002398 | 2.32461E-84 | 9.55157E-80 |
| S100A9 | -1.493967041 | 4.00804E-84 | 1.64686E-79 |
| S100A12 | -1.507907244 | 2.91678E-48 | 1.19848E-43 |
