# Supplementary material for: Decoding the immune landscape following hip fracture in elderly patients: unveiling temporal dynamics through single-cell RNA sequencing
Source: Immun Ageing. 2023 Oct 17;20:54. doi: 10.1186/s12979-023-00380-6 (PMC10580557; doi:10.1186/s12979-023-00380-6)
Supplement: Supplementary file 4 — Supplementary Material 4 [file 12979_2023_380_MOESM4_ESM.docx]

**Supplementary Table 3.** Top 100 DEGs in Treg cells(24h post-surgery vs. 24h post-trauma)

| **GeneName** | **log2FC** | **Pvlaue** | **Qvalue** |
| --- | --- | --- | --- |
| HBB | 3.003164689 | 2.57555E-42 | 1.05827E-37 |
| HBA2 | 1.414476471 | 6.04359E-15 | 2.48325E-10 |
| HBA1 | 1.273871965 | 1.2789E-12 | 5.25485E-08 |
| IGKC | 0.956416793 | 5.36329E-22 | 2.20372E-17 |
| ENSG00000289474 | 0.785403462 | 2.95962E-10 | 1.21608E-05 |
| MTATP6P1 | 0.641161734 | 5.37687E-14 | 2.2093E-09 |
| MT-RNR2 | 0.58182796 | 6.52388E-11 | 2.6806E-06 |
| MT-CO3 | 0.551114018 | 9.06595E-27 | 3.72511E-22 |
| RPL10P9 | 0.484176919 | 3.5125E-05 | 1 |
| ACTG1 | 0.453434324 | 0.000106299 | 1 |
| IGLC2 | 0.428346807 | 6.21453E-06 | 0.255348993 |
| ENSG00000268027 | 0.422292603 | 1.52635E-05 | 0.627162839 |
| MT-CO1 | 0.416742371 | 3.22916E-13 | 1.32683E-08 |
| MT-RNR1 | 0.378478693 | 3.72178E-05 | 1 |
| MT-CYB | 0.344923359 | 4.15941E-08 | 0.001709059 |
| OSTF1 | 0.342491585 | 0.000315446 | 1 |
| SOCS3 | 0.325269465 | 0.010995995 | 1 |
| SLC25A3 | 0.322020967 | 0.000519404 | 1 |
| GAPDH | 0.308694899 | 0.000923939 | 1 |
| IGHA2 | 0.298482514 | 3.02494E-07 | 0.012429165 |
| ITM2A | 0.291011301 | 0.015953087 | 1 |
| CD69 | 0.290300281 | 0.006856892 | 1 |
| RPS5 | 0.288806402 | 4.61811E-07 | 0.018975348 |
| RPS4X | 0.288570675 | 1.48666E-12 | 6.10854E-08 |
| EEF1B2 | 0.285193371 | 4.69201E-06 | 0.1927899 |
| C4orf48 | 0.282187496 | 0.026188953 | 1 |
| LGALS1 | 0.277107553 | 0.04075592 | 1 |
| MZT2B | 0.270807765 | 0.000294973 | 1 |
| SPAG7 | 0.254571097 | 0.003550449 | 1 |
| DCTN3 | 0.253073516 | 0.002418141 | 1 |
| HMGN2 | 0.251650066 | 0.000421348 | 1 |
| RPL12 | 0.245721719 | 0.000167888 | 1 |
| TRADD | 0.244055037 | 0.011651105 | 1 |
| NACA | 0.24380392 | 1.14299E-05 | 0.469642289 |
| RPLP1 | 0.241613662 | 1.0769E-08 | 0.000442489 |
| UQCRH | 0.241298856 | 0.005727746 | 1 |
| MT1E | 0.238627118 | 0.002903046 | 1 |
| NDUFB5 | 0.238104875 | 0.006297186 | 1 |
| PA2G4 | 0.235306114 | 0.030015497 | 1 |
| RPS12 | 0.233070754 | 0.000859731 | 1 |
| RPL39 | 0.230363743 | 1.06878E-05 | 0.439149394 |
| COMMD6 | 0.228927088 | 0.006355748 | 1 |
| DEFA3 | 0.226359632 | 1.11111E-06 | 0.045654341 |
| RPL10A | 0.225724194 | 0.000132319 | 1 |
| COMTD1 | 0.223609291 | 0.024255396 | 1 |
| CLTB | 0.221778384 | 0.001340044 | 1 |
| TRAT1 | 0.220233368 | 0.022729889 | 1 |
| CREM | 0.219944509 | 0.00033337 | 1 |
| GNG5 | 0.219918522 | 0.000112013 | 1 |
| ANAPC15 | 0.215314049 | 0.002035045 | 1 |
| AP3M2 | 0.214476998 | 0.048155095 | 1 |
| RGL4 | 0.213878414 | 0.094900711 | 1 |
| DAD1 | 0.212105543 | 0.016804179 | 1 |
| RPL6 | 0.211758945 | 2.10219E-06 | 0.086376897 |
| CCDC167 | 0.210627615 | 0.008155215 | 1 |
| KLHL6 | 0.208554088 | 0.004749999 | 1 |
| SNHG29 | 0.207925842 | 0.070492428 | 1 |
| RPLP0 | 0.206795442 | 0.00373069 | 1 |
| GBP2 | 0.206380021 | 0.039636099 | 1 |
| RPL34 | 0.205696936 | 0.001429411 | 1 |
| POP5 | 0.205508714 | 9.78696E-05 | 1 |
| RPL3 | 0.205053401 | 8.43729E-05 | 1 |
| MIF | 0.204843358 | 0.000372908 | 1 |
| GART | 0.204842968 | 0.05295041 | 1 |
| SEC11C | 0.203611767 | 0.021556079 | 1 |
| EXOSC7 | 0.202841227 | 0.058608774 | 1 |
| NPM1 | 0.202736056 | 0.000874478 | 1 |
| ENSG00000267737 | 0.201836486 | 0.001165666 | 1 |
| PCLAF | 0.201462775 | 0.011750131 | 1 |
| MT-ATP6 | 0.201310219 | 0.000416772 | 1 |
| CISD2 | 0.200426884 | 0.013252784 | 1 |
| SSR2 | 0.199189465 | 0.064858918 | 1 |
| NKAPD1 | 0.199060106 | 0.015862506 | 1 |
| PTTG1 | 0.198588592 | 0.01365681 | 1 |
| PDSS2 | 0.196412884 | 0.000237248 | 1 |
| MT-CO2 | 0.194856394 | 0.001272599 | 1 |
| CLEC2B | 0.192929726 | 0.094715602 | 1 |
| ROMO1 | 0.192924051 | 0.021826427 | 1 |
| CTLA4 | 0.192849341 | 0.059972985 | 1 |
| RPL18 | 0.192111227 | 0.000207528 | 1 |
| SELENOH | 0.191738196 | 0.114752081 | 1 |
| SNHG5 | 0.190824806 | 0.134826328 | 1 |
| PLGRKT | 0.189540133 | 0.104513226 | 1 |
| SLC25A6 | 0.188590627 | 0.040653095 | 1 |
| SAT1 | 0.187653371 | 0.091739907 | 1 |
| RPL19 | 0.187348274 | 0.000223717 | 1 |
| RPL32 | 0.187232342 | 0.002989116 | 1 |
| FAM177A1 | 0.187058866 | 0.009180402 | 1 |
| ATP5IF1 | 0.186427107 | 0.100083961 | 1 |
| COPS4 | 0.185413801 | 0.002166988 | 1 |
| CARD8 | 0.185120096 | 0.041613768 | 1 |
| JCHAIN | 0.184723652 | 0.000306299 | 1 |
| BBLN | 0.18392374 | 0.126168815 | 1 |
| RBIS | 0.1833935 | 0.105786713 | 1 |
| NSA2 | 0.183291229 | 0.09243695 | 1 |
| TRAPPC3 | 0.1832064 | 0.123786028 | 1 |
| PDE4B | 0.183045814 | 1.68074E-05 | 0.690598125 |
| RPS27A | 0.182787662 | 8.32801E-07 | 0.034218968 |
| RPL29 | 0.181879338 | 0.00036797 | 1 |
| RPS19 | 0.181858846 | 0.001726986 | 1 |
| MBP | -0.282907527 | 0.019003853 | 1 |
| LMBRD1 | -0.28349381 | 0.002349109 | 1 |
| LRRFIP1 | -0.283535317 | 0.000600124 | 1 |
| FOS | -0.284177615 | 0.005145617 | 1 |
| MT-ND2 | -0.285250325 | 0.000119907 | 1 |
| XRRA1 | -0.285473883 | 0.000547887 | 1 |
| NAMPT | -0.287150829 | 0.104092405 | 1 |
| MYH9 | -0.288472343 | 8.73758E-05 | 1 |
| DDX3X | -0.289337995 | 0.014959398 | 1 |
| GPRIN3 | -0.289355721 | 0.019180914 | 1 |
| SMCHD1 | -0.289589472 | 0.002745819 | 1 |
| HLA-DRA | -0.290102211 | 0.080827792 | 1 |
| ANKRD12 | -0.291793386 | 0.001709831 | 1 |
| LRRC58 | -0.292183448 | 0.0020728 | 1 |
| CALR | -0.292637145 | 0.00864853 | 1 |
| TRIM14 | -0.294025844 | 0.003686515 | 1 |
| IRF1 | -0.295713482 | 0.015691867 | 1 |
| UTRN | -0.295741949 | 1.35561E-05 | 0.557007988 |
| RAB29 | -0.297933685 | 0.013835563 | 1 |
| PRKCB | -0.29824924 | 0.00047771 | 1 |
| RBBP4 | -0.299516709 | 0.00083896 | 1 |
| SLFN5 | -0.299663686 | 0.00856862 | 1 |
| MPHOSPH8 | -0.29976276 | 0.000371935 | 1 |
| MAP4K4 | -0.299953514 | 0.008986379 | 1 |
| GLS | -0.300294893 | 0.006113538 | 1 |
| XIAP | -0.30153491 | 0.002632105 | 1 |
| ODF2L | -0.301905684 | 0.001252886 | 1 |
| GLG1 | -0.30276769 | 0.000750351 | 1 |
| SRRM2 | -0.304583121 | 0.000633883 | 1 |
| RAF1 | -0.304726428 | 0.001404703 | 1 |
| AKAP13 | -0.305061653 | 0.003731711 | 1 |
| RSBN1L | -0.305348189 | 3.5906E-06 | 0.147534248 |
| VHL | -0.305415357 | 1.18124E-06 | 0.048535912 |
| RASSF5 | -0.306974822 | 0.005278405 | 1 |
| ZNF91 | -0.307659022 | 3.55366E-05 | 1 |
| WDR82 | -0.309777687 | 4.20421E-05 | 1 |
| MIA3 | -0.311607249 | 0.006337375 | 1 |
| ENSG00000230606 | -0.312057414 | 0.00136102 | 1 |
| ERICH1 | -0.312105366 | 0.007239951 | 1 |
| ARFGAP2 | -0.313175735 | 0.003974754 | 1 |
| ERAP2 | -0.313338226 | 0.005081044 | 1 |
| SEMA4D | -0.31372514 | 0.002532193 | 1 |
| DNMT1 | -0.315181939 | 0.001192299 | 1 |
| DIAPH1 | -0.315666842 | 2.4994E-05 | 1 |
| TSPAN14 | -0.319280225 | 0.002626659 | 1 |
| EIF4A1 | -0.320014845 | 0.005143746 | 1 |
| CCL5 | -0.32145859 | 0.017272286 | 1 |
| KMT2A | -0.321890725 | 0.004025421 | 1 |
| AHNAK | -0.324851892 | 0.002499073 | 1 |
| TMC6 | -0.324985499 | 3.68559E-05 | 1 |
| PDE7A | -0.326440583 | 0.000340762 | 1 |
| TYMP | -0.326854373 | 7.43741E-06 | 0.305595559 |
| PPM1K | -0.329206131 | 0.002066091 | 1 |
| DIP2A | -0.331192441 | 0.002638038 | 1 |
| AKNA | -0.33249805 | 1.82569E-06 | 0.075015927 |
| XAF1 | -0.334243114 | 0.009726347 | 1 |
| SLC39A10 | -0.33521331 | 0.008477542 | 1 |
| MLLT6 | -0.335294947 | 0.000825226 | 1 |
| NFKBIA | -0.336495494 | 0.000372802 | 1 |
| ANKRD13D | -0.336588405 | 0.001349898 | 1 |
| BIRC3 | -0.337423191 | 0.000611113 | 1 |
| RRN3P1 | -0.338676584 | 0.000551738 | 1 |
| TUBA1B | -0.339235591 | 5.2599E-05 | 1 |
| NEAT1 | -0.340243033 | 0.020852268 | 1 |
| UBE2G2 | -0.340759212 | 0.000403314 | 1 |
| KPNA3 | -0.341607812 | 0.000322974 | 1 |
| TRIM56 | -0.341926289 | 0.002882619 | 1 |
| GOLGA4 | -0.343805898 | 0.000852229 | 1 |
| CUTALP | -0.344334545 | 0.001395078 | 1 |
| H4C3 | -0.345323482 | 0.001055407 | 1 |
| RHOG | -0.345693301 | 0.000954191 | 1 |
| CDK13 | -0.346446291 | 0.000739553 | 1 |
| SLAIN2 | -0.347264595 | 0.000456078 | 1 |
| FERMT3 | -0.353333282 | 8.84209E-05 | 1 |
| ZFP36L1 | -0.353721303 | 0.000169038 | 1 |
| MACF1 | -0.355057507 | 0.001746016 | 1 |
| RESF1 | -0.355298807 | 0.000112733 | 1 |
| ADD3 | -0.355451813 | 3.15316E-06 | 0.129560079 |
| PHIP | -0.356694622 | 0.001424833 | 1 |
| ANKRD44 | -0.356794514 | 2.15359E-05 | 0.884889203 |
| NLRC5 | -0.360445249 | 0.001288468 | 1 |
| XIST | -0.360608211 | 3.03736E-05 | 1 |
| DUSP1 | -0.372993162 | 0.001581857 | 1 |
| KMT2E | -0.377409202 | 5.95751E-06 | 0.24478819 |
| FCN1 | -0.378377512 | 1.35575E-05 | 0.557062479 |
| SPN | -0.379338301 | 0.000301779 | 1 |
| SORL1 | -0.381708283 | 0.000106443 | 1 |
| MX1 | -0.391245032 | 0.000873714 | 1 |
| PCSK7 | -0.39152541 | 0.000522035 | 1 |
| DYNC1H1 | -0.393766076 | 3.61292E-06 | 0.148451277 |
| GNLY | -0.399343221 | 0.010843636 | 1 |
| ZBTB7A | -0.400132371 | 0.000265667 | 1 |
| TTC3 | -0.415139304 | 3.42392E-05 | 1 |
| HLA-DRB1 | -0.455119156 | 0.00050179 | 1 |
| LYZ | -0.458496303 | 7.20276E-06 | 0.295954372 |
| HLA-DRB5 | -0.471860219 | 8.72036E-05 | 1 |
| JUN | -0.513869524 | 4.78204E-06 | 0.196489289 |
| S100A8 | -0.549878253 | 3.11257E-07 | 0.012789219 |
| TTN | -0.554305804 | 8.29001E-07 | 0.034062824 |
| S100A9 | -0.774261736 | 1.76068E-15 | 7.23446E-11 |
