# Supplementary material for: Decoding the immune landscape following hip fracture in elderly patients: unveiling temporal dynamics through single-cell RNA sequencing
Source: Immun Ageing. 2023 Oct 17;20:54. doi: 10.1186/s12979-023-00380-6 (PMC10580557; doi:10.1186/s12979-023-00380-6)
Supplement: Supplementary file 5 — Supplementary Material 5 [file 12979_2023_380_MOESM5_ESM.docx]

**Supplementary Table 4.** Top 100 DEGs in Memory B cells ( 24h post-surgery vs. 24h post-trauma)

| **GeneName** | **log2FC** | **Pvlaue** | **Qvalue** |
| --- | --- | --- | --- |
| MTATP6P1 | 0.815577576 | 3.2583E-28 | 1.3388E-23 |
| JUN | 0.755888038 | 2.11012E-10 | 8.67026E-06 |
| MT-RNR2 | 0.671209463 | 3.02342E-21 | 1.24229E-16 |
| IER2 | 0.658140744 | 4.87377E-08 | 0.002002583 |
| CD69 | 0.657470301 | 2.07031E-08 | 0.000850671 |
| DUSP1 | 0.587724431 | 4.36253E-10 | 1.79252E-05 |
| ENSG00000289474 | 0.521485573 | 1.3531E-08 | 0.000555975 |
| DUSP2 | 0.507693767 | 0.002643907 | 1 |
| NFKBIA | 0.500954345 | 1.23445E-08 | 0.000507222 |
| JUNB | 0.492235865 | 8.08641E-06 | 0.33226266 |
| TSC22D3 | 0.414150809 | 1.1803E-06 | 0.048497381 |
| JUND | 0.391148652 | 3.62054E-07 | 0.014876443 |
| CXCR4 | 0.389486233 | 5.00867E-05 | 1 |
| MT-CO3 | 0.358740285 | 1.70248E-20 | 6.99531E-16 |
| MT-RNR1 | 0.354435995 | 4.15306E-07 | 0.017064499 |
| ZFP36 | 0.350889339 | 0.000458582 | 1 |
| H1-3 | 0.327684474 | 0.000265441 | 1 |
| MZT2A | 0.324457469 | 6.5016E-05 | 1 |
| ENSG00000287979 | 0.319220823 | 0.001821645 | 1 |
| H3-3B | 0.297742492 | 1.64417E-05 | 0.675573093 |
| H1-10 | 0.294523959 | 0.030092703 | 1 |
| PPP1R15A | 0.285317481 | 0.001499373 | 1 |
| KLF6 | 0.284667645 | 0.009705532 | 1 |
| CD27 | 0.279514064 | 0.00621861 | 1 |
| ENSG00000288943 | 0.276988744 | 0.00026966 | 1 |
| GPR183 | 0.276739028 | 0.000457495 | 1 |
| JCHAIN | 0.274215402 | 0.020374966 | 1 |
| IGHA2 | 0.25783708 | 0.007517496 | 1 |
| MT-ATP8 | 0.25348637 | 0.002745268 | 1 |
| RPL41P2 | 0.251524818 | 0.006267 | 1 |
| ZCRB1 | 0.249604445 | 0.007222675 | 1 |
| MT-CO1 | 0.246717596 | 4.38087E-15 | 1.80006E-10 |
| PMAIP1 | 0.24501452 | 0.034169586 | 1 |
| NDUFA3 | 0.242479857 | 0.007369344 | 1 |
| SF3B5 | 0.240958726 | 0.010191539 | 1 |
| HMGB2 | 0.235111789 | 0.010673801 | 1 |
| CRIP1 | 0.233618464 | 0.018168687 | 1 |
| LSM2 | 0.233447525 | 0.001443621 | 1 |
| EEF1B2 | 0.230063978 | 7.68503E-07 | 0.031577038 |
| S1PR4 | 0.228666944 | 0.002104488 | 1 |
| ZC2HC1A | 0.227617894 | 0.011219892 | 1 |
| PPIB | 0.220026639 | 0.007548312 | 1 |
| NOP10 | 0.215240861 | 0.019036044 | 1 |
| ENSG00000237550 | 0.214524607 | 0.001476186 | 1 |
| ODC1 | 0.214075727 | 0.001757776 | 1 |
| RUVBL2 | 0.213188475 | 8.71871E-06 | 0.358243111 |
| MT-CO2 | 0.212520453 | 1.47109E-07 | 0.00604455 |
| MT-CYB | 0.209590581 | 5.08454E-07 | 0.020891863 |
| SNU13 | 0.205613149 | 0.043828654 | 1 |
| RPL6 | 0.204383123 | 2.71404E-06 | 0.111517052 |
| EAPP | 0.203866588 | 0.073684261 | 1 |
| FIS1 | 0.202970447 | 0.04452007 | 1 |
| YIPF5 | 0.202955401 | 0.004520975 | 1 |
| USP3 | 0.202688501 | 0.068428042 | 1 |
| PHB2 | 0.202079076 | 0.002705094 | 1 |
| NME4 | 0.198393092 | 0.016451546 | 1 |
| RBIS | 0.197688813 | 0.008970614 | 1 |
| JPT1 | 0.196622194 | 0.042425818 | 1 |
| PPP3CC | 0.196453182 | 0.00485542 | 1 |
| GTF3A | 0.187663864 | 0.04572683 | 1 |
| PSENEN | 0.187440971 | 0.0212939 | 1 |
| DDAH2 | 0.186989752 | 0.018863032 | 1 |
| RPS27 | 0.186127226 | 2.40001E-09 | 9.86139E-05 |
| CD52 | 0.185477527 | 0.000147563 | 1 |
| PFDN1 | 0.183272008 | 0.005646441 | 1 |
| HMGN1 | 0.181649638 | 0.047131137 | 1 |
| RPS12 | 0.180680652 | 5.37516E-05 | 1 |
| RPS4X | 0.179742179 | 4.357E-06 | 0.179024686 |
| ENSG00000223583 | 0.179653567 | 0.024895152 | 1 |
| COX14 | 0.179284361 | 0.085503632 | 1 |
| RWDD1 | 0.175164132 | 0.027245154 | 1 |
| IER5 | 0.174437616 | 0.132075956 | 1 |
| CARD16 | 0.174343088 | 0.084577882 | 1 |
| RPL10A | 0.173912147 | 0.000419801 | 1 |
| ATXN7L3B | 0.173810751 | 0.101612706 | 1 |
| FKBP8 | 0.173723677 | 0.101465481 | 1 |
| PAICS | 0.173494101 | 0.035071153 | 1 |
| COX6B1 | 0.173296912 | 0.063068668 | 1 |
| ARID5B | 0.172682434 | 0.177135636 | 1 |
| MT-ND4 | 0.172510254 | 0.003212418 | 1 |
| MRPL23 | 0.172502571 | 0.067963646 | 1 |
| TAGLN2 | 0.1721086 | 0.246625767 | 1 |
| VAMP8 | 0.17129615 | 0.002548133 | 1 |
| MRPL36 | 0.170443384 | 0.018152118 | 1 |
| BTG1 | 0.170332072 | 0.031602815 | 1 |
| TEX264 | 0.169485148 | 0.157266798 | 1 |
| HNRNPA0 | 0.169282171 | 0.13473666 | 1 |
| RPS5 | 0.169258651 | 0.000329416 | 1 |
| DDIT4 | 0.168727157 | 0.070491422 | 1 |
| KDELR1 | 0.167489298 | 0.07244803 | 1 |
| HERPUD1 | 0.166845521 | 0.123089081 | 1 |
| HSP90B1 | 0.165729898 | 0.009471332 | 1 |
| EIF1 | 0.16194495 | 0.00157477 | 1 |
| S100A10 | 0.159787936 | 0.159108104 | 1 |
| IQGAP1 | 0.159255515 | 0.013337411 | 1 |
| PTPN22 | 0.157945075 | 0.125471565 | 1 |
| CHMP5 | 0.157665075 | 0.10714769 | 1 |
| CCDC115 | 0.156446259 | 0.069645469 | 1 |
| RNF181 | 0.156372902 | 0.007777598 | 1 |
| CYB5A | 0.156097416 | 0.133629733 | 1 |
| EPS15 | -0.272832305 | 0.000788559 | 1 |
| NIN | -0.272917385 | 0.000567747 | 1 |
| IKZF1 | -0.272990842 | 0.000128198 | 1 |
| RBM41 | -0.273944795 | 0.000784804 | 1 |
| FNBP1 | -0.274439444 | 0.000726333 | 1 |
| FNBP4 | -0.274874838 | 0.002786489 | 1 |
| SLC35E2B | -0.27512091 | 0.000587454 | 1 |
| NIBAN3 | -0.275269464 | 0.000759522 | 1 |
| PRPF40A | -0.27684628 | 0.001784272 | 1 |
| HECTD1 | -0.276881582 | 0.000642598 | 1 |
| CELF1 | -0.27731246 | 0.000356417 | 1 |
| RBM23 | -0.277497519 | 4.42994E-06 | 0.182021628 |
| FIG4 | -0.277813298 | 0.00034901 | 1 |
| ARHGAP30 | -0.278139659 | 6.41056E-06 | 0.26340339 |
| ARF6 | -0.278498796 | 5.79457E-05 | 1 |
| KMT2C | -0.279140601 | 0.001396006 | 1 |
| BIRC6 | -0.279388824 | 0.000564739 | 1 |
| TUG1 | -0.279951805 | 0.001156728 | 1 |
| MDM4 | -0.280110413 | 0.000704741 | 1 |
| FOXP1 | -0.280861176 | 0.026469076 | 1 |
| HVCN1 | -0.280918875 | 0.002134023 | 1 |
| ADAM28 | -0.281049271 | 0.008152348 | 1 |
| PUM1 | -0.282170919 | 2.10276E-05 | 0.864001106 |
| HLA-DRB6 | -0.283517228 | 0.054436451 | 1 |
| SFPQ | -0.284369935 | 0.000651023 | 1 |
| KLHL5 | -0.284830376 | 0.003155607 | 1 |
| MUC20-OT1 | -0.285033752 | 0.000281154 | 1 |
| ITGB1 | -0.286195758 | 0.16040224 | 1 |
| ATF7IP | -0.286453466 | 0.000747488 | 1 |
| TLN1 | -0.287360691 | 0.000985123 | 1 |
| PRDM2 | -0.287786647 | 1.4812E-05 | 0.608610235 |
| AMFR | -0.288414451 | 5.92944E-05 | 1 |
| WAC | -0.288794191 | 0.000593359 | 1 |
| GLIPR1 | -0.289072699 | 0.000193198 | 1 |
| CDK12 | -0.292689691 | 0.000600775 | 1 |
| SMG1 | -0.293028116 | 0.000998879 | 1 |
| SRSF10 | -0.293072124 | 0.00035511 | 1 |
| BOD1L1 | -0.296713272 | 9.64774E-05 | 1 |
| SELL | -0.29690943 | 0.000840895 | 1 |
| KMT2E | -0.298256688 | 0.00015828 | 1 |
| RBM33 | -0.301099779 | 3.64548E-05 | 1 |
| PLCG2 | -0.301552335 | 0.000549873 | 1 |
| LUC7L3 | -0.301690118 | 0.00028057 | 1 |
| TARDBP | -0.301991511 | 0.000837787 | 1 |
| TRIM38 | -0.302604773 | 0.000401696 | 1 |
| DGKD | -0.303728296 | 4.31007E-05 | 1 |
| GLS | -0.304281927 | 0.001053219 | 1 |
| ZNF207 | -0.304694913 | 0.000694841 | 1 |
| STAG2 | -0.305310922 | 0.000291415 | 1 |
| POU2F2 | -0.305762552 | 0.000418915 | 1 |
| S100A4 | -0.30612014 | 0.000826923 | 1 |
| LYZ | -0.307481902 | 1.27517E-05 | 0.523953037 |
| PTPRC | -0.311900666 | 0.000114297 | 1 |
| P2RY10 | -0.312273279 | 1.01312E-05 | 0.416279715 |
| YIPF4 | -0.313749275 | 0.00041156 | 1 |
| MYCBP2 | -0.314806374 | 5.90155E-05 | 1 |
| TCL1A | -0.317251846 | 0.00552436 | 1 |
| DDX17 | -0.317332341 | 2.20677E-05 | 0.906740625 |
| ITSN2 | -0.317722745 | 0.000364637 | 1 |
| SETX | -0.318355417 | 2.82936E-05 | 1 |
| PIAS2 | -0.318462083 | 3.64159E-07 | 0.01496293 |
| LINC02397 | -0.322305373 | 0.002167191 | 1 |
| DDX3X | -0.324644942 | 0.000583152 | 1 |
| POLR2J3.1 | -0.327100469 | 3.99374E-05 | 1 |
| PCSK7 | -0.329079118 | 7.814E-05 | 1 |
| BCL11A | -0.329369485 | 0.000247757 | 1 |
| ANKRD12 | -0.332651241 | 2.91197E-05 | 1 |
| LYST | -0.334081801 | 0.000431659 | 1 |
| S100A8 | -0.334469264 | 0.000463308 | 1 |
| CIITA | -0.335799859 | 0.000139539 | 1 |
| RNF213 | -0.340758758 | 8.14657E-05 | 1 |
| IQSEC1 | -0.342593036 | 1.62915E-06 | 0.066940201 |
| EBF1 | -0.345443123 | 2.24479E-05 | 0.922360183 |
| ITPR1 | -0.352245916 | 0.000288775 | 1 |
| ACTR2 | -0.354544787 | 1.51675E-05 | 0.623219109 |
| TTN | -0.35601542 | 0.00149683 | 1 |
| CNTRL | -0.356528613 | 1.08738E-05 | 0.446795356 |
| NOTCH2NLC | -0.358801018 | 2.41331E-06 | 0.099160699 |
| ANKRD44 | -0.370712942 | 5.12477E-06 | 0.210571614 |
| USP8 | -0.372011121 | 7.9222E-05 | 1 |
| GOLGA4 | -0.37338984 | 7.9954E-07 | 0.032852293 |
| MAP4K4 | -0.378122137 | 9.62777E-08 | 0.003955952 |
| BCLAF1 | -0.378141825 | 1.66573E-06 | 0.068443174 |
| FTX | -0.382089529 | 3.84619E-05 | 1 |
| STX7 | -0.384112683 | 2.99266E-05 | 1 |
| MSN | -0.390623798 | 6.55992E-06 | 0.26954036 |
| KCNQ1OT1 | -0.400347148 | 1.59189E-05 | 0.654091547 |
| ATM | -0.419926733 | 4.23058E-07 | 0.01738304 |
| BPTF | -0.425777699 | 1.21012E-07 | 0.004972251 |
| DAPP1 | -0.425872338 | 4.28872E-09 | 0.000176219 |
| CREBRF | -0.427665171 | 1.41072E-07 | 0.005796495 |
| CELF2 | -0.433851807 | 1.89682E-07 | 0.007793832 |
| NEAT1 | -0.479628742 | 1.08779E-06 | 0.044696007 |
| SMCHD1 | -0.490160655 | 9.53273E-09 | 0.00039169 |
| XIST | -0.500612225 | 4.80057E-08 | 0.001972505 |
| S100A9 | -0.502093258 | 6.1638E-11 | 2.53264E-06 |
| RPS4Y1 | -0.526824453 | 0.003247007 | 1 |
| HBA1 | -1.04290605 | 0.000176773 | 1 |
| HBA2 | -1.511680318 | 1.96441E-08 | 0.000807158 |
| HBB | -3.100006728 | 8.32183E-17 | 3.41936E-12 |
