# Supplementary material for: Decoding the immune landscape following hip fracture in elderly patients: unveiling temporal dynamics through single-cell RNA sequencing
Source: Immun Ageing. 2023 Oct 17;20:54. doi: 10.1186/s12979-023-00380-6 (PMC10580557; doi:10.1186/s12979-023-00380-6)
Supplement: Supplementary file 6 — Supplementary Material 6 [file 12979_2023_380_MOESM6_ESM.docx]

**Supplementary Table 5.** Top 100 DEGs in Naive B cells(24h post-surgery vs. 24h post-trauma)

| **GeneName** | **log2FC** | **Pvlaue** | **Qvalue** |
| --- | --- | --- | --- |
| CD69 | 0.84287019 | 4.8274E-36 | 1.98353E-31 |
| H1-10 | 0.737852752 | 3.7623E-22 | 1.54589E-17 |
| MTATP6P1 | 0.733750594 | 5.91219E-61 | 2.42926E-56 |
| MT-RNR2 | 0.648059044 | 3.55712E-71 | 1.46158E-66 |
| ENSG00000289474 | 0.647905559 | 2.31042E-25 | 9.49329E-21 |
| CXCR4 | 0.621598882 | 5.48036E-35 | 2.25183E-30 |
| JUN | 0.583984013 | 2.24806E-18 | 9.23707E-14 |
| DUSP1 | 0.566274405 | 2.99009E-20 | 1.2286E-15 |
| JUNB | 0.494982213 | 1.73849E-14 | 7.14326E-10 |
| IGHA2 | 0.444899557 | 7.20775E-17 | 2.96159E-12 |
| H3-3B | 0.418266308 | 3.12717E-18 | 1.28492E-13 |
| H1-2 | 0.409920735 | 7.56135E-11 | 3.10688E-06 |
| RPL41P2 | 0.393637719 | 1.82869E-13 | 7.51392E-09 |
| H1-3 | 0.386209446 | 7.80522E-10 | 3.20709E-05 |
| H1-4 | 0.376916329 | 2.82586E-11 | 1.16112E-06 |
| MT-CO3 | 0.323379183 | 1.35713E-52 | 5.57632E-48 |
| ENSG00000237550 | 0.311737087 | 5.54519E-12 | 2.27846E-07 |
| RPS12 | 0.28754066 | 5.01482E-36 | 2.06054E-31 |
| RPS27 | 0.287393074 | 3.84093E-58 | 1.5782E-53 |
| MT-RNR1 | 0.284778613 | 6.60999E-19 | 2.71598E-14 |
| JUND | 0.284498198 | 2.1957E-08 | 0.000902189 |
| IER2 | 0.271280795 | 4.40847E-06 | 0.181139542 |
| RPL10A | 0.266473796 | 7.73987E-21 | 3.18024E-16 |
| S100A8 | 0.260656974 | 2.09726E-06 | 0.086174184 |
| SOCS1 | 0.259980935 | 1.38407E-10 | 5.68701E-06 |
| RPS5 | 0.257447345 | 8.61487E-22 | 3.53976E-17 |
| RPS19 | 0.251698934 | 1.16273E-24 | 4.77753E-20 |
| NFKBIA | 0.249782048 | 1.96833E-05 | 0.808767073 |
| RPS4X | 0.247777288 | 3.97889E-27 | 1.63489E-22 |
| EEF1B2 | 0.238249647 | 1.11951E-11 | 4.59997E-07 |
| BTG1 | 0.238099131 | 1.33055E-12 | 5.46708E-08 |
| RPL6 | 0.235240157 | 2.46761E-16 | 1.01392E-11 |
| TCL1A | 0.230501135 | 4.11443E-05 | 1 |
| RPL32 | 0.228418684 | 3.94139E-36 | 1.61948E-31 |
| RPL3 | 0.227929525 | 1.43764E-27 | 5.9071E-23 |
| RPL35 | 0.227599038 | 2.35935E-13 | 9.69432E-09 |
| ODC1 | 0.22734815 | 3.9473E-05 | 1 |
| RPL35A | 0.226312561 | 1.15762E-22 | 4.75655E-18 |
| TSC22D3 | 0.225934578 | 0.000192828 | 1 |
| SNRPD2 | 0.225543426 | 4.18087E-07 | 0.017178768 |
| RPL13 | 0.225415463 | 3.26318E-41 | 1.34081E-36 |
| RPL29 | 0.224128175 | 6.41723E-23 | 2.63677E-18 |
| NACA | 0.22340536 | 1.64387E-16 | 6.7545E-12 |
| HMGN2 | 0.221491957 | 6.39769E-06 | 0.262874653 |
| UBC | 0.216892201 | 3.36036E-06 | 0.138073998 |
| C7orf50 | 0.215154713 | 0.000187106 | 1 |
| MZT2A | 0.210642245 | 2.39127E-06 | 0.098254969 |
| RPS8 | 0.205205712 | 2.72576E-24 | 1.11999E-19 |
| RPS6 | 0.204970652 | 1.15695E-20 | 4.75381E-16 |
| JCHAIN | 0.204505735 | 0.001670258 | 1 |
| DDIT4 | 0.200687072 | 0.000230004 | 1 |
| MT-CYB | 0.199501565 | 3.62622E-20 | 1.48998E-15 |
| PPDPF | 0.198734511 | 2.24879E-05 | 0.924003351 |
| PRDX1 | 0.19704903 | 1.10361E-05 | 0.453462177 |
| RPL37 | 0.194227853 | 1.05063E-26 | 4.31693E-22 |
| RPLP1 | 0.192211095 | 1.59849E-16 | 6.56804E-12 |
| RPS13 | 0.187980609 | 1.72999E-22 | 7.10836E-18 |
| PPP1R15A | 0.187373542 | 0.000114893 | 1 |
| TMEM258 | 0.18619906 | 0.000329515 | 1 |
| RPS28 | 0.18591338 | 4.30381E-23 | 1.76839E-18 |
| RPL8 | 0.183604612 | 5.26453E-15 | 2.16314E-10 |
| COMMD6 | 0.181346808 | 5.5146E-05 | 1 |
| EIF1 | 0.178327434 | 2.82071E-06 | 0.115900171 |
| RPS18 | 0.1782542 | 5.09323E-20 | 2.09276E-15 |
| CD52 | 0.176179338 | 6.99914E-07 | 0.028758751 |
| HERPUD1 | 0.175018176 | 0.003701339 | 1 |
| SNHG8 | 0.17394059 | 5.54875E-06 | 0.227992514 |
| IGHG2 | 0.172918109 | 3.7637E-05 | 1 |
| RPS21 | 0.172077403 | 5.14174E-16 | 2.11269E-11 |
| ARRDC3 | 0.171848148 | 6.35143E-05 | 1 |
| RPL19 | 0.17160241 | 2.67564E-19 | 1.09939E-14 |
| RPL10 | 0.17041616 | 2.26036E-16 | 9.28761E-12 |
| RPL18 | 0.170001775 | 8.26086E-18 | 3.3943E-13 |
| C12orf57 | 0.169800684 | 0.006214808 | 1 |
| H2AJ | 0.168623166 | 1.84751E-05 | 0.759124584 |
| RPS27A | 0.166718296 | 3.76223E-22 | 1.54586E-17 |
| WASHC3 | 0.165900515 | 7.5045E-05 | 1 |
| RPS3 | 0.164916683 | 2.97279E-16 | 1.22149E-11 |
| CYB5A | 0.162182243 | 3.49937E-05 | 1 |
| ENSG00000288943 | 0.161441014 | 0.003971976 | 1 |
| NDUFA1 | 0.161253244 | 3.48588E-05 | 1 |
| RPL12 | 0.160835431 | 1.37798E-07 | 0.005661989 |
| SAP18 | 0.159860845 | 0.000978393 | 1 |
| ENSG00000272256 | 0.158782416 | 0.000141978 | 1 |
| RPS25 | 0.158081413 | 1.63723E-08 | 0.000672723 |
| ARHGDIB | 0.157119967 | 1.50727E-05 | 0.619322248 |
| IGKC | 0.156898276 | 6.11255E-11 | 2.51159E-06 |
| ZNHIT3 | 0.156555415 | 2.70501E-05 | 1 |
| RPL11 | 0.15616553 | 1.68795E-14 | 6.93563E-10 |
| RBIS | 0.154942604 | 5.45389E-06 | 0.224094741 |
| RPL22 | 0.153214791 | 0.00014277 | 1 |
| VPREB3 | 0.152931124 | 0.043670252 | 1 |
| CYCS | 0.152160792 | 0.000219914 | 1 |
| SNRPB2 | 0.15186278 | 0.001630833 | 1 |
| RPS7 | 0.151318956 | 2.72024E-11 | 1.11772E-06 |
| RPL34 | 0.14916827 | 3.8589E-14 | 1.58558E-09 |
| RPS24 | 0.148116623 | 4.41953E-09 | 0.000181594 |
| EDF1 | 0.146970129 | 0.00183801 | 1 |
| MCRIP1 | 0.146806851 | 0.000109272 | 1 |
| RPS23 | 0.146133985 | 2.09666E-15 | 8.61497E-11 |
| NIPBL | -0.298676775 | 4.40579E-09 | 0.00018103 |
| SMG1 | -0.298866004 | 6.40486E-09 | 0.000263169 |
| TMEM165 | -0.299151987 | 1.63421E-10 | 6.71479E-06 |
| IKZF3 | -0.299789786 | 5.30734E-09 | 0.000218073 |
| SP100 | -0.299986398 | 6.48782E-08 | 0.00266578 |
| ASH1L | -0.300524532 | 1.15742E-10 | 4.7557E-06 |
| SEL1L3 | -0.301122708 | 1.38433E-07 | 0.005688083 |
| NUDT3 | -0.302093276 | 1.00207E-10 | 4.11742E-06 |
| ZBTB20 | -0.302128914 | 3.30639E-08 | 0.001358561 |
| LRRFIP1 | -0.304956194 | 4.36538E-08 | 0.001793691 |
| KANSL1 | -0.305798263 | 3.90122E-10 | 1.60297E-05 |
| BRD4 | -0.306233048 | 1.72201E-10 | 7.07558E-06 |
| CREBZF | -0.30755373 | 8.79703E-10 | 3.61461E-05 |
| MALT1 | -0.307596012 | 3.72594E-08 | 0.00153095 |
| DOCK8 | -0.307676435 | 1.48137E-08 | 0.00060868 |
| DAPP1 | -0.309187063 | 5.4679E-13 | 2.2467E-08 |
| AKNA | -0.309351901 | 1.04493E-08 | 0.000429352 |
| MDM4 | -0.309507856 | 4.54808E-11 | 1.86876E-06 |
| ZEB2 | -0.309608498 | 5.78879E-09 | 0.000237855 |
| PAX5 | -0.310135583 | 6.2971E-09 | 0.000258741 |
| EIF4G2 | -0.310468474 | 2.12056E-09 | 8.71318E-05 |
| PNN | -0.313561579 | 1.02048E-08 | 0.000419306 |
| NRGN | -0.313787235 | 0.001324008 | 1 |
| CREBRF | -0.314877812 | 2.52033E-09 | 0.000103558 |
| HNRNPH1 | -0.315329898 | 1.96623E-09 | 8.07903E-05 |
| PHF3 | -0.317020315 | 1.0613E-07 | 0.004360773 |
| PCBP2 | -0.317174971 | 1.90334E-11 | 7.82063E-07 |
| MARCHF1 | -0.317811268 | 4.92792E-11 | 2.02483E-06 |
| MCL1 | -0.318272467 | 5.34566E-11 | 2.19648E-06 |
| RNF213 | -0.32009338 | 1.51152E-08 | 0.00062107 |
| ADD3 | -0.320245535 | 3.18232E-09 | 0.000130758 |
| KMT2C | -0.320913248 | 5.23783E-10 | 2.15217E-05 |
| SREK1 | -0.322073527 | 4.40735E-10 | 1.81094E-05 |
| BRD2 | -0.323261263 | 5.88184E-10 | 2.41679E-05 |
| NAIP | -0.324775165 | 5.19547E-11 | 2.13477E-06 |
| INPP5D | -0.327048629 | 9.06622E-11 | 3.72522E-06 |
| RERE | -0.329788135 | 4.41488E-10 | 1.81403E-05 |
| ZBTB44 | -0.330185215 | 6.32345E-09 | 0.000259824 |
| MED13L | -0.332195384 | 1.97712E-13 | 8.12377E-09 |
| S100A11 | -0.33279505 | 7.21465E-09 | 0.000296443 |
| TCTN1 | -0.333900079 | 1.22008E-09 | 5.0132E-05 |
| SMCHD1 | -0.333957199 | 7.68882E-10 | 3.15926E-05 |
| FOXP1 | -0.336294594 | 1.13894E-10 | 4.6798E-06 |
| IKZF1 | -0.336371915 | 1.46089E-10 | 6.00266E-06 |
| AKAP13 | -0.337424531 | 3.25229E-14 | 1.33633E-09 |
| ITSN2 | -0.337667668 | 2.24322E-11 | 9.21716E-07 |
| EP300 | -0.340539072 | 3.57969E-12 | 1.47086E-07 |
| SNX29 | -0.340628163 | 1.15875E-10 | 4.76118E-06 |
| TRIM56 | -0.34216548 | 1.55708E-09 | 6.3979E-05 |
| IL4R | -0.342538511 | 2.40054E-08 | 0.000986358 |
| FCRL2 | -0.34391618 | 2.74334E-10 | 1.12721E-05 |
| TNRC6B | -0.344420169 | 2.86643E-12 | 1.17779E-07 |
| ST6GAL1 | -0.345093001 | 8.19328E-12 | 3.36654E-07 |
| RIPOR2 | -0.347196535 | 7.22981E-12 | 2.97066E-07 |
| HNRNPU | -0.35039651 | 3.66721E-12 | 1.50682E-07 |
| TCF4 | -0.351712781 | 5.06715E-09 | 0.000208204 |
| XIST | -0.353806969 | 2.88393E-09 | 0.000118498 |
| PARP14 | -0.355195441 | 3.9495E-11 | 1.62281E-06 |
| USP8 | -0.355424925 | 1.76152E-09 | 7.2379E-05 |
| SNHG14 | -0.35573628 | 2.55742E-08 | 0.001050819 |
| CELF1 | -0.358305155 | 1.04481E-11 | 4.29302E-07 |
| CLEC2D | -0.362165684 | 2.86187E-10 | 1.17591E-05 |
| ZNF207 | -0.365631356 | 2.95241E-12 | 1.21311E-07 |
| CIITA | -0.368381508 | 4.05199E-13 | 1.66492E-08 |
| PPBP | -0.369189136 | 0.000508431 | 1 |
| ATM | -0.372423549 | 1.23424E-13 | 5.07136E-09 |
| GOLGA4 | -0.372585888 | 1.33572E-10 | 5.48836E-06 |
| DDX17 | -0.37280613 | 4.05398E-15 | 1.66574E-10 |
| PNISR | -0.373167719 | 3.82657E-17 | 1.5723E-12 |
| ORAI2 | -0.375886369 | 1.39614E-13 | 5.73662E-09 |
| ATRX | -0.376064869 | 1.64136E-11 | 6.74418E-07 |
| BPTF | -0.385288589 | 1.04118E-12 | 4.27809E-08 |
| PDE7A | -0.387943111 | 3.81419E-15 | 1.56721E-10 |
| FTX | -0.403245284 | 1.38223E-12 | 5.67945E-08 |
| BCL11A | -0.407605514 | 5.92203E-14 | 2.4333E-09 |
| ANKRD44 | -0.415005981 | 2.46503E-15 | 1.01286E-10 |
| LINC02397 | -0.415461031 | 2.42768E-12 | 9.97509E-08 |
| STX7 | -0.416485861 | 9.35298E-16 | 3.84305E-11 |
| PSMA3-AS1 | -0.420155699 | 1.98598E-14 | 8.16018E-10 |
| KMT2A | -0.423330359 | 4.3316E-17 | 1.77981E-12 |
| MACF1 | -0.427306818 | 2.12992E-14 | 8.75164E-10 |
| PCSK7 | -0.448276993 | 2.81248E-12 | 1.15562E-07 |
| ITPR1 | -0.460799488 | 2.24254E-17 | 9.21437E-13 |
| ARID1B | -0.466642863 | 5.14962E-22 | 2.11593E-17 |
| LUC7L3 | -0.470841676 | 7.59774E-19 | 3.12183E-14 |
| LYZ | -0.472387893 | 2.3563E-19 | 9.6818E-15 |
| S100A4 | -0.476083287 | 2.02845E-16 | 8.33471E-12 |
| HLA-DRB6 | -0.476111369 | 1.26942E-08 | 0.000521592 |
| ANKRD11 | -0.485505856 | 2.62102E-17 | 1.07695E-12 |
| RPS4Y1 | -0.491167651 | 1.47963E-13 | 6.07966E-09 |
| POU2F2 | -0.492339204 | 2.63616E-21 | 1.08317E-16 |
| ZNF292 | -0.492551453 | 4.27255E-18 | 1.75555E-13 |
| NEAT1 | -0.502813855 | 2.65758E-13 | 1.09197E-08 |
| KLHL5 | -0.509862411 | 1.68108E-15 | 6.90738E-11 |
| HBA1 | -0.521031477 | 2.53758E-08 | 0.001042667 |
| PRKCB | -0.555714436 | 2.57078E-22 | 1.05631E-17 |
| S100A9 | -0.576870492 | 1.42392E-28 | 5.85074E-24 |
| POLR2J3.1 | -0.656459065 | 8.29643E-33 | 3.40892E-28 |
| HBA2 | -1.047167875 | 9.4922E-24 | 3.90025E-19 |
| HBB | -2.563135808 | 2.38293E-51 | 9.79124E-47 |
