# Supplementary material for: Decoding the immune landscape following hip fracture in elderly patients: unveiling temporal dynamics through single-cell RNA sequencing
Source: Immun Ageing. 2023 Oct 17;20:54. doi: 10.1186/s12979-023-00380-6 (PMC10580557; doi:10.1186/s12979-023-00380-6)
Supplement: Supplementary file 7 — Supplementary Material 7 [file 12979_2023_380_MOESM7_ESM.docx]

**Supplementary Table 6.** Top 100 DEGs in C-Mono2 (7d post-surgery vs. 24h post-surgery)

| **GeneName** | **log2FC** | **Pvlaue** | **Qvalue** |
| --- | --- | --- | --- |
| MT-ND4L | 1.156983511 | 0 | 0 |
| STAT1 | 0.845465369 | 2.9189E-246 | 1.1994E-241 |
| H1-4 | 0.819868874 | 6.894E-217 | 2.8327E-212 |
| IRF1 | 0.794272206 | 6.1506E-192 | 2.5272E-187 |
| VPS13C | 0.749701722 | 2.0469E-246 | 8.4103E-242 |
| LRRK2 | 0.727233807 | 2.3713E-236 | 9.7436E-232 |
| IFI44L | 0.721502678 | 2.067E-160 | 8.4931E-156 |
| MT-ATP8 | 0.720983707 | 1.1227E-192 | 4.613E-188 |
| XAF1 | 0.707568592 | 4.9506E-151 | 2.0341E-146 |
| RNF213 | 0.62755246 | 4.2873E-159 | 1.7616E-154 |
| PPBP | 0.626191739 | 9.11301E-68 | 3.74444E-63 |
| SMCHD1 | 0.619696269 | 9.9681E-165 | 4.0958E-160 |
| OGFRL1 | 0.618671049 | 7.6673E-162 | 3.1504E-157 |
| AHNAK | 0.616102047 | 2.234E-229 | 9.1794E-225 |
| LY6E | 0.607388671 | 1.1247E-115 | 4.6213E-111 |
| HLA-DQB1 | 0.596611237 | 6.4081E-120 | 2.633E-115 |
| LYST | 0.590165765 | 1.2233E-173 | 5.0265E-169 |
| PARP14 | 0.574904068 | 1.8595E-122 | 7.6406E-118 |
| MX1 | 0.560607423 | 8.46063E-91 | 3.47639E-86 |
| DMXL2 | 0.554999774 | 6.362E-132 | 2.6141E-127 |
| HLA-DMB | 0.554388706 | 2.836E-114 | 1.1653E-109 |
| RPS26 | 0.541058217 | 5.2817E-137 | 2.1702E-132 |
| JAK2 | 0.537676803 | 3.1802E-111 | 1.3067E-106 |
| USP15 | 0.527151459 | 1.3994E-137 | 5.7501E-133 |
| MT-RNR2 | 0.525442559 | 0 | 0 |
| SETX | 0.524534027 | 5.089E-113 | 2.091E-108 |
| ITGA4 | 0.519739396 | 3.9704E-103 | 1.63141E-98 |
| ATP2B1 | 0.51030215 | 6.4658E-94 | 2.65673E-89 |
| FGL2 | 0.509365127 | 6.2089E-174 | 2.5512E-169 |
| CD74 | 0.509276334 | 1.4895E-170 | 6.1203E-166 |
| AGTPBP1 | 0.49326588 | 6.95837E-95 | 2.85913E-90 |
| CYBB | 0.491942579 | 1.9705E-222 | 8.0966E-218 |
| NCOA4 | 0.485780714 | 1.7759E-116 | 7.2971E-112 |
| HLA-DPA1 | 0.478743232 | 1.86564E-81 | 7.66574E-77 |
| AKAP9 | 0.47671093 | 1.18335E-83 | 4.86225E-79 |
| MBNL1 | 0.471269276 | 2.8546E-132 | 1.1729E-127 |
| HLA-DRA | 0.462843964 | 2.8769E-126 | 1.1821E-121 |
| NIN | 0.462032297 | 5.88344E-92 | 2.41745E-87 |
| PHF3 | 0.460600777 | 1.14516E-85 | 4.70533E-81 |
| ALDH1A1 | 0.454379622 | 4.83525E-84 | 1.98676E-79 |
| TMEM123 | 0.448688329 | 1.84628E-80 | 7.58617E-76 |
| CTSS | 0.44644519 | 0 | 0 |
| TMEM154 | 0.445737334 | 2.46357E-71 | 1.01225E-66 |
| CELF2 | 0.444471386 | 2.65888E-94 | 1.09251E-89 |
| MYCBP2 | 0.442328983 | 2.93602E-82 | 1.20638E-77 |
| ANKRD44 | 0.442297823 | 9.24869E-79 | 3.80019E-74 |
| ISG15 | 0.441662295 | 2.11452E-75 | 8.68837E-71 |
| ATF7IP | 0.441133782 | 2.47765E-74 | 1.01804E-69 |
| APOL6 | 0.438610841 | 1.06512E-75 | 4.37648E-71 |
| BACH1 | 0.438584076 | 4.6252E-83 | 1.90045E-78 |
| LUC7L3 | 0.437528716 | 1.23537E-81 | 5.07602E-77 |
| EVI2B | 0.435979029 | 3.2047E-136 | 1.3168E-131 |
| ZEB2 | 0.429851497 | 5.6791E-114 | 2.3335E-109 |
| HNRNPU | 0.429472431 | 3.1144E-118 | 1.2797E-113 |
| ATM | 0.426372753 | 9.95211E-68 | 4.08922E-63 |
| UTRN | 0.426001141 | 5.57976E-69 | 2.29267E-64 |
| AP1S2 | 0.42493617 | 4.0633E-108 | 1.6696E-103 |
| RESF1 | 0.421765845 | 1.18347E-62 | 4.86276E-58 |
| HIPK3 | 0.420803338 | 5.12716E-76 | 2.1067E-71 |
| SF3B1 | 0.4203899 | 1.90245E-86 | 7.81698E-82 |
| DOCK8 | 0.418281478 | 2.26543E-93 | 9.30842E-89 |
| PTPRC | 0.417194005 | 3.2303E-157 | 1.3273E-152 |
| TNFSF10 | 0.416413055 | 5.1914E-73 | 2.13309E-68 |
| SAMD9L | 0.413204674 | 4.59771E-68 | 1.88915E-63 |
| MS4A7 | 0.410451074 | 6.11265E-52 | 2.51163E-47 |
| PSME2 | 0.408743609 | 1.55705E-66 | 6.39777E-62 |
| MT-ND5 | 0.407209365 | 4.522E-122 | 1.858E-117 |
| HLA-DPB1 | 0.407186796 | 2.74178E-56 | 1.12657E-51 |
| TET2 | 0.406671496 | 5.39108E-71 | 2.21514E-66 |
| CX3CR1 | 0.406353731 | 2.4362E-56 | 1.00101E-51 |
| MARCKS | 0.404661694 | 1.75439E-49 | 7.20861E-45 |
| KMT2C | 0.403852137 | 7.02914E-76 | 2.88821E-71 |
| MIS18BP1 | 0.398446404 | 3.68571E-65 | 1.51442E-60 |
| CD46 | 0.397700575 | 6.25359E-67 | 2.56954E-62 |
| EIF2AK2 | 0.392573111 | 4.25168E-64 | 1.74697E-59 |
| SCAF11 | 0.390673491 | 1.44438E-82 | 5.93482E-78 |
| DPYD | 0.388724854 | 1.31142E-71 | 5.3885E-67 |
| DDX60L | 0.38659132 | 1.35671E-60 | 5.5746E-56 |
| PICALM | 0.386114641 | 4.77512E-70 | 1.96205E-65 |
| CNTRL | 0.384028335 | 1.51092E-57 | 6.20822E-53 |
| PTBP3 | 0.383598633 | 2.40602E-70 | 9.88608E-66 |
| HLA-DRB1 | 0.383037549 | 8.03183E-65 | 3.3002E-60 |
| EIF3A | 0.378634373 | 2.62491E-77 | 1.07855E-72 |
| CCDC88A | 0.376995871 | 3.36469E-68 | 1.38252E-63 |
| ENSG00000257764 | 0.376802696 | 1.2413E-122 | 5.1003E-118 |
| BIRC6 | 0.376599185 | 6.13062E-60 | 2.51901E-55 |
| IGKC | 0.374933417 | 8.58645E-38 | 3.52809E-33 |
| PRRC2C | 0.374567828 | 1.20309E-67 | 4.94336E-63 |
| ENSG00000255197 | 0.374389449 | 2.34065E-57 | 9.61749E-53 |
| NIPBL | 0.373933855 | 6.134E-61 | 2.5204E-56 |
| ENSG00000237550 | 0.373133131 | 3.45677E-59 | 1.42035E-54 |
| ACAP2 | 0.371650953 | 4.02388E-68 | 1.65337E-63 |
| WARS1 | 0.370909526 | 2.29096E-56 | 9.41331E-52 |
| MEF2C | 0.369889567 | 3.42044E-55 | 1.40542E-50 |
| CLEC12A | 0.367458041 | 8.61042E-90 | 3.53794E-85 |
| MPEG1 | 0.36675624 | 9.79746E-82 | 4.02568E-77 |
| IFI6 | 0.365083326 | 8.77512E-50 | 3.60561E-45 |
| XRN1 | 0.363696785 | 4.5883E-58 | 1.88529E-53 |
| PKN2 | 0.363193232 | 3.74571E-56 | 1.53908E-51 |
| BOD1L1 | 0.36180761 | 4.69228E-55 | 1.92801E-50 |
| MGST3 | -0.291747982 | 1.19262E-36 | 4.90036E-32 |
| RPL27A | -0.292707674 | 2.547E-66 | 1.04653E-61 |
| C5AR1 | -0.292817446 | 4.72733E-30 | 1.94241E-25 |
| IGLC2 | -0.293528801 | 1.99442E-51 | 8.19487E-47 |
| RPL7 | -0.295803238 | 9.58199E-68 | 3.93714E-63 |
| GPX1 | -0.297120824 | 2.30379E-77 | 9.46604E-73 |
| SAMSN1 | -0.297128613 | 1.64658E-32 | 6.76564E-28 |
| MYL12A | -0.297181974 | 8.24685E-58 | 3.38855E-53 |
| RPL36A | -0.301187863 | 7.747E-42 | 3.18317E-37 |
| H3-3A | -0.302099714 | 9.8502E-117 | 4.0474E-112 |
| CSTB | -0.3042521 | 4.59335E-51 | 1.88736E-46 |
| UPP1 | -0.30576554 | 3.82366E-41 | 1.57111E-36 |
| H2AJ | -0.307173833 | 9.35473E-39 | 3.84376E-34 |
| SPI1 | -0.307735206 | 5.70352E-55 | 2.34352E-50 |
| COX6C | -0.309176436 | 1.70632E-48 | 7.01111E-44 |
| PGAM1 | -0.312318938 | 8.45532E-42 | 3.47421E-37 |
| COX8A | -0.316375451 | 3.49797E-56 | 1.43728E-51 |
| MCL1 | -0.316729547 | 4.39587E-60 | 1.80622E-55 |
| MXD1 | -0.317410782 | 8.76835E-37 | 3.60283E-32 |
| SH3BGRL3 | -0.319427988 | 4.5195E-147 | 1.857E-142 |
| GAPDH | -0.325308499 | 1.86061E-82 | 7.64507E-78 |
| CD14 | -0.326435633 | 3.03E-61 | 1.245E-56 |
| DBI | -0.327104006 | 4.95803E-54 | 2.03721E-49 |
| RPS17 | -0.328590184 | 1.12211E-46 | 4.61065E-42 |
| KLF6 | -0.32998382 | 3.00659E-61 | 1.23538E-56 |
| RPL10 | -0.331307341 | 1.8242E-106 | 7.4954E-102 |
| SEC61B | -0.331356018 | 1.76375E-55 | 7.24709E-51 |
| MT-ATP6 | -0.3350483 | 5.49825E-93 | 2.25917E-88 |
| RPL23A | -0.335899398 | 4.20145E-90 | 1.72633E-85 |
| GLUL | -0.337599105 | 3.18077E-45 | 1.30695E-40 |
| ACSL1 | -0.340212842 | 4.79968E-41 | 1.97214E-36 |
| MT-CYB | -0.343482446 | 1.67169E-90 | 6.8688E-86 |
| H3-3B | -0.349478199 | 5.5738E-124 | 2.2902E-119 |
| RPS3A | -0.350923024 | 2.6048E-129 | 1.0703E-124 |
| CTSD | -0.351512998 | 3.08969E-80 | 1.26952E-75 |
| NDUFA13 | -0.352082617 | 6.70813E-52 | 2.7563E-47 |
| ALOX5AP | -0.352805361 | 7.1128E-40 | 2.92258E-35 |
| MYL6 | -0.354002699 | 4.6216E-141 | 1.899E-136 |
| THBS1 | -0.361440585 | 3.02611E-62 | 1.2434E-57 |
| SRGN | -0.36471552 | 6.689E-131 | 2.7485E-126 |
| NFKBIZ | -0.365520676 | 1.68053E-44 | 6.90512E-40 |
| MIR23AHG | -0.367541971 | 5.60001E-45 | 2.30099E-40 |
| RPL26 | -0.369464301 | 8.6607E-139 | 3.5586E-134 |
| NDUFB3 | -0.37368191 | 2.12775E-58 | 8.7427E-54 |
| LDHA | -0.374311649 | 1.08095E-58 | 4.44151E-54 |
| TRIB1 | -0.374330236 | 2.86606E-61 | 1.17764E-56 |
| MAP3K8 | -0.381829165 | 1.15335E-57 | 4.73899E-53 |
| NDUFA4 | -0.383370615 | 1.14033E-69 | 4.6855E-65 |
| PLBD1 | -0.383413456 | 5.60679E-60 | 2.30377E-55 |
| PIM3 | -0.388320773 | 1.88632E-65 | 7.75069E-61 |
| LGALS1 | -0.389936172 | 3.074E-121 | 1.2631E-116 |
| S100A10 | -0.392251649 | 3.2067E-116 | 1.3176E-111 |
| PHC2 | -0.397507323 | 2.83965E-67 | 1.16678E-62 |
| ARPC1B | -0.414537303 | 2.9923E-136 | 1.2295E-131 |
| ADAMTS2 | -0.415239216 | 1.0942E-146 | 4.4962E-142 |
| NOP10 | -0.418954664 | 4.74092E-74 | 1.948E-69 |
| SAP30 | -0.426962533 | 4.30955E-72 | 1.77075E-67 |
| TIMP1 | -0.427631249 | 4.13469E-63 | 1.6989E-58 |
| TXN | -0.428091137 | 1.46456E-81 | 6.01774E-77 |
| LILRA5 | -0.429803962 | 9.43777E-72 | 3.87788E-67 |
| HP | -0.432361882 | 1.28943E-48 | 5.29813E-44 |
| EREG | -0.438008327 | 2.30994E-65 | 9.4913E-61 |
| CLU | -0.44232435 | 2.37305E-68 | 9.75063E-64 |
| IFITM2 | -0.444379937 | 1.12006E-73 | 4.60222E-69 |
| S100A11 | -0.445170157 | 1.0081E-177 | 4.1423E-173 |
| SAT1 | -0.452299856 | 1.5686E-140 | 6.4454E-136 |
| NFIL3 | -0.470251215 | 1.91798E-87 | 7.88079E-83 |
| DDIT4 | -0.474550422 | 1.7639E-155 | 7.2477E-151 |
| IRS2 | -0.474947956 | 2.87395E-80 | 1.18088E-75 |
| RNASE2 | -0.475646766 | 1.26497E-77 | 5.19763E-73 |
| CD63 | -0.479156142 | 5.9337E-111 | 2.4381E-106 |
| PER1 | -0.489465926 | 4.69863E-97 | 1.93062E-92 |
| PIM1 | -0.498254471 | 1.9997E-90 | 8.21655E-86 |
| JUNB | -0.499804828 | 5.8975E-102 | 2.42324E-97 |
| CEBPD | -0.507963526 | 2.7788E-169 | 1.1418E-164 |
| S100A9 | -0.5101474 | 1.4567E-155 | 5.9855E-151 |
| MAFB | -0.512708642 | 5.4095E-80 | 2.22271E-75 |
| SLC2A3 | -0.513765473 | 1.09125E-82 | 4.48385E-78 |
| ADM | -0.534199455 | 1.4372E-114 | 5.9051E-110 |
| FCER1G | -0.53581561 | 7.4053E-167 | 3.0428E-162 |
| G0S2 | -0.536680001 | 2.00037E-65 | 8.21934E-61 |
| CD163 | -0.536850599 | 2.1696E-107 | 8.9145E-103 |
| RPS10 | -0.553309662 | 1.7024E-187 | 6.9949E-183 |
| MCEMP1 | -0.601236264 | 9.2175E-118 | 3.7874E-113 |
| PLAC8 | -0.624561016 | 2.4535E-116 | 1.0081E-111 |
| TSC22D3 | -0.672661648 | 1.3856E-173 | 5.6932E-169 |
| BCL2A1 | -0.672669538 | 8.1626E-143 | 3.3539E-138 |
| IL1R2 | -0.675119741 | 3.0504E-196 | 1.2534E-191 |
| CEBPB | -0.743968318 | 3.1423E-231 | 1.2911E-226 |
| S100A8 | -0.772336487 | 0 | 0 |
| NFKBIA | -0.7761827 | 1.5404E-162 | 6.3295E-158 |
| TNFAIP3 | -0.78601553 | 1.4062E-165 | 5.7781E-161 |
| FOLR3 | -0.805813962 | 2.93493E-44 | 1.20593E-39 |
| FOS | -0.819109933 | 0 | 0 |
| RETN | -0.858188685 | 2.40138E-90 | 9.86705E-86 |
| ZFP36 | -0.931078731 | 3.3474E-261 | 1.3754E-256 |
| DUSP1 | -1.061466097 | 0 | 0 |
| S100A12 | -1.065088293 | 0 | 0 |
| NAMPT | -1.16076314 | 0 | 0 |
| SOCS3 | -1.461311341 | 0 | 0 |
