# Supplementary material for: Decoding the immune landscape following hip fracture in elderly patients: unveiling temporal dynamics through single-cell RNA sequencing
Source: Immun Ageing. 2023 Oct 17;20:54. doi: 10.1186/s12979-023-00380-6 (PMC10580557; doi:10.1186/s12979-023-00380-6)
Supplement: Supplementary file 8 — Supplementary Material 8 [file 12979_2023_380_MOESM8_ESM.docx]

**Supplementary Table 7.** Top 100 DEGs in GZMK^+^CD8^+_^cytotoxic T cells （7d post-surgery vs. 24h post-surgery）

| **GeneName** | **log2FC** | **Pvlaue** | **Qvalue** |
| --- | --- | --- | --- |
| HBB | 1.259922657 | 2.98246E-25 | 1.22546E-20 |
| H1-4 | 0.94607672 | 7.76351E-78 | 3.18995E-73 |
| XIST | 0.865867013 | 6.41821E-42 | 2.63718E-37 |
| STAT1 | 0.613767198 | 7.17315E-28 | 2.94738E-23 |
| IGHA1 | 0.60468977 | 3.43045E-39 | 1.40954E-34 |
| AHNAK | 0.599935412 | 1.73117E-47 | 7.11321E-43 |
| SYNE2 | 0.593495107 | 3.19466E-38 | 1.31266E-33 |
| MT-ND6 | 0.59174806 | 3.41725E-31 | 1.40411E-26 |
| MT-ND4L | 0.582400838 | 6.22022E-33 | 2.55583E-28 |
| ATM | 0.5451088 | 2.54671E-38 | 1.04642E-33 |
| H1-3 | 0.492970802 | 3.73292E-29 | 1.53382E-24 |
| LTB | 0.48740113 | 2.2145E-15 | 9.09918E-11 |
| TCF7 | 0.451077082 | 3.54041E-23 | 1.45472E-18 |
| MX1 | 0.448493095 | 6.88655E-19 | 2.82962E-14 |
| SLFN12L | 0.430283108 | 2.80335E-18 | 1.15187E-13 |
| MYBL1 | 0.427974729 | 1.66656E-16 | 6.84773E-12 |
| DDX3X | 0.425208399 | 9.20943E-20 | 3.78406E-15 |
| MBNL1 | 0.414758647 | 2.24615E-24 | 9.22922E-20 |
| LCP1 | 0.407817776 | 2.19863E-26 | 9.03396E-22 |
| VPS13C | 0.397949296 | 4.22883E-17 | 1.73759E-12 |
| GOLGA4 | 0.397778608 | 7.29764E-15 | 2.99853E-10 |
| SLFN5 | 0.397760878 | 5.82931E-18 | 2.3952E-13 |
| IFI44L | 0.394287579 | 1.13856E-22 | 4.67821E-18 |
| ETS1 | 0.384155726 | 2.51043E-20 | 1.03151E-15 |
| XAF1 | 0.383414666 | 3.87437E-13 | 1.59194E-08 |
| IGKC | 0.378235892 | 8.34223E-22 | 3.42774E-17 |
| IL7R | 0.377478799 | 1.73211E-14 | 7.11705E-10 |
| RIPOR2 | 0.375455566 | 6.86657E-22 | 2.8214E-17 |
| A2M-AS1 | 0.367854778 | 3.67976E-13 | 1.51198E-08 |
| TPM3 | 0.365382793 | 4.73955E-21 | 1.94743E-16 |
| ADAR | 0.360545423 | 1.12269E-15 | 4.61303E-11 |
| EZR | 0.359544964 | 1.00508E-14 | 4.12977E-10 |
| BCL2 | 0.353022181 | 8.6757E-11 | 3.56476E-06 |
| PDCD4 | 0.347709743 | 5.12994E-14 | 2.10784E-09 |
| TRIM44 | 0.345686669 | 7.33502E-13 | 3.01389E-08 |
| DDX39B | 0.345405985 | 3.77697E-15 | 1.55192E-10 |
| SEPTIN6 | 0.341016213 | 2.12945E-14 | 8.7497E-10 |
| NCL | 0.340453357 | 8.00363E-16 | 3.28861E-11 |
| RCAN3 | 0.339018993 | 3.22143E-11 | 1.32365E-06 |
| LINC00342 | 0.33807418 | 2.83609E-11 | 1.16532E-06 |
| MT-ATP8 | 0.336474379 | 2.75198E-12 | 1.13076E-07 |
| HSPA5 | 0.335198807 | 7.08912E-13 | 2.91285E-08 |
| RESF1 | 0.331131351 | 7.90328E-13 | 3.24738E-08 |
| PRPF4B | 0.325130113 | 1.27496E-11 | 5.23868E-07 |
| WBP11 | 0.322978648 | 1.77004E-12 | 7.27292E-08 |
| GLS | 0.32238453 | 2.17676E-12 | 8.94409E-08 |
| PHACTR2 | 0.319647982 | 2.29233E-09 | 9.41897E-05 |
| ENSG00000265206 | 0.318092024 | 1.8566E-10 | 7.62857E-06 |
| CTDSPL2 | 0.318028673 | 8.44632E-15 | 3.47051E-10 |
| ANKRD12 | 0.316306376 | 3.78371E-13 | 1.55469E-08 |
| HNRNPA2B1 | 0.310632467 | 4.74681E-18 | 1.95042E-13 |
| EIF2AK2 | 0.310596171 | 1.86126E-11 | 7.64773E-07 |
| HNRNPU | 0.309919358 | 4.22093E-14 | 1.73434E-09 |
| HSP90B1 | 0.308871613 | 2.14698E-11 | 8.82171E-07 |
| AAK1 | 0.307800919 | 3.89866E-13 | 1.60192E-08 |
| DTX3L | 0.304978745 | 5.36454E-10 | 2.20423E-05 |
| PHF3 | 0.304737513 | 3.04524E-09 | 0.000125126 |
| RNF213 | 0.300406128 | 9.48628E-13 | 3.89782E-08 |
| GCC2 | 0.297222892 | 1.12196E-10 | 4.61003E-06 |
| KIF2A | 0.296863697 | 7.47322E-12 | 3.07067E-07 |
| KAT6B | 0.295715963 | 6.55632E-09 | 0.000269393 |
| DDX60 | 0.292521839 | 1.50174E-09 | 6.17051E-05 |
| PTPRC | 0.291740771 | 1.06054E-21 | 4.35767E-17 |
| CD46 | 0.290298571 | 6.92199E-10 | 2.84418E-05 |
| EML4 | 0.29019821 | 3.85875E-10 | 1.58552E-05 |
| PRRC2C | 0.289064908 | 1.0531E-12 | 4.3271E-08 |
| NUB1 | 0.28891435 | 1.35185E-09 | 5.55462E-05 |
| SH2D1A | 0.288016166 | 4.42735E-12 | 1.81915E-07 |
| FAM107B | 0.287756974 | 5.11805E-11 | 2.10296E-06 |
| INPP4B | 0.285342646 | 5.98927E-09 | 0.000246093 |
| TRIM22 | 0.284824281 | 3.09301E-10 | 1.27089E-05 |
| ANKRD11 | 0.284379134 | 1.01138E-08 | 0.000415565 |
| CALR | 0.283836283 | 2.40319E-10 | 9.87447E-06 |
| IRF9 | 0.280279573 | 2.30052E-07 | 0.009452594 |
| ASH1L | 0.278541947 | 2.12607E-08 | 0.000873582 |
| EIF4G2 | 0.277306261 | 2.64472E-10 | 1.08669E-05 |
| TRIP12 | 0.275356906 | 9.92908E-09 | 0.000407976 |
| SPTBN1 | 0.27511334 | 3.54302E-08 | 0.001455792 |
| ATF7IP | 0.27356592 | 2.69372E-08 | 0.001106823 |
| MT-ND5 | 0.273528693 | 1.28681E-17 | 5.28738E-13 |
| SENP6 | 0.27347748 | 2.03006E-08 | 0.000834132 |
| UTRN | 0.273258424 | 7.82177E-09 | 0.000321389 |
| SF3B1 | 0.273062205 | 3.72339E-09 | 0.00015299 |
| RIF1 | 0.270646375 | 1.41171E-07 | 0.005800559 |
| LGALS1 | 0.270150762 | 0.000526922 | 1 |
| MACF1 | 0.269934097 | 5.98371E-09 | 0.000245865 |
| STK17B | 0.269931669 | 1.80958E-08 | 0.00074354 |
| EOMES | 0.26848367 | 9.76757E-09 | 0.00040134 |
| CEP350 | 0.268442648 | 4.07308E-09 | 0.000167359 |
| NIN | 0.268051111 | 8.00142E-09 | 0.00032877 |
| IL10RA | 0.267284627 | 1.63676E-08 | 0.000672529 |
| KLRG1 | 0.267006625 | 3.5504E-06 | 0.145882499 |
| PARP14 | 0.266850783 | 2.26501E-07 | 0.009306706 |
| CAST | 0.266632973 | 1.49353E-08 | 0.000613675 |
| HBA2 | 0.265002544 | 1.7717E-09 | 7.27975E-05 |
| ARHGAP26 | 0.264316051 | 2.07426E-11 | 8.52292E-07 |
| HBA1 | 0.264256765 | 2.29842E-07 | 0.00944399 |
| SAMD9 | 0.263859923 | 4.3516E-08 | 0.001788028 |
| CYLD | 0.263843283 | 1.95388E-09 | 8.02831E-05 |
| SETX | 0.263378284 | 4.89915E-08 | 0.002013013 |
| S100A8 | -1.123534242 | 3.1585E-111 | 1.2978E-106 |
| GZMB | -0.995263155 | 1.10975E-32 | 4.55984E-28 |
| S100A9 | -0.839668835 | 8.89606E-79 | 3.6553E-74 |
| RPS4Y1 | -0.691909975 | 2.70594E-27 | 1.11184E-22 |
| CCL4 | -0.63416062 | 1.55912E-29 | 6.40626E-25 |
| JUNB | -0.611650503 | 6.1523E-34 | 2.52792E-29 |
| CXCR4 | -0.601118158 | 3.94985E-40 | 1.62295E-35 |
| DUSP2 | -0.593678772 | 9.2745E-26 | 3.8108E-21 |
| CLIC3 | -0.555495878 | 7.42522E-21 | 3.05095E-16 |
| DDIT4 | -0.543184666 | 1.89051E-29 | 7.76793E-25 |
| DUSP1 | -0.537072851 | 1.1031E-25 | 4.53252E-21 |
| FGFBP2 | -0.513696836 | 1.183E-10 | 4.86081E-06 |
| GZMH | -0.502453632 | 1.92723E-13 | 7.91878E-09 |
| TSC22D3 | -0.486712994 | 2.99659E-28 | 1.23127E-23 |
| NFKBIA | -0.470069055 | 2.76179E-18 | 1.13479E-13 |
| TNFAIP3 | -0.466212967 | 1.52999E-18 | 6.28658E-14 |
| ID2 | -0.465571617 | 3.64544E-18 | 1.49787E-13 |
| GZMA | -0.465542472 | 1.02437E-26 | 4.20903E-22 |
| RPS10 | -0.449835379 | 1.30032E-36 | 5.34289E-32 |
| PRDM1 | -0.444906993 | 2.29583E-19 | 9.43334E-15 |
| PPDPF | -0.429515792 | 2.98784E-27 | 1.22767E-22 |
| CCL5 | -0.409979292 | 2.85637E-27 | 1.17365E-22 |
| GAPDH | -0.408767019 | 6.58168E-29 | 2.70435E-24 |
| ZFP36 | -0.407243021 | 5.58318E-16 | 2.29407E-11 |
| CD69 | -0.406670398 | 4.37321E-16 | 1.79691E-11 |
| RPL26 | -0.400060214 | 9.09468E-55 | 3.73691E-50 |
| PRF1 | -0.389950025 | 3.08133E-23 | 1.26609E-18 |
| HOPX | -0.379398663 | 6.97786E-11 | 2.86713E-06 |
| HERPUD1 | -0.376163067 | 1.23359E-14 | 5.0687E-10 |
| CYTOR | -0.375568822 | 7.73105E-14 | 3.17661E-09 |
| PRR5 | -0.373000628 | 2.07436E-15 | 8.52336E-11 |
| CCL3 | -0.372480403 | 4.35825E-21 | 1.79076E-16 |
| ZFP36L2 | -0.371866428 | 4.64287E-18 | 1.90771E-13 |
| SUB1 | -0.369884965 | 5.19621E-22 | 2.13507E-17 |
| UTS2 | -0.368192778 | 2.34557E-16 | 9.63772E-12 |
| DYNLL1 | -0.367426794 | 4.36153E-19 | 1.79211E-14 |
| BTG1 | -0.359821419 | 1.26466E-27 | 5.19636E-23 |
| DEFA3 | -0.354910823 | 5.42728E-12 | 2.23001E-07 |
| MXD4 | -0.346097849 | 1.40772E-13 | 5.78419E-09 |
| SLC25A5 | -0.343872387 | 1.56361E-16 | 6.42472E-12 |
| CEBPD | -0.342213208 | 1.30235E-12 | 5.35123E-08 |
| C12orf75 | -0.335790185 | 2.1752E-13 | 8.93769E-09 |
| TXNIP | -0.328820823 | 9.90503E-17 | 4.06988E-12 |
| ACTG1 | -0.326609556 | 8.71277E-13 | 3.57999E-08 |
| TRAC | -0.324498087 | 2.09589E-15 | 8.6118E-11 |
| RPL7 | -0.315779266 | 2.65076E-22 | 1.08917E-17 |
| H1-10 | -0.314535454 | 1.93551E-07 | 0.00795282 |
| SRGN | -0.312950102 | 4.34834E-15 | 1.78669E-10 |
| CST7 | -0.307374639 | 2.97031E-12 | 1.22047E-07 |
| LAIR2 | -0.306434566 | 2.98386E-08 | 0.001226038 |
| S100A12 | -0.301467853 | 1.90352E-22 | 7.82137E-18 |
| NEAT1 | -0.297477122 | 1.47963E-15 | 6.07966E-11 |
| LAG3 | -0.296964595 | 3.55671E-09 | 0.000146142 |
| NKG7 | -0.295201596 | 2.10429E-08 | 0.000864633 |
| PMAIP1 | -0.289355562 | 7.20316E-09 | 0.000295971 |
| HLA-DRB1 | -0.2881641 | 5.85667E-08 | 0.002406448 |
| ARPC3 | -0.284190586 | 1.63539E-15 | 6.71966E-11 |
| RGS10 | -0.282772795 | 5.89336E-09 | 0.000242152 |
| JUND | -0.280255694 | 1.2618E-13 | 5.18461E-09 |
| GMFG | -0.277629087 | 1.6966E-13 | 6.97115E-09 |
| LIMD2 | -0.274734487 | 4.73311E-12 | 1.94479E-07 |
| LINC01871 | -0.274686275 | 3.9675E-08 | 0.001630204 |
| TMEM59 | -0.274538665 | 4.05861E-11 | 1.66764E-06 |
| ARPC4 | -0.273871495 | 2.52809E-09 | 0.000103877 |
| HLA-DRA | -0.271647148 | 9.27354E-06 | 0.381040609 |
| SNHG29 | -0.271586281 | 9.57375E-12 | 3.93376E-07 |
| CD8A | -0.271570117 | 7.68572E-10 | 3.15799E-05 |
| FRG1CP | -0.271448146 | 7.06399E-08 | 0.002902521 |
| GAS5 | -0.269300497 | 1.7738E-10 | 7.28838E-06 |
| UQCRH | -0.267051045 | 3.79835E-10 | 1.5607E-05 |
| H3-3A | -0.266770674 | 3.38815E-17 | 1.39216E-12 |
| RPS3A | -0.266186167 | 2.57515E-30 | 1.0581E-25 |
| ATP5MG | -0.265893481 | 1.04933E-13 | 4.31161E-09 |
| UQCRB | -0.26454619 | 9.36719E-15 | 3.84889E-10 |
| CD52 | -0.262705455 | 1.56602E-08 | 0.000643463 |
| OSTF1 | -0.260447714 | 9.10899E-08 | 0.003742793 |
| CLIC1 | -0.260235711 | 7.89837E-11 | 3.24536E-06 |
| ATP5MC2 | -0.259624834 | 3.18555E-12 | 1.30891E-07 |
| CD38 | -0.259244686 | 4.61159E-11 | 1.89486E-06 |
| RPS24 | -0.256303996 | 1.24583E-38 | 5.11899E-34 |
| CEBPB | -0.255201372 | 1.62968E-09 | 6.6962E-05 |
| SNHG5 | -0.255167094 | 1.6498E-09 | 6.77886E-05 |
| BTF3 | -0.254692819 | 4.64218E-14 | 1.90743E-09 |
| COX7B | -0.254312013 | 7.98368E-10 | 3.28041E-05 |
| CASP4 | -0.25193682 | 1.87349E-09 | 7.69799E-05 |
| SNHG7 | -0.250855174 | 3.1398E-07 | 0.01290111 |
| PLAAT4 | -0.250220657 | 6.42788E-10 | 2.64115E-05 |
| PPP1CA | -0.250198154 | 1.58585E-10 | 6.51611E-06 |
| NDUFB5 | -0.250036749 | 2.7879E-07 | 0.011455206 |
| NDUFA12 | -0.249932672 | 1.06717E-09 | 4.3849E-05 |
| HLA-DPA1 | -0.249738642 | 1.69151E-07 | 0.006950244 |
| ABRACL | -0.247544192 | 9.54282E-08 | 0.003921049 |
| NOP10 | -0.247427685 | 3.71265E-08 | 0.001525489 |
| CD3D | -0.246867256 | 2.31997E-12 | 9.53253E-08 |
| SPON2 | -0.244763553 | 9.86251E-08 | 0.004052407 |
| TRBC2 | -0.244213086 | 1.04558E-08 | 0.000429617 |
| UCP2 | -0.242594116 | 1.02557E-10 | 4.21398E-06 |
| NDUFA1 | -0.24259379 | 8.03944E-10 | 3.30332E-05 |
| H3-3B | -0.240850586 | 3.27358E-12 | 1.34508E-07 |
| RPL13A | -0.240125446 | 1.28681E-19 | 5.28736E-15 |
