# Supplementary material for: Decoding the immune landscape following hip fracture in elderly patients: unveiling temporal dynamics through single-cell RNA sequencing
Source: Immun Ageing. 2023 Oct 17;20:54. doi: 10.1186/s12979-023-00380-6 (PMC10580557; doi:10.1186/s12979-023-00380-6)
Supplement: Supplementary file 9 — Supplementary Material 9 [file 12979_2023_380_MOESM9_ESM.docx]

**Supplementary Table 8.** Top 100 DEGs in Treg cells （7d post-surgery vs. 24h post-surgery）

| **GeneName** | **log2FC** | **Pvlaue** | **Qvalue** |
| --- | --- | --- | --- |
| H1-4 | 1.118630448 | 1.33784E-26 | 5.49704E-22 |
| MT-ND6 | 0.90840465 | 6.62237E-21 | 2.72106E-16 |
| STAT1 | 0.824733833 | 5.14409E-12 | 2.11366E-07 |
| MX1 | 0.809330501 | 6.65828E-12 | 2.73582E-07 |
| H1-3 | 0.792289282 | 3.87562E-16 | 1.59245E-11 |
| AHNAK | 0.667125386 | 5.73747E-11 | 2.35747E-06 |
| SYNE2 | 0.606241406 | 3.46291E-11 | 1.42287E-06 |
| ETS1 | 0.589179239 | 2.71003E-12 | 1.11352E-07 |
| TTN | 0.572009713 | 3.46908E-08 | 0.001425411 |
| SAMD9 | 0.57190834 | 2.96159E-09 | 0.000121689 |
| GOLGB1 | 0.565948287 | 8.22499E-08 | 0.003379566 |
| XIST | 0.54359584 | 2.63213E-06 | 0.108151472 |
| ANKRD44 | 0.537585818 | 2.89461E-10 | 1.18937E-05 |
| DDX17 | 0.533581715 | 2.56567E-11 | 1.05421E-06 |
| DDX3X | 0.529923182 | 1.10595E-08 | 0.000454424 |
| MT-ND4L | 0.523718405 | 2.25777E-07 | 0.009276935 |
| EPB41 | 0.518796017 | 2.64911E-10 | 1.08849E-05 |
| CNTRL | 0.508166274 | 2.1076E-08 | 0.000865991 |
| SLFN5 | 0.50426485 | 7.16199E-08 | 0.002942789 |
| H1-2 | 0.504166156 | 3.21457E-09 | 0.000132083 |
| SPTAN1 | 0.501716609 | 1.341E-08 | 0.000551004 |
| GLS | 0.501690395 | 2.36759E-08 | 0.000972818 |
| TRIM22 | 0.494361217 | 1.42422E-08 | 0.000585197 |
| KMT2A | 0.489269861 | 2.46092E-07 | 0.010111668 |
| HNRNPU | 0.485216018 | 2.04743E-09 | 8.4127E-05 |
| DDX39B | 0.483929154 | 4.64869E-08 | 0.001910101 |
| IFI44L | 0.481887044 | 2.69844E-08 | 0.001108761 |
| SMCHD1 | 0.465753438 | 1.26446E-07 | 0.005195535 |
| EIF2AK2 | 0.464518739 | 1.62974E-07 | 0.006696436 |
| ENSG00000265206 | 0.461379788 | 6.60166E-08 | 0.002712555 |
| VPS13C | 0.45990613 | 1.53809E-07 | 0.006319874 |
| XAF1 | 0.446234658 | 0.000143165 | 1 |
| PPM1K | 0.445757277 | 1.54074E-05 | 0.633076315 |
| PPP1R10 | 0.444624036 | 2.31561E-06 | 0.095146132 |
| CDK13 | 0.444617621 | 3.7191E-06 | 0.152814161 |
| FTX | 0.442982119 | 1.34757E-05 | 0.553701319 |
| IGHA1 | 0.442897054 | 2.35893E-06 | 0.096926092 |
| GNLY | 0.440671976 | 0.003447492 | 1 |
| PHF3 | 0.440283409 | 1.8446E-05 | 0.757926308 |
| MSL3 | 0.438164883 | 3.62211E-07 | 0.014882884 |
| LINC00342 | 0.438070087 | 2.25459E-05 | 0.926388897 |
| RIPOR2 | 0.437857056 | 5.47808E-07 | 0.022508886 |
| TMEM161B-DT | 0.431761577 | 1.78524E-05 | 0.733538465 |
| TUT4 | 0.422613365 | 1.7863E-05 | 0.733971876 |
| ZNF638 | 0.421918707 | 1.49511E-06 | 0.061432413 |
| SENP6 | 0.421302166 | 2.10982E-06 | 0.086690424 |
| MT-ATP8 | 0.416030039 | 1.45517E-05 | 0.597913766 |
| PARP14 | 0.415078136 | 6.06352E-05 | 1 |
| TTC3 | 0.414936952 | 2.44036E-05 | 1 |
| BAZ1B | 0.414738977 | 1.84416E-07 | 0.007577461 |
| INPP4B | 0.409662499 | 3.91394E-06 | 0.160819787 |
| OAS1 | 0.409241821 | 2.00422E-05 | 0.823513534 |
| MBP | 0.40901467 | 8.54905E-06 | 0.351271829 |
| BCL2 | 0.408458207 | 0.00012272 | 1 |
| NCL | 0.40766126 | 3.93668E-06 | 0.16175438 |
| RASA2 | 0.406293081 | 9.8953E-06 | 0.406588142 |
| TCF7 | 0.402597725 | 0.00033893 | 1 |
| SPTBN1 | 0.402272197 | 1.36731E-05 | 0.561815458 |
| ANKRD12 | 0.401171613 | 5.48305E-07 | 0.022529307 |
| UTRN | 0.400593289 | 3.81137E-07 | 0.015660537 |
| RNF213 | 0.399497057 | 2.6684E-05 | 1 |
| SPAG9 | 0.397957611 | 2.00996E-05 | 0.825872293 |
| ATRX | 0.394450709 | 4.7481E-05 | 1 |
| WAPL | 0.392024188 | 9.03445E-05 | 1 |
| USP15 | 0.390853621 | 1.43239E-05 | 0.58855484 |
| GCC2 | 0.389865454 | 0.000151753 | 1 |
| IKZF2 | 0.3887238 | 5.17546E-05 | 1 |
| ATF7IP | 0.388600658 | 6.84142E-09 | 0.000281107 |
| SF3B1 | 0.388212282 | 1.21865E-05 | 0.500729623 |
| SLFN12L | 0.387845063 | 0.000311166 | 1 |
| PPBP | 0.385071094 | 2.90956E-05 | 1 |
| CREBZF | 0.382745723 | 4.98288E-05 | 1 |
| DDX60 | 0.382065958 | 6.95387E-05 | 1 |
| IRF1 | 0.379550699 | 0.000206126 | 1 |
| GPRIN3 | 0.374447144 | 0.000165731 | 1 |
| RPS6KA5 | 0.371129749 | 2.38242E-05 | 0.978912128 |
| SETD2 | 0.366697879 | 0.000381602 | 1 |
| XPO1 | 0.366687488 | 0.000254833 | 1 |
| ANKRD11 | 0.365614454 | 0.000134591 | 1 |
| APOL6 | 0.364788187 | 1.9447E-05 | 0.799056001 |
| SLC12A6 | 0.364099798 | 1.43923E-05 | 0.591364178 |
| DYNC1H1 | 0.363940196 | 1.3786E-06 | 0.056645452 |
| MACF1 | 0.363433148 | 0.000567548 | 1 |
| ZNF37BP | 0.36289413 | 0.00040628 | 1 |
| GOLGA4 | 0.361663649 | 0.000115882 | 1 |
| MAP3K1 | 0.360865717 | 0.000217903 | 1 |
| CLIP1 | 0.359383803 | 0.000236664 | 1 |
| ZNF91 | 0.358851798 | 4.02583E-06 | 0.165417198 |
| YTHDC1 | 0.357579411 | 0.000646789 | 1 |
| JAK1 | 0.353912965 | 2.07405E-06 | 0.085220436 |
| USP34 | 0.353450793 | 8.68348E-05 | 1 |
| SEMA4D | 0.353356592 | 0.000178984 | 1 |
| NIN | 0.352618392 | 0.000467257 | 1 |
| TIAM1 | 0.352501117 | 1.84828E-05 | 0.759441225 |
| AKAP9 | 0.351306498 | 0.00097038 | 1 |
| ISG15 | 0.35087433 | 1.95657E-07 | 0.00803937 |
| PDE3B | 0.349592363 | 1.61355E-05 | 0.662990859 |
| RESF1 | 0.349470191 | 6.63051E-05 | 1 |
| N4BP2L2 | 0.349214946 | 7.58756E-07 | 0.031176519 |
| TRIM38 | 0.347898413 | 1.078E-05 | 0.442937808 |
| ATP5MF | -0.294606394 | 0.000259476 | 1 |
| ENSG00000272211 | -0.295785981 | 3.60759E-06 | 0.148232297 |
| CHMP2A | -0.297573412 | 5.38711E-05 | 1 |
| CHCHD10 | -0.298689978 | 5.52149E-05 | 1 |
| ABRACL | -0.300175289 | 0.000717742 | 1 |
| SEC61B | -0.301862844 | 0.000310942 | 1 |
| FKBP1A | -0.302274852 | 2.48838E-06 | 0.102244996 |
| SLC25A3 | -0.302814481 | 0.000131211 | 1 |
| COX6A1 | -0.302822502 | 0.000341133 | 1 |
| DYNLL1 | -0.304828205 | 0.001731304 | 1 |
| RPL22L1 | -0.30572787 | 0.001031406 | 1 |
| COX5B | -0.306313772 | 2.21112E-05 | 0.908527913 |
| SEC61G | -0.306443279 | 3.54675E-05 | 1 |
| ATP5F1C | -0.306975339 | 0.00093385 | 1 |
| JUND | -0.307075226 | 3.85708E-05 | 1 |
| GAPDH | -0.307220701 | 0.00061295 | 1 |
| RPL7 | -0.30731951 | 6.98616E-07 | 0.028705432 |
| TBCA | -0.307858977 | 0.000790324 | 1 |
| CLTB | -0.307931034 | 0.000197137 | 1 |
| RPS24 | -0.308169874 | 7.97969E-13 | 3.27878E-08 |
| ENSG00000267737 | -0.308292618 | 2.0082E-11 | 8.25149E-07 |
| JTB | -0.309349524 | 0.000108095 | 1 |
| RPL36A | -0.309488955 | 0.000134021 | 1 |
| EIF3K | -0.312291283 | 1.50902E-05 | 0.620041156 |
| NDUFA12 | -0.312537435 | 0.00065462 | 1 |
| ATP5PB | -0.315075882 | 0.001065931 | 1 |
| CD53 | -0.319793778 | 0.000257268 | 1 |
| COX6C | -0.321802022 | 1.44262E-05 | 0.592756918 |
| ITM2A | -0.32253912 | 0.00289727 | 1 |
| C12orf75 | -0.323281736 | 1.02965E-05 | 0.423074569 |
| ENSG00000271204 | -0.323491771 | 0.000164882 | 1 |
| ARPC4 | -0.325322942 | 0.000367366 | 1 |
| SF3B6 | -0.327660559 | 0.000224427 | 1 |
| CLIC1 | -0.329154158 | 0.000194949 | 1 |
| COX7B | -0.331005886 | 7.74263E-06 | 0.318137048 |
| CKLF | -0.332867915 | 5.21474E-05 | 1 |
| MYL12A | -0.335499135 | 9.52168E-09 | 0.000391236 |
| PLGRKT | -0.335672924 | 1.33291E-05 | 0.547680182 |
| GNG5 | -0.337694988 | 1.09986E-06 | 0.045192013 |
| SUMO2 | -0.339652966 | 2.69341E-08 | 0.001106694 |
| ARPC3 | -0.341120934 | 6.07955E-06 | 0.249802488 |
| PSMB3 | -0.341931139 | 3.47609E-05 | 1 |
| ATP5MC3 | -0.343812799 | 1.17614E-06 | 0.048326452 |
| NFKBIA | -0.344375266 | 3.93206E-05 | 1 |
| COX8A | -0.345781366 | 6.9691E-06 | 0.286353442 |
| RPL10P9 | -0.348810502 | 4.66787E-11 | 1.91798E-06 |
| BTG1 | -0.348918543 | 6.04221E-08 | 0.002482684 |
| DCTN3 | -0.35257457 | 3.10583E-06 | 0.12761565 |
| TSPO | -0.358244424 | 7.94359E-06 | 0.32639411 |
| SRGN | -0.361227331 | 7.47019E-06 | 0.306942678 |
| SLC2A3 | -0.362574622 | 3.43409E-05 | 1 |
| JPT1 | -0.363528749 | 4.81908E-05 | 1 |
| OSTF1 | -0.364999752 | 1.17975E-06 | 0.048474737 |
| CD70 | -0.367181447 | 0.000104458 | 1 |
| UQCR11 | -0.369308591 | 7.26825E-06 | 0.298644971 |
| TMSB10 | -0.375185699 | 5.36429E-12 | 2.20413E-07 |
| GMFG | -0.380091638 | 3.74826E-07 | 0.015401224 |
| CYBA | -0.380598183 | 1.56279E-08 | 0.000642133 |
| TMEM59 | -0.382503013 | 4.34834E-07 | 0.017866914 |
| CTSC | -0.384343399 | 9.32244E-06 | 0.38304983 |
| SAT1 | -0.390685048 | 1.14935E-05 | 0.472255114 |
| LDHA | -0.392162027 | 1.28382E-06 | 0.052750716 |
| KLRB1 | -0.398478854 | 0.084690802 | 1 |
| BBLN | -0.404231737 | 4.05052E-07 | 0.016643165 |
| CXCR4 | -0.405284177 | 5.0463E-06 | 0.207347263 |
| SERF2 | -0.406513833 | 2.55571E-15 | 1.05012E-10 |
| CORO1B | -0.406751032 | 5.84488E-05 | 1 |
| TSC22D3 | -0.410715564 | 3.36922E-07 | 0.013843779 |
| S100A4 | -0.410954469 | 1.2593E-06 | 0.051743362 |
| NAMPT | -0.411749724 | 9.83193E-07 | 0.04039843 |
| H3-3A | -0.416559176 | 7.15134E-10 | 2.93841E-05 |
| RPS27L | -0.423824887 | 2.15206E-06 | 0.088426177 |
| S100A8 | -0.424947708 | 1.09587E-11 | 4.50282E-07 |
| SUB1 | -0.426395919 | 4.37331E-09 | 0.000179695 |
| COTL1 | -0.427286338 | 4.83335E-06 | 0.198597466 |
| NDUFA13 | -0.428263945 | 3.86271E-08 | 0.00158715 |
| ACTB | -0.430464321 | 6.12542E-08 | 0.002516876 |
| CYTOR | -0.431161242 | 9.45107E-05 | 1 |
| RPL26 | -0.439570271 | 5.9765E-22 | 2.45568E-17 |
| LGALS1 | -0.44825117 | 0.001798283 | 1 |
| BAX | -0.450791474 | 5.66217E-08 | 0.002326529 |
| TXN | -0.454783573 | 1.06992E-08 | 0.000439618 |
| MYL12B | -0.468493405 | 4.21486E-13 | 1.73184E-08 |
| MYL6 | -0.482017396 | 1.78174E-11 | 7.321E-07 |
| ARPC1B | -0.494947105 | 3.53986E-10 | 1.45449E-05 |
| SLC25A5 | -0.496650089 | 2.99355E-09 | 0.000123002 |
| S100A9 | -0.507107347 | 2.46187E-09 | 0.000101156 |
| ZFP36L2 | -0.519369686 | 4.91945E-10 | 2.02135E-05 |
| ACTG1 | -0.528081979 | 2.37204E-07 | 0.009746458 |
| DDIT4 | -0.584958122 | 3.96172E-11 | 1.62783E-06 |
| RPS10 | -0.590266088 | 2.5902E-18 | 1.06429E-13 |
| RGS1 | -0.609121423 | 1.71997E-09 | 7.06718E-05 |
| PMAIP1 | -0.712590969 | 1.92293E-13 | 7.90111E-09 |
| SOCS3 | -0.734347252 | 2.06E-20 | 8.46435E-16 |
| CD69 | -0.76442806 | 2.01628E-17 | 8.28467E-13 |
| JUNB | -0.777705687 | 1.45324E-19 | 5.97123E-15 |
| PRDM1 | -0.868087685 | 4.48111E-24 | 1.84124E-19 |
| HBA1 | -1.399996262 | 0.003125787 | 1 |
| HBA2 | -1.620959617 | 1.31226E-05 | 0.53919392 |
| HBB | -3.501756726 | 2.71792E-22 | 1.11676E-17 |
