# Supplementary material for: Decoding the immune landscape following hip fracture in elderly patients: unveiling temporal dynamics through single-cell RNA sequencing
Source: Immun Ageing. 2023 Oct 17;20:54. doi: 10.1186/s12979-023-00380-6 (PMC10580557; doi:10.1186/s12979-023-00380-6)
Supplement: Supplementary file 10 — Supplementary Material 10 [file 12979_2023_380_MOESM10_ESM.docx]

**Supplementary Table 9.** Top 100 DEGs in CD4 naiveT cells （7d post-surgery vs. 24h post-surgery）

| **GeneName** | **log2FC** | **Pvlaue** | **Qvalue** |
| --- | --- | --- | --- |
| MT-RNR2 | 0.763901365 | 7.8079E-168 | 3.2082E-163 |
| IGKC | 0.672855266 | 7.6876E-129 | 3.1588E-124 |
| MTATP6P1 | 0.64534641 | 4.3916E-180 | 1.8045E-175 |
| MT-RNR1 | 0.551423709 | 1.97779E-98 | 8.12653E-94 |
| ENSG00000289474 | 0.511662325 | 5.42798E-56 | 2.2303E-51 |
| SOCS3 | 0.497894525 | 9.10985E-81 | 3.74314E-76 |
| CD69 | 0.469772604 | 4.52322E-72 | 1.85855E-67 |
| MT-CO3 | 0.420383602 | 8.579E-219 | 3.525E-214 |
| H1-3 | 0.302603411 | 3.73456E-30 | 1.53449E-25 |
| RPL41P2 | 0.300778917 | 3.27559E-36 | 1.34591E-31 |
| RPS4X | 0.29821819 | 7.9745E-224 | 3.2766E-219 |
| IGLC2 | 0.273043585 | 8.00558E-38 | 3.28941E-33 |
| DEFA3 | 0.247054708 | 1.34631E-40 | 5.53187E-36 |
| EEF1B2 | 0.240975847 | 1.28917E-95 | 5.29706E-91 |
| IGHA2 | 0.238858825 | 7.67191E-51 | 3.15231E-46 |
| MT-CO1 | 0.237169527 | 2.6619E-112 | 1.0938E-107 |
| RPL34 | 0.234604986 | 5.653E-245 | 2.3228E-240 |
| ENSG00000213058 | 0.214227306 | 3.11579E-26 | 1.28025E-21 |
| RPS8 | 0.211670236 | 1.341E-146 | 5.5099E-142 |
| RPS27A | 0.209813568 | 3.5361E-212 | 1.4529E-207 |
| SNHG29 | 0.209723714 | 9.73669E-34 | 4.00071E-29 |
| SOCS1 | 0.207672718 | 1.15313E-18 | 4.7381E-14 |
| RPS12 | 0.19582613 | 5.1875E-158 | 2.1315E-153 |
| RPL22 | 0.19344663 | 2.05256E-48 | 8.43378E-44 |
| ENSG00000237550 | 0.192130576 | 5.17268E-18 | 2.1254E-13 |
| RPS14 | 0.182075494 | 5.6618E-185 | 2.3264E-180 |
| RPS5 | 0.180479413 | 1.23612E-71 | 5.0791E-67 |
| RPL19 | 0.176494387 | 1.0806E-127 | 4.4402E-123 |
| RPL10 | 0.175521255 | 5.0548E-95 | 2.07697E-90 |
| RPS3 | 0.174803848 | 3.813E-112 | 1.5667E-107 |
| TPT1 | 0.170204642 | 2.9347E-119 | 1.2058E-114 |
| MZT2B | 0.169878607 | 5.23009E-16 | 2.14899E-11 |
| MT2A | 0.168941331 | 4.83397E-09 | 0.000198623 |
| RPL10P9 | 0.168438749 | 1.32848E-64 | 5.4586E-60 |
| NACA | 0.165745134 | 3.27235E-64 | 1.34458E-59 |
| H1-10 | 0.165038448 | 1.76998E-19 | 7.27269E-15 |
| RPL32 | 0.16430333 | 1.2577E-104 | 5.16768E-100 |
| CRYBG1 | 0.163950546 | 6.82582E-12 | 2.80466E-07 |
| RPL39 | 0.161736688 | 6.2441E-118 | 2.5656E-113 |
| RPS3A | 0.161176214 | 1.14063E-79 | 4.68674E-75 |
| IGLC3 | 0.160749951 | 2.4386E-30 | 1.002E-25 |
| IGHG2 | 0.15616227 | 5.46703E-55 | 2.24635E-50 |
| IGHA1 | 0.15538613 | 4.13699E-18 | 1.69985E-13 |
| MT-CYB | 0.148999443 | 2.72739E-30 | 1.12066E-25 |
| ENSG00000227615 | 0.148875034 | 1.21413E-12 | 4.98873E-08 |
| RPL35A | 0.148536091 | 8.33096E-86 | 3.42311E-81 |
| RPL29 | 0.148316856 | 2.07647E-72 | 8.53201E-68 |
| MT-CO2 | 0.147504316 | 1.51769E-27 | 6.23605E-23 |
| ENSG00000268027 | 0.147115424 | 3.39622E-10 | 1.39547E-05 |
| RPL6 | 0.144849663 | 6.31296E-58 | 2.59393E-53 |
| RPL11 | 0.142264283 | 1.00252E-97 | 4.11925E-93 |
| RPL18 | 0.142245991 | 1.35535E-74 | 5.56898E-70 |
| UQCRB | 0.141725405 | 1.8692E-18 | 7.68036E-14 |
| RPL37 | 0.138704722 | 3.88906E-86 | 1.59798E-81 |
| RPS28 | 0.138219719 | 1.55315E-93 | 6.38173E-89 |
| C12orf57 | 0.136182877 | 6.22525E-10 | 2.55789E-05 |
| PNRC1 | 0.134030078 | 9.0763E-10 | 3.72936E-05 |
| SNRPD2 | 0.132777948 | 7.66926E-13 | 3.15122E-08 |
| RPS15A | 0.132634654 | 2.91618E-92 | 1.19823E-87 |
| RPS18 | 0.131909056 | 3.63949E-62 | 1.49543E-57 |
| GAS5 | 0.131026796 | 1.42349E-17 | 5.84897E-13 |
| IFITM1 | 0.130914805 | 2.85096E-07 | 0.011714292 |
| ENSG00000286646 | 0.130868609 | 2.85147E-14 | 1.17164E-09 |
| RPL10A | 0.12776959 | 1.60856E-57 | 6.60941E-53 |
| RBIS | 0.125738902 | 2.85657E-10 | 1.17374E-05 |
| PRKCQ-AS1 | 0.125496598 | 6.12863E-07 | 0.025181943 |
| RPLP1 | 0.124032357 | 6.21544E-38 | 2.55386E-33 |
| RPS23 | 0.119819534 | 3.61595E-68 | 1.48576E-63 |
| RPS6 | 0.11855881 | 5.47055E-62 | 2.24779E-57 |
| TOMM7 | 0.118006426 | 8.37899E-24 | 3.44284E-19 |
| EEF1D | 0.117335289 | 6.31754E-16 | 2.59582E-11 |
| RPL13 | 0.116085376 | 7.55806E-93 | 3.10553E-88 |
| MT-ATP8 | 0.116081982 | 2.09965E-09 | 8.62724E-05 |
| ENSG00000288826 | 0.113063075 | 7.13946E-12 | 2.93353E-07 |
| COMMD6 | 0.112273833 | 4.65342E-10 | 1.91204E-05 |
| CRIP1 | 0.11225449 | 0.000119742 | 1 |
| RPL8 | 0.110447397 | 5.37357E-37 | 2.20795E-32 |
| JCHAIN | 0.109918964 | 2.59941E-22 | 1.06807E-17 |
| COX4I1 | 0.109818053 | 1.63426E-13 | 6.71499E-09 |
| RPL3 | 0.109500372 | 2.38745E-60 | 9.8098E-56 |
| RPS13 | 0.107615529 | 3.40114E-37 | 1.39749E-32 |
| PRDM1 | 0.107452971 | 4.95463E-05 | 1 |
| RPL21 | 0.106969313 | 6.82679E-81 | 2.80506E-76 |
| TMEM156 | 0.106544444 | 1.36155E-06 | 0.055944932 |
| FAU | 0.102940145 | 1.1299E-42 | 4.64263E-38 |
| ARID5B | 0.102557205 | 0.001682079 | 1 |
| H1-4 | 0.100634579 | 8.62033E-16 | 3.54201E-11 |
| RPS25 | 0.100207419 | 2.67201E-41 | 1.0979E-36 |
| UXT | 0.099629098 | 3.88666E-06 | 0.159698769 |
| RPS27 | 0.09902298 | 3.35015E-37 | 1.37654E-32 |
| ICAM3 | 0.097286792 | 0.000117254 | 1 |
| C7orf50 | 0.096400309 | 2.59984E-18 | 1.06825E-13 |
| RPL30 | 0.095452794 | 1.61782E-65 | 6.64747E-61 |
| RPLP2 | 0.094692252 | 7.7796E-52 | 3.19656E-47 |
| CLEC2B | 0.093110431 | 8.5903E-05 | 1 |
| RPL24 | 0.091203385 | 1.63519E-37 | 6.71885E-33 |
| ANXA2R | 0.090655279 | 0.000172427 | 1 |
| RPL12P4 | 0.090284044 | 9.61717E-08 | 0.003951598 |
| UBA52 | 0.089321414 | 2.17272E-32 | 8.92748E-28 |
| LSM5 | 0.088946488 | 9.64679E-06 | 0.396377125 |
| GOLGA4 | -0.194430034 | 1.76001E-17 | 7.23172E-13 |
| S100A12 | -0.195871664 | 3.1206E-17 | 1.28222E-12 |
| MARK3 | -0.196394776 | 1.29125E-20 | 5.30562E-16 |
| NAMPT | -0.197731442 | 1.36003E-39 | 5.58822E-35 |
| HLA-E | -0.198300007 | 1.50438E-25 | 6.18136E-21 |
| DYNC1H1 | -0.199236383 | 1.8999E-22 | 7.80648E-18 |
| TSC22D3 | -0.199397126 | 2.63294E-16 | 1.08185E-11 |
| PDE7A | -0.199562162 | 4.18862E-27 | 1.72106E-22 |
| MCL1 | -0.200044926 | 3.32069E-19 | 1.36444E-14 |
| KCNQ1OT1 | -0.20031225 | 9.17697E-16 | 3.77072E-11 |
| KLF6 | -0.200776142 | 6.15694E-16 | 2.52982E-11 |
| SLC25A5 | -0.201716084 | 1.6112E-20 | 6.62026E-16 |
| CDC42SE1 | -0.201801415 | 7.44781E-36 | 3.06023E-31 |
| PTP4A2 | -0.201987598 | 4.02438E-20 | 1.65358E-15 |
| LRRFIP1 | -0.202162189 | 5.54706E-19 | 2.27923E-14 |
| LSP1 | -0.202268837 | 3.36178E-26 | 1.38132E-21 |
| PRKCA | -0.202471946 | 1.93126E-24 | 7.93533E-20 |
| S100A8 | -0.202685011 | 8.99812E-26 | 3.69724E-21 |
| KMT2C | -0.203059623 | 5.96163E-20 | 2.44958E-15 |
| KANSL1 | -0.203134732 | 1.46585E-24 | 6.02305E-20 |
| TRBC1 | -0.203367856 | 1.48499E-07 | 0.006101682 |
| PSMA3-AS1 | -0.203407104 | 7.61721E-22 | 3.12983E-17 |
| PUM1 | -0.203478823 | 4.58782E-29 | 1.88509E-24 |
| GNAI2 | -0.204224043 | 2.79481E-25 | 1.14836E-20 |
| AKAP13 | -0.205551818 | 7.25681E-20 | 2.98175E-15 |
| TUBA1A | -0.205889896 | 1.01099E-36 | 4.15408E-32 |
| TSPAN14 | -0.205998964 | 2.85935E-23 | 1.17488E-18 |
| SYNE2 | -0.206476506 | 1.0937E-19 | 4.49391E-15 |
| ZC3HAV1 | -0.20698246 | 1.02864E-22 | 4.22659E-18 |
| BCL11B | -0.207833051 | 1.21732E-24 | 5.00186E-20 |
| ZBTB7A | -0.208969588 | 2.94926E-20 | 1.21182E-15 |
| PRKCB | -0.209192328 | 3.43142E-24 | 1.40994E-19 |
| ZFP36L1 | -0.209612372 | 1.83516E-18 | 7.54049E-14 |
| ZNF207 | -0.209976488 | 1.39901E-22 | 5.7484E-18 |
| HLA-C | -0.210003426 | 1.4696E-34 | 6.03842E-30 |
| TMEM63A | -0.21070682 | 2.9205E-28 | 1.2E-23 |
| SATB1 | -0.210928687 | 1.17097E-22 | 4.8114E-18 |
| RPS17 | -0.211646135 | 1.26327E-22 | 5.19064E-18 |
| ZNF292 | -0.215393461 | 1.18622E-21 | 4.87404E-17 |
| KPNB1 | -0.216221488 | 1.12045E-22 | 4.60382E-18 |
| SERINC5 | -0.217092442 | 3.61382E-25 | 1.48488E-20 |
| SAMHD1 | -0.21822857 | 8.12863E-23 | 3.33997E-18 |
| CST3 | -0.218441879 | 7.11012E-34 | 2.92148E-29 |
| MACF1 | -0.218666807 | 4.31072E-21 | 1.77123E-16 |
| CREBRF | -0.218944133 | 5.92038E-26 | 2.43262E-21 |
| KMT2A | -0.220226237 | 2.97042E-25 | 1.22052E-20 |
| FAM153CP | -0.220913815 | 7.86602E-20 | 3.23207E-15 |
| IL32 | -0.220963368 | 7.21032E-17 | 2.96265E-12 |
| ATP1A1 | -0.221163778 | 7.74943E-25 | 3.18416E-20 |
| SLC38A2 | -0.223330499 | 3.49118E-23 | 1.43449E-18 |
| TNRC6B | -0.223373734 | 9.09297E-22 | 3.73621E-17 |
| ANKRD12 | -0.223525432 | 3.74218E-21 | 1.53762E-16 |
| ERAP2 | -0.224760489 | 7.99492E-24 | 3.28503E-19 |
| AKNA | -0.225709984 | 5.10192E-29 | 2.09633E-24 |
| SPN | -0.22636962 | 8.22837E-23 | 3.38096E-18 |
| C1orf56 | -0.227531668 | 7.77631E-59 | 3.19521E-54 |
| LUC7L3 | -0.229859878 | 5.46474E-25 | 2.24541E-20 |
| YIPF4 | -0.23076455 | 6.38287E-25 | 2.62266E-20 |
| ANKRD44 | -0.231570262 | 3.17122E-24 | 1.30302E-19 |
| UCP2 | -0.232318995 | 1.15469E-31 | 4.74449E-27 |
| DDX17 | -0.233071907 | 1.18823E-36 | 4.8823E-32 |
| SNHG14 | -0.233529736 | 2.05776E-23 | 8.45515E-19 |
| NFATC2IP | -0.234123494 | 2.08366E-27 | 8.56157E-23 |
| PCBP1 | -0.234614963 | 3.89413E-33 | 1.60006E-28 |
| LINC00342 | -0.237566071 | 2.66958E-27 | 1.0969E-22 |
| ANKRD11 | -0.23863654 | 1.03434E-23 | 4.24999E-19 |
| IER2 | -0.239318308 | 1.4492E-22 | 5.95464E-18 |
| ARID1B | -0.242799597 | 3.66865E-26 | 1.50741E-21 |
| FKBP5 | -0.243493135 | 4.63934E-27 | 1.90626E-22 |
| RPL23 | -0.244012713 | 2.01969E-93 | 8.29872E-89 |
| RASGRP1 | -0.244070594 | 3.86315E-25 | 1.58733E-20 |
| CCR7 | -0.244082641 | 1.301E-20 | 5.34566E-16 |
| ADD3 | -0.246506143 | 3.39219E-28 | 1.39382E-23 |
| TCF25 | -0.248875653 | 1.27806E-35 | 5.2514E-31 |
| XIST | -0.249544111 | 7.78912E-51 | 3.20047E-46 |
| ACAP1 | -0.253027379 | 1.40564E-33 | 5.77563E-29 |
| PPP2R5C | -0.253856492 | 4.12056E-27 | 1.6931E-22 |
| GABPB1-AS1 | -0.255144345 | 1.53219E-25 | 6.2956E-21 |
| DAZAP2 | -0.25831852 | 4.9517E-31 | 2.0346E-26 |
| ATM | -0.262843069 | 1.69991E-29 | 6.98475E-25 |
| SORL1 | -0.266910352 | 1.55558E-32 | 6.39172E-28 |
| HLA-B | -0.267366666 | 3.73137E-73 | 1.53318E-68 |
| CELF2 | -0.270723801 | 3.44756E-32 | 1.41657E-27 |
| FOXP1 | -0.273389422 | 7.04072E-33 | 2.89296E-28 |
| SLFN12L | -0.273414063 | 1.47258E-32 | 6.05069E-28 |
| SMCHD1 | -0.288197374 | 5.99694E-34 | 2.46408E-29 |
| CALR | -0.289143453 | 9.30909E-42 | 3.82501E-37 |
| MYH9 | -0.2902931 | 2.41145E-41 | 9.9084E-37 |
| MT-ND2 | -0.297449084 | 1.55605E-66 | 6.39367E-62 |
| PCSK7 | -0.297586836 | 2.43652E-31 | 1.00114E-26 |
| HBB | -0.305363706 | 5.2531E-147 | 2.1584E-142 |
| FYB1 | -0.311100254 | 2.8365E-57 | 1.16549E-52 |
| H4C3 | -0.314121044 | 1.80471E-48 | 7.41537E-44 |
| RNF213 | -0.336898343 | 3.10039E-49 | 1.27392E-44 |
| AAK1 | -0.339140235 | 1.0798E-54 | 4.43679E-50 |
| ZFP36L2 | -0.376485313 | 4.98468E-59 | 2.04816E-54 |
| LYZ | -0.384108327 | 1.97711E-60 | 8.12376E-56 |
| POLR2J3.1 | -0.409084283 | 2.0247E-61 | 8.3193E-57 |
| NEAT1 | -0.472789541 | 2.38784E-71 | 9.81138E-67 |
| S100A9 | -0.705629717 | 3.7727E-200 | 1.5502E-195 |
