# Supplementary material for: Decoding the immune landscape following hip fracture in elderly patients: unveiling temporal dynamics through single-cell RNA sequencing
Source: Immun Ageing. 2023 Oct 17;20:54. doi: 10.1186/s12979-023-00380-6 (PMC10580557; doi:10.1186/s12979-023-00380-6)
Supplement: Supplementary file 11 — Supplementary Material 11 [file 12979_2023_380_MOESM11_ESM.docx]

**Supplementary Table 10.** Top 20 (means) interacting pair (24 hours after trauma)

| **interacting pair** | **HLA-C_KIR2DL1** | **CCL5_CCR1** | **CD52_SIGLEC10** | **CD48_CD244** | **HLA-E_KLRC1** | **HLA-E_KLRC2** | **HLA-E_KLRK1** | **CD94:NKG2A_HLA-E** | **CD94:NKG2C_HLA-E** | **BSG_PPIA** | **PTPRC_CD22** | **PTPRC_SEMA4D** | **HLA-A_KIR3DL1** | **HLA-C_FAM3C** | **CD74_APP** | **CD74_MIF** | **HLA-B_KIR3DL2** | **CD74_COPA** | **C5AR1_RPS19** | **TYROBP_CD44** |
| --- | --- | --- | --- | --- | --- | --- | --- | --- | --- | --- | --- | --- | --- | --- | --- | --- | --- | --- | --- | --- |
| C-Mono1\|C-Mono1 | 0.00 | 0.44 | 0.96 | 0.95 | 1.01 | 1.01 | 0.00 | 1.01 | 1.01 | 1.20 | 1.13 | 1.30 | 0.00 | 1.42 | 1.62 | 1.67 | 1.62 | 1.84 | 2.04 | 2.398 |
| C-Mono1\|C-Mono2 | 1.38 | 0.40 | 0.91 | 0.93 | 1.01 | 1.01 | 1.01 | 0.93 | 0.93 | 0.99 | 1.13 | 1.30 | 1.34 | 1.40 | 1.61 | 1.60 | 1.62 | 1.77 | 1.87 | 2.385 |
| C-Mono1\|C-Mono3 | 0.00 | 0.52 | 0.91 | 0.93 | 1.01 | 1.01 | 1.01 | 1.03 | 1.03 | 1.06 | 1.13 | 1.31 | 1.34 | 1.40 | 1.60 | 1.61 | 1.62 | 1.79 | 1.90 | 2.387 |
| C-Mono1\|CD4+_effector_T_cell | 1.38 | 0.08 | 0.84 | 0.92 | 1.01 | 1.01 | 1.01 | 1.31 | 1.31 | 1.30 | 0.00 | 1.39 | 1.34 | 1.43 | 1.46 | 1.80 | 1.62 | 1.64 | 2.38 | 1.992 |
| C-Mono1\|CD4+_memory_T_cell | 1.38 | 0.08 | 0.84 | 0.88 | 1.01 | 1.01 | 1.01 | 1.23 | 1.23 | 1.21 | 1.13 | 1.42 | 1.34 | 1.42 | 1.49 | 1.80 | 1.63 | 1.63 | 2.41 | 2.091 |
| C-Mono1\|CD4+_naÃ¯ve_T_cell | 1.38 | 0.08 | 0.83 | 0.88 | 1.01 | 1.01 | 1.01 | 1.09 | 1.09 | 1.13 | 1.13 | 1.38 | 1.34 | 1.41 | 1.52 | 1.81 | 1.62 | 1.58 | 2.51 | 1.979 |
| C-Mono1\|CD56brightCD16-_NK | 1.39 | 0.19 | 0.85 | 0.98 | 1.73 | 1.28 | 1.08 | 1.27 | 1.27 | 1.25 | 0.00 | 1.37 | 1.35 | 1.43 | 1.46 | 1.83 | 1.67 | 1.69 | 2.33 | 2.14 |
| C-Mono1\|CD56dimCD16+_NK | 1.41 | 0.09 | 0.85 | 1.00 | 1.18 | 1.16 | 1.06 | 1.42 | 1.42 | 1.27 | 1.13 | 1.40 | 1.39 | 1.47 | 1.46 | 1.74 | 1.73 | 1.61 | 2.13 | 1.75 |
| C-Mono1\|CD8+_naÃ¯ve_T_cell | 1.39 | 0.09 | 0.84 | 0.91 | 1.07 | 1.07 | 1.03 | 1.26 | 1.26 | 1.21 | 1.13 | 1.44 | 1.35 | 1.43 | 1.51 | 1.85 | 1.64 | 1.63 | 2.46 | 1.956 |
| C-Mono1\|GNLY+CD8+_cytotoxic_T_cell | 1.38 | 0.09 | 0.84 | 0.95 | 1.03 | 1.09 | 1.05 | 1.32 | 1.32 | 1.26 | 1.13 | 1.38 | 1.36 | 1.43 | 1.46 | 1.76 | 1.66 | 1.62 | 2.33 | 1.9 |
| C-Mono1\|GZMK+CD8+_cytotoxic_T_cell | 1.38 | 0.10 | 0.83 | 0.93 | 1.04 | 1.03 | 1.04 | 1.25 | 1.25 | 1.21 | 1.13 | 1.40 | 1.34 | 1.42 | 1.46 | 1.79 | 1.62 | 1.62 | 2.41 | 2.009 |
| C-Mono1\|MAI_T | 0.00 | 0.16 | 0.84 | 0.91 | 1.07 | 1.01 | 1.02 | 1.27 | 1.27 | 1.21 | 0.00 | 1.35 | 0.00 | 1.47 | 1.46 | 1.77 | 0.00 | 1.65 | 2.40 | 1.972 |
| C-Mono1\|Memory_B_cell | 0.00 | 0.09 | 0.90 | 0.88 | 0.00 | 0.00 | 1.01 | 1.18 | 1.18 | 1.29 | 1.51 | 1.22 | 0.00 | 1.52 | 1.50 | 1.78 | 1.62 | 1.63 | 2.42 | 1.98 |
| C-Mono1\|NC-Mono | 1.38 | 0.34 | 1.05 | 0.96 | 1.01 | 0.00 | 0.00 | 1.00 | 1.00 | 1.12 | 1.14 | 1.27 | 0.00 | 1.42 | 1.56 | 1.65 | 1.62 | 1.76 | 2.14 | 2.2 |
| C-Mono1\|NKT | 1.39 | 0.09 | 0.85 | 0.99 | 1.15 | 1.16 | 1.06 | 1.39 | 1.39 | 1.32 | 0.00 | 1.47 | 1.38 | 1.47 | 1.46 | 1.80 | 1.70 | 1.63 | 2.28 | 1.888 |
| C-Mono1\|NaÃ¯ve_B_cell | 0.00 | 0.08 | 0.87 | 0.88 | 1.01 | 1.01 | 1.01 | 1.07 | 1.07 | 1.20 | 1.66 | 1.21 | 1.34 | 1.56 | 1.54 | 1.68 | 1.62 | 1.59 | 2.50 | 1.821 |
| C-Mono1\|Plasma | 0.00 | 0.08 | 0.00 | 0.88 | 1.02 | 0.00 | 0.00 | 0.67 | 0.67 | 1.19 | 1.15 | 1.23 | 0.00 | 1.47 | 1.46 | 1.95 | 0.00 | 1.66 | 2.04 | 1.966 |
| C-Mono1\|Treg | 0.00 | 0.08 | 0.84 | 0.88 | 0.00 | 0.00 | 1.01 | 1.26 | 1.26 | 1.29 | 1.13 | 1.38 | 0.00 | 1.40 | 1.47 | 1.78 | 1.62 | 1.64 | 2.34 | 2.103 |
| C-Mono1\|γδ_T | 0.00 | 0.11 | 0.84 | 0.94 | 1.36 | 1.04 | 1.04 | 1.27 | 1.26 | 1.25 | 0.00 | 1.41 | 1.35 | 1.44 | 1.47 | 1.76 | 1.65 | 1.62 | 2.42 | 1.976 |
| C-Mono2\|C-Mono1 | 0.00 | 0.44 | 0.82 | 0.82 | 0.93 | 0.93 | 0.00 | 1.01 | 1.01 | 1.15 | 1.12 | 1.29 | 0.00 | 1.38 | 1.14 | 1.19 | 1.55 | 1.36 | 2.08 | 2.382 |
| C-Mono2\|C-Mono2 | 1.33 | 0.40 | 0.77 | 0.80 | 0.93 | 0.93 | 0.93 | 0.93 | 0.93 | 0.95 | 1.12 | 1.29 | 1.24 | 1.35 | 1.13 | 1.12 | 1.55 | 1.29 | 1.90 | 2.37 |
| C-Mono2\|C-Mono3 | 0.00 | 0.52 | 0.77 | 0.80 | 0.93 | 0.93 | 0.93 | 1.03 | 1.03 | 1.02 | 1.12 | 1.30 | 1.24 | 1.36 | 1.12 | 1.13 | 1.55 | 1.31 | 1.94 | 2.372 |
| C-Mono2\|CD4+_effector_T_cell | 1.33 | 0.08 | 0.69 | 0.79 | 0.93 | 0.93 | 0.93 | 1.31 | 1.31 | 1.26 | 0.00 | 1.38 | 1.24 | 1.38 | 0.98 | 1.32 | 1.55 | 1.15 | 2.42 | 1.977 |
| C-Mono2\|CD4+_memory_T_cell | 1.33 | 0.09 | 0.69 | 0.75 | 0.93 | 0.93 | 0.93 | 1.23 | 1.23 | 1.17 | 1.12 | 1.41 | 1.24 | 1.37 | 1.01 | 1.32 | 1.55 | 1.15 | 2.45 | 2.076 |
| C-Mono2\|CD4+_naÃ¯ve_T_cell | 1.33 | 0.08 | 0.69 | 0.75 | 0.93 | 0.93 | 0.93 | 1.09 | 1.09 | 1.09 | 1.12 | 1.37 | 1.24 | 1.36 | 1.04 | 1.33 | 1.55 | 1.10 | 2.55 | 1.964 |
| C-Mono2\|CD56brightCD16-_NK | 1.34 | 0.19 | 0.71 | 0.85 | 1.65 | 1.20 | 1.00 | 1.27 | 1.27 | 1.21 | 0.00 | 1.36 | 1.25 | 1.38 | 0.98 | 1.35 | 1.59 | 1.21 | 2.37 | 2.125 |
| C-Mono2\|CD56dimCD16+_NK | 1.37 | 0.09 | 0.71 | 0.86 | 1.10 | 1.08 | 0.98 | 1.42 | 1.42 | 1.23 | 1.12 | 1.39 | 1.30 | 1.42 | 0.98 | 1.26 | 1.66 | 1.13 | 2.16 | 1.735 |
| C-Mono2\|CD8+_naÃ¯ve_T_cell | 1.34 | 0.09 | 0.69 | 0.78 | 0.99 | 0.99 | 0.95 | 1.26 | 1.26 | 1.17 | 1.12 | 1.43 | 1.26 | 1.38 | 1.03 | 1.37 | 1.56 | 1.15 | 2.49 | 1.941 |
| C-Mono2\|GNLY+CD8+_cytotoxic_T_cell | 1.33 | 0.10 | 0.69 | 0.82 | 0.95 | 1.01 | 0.97 | 1.32 | 1.32 | 1.21 | 1.12 | 1.37 | 1.26 | 1.38 | 0.98 | 1.28 | 1.59 | 1.14 | 2.37 | 1.885 |
| C-Mono2\|GZMK+CD8+_cytotoxic_T_cell | 1.33 | 0.11 | 0.69 | 0.80 | 0.96 | 0.95 | 0.96 | 1.25 | 1.25 | 1.17 | 1.12 | 1.39 | 1.24 | 1.38 | 0.98 | 1.30 | 1.55 | 1.14 | 2.45 | 1.994 |
| C-Mono2\|MAI_T | 0.00 | 0.16 | 0.69 | 0.78 | 0.99 | 0.93 | 0.94 | 1.27 | 1.27 | 1.16 | 0.00 | 1.35 | 0.00 | 1.42 | 0.98 | 1.29 | 0.00 | 1.17 | 2.44 | 1.957 |
| C-Mono2\|Memory_B_cell | 0.00 | 0.09 | 0.76 | 0.75 | 0.00 | 0.00 | 0.93 | 1.18 | 1.18 | 1.25 | 1.50 | 1.21 | 0.00 | 1.47 | 1.02 | 1.30 | 1.55 | 1.15 | 2.45 | 1.965 |
| C-Mono2\|NC-Mono | 1.33 | 0.34 | 0.91 | 0.83 | 0.93 | 0.00 | 0.00 | 1.00 | 1.00 | 1.08 | 1.13 | 1.26 | 0.00 | 1.38 | 1.08 | 1.17 | 1.55 | 1.28 | 2.18 | 2.185 |
| C-Mono2\|NKT | 1.34 | 0.09 | 0.70 | 0.86 | 1.07 | 1.08 | 0.98 | 1.39 | 1.39 | 1.28 | 0.00 | 1.47 | 1.28 | 1.42 | 0.98 | 1.32 | 1.63 | 1.15 | 2.31 | 1.873 |
| C-Mono2\|NaÃ¯ve_B_cell | 0.00 | 0.08 | 0.73 | 0.75 | 0.93 | 0.93 | 0.93 | 1.07 | 1.07 | 1.16 | 1.65 | 1.20 | 1.24 | 1.52 | 1.06 | 1.20 | 1.55 | 1.11 | 2.53 | 1.806 |
| C-Mono2\|Plasma | 0.00 | 0.08 | 0.00 | 0.75 | 0.94 | 0.00 | 0.00 | 0.67 | 0.67 | 1.14 | 1.14 | 1.22 | 0.00 | 1.42 | 0.98 | 1.46 | 0.00 | 1.18 | 2.08 | 1.951 |
| C-Mono2\|Treg | 0.00 | 0.08 | 0.69 | 0.75 | 0.00 | 0.00 | 0.93 | 1.26 | 1.26 | 1.25 | 1.12 | 1.37 | 0.00 | 1.35 | 0.99 | 1.30 | 1.55 | 1.15 | 2.38 | 2.088 |
| C-Mono2\|γδ_T | 0.00 | 0.11 | 0.70 | 0.81 | 1.28 | 0.96 | 0.95 | 1.27 | 1.27 | 1.21 | 0.00 | 1.40 | 1.25 | 1.39 | 0.99 | 1.28 | 1.58 | 1.14 | 2.46 | 1.961 |
| C-Mono3\|C-Mono1 | 0.00 | 0.45 | 0.83 | 0.91 | 1.03 | 1.03 | 0.00 | 1.01 | 1.01 | 1.20 | 1.13 | 1.30 | 0.00 | 1.44 | 1.25 | 1.30 | 1.60 | 1.46 | 2.09 | 2.425 |
| C-Mono3\|C-Mono2 | 1.40 | 0.41 | 0.78 | 0.89 | 1.03 | 1.03 | 1.03 | 0.93 | 0.93 | 1.00 | 1.13 | 1.29 | 1.36 | 1.42 | 1.23 | 1.23 | 1.60 | 1.39 | 1.92 | 2.413 |
| C-Mono3\|C-Mono3 | 0.00 | 0.53 | 0.78 | 0.90 | 1.03 | 1.03 | 1.03 | 1.03 | 1.03 | 1.07 | 1.13 | 1.31 | 1.36 | 1.42 | 1.22 | 1.23 | 1.60 | 1.41 | 1.95 | 2.414 |
| C-Mono3\|CD4+_effector_T_cell | 1.40 | 0.09 | 0.70 | 0.88 | 1.03 | 1.03 | 1.03 | 1.32 | 1.31 | 1.31 | 0.00 | 1.39 | 1.36 | 1.45 | 1.08 | 1.42 | 1.60 | 1.26 | 2.43 | 2.02 |
| C-Mono3\|CD4+_memory_T_cell | 1.40 | 0.09 | 0.71 | 0.84 | 1.03 | 1.03 | 1.03 | 1.23 | 1.23 | 1.22 | 1.13 | 1.41 | 1.36 | 1.44 | 1.11 | 1.42 | 1.61 | 1.25 | 2.47 | 2.119 |
| C-Mono3\|CD4+_naÃ¯ve_T_cell | 1.40 | 0.09 | 0.70 | 0.84 | 1.03 | 1.03 | 1.03 | 1.09 | 1.09 | 1.14 | 1.13 | 1.38 | 1.36 | 1.43 | 1.14 | 1.43 | 1.60 | 1.20 | 2.57 | 2.007 |
| C-Mono3\|CD56brightCD16-_NK | 1.41 | 0.20 | 0.72 | 0.94 | 1.75 | 1.30 | 1.10 | 1.27 | 1.27 | 1.26 | 0.00 | 1.37 | 1.37 | 1.45 | 1.08 | 1.45 | 1.65 | 1.31 | 2.38 | 2.167 |
| C-Mono3\|CD56dimCD16+_NK | 1.43 | 0.10 | 0.72 | 0.96 | 1.20 | 1.18 | 1.07 | 1.43 | 1.42 | 1.28 | 1.13 | 1.39 | 1.41 | 1.49 | 1.08 | 1.36 | 1.71 | 1.24 | 2.18 | 1.777 |
| C-Mono3\|CD8+_naÃ¯ve_T_cell | 1.41 | 0.10 | 0.70 | 0.88 | 1.09 | 1.09 | 1.05 | 1.26 | 1.26 | 1.22 | 1.13 | 1.44 | 1.37 | 1.45 | 1.13 | 1.47 | 1.62 | 1.25 | 2.51 | 1.984 |
| C-Mono3\|GNLY+CD8+_cytotoxic_T_cell | 1.40 | 0.10 | 0.71 | 0.92 | 1.05 | 1.11 | 1.07 | 1.32 | 1.32 | 1.26 | 1.13 | 1.37 | 1.37 | 1.45 | 1.08 | 1.38 | 1.64 | 1.24 | 2.38 | 1.928 |
| C-Mono3\|GZMK+CD8+_cytotoxic_T_cell | 1.40 | 0.11 | 0.70 | 0.89 | 1.06 | 1.05 | 1.06 | 1.25 | 1.25 | 1.22 | 1.13 | 1.40 | 1.36 | 1.44 | 1.08 | 1.41 | 1.61 | 1.24 | 2.46 | 2.037 |
| C-Mono3\|MAI_T | 0.00 | 0.17 | 0.71 | 0.87 | 1.09 | 1.03 | 1.04 | 1.28 | 1.28 | 1.21 | 0.00 | 1.35 | 0.00 | 1.49 | 1.08 | 1.39 | 0.00 | 1.27 | 2.46 | 1.999 |
| C-Mono3\|Memory_B_cell | 0.00 | 0.10 | 0.77 | 0.84 | 0.00 | 0.00 | 1.03 | 1.18 | 1.18 | 1.30 | 1.51 | 1.22 | 0.00 | 1.54 | 1.12 | 1.40 | 1.60 | 1.25 | 2.47 | 2.008 |
| C-Mono3\|NC-Mono | 1.40 | 0.35 | 0.92 | 0.93 | 1.03 | 0.00 | 0.00 | 1.00 | 1.00 | 1.13 | 1.14 | 1.27 | 0.00 | 1.44 | 1.18 | 1.28 | 1.60 | 1.39 | 2.20 | 2.228 |
| C-Mono3\|NKT | 1.41 | 0.10 | 0.71 | 0.96 | 1.17 | 1.18 | 1.08 | 1.39 | 1.39 | 1.33 | 0.00 | 1.47 | 1.40 | 1.49 | 1.08 | 1.43 | 1.68 | 1.25 | 2.33 | 1.916 |
| C-Mono3\|NaÃ¯ve_B_cell | 0.00 | 0.09 | 0.74 | 0.84 | 1.03 | 1.03 | 1.03 | 1.07 | 1.07 | 1.21 | 1.66 | 1.20 | 1.36 | 1.58 | 1.17 | 1.30 | 1.60 | 1.21 | 2.55 | 1.849 |
| C-Mono3\|Plasma | 0.00 | 0.09 | 0.00 | 0.85 | 1.04 | 0.00 | 0.00 | 0.67 | 0.67 | 1.19 | 1.15 | 1.22 | 0.00 | 1.49 | 1.08 | 1.57 | 0.00 | 1.28 | 2.10 | 1.994 |
| C-Mono3\|Treg | 0.00 | 0.09 | 0.70 | 0.84 | 0.00 | 0.00 | 1.03 | 1.26 | 1.26 | 1.30 | 1.13 | 1.38 | 0.00 | 1.42 | 1.09 | 1.40 | 1.60 | 1.26 | 2.39 | 2.131 |
| C-Mono3\|γδ_T | 0.00 | 0.12 | 0.71 | 0.90 | 1.38 | 1.06 | 1.05 | 1.27 | 1.27 | 1.25 | 0.00 | 1.41 | 1.37 | 1.46 | 1.09 | 1.38 | 1.63 | 1.25 | 2.48 | 2.004 |
| CD4+_effector_T_cell\|C-Mono1 | 0.00 | 1.98 | 1.61 | 0.96 | 1.31 | 1.31 | 0.00 | 1.01 | 1.01 | 1.10 | 1.29 | 1.46 | 0.00 | 1.78 | 0.70 | 0.75 | 1.77 | 0.91 | 1.62 | 0.947 |
| CD4+_effector_T_cell\|C-Mono2 | 1.73 | 1.93 | 1.56 | 0.94 | 1.31 | 1.31 | 1.31 | 0.93 | 0.93 | 0.90 | 1.29 | 1.46 | 1.62 | 1.75 | 0.68 | 0.68 | 1.77 | 0.84 | 1.44 | 0.935 |
| CD4+_effector_T_cell\|C-Mono3 | 0.00 | 2.06 | 1.57 | 0.94 | 1.32 | 1.31 | 1.31 | 1.03 | 1.03 | 0.97 | 1.29 | 1.47 | 1.62 | 1.76 | 0.68 | 0.68 | 1.77 | 0.86 | 1.48 | 0.936 |
| CD4+_effector_T_cell\|CD4+_effector_T_cell | 1.73 | 1.62 | 1.49 | 0.93 | 1.31 | 1.32 | 1.31 | 1.31 | 1.32 | 1.21 | 0.00 | 1.55 | 1.62 | 1.79 | 0.54 | 0.87 | 1.78 | 0.71 | 1.96 | 0.542 |
| CD4+_effector_T_cell\|CD4+_memory_T_cell | 1.73 | 1.62 | 1.49 | 0.89 | 1.32 | 1.31 | 1.32 | 1.23 | 1.23 | 1.12 | 1.29 | 1.58 | 1.62 | 1.77 | 0.56 | 0.87 | 1.78 | 0.70 | 1.99 | 0.64 |
| CD4+_effector_T_cell\|CD4+_naÃ¯ve_T_cell | 1.73 | 1.62 | 1.49 | 0.89 | 1.31 | 1.31 | 1.31 | 1.09 | 1.09 | 1.04 | 1.29 | 1.54 | 1.62 | 1.76 | 0.59 | 0.88 | 1.77 | 0.65 | 2.09 | 0.528 |
| CD4+_effector_T_cell\|CD56brightCD16-_NK | 1.75 | 1.72 | 1.51 | 0.99 | 2.03 | 1.58 | 1.38 | 1.27 | 1.27 | 1.16 | 0.00 | 1.53 | 1.63 | 1.78 | 0.53 | 0.90 | 1.82 | 0.76 | 1.91 | 0.689 |
| CD4+_effector_T_cell\|CD56dimCD16+_NK | 1.77 | 1.63 | 1.50 | 1.01 | 1.49 | 1.46 | 1.36 | 1.42 | 1.43 | 1.18 | 1.29 | 1.55 | 1.67 | 1.82 | 0.53 | 0.82 | 1.88 | 0.69 | 1.70 | 0.299 |
| CD4+_effector_T_cell\|CD8+_naÃ¯ve_T_cell | 1.74 | 1.62 | 1.49 | 0.93 | 1.37 | 1.38 | 1.33 | 1.26 | 1.26 | 1.12 | 1.29 | 1.60 | 1.63 | 1.79 | 0.58 | 0.92 | 1.79 | 0.70 | 2.03 | 0.506 |
| CD4+_effector_T_cell\|GNLY+CD8+_cytotoxic_T_cell | 1.74 | 1.63 | 1.49 | 0.97 | 1.33 | 1.40 | 1.35 | 1.32 | 1.32 | 1.16 | 1.29 | 1.54 | 1.63 | 1.78 | 0.53 | 0.83 | 1.81 | 0.69 | 1.91 | 0.45 |
| CD4+_effector_T_cell\|GZMK+CD8+_cytotoxic_T_cell | 1.73 | 1.64 | 1.49 | 0.94 | 1.35 | 1.33 | 1.34 | 1.25 | 1.25 | 1.12 | 1.29 | 1.56 | 1.62 | 1.78 | 0.54 | 0.86 | 1.78 | 0.69 | 1.99 | 0.559 |
| CD4+_effector_T_cell\|MAI_T | 0.00 | 1.70 | 1.49 | 0.92 | 1.37 | 1.32 | 1.32 | 1.27 | 1.28 | 1.11 | 0.00 | 1.51 | 0.00 | 1.82 | 0.53 | 0.84 | 0.00 | 0.72 | 1.98 | 0.521 |
| CD4+_effector_T_cell\|Memory_B_cell | 0.00 | 1.63 | 1.56 | 0.89 | 0.00 | 0.00 | 1.31 | 1.18 | 1.18 | 1.20 | 1.67 | 1.38 | 0.00 | 1.87 | 0.58 | 0.85 | 1.78 | 0.70 | 1.99 | 0.53 |
| CD4+_effector_T_cell\|NC-Mono | 1.73 | 1.88 | 1.70 | 0.97 | 1.32 | 0.00 | 0.00 | 1.00 | 1.00 | 1.03 | 1.30 | 1.43 | 0.00 | 1.78 | 0.64 | 0.73 | 1.77 | 0.84 | 1.72 | 0.749 |
| CD4+_effector_T_cell\|NKT | 1.74 | 1.62 | 1.50 | 1.01 | 1.46 | 1.46 | 1.36 | 1.39 | 1.39 | 1.23 | 0.00 | 1.63 | 1.66 | 1.82 | 0.53 | 0.88 | 1.85 | 0.71 | 1.85 | 0.438 |
| CD4+_effector_T_cell\|NaÃ¯ve_B_cell | 0.00 | 1.62 | 1.52 | 0.89 | 1.31 | 1.31 | 1.31 | 1.07 | 1.07 | 1.11 | 1.82 | 1.37 | 1.62 | 1.92 | 0.62 | 0.76 | 1.77 | 0.66 | 2.07 | 0.371 |
| CD4+_effector_T_cell\|Plasma | 0.00 | 1.62 | 0.00 | 0.90 | 1.33 | 0.00 | 0.00 | 0.67 | 0.67 | 1.09 | 1.31 | 1.38 | 0.00 | 1.82 | 0.53 | 1.02 | 0.00 | 0.73 | 1.62 | 0.516 |
| CD4+_effector_T_cell\|Treg | 0.00 | 1.62 | 1.49 | 0.89 | 0.00 | 0.00 | 1.31 | 1.26 | 1.26 | 1.20 | 1.29 | 1.54 | 0.00 | 1.75 | 0.55 | 0.85 | 1.77 | 0.71 | 1.92 | 0.653 |
| CD4+_effector_T_cell\|γδ_T | 0.00 | 1.64 | 1.49 | 0.95 | 1.66 | 1.34 | 1.34 | 1.27 | 1.27 | 1.15 | 0.00 | 1.57 | 1.63 | 1.79 | 0.54 | 0.83 | 1.81 | 0.70 | 2.00 | 0.525 |
| CD4+_memory_T_cell\|C-Mono1 | 0.00 | 1.10 | 1.53 | 0.88 | 1.23 | 1.23 | 0.00 | 1.01 | 1.01 | 1.07 | 1.18 | 1.35 | 0.00 | 1.62 | 0.66 | 0.71 | 1.62 | 0.87 | 1.62 | 0.947 |
| CD4+_memory_T_cell\|C-Mono2 | 1.57 | 1.06 | 1.48 | 0.86 | 1.23 | 1.23 | 1.23 | 0.93 | 0.93 | 0.86 | 1.18 | 1.34 | 1.53 | 1.59 | 0.64 | 0.64 | 1.62 | 0.81 | 1.44 | 0.934 |
| CD4+_memory_T_cell\|C-Mono3 | 0.00 | 1.18 | 1.48 | 0.86 | 1.23 | 1.23 | 1.23 | 1.03 | 1.03 | 0.93 | 1.18 | 1.36 | 1.53 | 1.60 | 0.64 | 0.64 | 1.62 | 0.82 | 1.48 | 0.936 |
| CD4+_memory_T_cell\|CD4+_effector_T_cell | 1.57 | 0.74 | 1.40 | 0.85 | 1.23 | 1.23 | 1.23 | 1.32 | 1.31 | 1.17 | 0.00 | 1.43 | 1.53 | 1.62 | 0.50 | 0.84 | 1.63 | 0.67 | 1.96 | 0.542 |
| CD4+_memory_T_cell\|CD4+_memory_T_cell | 1.57 | 0.75 | 1.40 | 0.81 | 1.23 | 1.23 | 1.23 | 1.23 | 1.23 | 1.08 | 1.17 | 1.46 | 1.53 | 1.61 | 0.52 | 0.84 | 1.63 | 0.66 | 1.99 | 0.64 |
| CD4+_memory_T_cell\|CD4+_naÃ¯ve_T_cell | 1.57 | 0.74 | 1.40 | 0.81 | 1.23 | 1.23 | 1.23 | 1.09 | 1.09 | 1.00 | 1.17 | 1.42 | 1.53 | 1.60 | 0.55 | 0.84 | 1.62 | 0.62 | 2.09 | 0.528 |
| CD4+_memory_T_cell\|CD56brightCD16-_NK | 1.59 | 0.85 | 1.42 | 0.91 | 1.94 | 1.49 | 1.30 | 1.27 | 1.27 | 1.12 | 0.00 | 1.42 | 1.54 | 1.62 | 0.49 | 0.86 | 1.67 | 0.73 | 1.91 | 0.689 |
| CD4+_memory_T_cell\|CD56dimCD16+_NK | 1.61 | 0.75 | 1.42 | 0.92 | 1.40 | 1.37 | 1.27 | 1.43 | 1.42 | 1.14 | 1.17 | 1.44 | 1.58 | 1.66 | 0.49 | 0.78 | 1.74 | 0.65 | 1.70 | 0.299 |
| CD4+_memory_T_cell\|CD8+_naÃ¯ve_T_cell | 1.58 | 0.75 | 1.40 | 0.84 | 1.29 | 1.29 | 1.25 | 1.26 | 1.26 | 1.08 | 1.17 | 1.49 | 1.54 | 1.63 | 0.54 | 0.88 | 1.64 | 0.66 | 2.03 | 0.505 |
| CD4+_memory_T_cell\|GNLY+CD8+_cytotoxic_T_cell | 1.58 | 0.75 | 1.40 | 0.88 | 1.25 | 1.31 | 1.27 | 1.32 | 1.32 | 1.13 | 1.17 | 1.42 | 1.54 | 1.62 | 0.49 | 0.79 | 1.66 | 0.66 | 1.91 | 0.449 |
| CD4+_memory_T_cell\|GZMK+CD8+_cytotoxic_T_cell | 1.57 | 0.76 | 1.40 | 0.85 | 1.26 | 1.24 | 1.26 | 1.25 | 1.25 | 1.08 | 1.17 | 1.45 | 1.53 | 1.62 | 0.50 | 0.82 | 1.63 | 0.66 | 1.99 | 0.558 |
| CD4+_memory_T_cell\|MAI_T | 0.00 | 0.82 | 1.40 | 0.83 | 1.28 | 1.23 | 1.24 | 1.28 | 1.27 | 1.07 | 0.00 | 1.40 | 0.00 | 1.66 | 0.49 | 0.80 | 0.00 | 0.69 | 1.98 | 0.521 |
| CD4+_memory_T_cell\|Memory_B_cell | 0.00 | 0.75 | 1.47 | 0.80 | 0.00 | 0.00 | 1.23 | 1.18 | 1.18 | 1.16 | 1.56 | 1.26 | 0.00 | 1.71 | 0.54 | 0.82 | 1.63 | 0.66 | 1.99 | 0.529 |
| CD4+_memory_T_cell\|NC-Mono | 1.57 | 1.00 | 1.62 | 0.89 | 1.23 | 0.00 | 0.00 | 1.00 | 1.00 | 0.99 | 1.18 | 1.32 | 0.00 | 1.62 | 0.60 | 0.69 | 1.62 | 0.80 | 1.72 | 0.749 |
| CD4+_memory_T_cell\|NKT | 1.58 | 0.75 | 1.41 | 0.92 | 1.37 | 1.37 | 1.28 | 1.39 | 1.39 | 1.19 | 0.00 | 1.52 | 1.57 | 1.66 | 0.50 | 0.84 | 1.70 | 0.67 | 1.85 | 0.438 |
| CD4+_memory_T_cell\|NaÃ¯ve_B_cell | 0.00 | 0.74 | 1.44 | 0.81 | 1.23 | 1.23 | 1.23 | 1.08 | 1.07 | 1.07 | 1.70 | 1.25 | 1.53 | 1.76 | 0.58 | 0.72 | 1.62 | 0.62 | 2.07 | 0.371 |
| CD4+_memory_T_cell\|Plasma | 0.00 | 0.74 | 0.00 | 0.81 | 1.24 | 0.00 | 0.00 | 0.68 | 0.67 | 1.06 | 1.19 | 1.27 | 0.00 | 1.66 | 0.49 | 0.98 | 0.00 | 0.69 | 1.62 | 0.516 |
| CD4+_memory_T_cell\|Treg | 0.00 | 0.74 | 1.40 | 0.81 | 0.00 | 0.00 | 1.23 | 1.26 | 1.26 | 1.16 | 1.17 | 1.42 | 0.00 | 1.59 | 0.51 | 0.81 | 1.62 | 0.67 | 1.92 | 0.652 |
| CD4+_memory_T_cell\|γδ_T | 0.00 | 0.77 | 1.41 | 0.87 | 1.57 | 1.26 | 1.25 | 1.27 | 1.26 | 1.12 | 0.00 | 1.45 | 1.54 | 1.63 | 0.51 | 0.79 | 1.66 | 0.66 | 2.00 | 0.525 |
| CD4+_naÃ¯ve_T_cell\|C-Mono1 | 0.00 | 0.44 | 1.24 | 1.01 | 1.09 | 1.09 | 0.00 | 1.01 | 1.01 | 1.01 | 1.03 | 1.20 | 0.00 | 1.44 | 0.38 | 0.43 | 1.47 | 0.59 | 1.62 | 0.945 |
| CD4+_naÃ¯ve_T_cell\|C-Mono2 | 1.40 | 0.40 | 1.19 | 0.99 | 1.09 | 1.09 | 1.09 | 0.93 | 0.93 | 0.81 | 1.03 | 1.19 | 1.30 | 1.42 | 0.36 | 0.36 | 1.47 | 0.52 | 1.44 | 0.932 |
| CD4+_naÃ¯ve_T_cell\|C-Mono3 | 0.00 | 0.52 | 1.20 | 1.00 | 1.09 | 1.09 | 1.09 | 1.03 | 1.03 | 0.88 | 1.03 | 1.20 | 1.31 | 1.42 | 0.35 | 0.36 | 1.47 | 0.54 | 1.47 | 0.934 |
| CD4+_naÃ¯ve_T_cell\|CD4+_effector_T_cell | 1.40 | 0.09 | 1.12 | 0.98 | 1.09 | 1.09 | 1.09 | 1.31 | 1.31 | 1.12 | 0.00 | 1.28 | 1.31 | 1.45 | 0.21 | 0.55 | 1.47 | 0.39 | 1.95 | 0.539 |
| CD4+_naÃ¯ve_T_cell\|CD4+_memory_T_cell | 1.40 | 0.09 | 1.12 | 0.94 | 1.09 | 1.09 | 1.09 | 1.23 | 1.23 | 1.03 | 1.02 | 1.31 | 1.31 | 1.44 | 0.24 | 0.55 | 1.48 | 0.38 | 1.99 | 0.638 |
| CD4+_naÃ¯ve_T_cell\|CD4+_naÃ¯ve_T_cell | 1.40 | 0.08 | 1.12 | 0.94 | 1.09 | 1.09 | 1.09 | 1.09 | 1.09 | 0.95 | 1.02 | 1.27 | 1.30 | 1.43 | 0.27 | 0.56 | 1.47 | 0.33 | 2.09 | 0.526 |
| CD4+_naÃ¯ve_T_cell\|CD56brightCD16-_NK | 1.41 | 0.19 | 1.14 | 1.04 | 1.81 | 1.36 | 1.16 | 1.27 | 1.27 | 1.07 | 0.00 | 1.27 | 1.31 | 1.45 | 0.21 | 0.58 | 1.52 | 0.44 | 1.91 | 0.687 |
| CD4+_naÃ¯ve_T_cell\|CD56dimCD16+_NK | 1.43 | 0.09 | 1.13 | 1.06 | 1.26 | 1.24 | 1.13 | 1.42 | 1.42 | 1.09 | 1.02 | 1.29 | 1.36 | 1.49 | 0.21 | 0.49 | 1.58 | 0.37 | 1.70 | 0.297 |
| CD4+_naÃ¯ve_T_cell\|CD8+_naÃ¯ve_T_cell | 1.41 | 0.09 | 1.12 | 0.98 | 1.15 | 1.15 | 1.11 | 1.26 | 1.26 | 1.03 | 1.02 | 1.34 | 1.32 | 1.45 | 0.26 | 0.60 | 1.49 | 0.38 | 2.03 | 0.503 |
| CD4+_naÃ¯ve_T_cell\|GNLY+CD8+_cytotoxic_T_cell | 1.40 | 0.10 | 1.12 | 1.02 | 1.11 | 1.17 | 1.13 | 1.32 | 1.32 | 1.07 | 1.02 | 1.27 | 1.32 | 1.45 | 0.21 | 0.51 | 1.51 | 0.37 | 1.90 | 0.447 |
| CD4+_naÃ¯ve_T_cell\|GZMK+CD8+_cytotoxic_T_cell | 1.40 | 0.11 | 1.12 | 0.99 | 1.12 | 1.11 | 1.12 | 1.25 | 1.25 | 1.03 | 1.02 | 1.29 | 1.30 | 1.44 | 0.21 | 0.54 | 1.47 | 0.37 | 1.98 | 0.556 |
| CD4+_naÃ¯ve_T_cell\|MAI_T | 0.00 | 0.17 | 1.12 | 0.97 | 1.15 | 1.09 | 1.10 | 1.27 | 1.27 | 1.02 | 0.00 | 1.25 | 0.00 | 1.49 | 0.21 | 0.52 | 0.00 | 0.40 | 1.98 | 0.519 |
| CD4+_naÃ¯ve_T_cell\|Memory_B_cell | 0.00 | 0.09 | 1.18 | 0.94 | 0.00 | 0.00 | 1.09 | 1.18 | 1.18 | 1.11 | 1.40 | 1.11 | 0.00 | 1.54 | 0.25 | 0.53 | 1.47 | 0.38 | 1.99 | 0.527 |
| CD4+_naÃ¯ve_T_cell\|NC-Mono | 1.40 | 0.34 | 1.33 | 1.03 | 1.09 | 0.00 | 0.00 | 1.00 | 1.00 | 0.94 | 1.03 | 1.17 | 0.00 | 1.44 | 0.31 | 0.41 | 1.47 | 0.52 | 1.72 | 0.747 |
| CD4+_naÃ¯ve_T_cell\|NKT | 1.41 | 0.09 | 1.13 | 1.06 | 1.23 | 1.24 | 1.14 | 1.39 | 1.39 | 1.13 | 0.00 | 1.37 | 1.34 | 1.49 | 0.21 | 0.56 | 1.55 | 0.39 | 1.85 | 0.435 |
| CD4+_naÃ¯ve_T_cell\|NaÃ¯ve_B_cell | 0.00 | 0.08 | 1.15 | 0.94 | 1.09 | 1.09 | 1.09 | 1.07 | 1.07 | 1.02 | 1.55 | 1.10 | 1.31 | 1.58 | 0.30 | 0.43 | 1.47 | 0.34 | 2.07 | 0.368 |
| CD4+_naÃ¯ve_T_cell\|Plasma | 0.00 | 0.08 | 0.00 | 0.95 | 1.10 | 0.00 | 0.00 | 0.67 | 0.67 | 1.00 | 1.04 | 1.12 | 0.00 | 1.49 | 0.21 | 0.70 | 0.00 | 0.41 | 1.62 | 0.513 |
| CD4+_naÃ¯ve_T_cell\|Treg | 0.00 | 0.08 | 1.12 | 0.95 | 0.00 | 0.00 | 1.09 | 1.26 | 1.26 | 1.11 | 1.02 | 1.27 | 0.00 | 1.42 | 0.23 | 0.53 | 1.47 | 0.39 | 1.91 | 0.65 |
| CD4+_naÃ¯ve_T_cell\|γδ_T | 0.00 | 0.11 | 1.12 | 1.00 | 1.44 | 1.12 | 1.11 | 1.27 | 1.26 | 1.06 | 0.00 | 1.30 | 1.31 | 1.46 | 0.22 | 0.51 | 1.50 | 0.38 | 2.00 | 0.523 |
| CD56brightCD16-_NK\|C-Mono1 | 0.00 | 1.48 | 0.94 | 0.69 | 1.27 | 1.27 | 0.00 | 1.73 | 1.28 | 1.14 | 1.17 | 1.34 | 0.00 | 1.69 | 0.74 | 0.79 | 1.66 | 0.95 | 1.62 | 1.964 |
| CD56brightCD16-_NK\|C-Mono2 | 1.64 | 1.44 | 0.89 | 0.67 | 1.27 | 1.27 | 1.27 | 1.65 | 1.20 | 0.94 | 1.16 | 1.33 | 1.60 | 1.67 | 0.73 | 0.72 | 1.66 | 0.89 | 1.45 | 1.952 |
| CD56brightCD16-_NK\|C-Mono3 | 0.00 | 1.56 | 0.89 | 0.68 | 1.27 | 1.27 | 1.27 | 1.75 | 1.30 | 1.00 | 1.17 | 1.34 | 1.60 | 1.67 | 0.72 | 0.72 | 1.66 | 0.91 | 1.48 | 1.954 |
| CD56brightCD16-_NK\|CD4+_effector_T_cell | 1.64 | 1.12 | 0.82 | 0.66 | 1.27 | 1.27 | 1.27 | 2.03 | 1.58 | 1.24 | 0.00 | 1.42 | 1.60 | 1.70 | 0.58 | 0.92 | 1.66 | 0.75 | 1.96 | 1.559 |
| CD56brightCD16-_NK\|CD4+_memory_T_cell | 1.64 | 1.13 | 0.82 | 0.62 | 1.27 | 1.27 | 1.27 | 1.94 | 1.49 | 1.16 | 1.16 | 1.45 | 1.60 | 1.69 | 0.60 | 0.92 | 1.67 | 0.74 | 2.00 | 1.658 |
| CD56brightCD16-_NK\|CD4+_naÃ¯ve_T_cell | 1.64 | 1.12 | 0.81 | 0.62 | 1.27 | 1.27 | 1.27 | 1.81 | 1.36 | 1.08 | 1.16 | 1.41 | 1.60 | 1.67 | 0.63 | 0.92 | 1.66 | 0.70 | 2.10 | 1.546 |
| CD56brightCD16-_NK\|CD56brightCD16-_NK | 1.66 | 1.23 | 0.83 | 0.72 | 1.98 | 1.53 | 1.34 | 1.98 | 1.53 | 1.20 | 0.00 | 1.40 | 1.61 | 1.70 | 0.57 | 0.95 | 1.71 | 0.81 | 1.91 | 1.707 |
| CD56brightCD16-_NK\|CD56dimCD16+_NK | 1.68 | 1.13 | 0.83 | 0.74 | 1.44 | 1.41 | 1.31 | 2.14 | 1.69 | 1.22 | 1.16 | 1.43 | 1.66 | 1.73 | 0.57 | 0.86 | 1.77 | 0.73 | 1.71 | 1.317 |
| CD56brightCD16-_NK\|CD8+_naÃ¯ve_T_cell | 1.65 | 1.13 | 0.81 | 0.66 | 1.33 | 1.33 | 1.29 | 1.98 | 1.53 | 1.15 | 1.16 | 1.48 | 1.61 | 1.70 | 0.62 | 0.97 | 1.68 | 0.74 | 2.04 | 1.523 |
| CD56brightCD16-_NK\|GNLY+CD8+_cytotoxic_T_cell | 1.65 | 1.14 | 0.82 | 0.70 | 1.28 | 1.35 | 1.31 | 2.03 | 1.58 | 1.20 | 1.16 | 1.41 | 1.62 | 1.69 | 0.58 | 0.87 | 1.70 | 0.74 | 1.91 | 1.467 |
| CD56brightCD16-_NK\|GZMK+CD8+_cytotoxic_T_cell | 1.64 | 1.14 | 0.81 | 0.67 | 1.30 | 1.28 | 1.29 | 1.96 | 1.51 | 1.15 | 1.16 | 1.43 | 1.60 | 1.69 | 0.58 | 0.90 | 1.67 | 0.74 | 1.99 | 1.576 |
| CD56brightCD16-_NK\|MAI_T | 0.00 | 1.20 | 0.82 | 0.65 | 1.32 | 1.27 | 1.28 | 1.99 | 1.54 | 1.15 | 0.00 | 1.39 | 0.00 | 1.73 | 0.57 | 0.88 | 0.00 | 0.77 | 1.99 | 1.539 |
| CD56brightCD16-_NK\|Memory_B_cell | 0.00 | 1.13 | 0.88 | 0.62 | 0.00 | 0.00 | 1.27 | 1.90 | 1.45 | 1.23 | 1.54 | 1.25 | 0.00 | 1.78 | 0.62 | 0.90 | 1.66 | 0.75 | 2.00 | 1.547 |
| CD56brightCD16-_NK\|NC-Mono | 1.64 | 1.38 | 1.03 | 0.71 | 1.27 | 0.00 | 0.00 | 1.72 | 1.27 | 1.06 | 1.17 | 1.31 | 0.00 | 1.69 | 0.68 | 0.77 | 1.66 | 0.88 | 1.73 | 1.767 |
| CD56brightCD16-_NK\|NKT | 1.66 | 1.13 | 0.82 | 0.74 | 1.41 | 1.41 | 1.32 | 2.10 | 1.65 | 1.26 | 0.00 | 1.51 | 1.64 | 1.73 | 0.58 | 0.92 | 1.74 | 0.75 | 1.86 | 1.455 |
| CD56brightCD16-_NK\|NaÃ¯ve_B_cell | 0.00 | 1.12 | 0.85 | 0.62 | 1.27 | 1.27 | 1.27 | 1.79 | 1.34 | 1.15 | 1.69 | 1.24 | 1.60 | 1.83 | 0.66 | 0.80 | 1.66 | 0.71 | 2.08 | 1.388 |
| CD56brightCD16-_NK\|Plasma | 0.00 | 1.12 | 0.00 | 0.63 | 1.28 | 0.00 | 0.00 | 1.39 | 0.94 | 1.13 | 1.18 | 1.26 | 0.00 | 1.73 | 0.57 | 1.06 | 0.00 | 0.77 | 1.63 | 1.533 |
| CD56brightCD16-_NK\|Treg | 0.00 | 1.12 | 0.81 | 0.63 | 0.00 | 0.00 | 1.27 | 1.98 | 1.53 | 1.23 | 1.16 | 1.41 | 0.00 | 1.66 | 0.59 | 0.89 | 1.66 | 0.75 | 1.92 | 1.67 |
| CD56brightCD16-_NK\|γδ_T | 0.00 | 1.15 | 0.82 | 0.68 | 1.61 | 1.30 | 1.29 | 1.98 | 1.53 | 1.19 | 0.00 | 1.44 | 1.61 | 1.70 | 0.59 | 0.87 | 1.70 | 0.74 | 2.01 | 1.543 |
| CD56dimCD16+_NK\|C-Mono1 | 0.00 | 2.03 | 0.96 | 0.76 | 1.42 | 1.42 | 0.00 | 1.18 | 1.16 | 1.18 | 1.24 | 1.41 | 0.00 | 1.86 | 0.58 | 0.63 | 1.82 | 0.79 | 1.62 | 1.942 |
| CD56dimCD16+_NK\|C-Mono2 | 1.81 | 1.99 | 0.91 | 0.74 | 1.42 | 1.42 | 1.42 | 1.10 | 1.08 | 0.98 | 1.23 | 1.40 | 1.66 | 1.84 | 0.57 | 0.56 | 1.82 | 0.73 | 1.45 | 1.929 |
| CD56dimCD16+_NK\|C-Mono3 | 0.00 | 2.11 | 0.91 | 0.75 | 1.43 | 1.42 | 1.42 | 1.20 | 1.18 | 1.05 | 1.24 | 1.41 | 1.66 | 1.84 | 0.56 | 0.56 | 1.83 | 0.75 | 1.48 | 1.931 |
| CD56dimCD16+_NK\|CD4+_effector_T_cell | 1.81 | 1.67 | 0.83 | 0.73 | 1.42 | 1.43 | 1.42 | 1.49 | 1.46 | 1.29 | 0.00 | 1.49 | 1.66 | 1.87 | 0.42 | 0.76 | 1.83 | 0.59 | 1.96 | 1.537 |
| CD56dimCD16+_NK\|CD4+_memory_T_cell | 1.81 | 1.67 | 0.84 | 0.69 | 1.43 | 1.42 | 1.43 | 1.40 | 1.37 | 1.20 | 1.23 | 1.52 | 1.66 | 1.86 | 0.45 | 0.76 | 1.83 | 0.59 | 2.00 | 1.635 |
| CD56dimCD16+_NK\|CD4+_naÃ¯ve_T_cell | 1.81 | 1.67 | 0.83 | 0.69 | 1.42 | 1.42 | 1.42 | 1.26 | 1.24 | 1.12 | 1.23 | 1.48 | 1.66 | 1.84 | 0.47 | 0.76 | 1.83 | 0.54 | 2.09 | 1.523 |
| CD56dimCD16+_NK\|CD56brightCD16-_NK | 1.83 | 1.78 | 0.85 | 0.79 | 2.14 | 1.69 | 1.49 | 1.44 | 1.41 | 1.24 | 0.00 | 1.47 | 1.66 | 1.87 | 0.41 | 0.79 | 1.87 | 0.65 | 1.91 | 1.684 |
| CD56dimCD16+_NK\|CD56dimCD16+_NK | 1.85 | 1.68 | 0.85 | 0.81 | 1.60 | 1.57 | 1.47 | 1.60 | 1.57 | 1.26 | 1.23 | 1.50 | 1.71 | 1.90 | 0.41 | 0.70 | 1.94 | 0.57 | 1.71 | 1.294 |
| CD56dimCD16+_NK\|CD8+_naÃ¯ve_T_cell | 1.82 | 1.68 | 0.83 | 0.73 | 1.48 | 1.49 | 1.44 | 1.43 | 1.41 | 1.20 | 1.23 | 1.55 | 1.67 | 1.87 | 0.46 | 0.81 | 1.84 | 0.58 | 2.04 | 1.5 |
| CD56dimCD16+_NK\|GNLY+CD8+_cytotoxic_T_cell | 1.82 | 1.68 | 0.84 | 0.77 | 1.44 | 1.51 | 1.46 | 1.49 | 1.46 | 1.24 | 1.23 | 1.48 | 1.67 | 1.86 | 0.42 | 0.71 | 1.86 | 0.58 | 1.91 | 1.444 |
| CD56dimCD16+_NK\|GZMK+CD8+_cytotoxic_T_cell | 1.81 | 1.69 | 0.83 | 0.74 | 1.46 | 1.44 | 1.45 | 1.42 | 1.39 | 1.20 | 1.23 | 1.50 | 1.66 | 1.86 | 0.42 | 0.74 | 1.83 | 0.58 | 1.99 | 1.553 |
| CD56dimCD16+_NK\|MAI_T | 0.00 | 1.75 | 0.83 | 0.72 | 1.48 | 1.43 | 1.43 | 1.45 | 1.42 | 1.19 | 0.00 | 1.46 | 0.00 | 1.90 | 0.42 | 0.73 | 0.00 | 0.61 | 1.99 | 1.516 |
| CD56dimCD16+_NK\|Memory_B_cell | 0.00 | 1.68 | 0.90 | 0.69 | 0.00 | 0.00 | 1.42 | 1.35 | 1.33 | 1.28 | 1.61 | 1.32 | 0.00 | 1.95 | 0.46 | 0.74 | 1.83 | 0.59 | 2.00 | 1.524 |
| CD56dimCD16+_NK\|NC-Mono | 1.81 | 1.93 | 1.05 | 0.78 | 1.43 | 0.00 | 0.00 | 1.17 | 1.15 | 1.11 | 1.24 | 1.38 | 0.00 | 1.86 | 0.52 | 0.61 | 1.83 | 0.72 | 1.73 | 1.744 |
| CD56dimCD16+_NK\|NKT | 1.83 | 1.68 | 0.84 | 0.81 | 1.57 | 1.57 | 1.47 | 1.56 | 1.53 | 1.31 | 0.00 | 1.58 | 1.69 | 1.91 | 0.42 | 0.76 | 1.91 | 0.59 | 1.86 | 1.432 |
| CD56dimCD16+_NK\|NaÃ¯ve_B_cell | 0.00 | 1.67 | 0.87 | 0.69 | 1.42 | 1.42 | 1.42 | 1.24 | 1.22 | 1.19 | 1.76 | 1.31 | 1.66 | 2.00 | 0.50 | 0.64 | 1.82 | 0.55 | 2.08 | 1.365 |
| CD56dimCD16+_NK\|Plasma | 0.00 | 1.67 | 0.00 | 0.70 | 1.44 | 0.00 | 0.00 | 0.84 | 0.82 | 1.17 | 1.25 | 1.33 | 0.00 | 1.91 | 0.42 | 0.90 | 0.00 | 0.61 | 1.62 | 1.51 |
| CD56dimCD16+_NK\|Treg | 0.00 | 1.67 | 0.83 | 0.70 | 0.00 | 0.00 | 1.42 | 1.43 | 1.41 | 1.28 | 1.23 | 1.48 | 0.00 | 1.83 | 0.43 | 0.73 | 1.83 | 0.59 | 1.92 | 1.647 |
| CD56dimCD16+_NK\|γδ_T | 0.00 | 1.70 | 0.84 | 0.75 | 1.77 | 1.45 | 1.45 | 1.44 | 1.41 | 1.24 | 0.00 | 1.51 | 1.66 | 1.87 | 0.43 | 0.71 | 1.86 | 0.58 | 2.00 | 1.52 |
| CD8+_naÃ¯ve_T_cell\|C-Mono1 | 0.00 | 1.36 | 1.27 | 0.97 | 1.26 | 1.26 | 0.00 | 1.07 | 1.07 | 1.09 | 1.12 | 1.29 | 0.00 | 1.60 | 0.54 | 0.59 | 1.64 | 0.75 | 1.62 | 1.219 |
| CD8+_naÃ¯ve_T_cell\|C-Mono2 | 1.55 | 1.31 | 1.22 | 0.95 | 1.26 | 1.26 | 1.26 | 0.99 | 0.99 | 0.89 | 1.12 | 1.28 | 1.47 | 1.58 | 0.53 | 0.52 | 1.64 | 0.69 | 1.45 | 1.207 |
| CD8+_naÃ¯ve_T_cell\|C-Mono3 | 0.00 | 1.44 | 1.23 | 0.96 | 1.26 | 1.26 | 1.26 | 1.09 | 1.09 | 0.96 | 1.12 | 1.30 | 1.47 | 1.58 | 0.52 | 0.52 | 1.64 | 0.70 | 1.48 | 1.208 |
| CD8+_naÃ¯ve_T_cell\|CD4+_effector_T_cell | 1.56 | 1.00 | 1.15 | 0.94 | 1.26 | 1.26 | 1.26 | 1.37 | 1.38 | 1.20 | 0.00 | 1.37 | 1.47 | 1.61 | 0.38 | 0.72 | 1.64 | 0.55 | 1.96 | 0.814 |
| CD8+_naÃ¯ve_T_cell\|CD4+_memory_T_cell | 1.55 | 1.00 | 1.15 | 0.90 | 1.26 | 1.26 | 1.26 | 1.29 | 1.29 | 1.11 | 1.11 | 1.40 | 1.47 | 1.60 | 0.40 | 0.72 | 1.64 | 0.54 | 1.99 | 0.913 |
| CD8+_naÃ¯ve_T_cell\|CD4+_naÃ¯ve_T_cell | 1.55 | 1.00 | 1.15 | 0.90 | 1.26 | 1.26 | 1.26 | 1.15 | 1.15 | 1.03 | 1.11 | 1.36 | 1.47 | 1.59 | 0.43 | 0.72 | 1.64 | 0.50 | 2.09 | 0.801 |
| CD8+_naÃ¯ve_T_cell\|CD56brightCD16-_NK | 1.57 | 1.10 | 1.17 | 1.00 | 1.98 | 1.53 | 1.33 | 1.33 | 1.33 | 1.15 | 0.00 | 1.36 | 1.47 | 1.61 | 0.37 | 0.74 | 1.68 | 0.61 | 1.91 | 0.961 |
| CD8+_naÃ¯ve_T_cell\|CD56dimCD16+_NK | 1.59 | 1.01 | 1.16 | 1.02 | 1.43 | 1.41 | 1.30 | 1.48 | 1.49 | 1.17 | 1.11 | 1.38 | 1.52 | 1.65 | 0.37 | 0.66 | 1.75 | 0.53 | 1.71 | 0.571 |
| CD8+_naÃ¯ve_T_cell\|CD8+_naÃ¯ve_T_cell | 1.56 | 1.00 | 1.15 | 0.94 | 1.32 | 1.32 | 1.28 | 1.32 | 1.32 | 1.11 | 1.11 | 1.43 | 1.48 | 1.61 | 0.42 | 0.77 | 1.65 | 0.54 | 2.03 | 0.778 |
| CD8+_naÃ¯ve_T_cell\|GNLY+CD8+_cytotoxic_T_cell | 1.56 | 1.01 | 1.15 | 0.98 | 1.28 | 1.34 | 1.30 | 1.38 | 1.38 | 1.15 | 1.11 | 1.36 | 1.48 | 1.60 | 0.38 | 0.67 | 1.68 | 0.54 | 1.91 | 0.722 |
| CD8+_naÃ¯ve_T_cell\|GZMK+CD8+_cytotoxic_T_cell | 1.56 | 1.02 | 1.15 | 0.95 | 1.29 | 1.28 | 1.29 | 1.31 | 1.31 | 1.11 | 1.11 | 1.39 | 1.47 | 1.60 | 0.38 | 0.70 | 1.64 | 0.54 | 1.99 | 0.831 |
| CD8+_naÃ¯ve_T_cell\|MAI_T | 0.00 | 1.08 | 1.15 | 0.93 | 1.32 | 1.26 | 1.27 | 1.33 | 1.34 | 1.10 | 0.00 | 1.34 | 0.00 | 1.64 | 0.37 | 0.68 | 0.00 | 0.57 | 1.98 | 0.793 |
| CD8+_naÃ¯ve_T_cell\|Memory_B_cell | 0.00 | 1.01 | 1.22 | 0.90 | 0.00 | 0.00 | 1.26 | 1.24 | 1.24 | 1.19 | 1.50 | 1.20 | 0.00 | 1.69 | 0.42 | 0.70 | 1.64 | 0.55 | 2.00 | 0.802 |
| CD8+_naÃ¯ve_T_cell\|NC-Mono | 1.56 | 1.25 | 1.37 | 0.99 | 1.26 | 0.00 | 0.00 | 1.06 | 1.06 | 1.02 | 1.12 | 1.26 | 0.00 | 1.60 | 0.48 | 0.57 | 1.64 | 0.68 | 1.72 | 1.022 |
| CD8+_naÃ¯ve_T_cell\|NKT | 1.57 | 1.00 | 1.16 | 1.02 | 1.40 | 1.41 | 1.31 | 1.45 | 1.45 | 1.21 | 0.00 | 1.46 | 1.50 | 1.65 | 0.38 | 0.72 | 1.72 | 0.55 | 1.86 | 0.71 |
| CD8+_naÃ¯ve_T_cell\|NaÃ¯ve_B_cell | 0.00 | 1.00 | 1.18 | 0.90 | 1.26 | 1.26 | 1.26 | 1.13 | 1.14 | 1.10 | 1.64 | 1.19 | 1.47 | 1.74 | 0.46 | 0.60 | 1.64 | 0.50 | 2.08 | 0.643 |
| CD8+_naÃ¯ve_T_cell\|Plasma | 0.00 | 1.00 | 0.00 | 0.91 | 1.27 | 0.00 | 0.00 | 0.73 | 0.74 | 1.08 | 1.13 | 1.21 | 0.00 | 1.65 | 0.37 | 0.86 | 0.00 | 0.57 | 1.62 | 0.788 |
| CD8+_naÃ¯ve_T_cell\|Treg | 0.00 | 1.00 | 1.15 | 0.91 | 0.00 | 0.00 | 1.26 | 1.32 | 1.33 | 1.19 | 1.11 | 1.36 | 0.00 | 1.57 | 0.39 | 0.69 | 1.64 | 0.55 | 1.92 | 0.925 |
| CD8+_naÃ¯ve_T_cell\|γδ_T | 0.00 | 1.02 | 1.16 | 0.96 | 1.61 | 1.29 | 1.28 | 1.32 | 1.33 | 1.14 | 0.00 | 1.39 | 1.47 | 1.62 | 0.39 | 0.67 | 1.67 | 0.54 | 2.00 | 0.798 |
| GNLY+CD8+_cytotoxic_T_cell\|C-Mono1 | 0.00 | 2.14 | 1.37 | 0.85 | 1.32 | 1.32 | 0.00 | 1.03 | 1.09 | 1.11 | 1.30 | 1.47 | 0.00 | 1.76 | 0.69 | 0.74 | 1.76 | 0.90 | 1.62 | 1.182 |
| GNLY+CD8+_cytotoxic_T_cell\|C-Mono2 | 1.71 | 2.10 | 1.32 | 0.83 | 1.32 | 1.32 | 1.32 | 0.95 | 1.01 | 0.90 | 1.30 | 1.46 | 1.60 | 1.73 | 0.67 | 0.67 | 1.76 | 0.83 | 1.44 | 1.17 |
| GNLY+CD8+_cytotoxic_T_cell\|C-Mono3 | 0.00 | 2.23 | 1.32 | 0.83 | 1.32 | 1.32 | 1.32 | 1.05 | 1.11 | 0.97 | 1.30 | 1.47 | 1.60 | 1.74 | 0.66 | 0.67 | 1.76 | 0.85 | 1.48 | 1.171 |
| GNLY+CD8+_cytotoxic_T_cell\|CD4+_effector_T_cell | 1.71 | 1.79 | 1.25 | 0.82 | 1.32 | 1.32 | 1.32 | 1.33 | 1.40 | 1.21 | 0.00 | 1.55 | 1.60 | 1.76 | 0.52 | 0.86 | 1.77 | 0.70 | 1.96 | 0.777 |
| GNLY+CD8+_cytotoxic_T_cell\|CD4+_memory_T_cell | 1.71 | 1.79 | 1.25 | 0.78 | 1.32 | 1.32 | 1.32 | 1.25 | 1.31 | 1.13 | 1.29 | 1.58 | 1.60 | 1.75 | 0.55 | 0.86 | 1.77 | 0.69 | 1.99 | 0.876 |
| GNLY+CD8+_cytotoxic_T_cell\|CD4+_naÃ¯ve_T_cell | 1.71 | 1.79 | 1.24 | 0.78 | 1.32 | 1.32 | 1.32 | 1.11 | 1.17 | 1.04 | 1.29 | 1.54 | 1.60 | 1.74 | 0.58 | 0.87 | 1.76 | 0.64 | 2.09 | 0.764 |
| GNLY+CD8+_cytotoxic_T_cell\|CD56brightCD16-_NK | 1.72 | 1.89 | 1.26 | 0.88 | 2.03 | 1.58 | 1.39 | 1.28 | 1.35 | 1.16 | 0.00 | 1.54 | 1.60 | 1.76 | 0.52 | 0.89 | 1.81 | 0.75 | 1.91 | 0.925 |
| GNLY+CD8+_cytotoxic_T_cell\|CD56dimCD16+_NK | 1.74 | 1.79 | 1.26 | 0.89 | 1.49 | 1.46 | 1.36 | 1.44 | 1.51 | 1.18 | 1.29 | 1.56 | 1.65 | 1.80 | 0.52 | 0.80 | 1.88 | 0.68 | 1.70 | 0.535 |
| GNLY+CD8+_cytotoxic_T_cell\|CD8+_naÃ¯ve_T_cell | 1.72 | 1.79 | 1.24 | 0.81 | 1.38 | 1.38 | 1.34 | 1.28 | 1.34 | 1.12 | 1.29 | 1.61 | 1.61 | 1.76 | 0.57 | 0.91 | 1.78 | 0.69 | 2.03 | 0.741 |
| GNLY+CD8+_cytotoxic_T_cell\|GNLY+CD8+_cytotoxic_T_cell | 1.71 | 1.80 | 1.25 | 0.85 | 1.33 | 1.40 | 1.36 | 1.33 | 1.40 | 1.17 | 1.29 | 1.54 | 1.61 | 1.76 | 0.52 | 0.82 | 1.80 | 0.68 | 1.91 | 0.685 |
| GNLY+CD8+_cytotoxic_T_cell\|GZMK+CD8+_cytotoxic_T_cell | 1.71 | 1.81 | 1.24 | 0.83 | 1.35 | 1.33 | 1.34 | 1.27 | 1.33 | 1.12 | 1.29 | 1.57 | 1.60 | 1.76 | 0.52 | 0.85 | 1.77 | 0.68 | 1.99 | 0.794 |
| GNLY+CD8+_cytotoxic_T_cell\|MAI_T | 0.00 | 1.87 | 1.25 | 0.80 | 1.37 | 1.32 | 1.33 | 1.29 | 1.36 | 1.12 | 0.00 | 1.52 | 0.00 | 1.80 | 0.52 | 0.83 | 0.00 | 0.71 | 1.98 | 0.756 |
| GNLY+CD8+_cytotoxic_T_cell\|Memory_B_cell | 0.00 | 1.80 | 1.31 | 0.78 | 0.00 | 0.00 | 1.32 | 1.20 | 1.26 | 1.20 | 1.68 | 1.38 | 0.00 | 1.85 | 0.56 | 0.84 | 1.77 | 0.69 | 1.99 | 0.765 |
| GNLY+CD8+_cytotoxic_T_cell\|NC-Mono | 1.71 | 2.04 | 1.46 | 0.86 | 1.32 | 0.00 | 0.00 | 1.02 | 1.09 | 1.03 | 1.30 | 1.44 | 0.00 | 1.76 | 0.62 | 0.72 | 1.76 | 0.83 | 1.72 | 0.985 |
| GNLY+CD8+_cytotoxic_T_cell\|NKT | 1.72 | 1.79 | 1.26 | 0.89 | 1.46 | 1.46 | 1.37 | 1.41 | 1.47 | 1.23 | 0.00 | 1.64 | 1.63 | 1.80 | 0.52 | 0.87 | 1.84 | 0.69 | 1.85 | 0.673 |
| GNLY+CD8+_cytotoxic_T_cell\|NaÃ¯ve_B_cell | 0.00 | 1.79 | 1.28 | 0.78 | 1.32 | 1.32 | 1.32 | 1.09 | 1.16 | 1.11 | 1.82 | 1.37 | 1.60 | 1.89 | 0.61 | 0.74 | 1.76 | 0.65 | 2.07 | 0.606 |
| GNLY+CD8+_cytotoxic_T_cell\|Plasma | 0.00 | 1.79 | 0.00 | 0.78 | 1.33 | 0.00 | 0.00 | 0.69 | 0.76 | 1.10 | 1.31 | 1.39 | 0.00 | 1.80 | 0.52 | 1.01 | 0.00 | 0.72 | 1.62 | 0.751 |
| GNLY+CD8+_cytotoxic_T_cell\|Treg | 0.00 | 1.79 | 1.25 | 0.78 | 0.00 | 0.00 | 1.32 | 1.28 | 1.35 | 1.20 | 1.29 | 1.54 | 0.00 | 1.73 | 0.53 | 0.84 | 1.76 | 0.70 | 1.92 | 0.888 |
| GNLY+CD8+_cytotoxic_T_cell\|γδ_T | 0.00 | 1.81 | 1.25 | 0.84 | 1.66 | 1.35 | 1.34 | 1.28 | 1.35 | 1.16 | 0.00 | 1.57 | 1.60 | 1.77 | 0.53 | 0.82 | 1.80 | 0.69 | 2.00 | 0.761 |
| GZMK+CD8+_cytotoxic_T_cell\|C-Mono1 | 0.00 | 2.10 | 1.28 | 0.85 | 1.25 | 1.25 | 0.00 | 1.04 | 1.03 | 1.07 | 1.27 | 1.44 | 0.00 | 1.68 | 0.83 | 0.88 | 1.69 | 1.04 | 1.62 | 0.955 |
| GZMK+CD8+_cytotoxic_T_cell\|C-Mono2 | 1.63 | 2.06 | 1.23 | 0.83 | 1.25 | 1.25 | 1.25 | 0.96 | 0.95 | 0.87 | 1.27 | 1.43 | 1.58 | 1.66 | 0.82 | 0.81 | 1.69 | 0.98 | 1.44 | 0.943 |
| GZMK+CD8+_cytotoxic_T_cell\|C-Mono3 | 0.00 | 2.18 | 1.24 | 0.84 | 1.25 | 1.25 | 1.25 | 1.06 | 1.05 | 0.93 | 1.27 | 1.45 | 1.58 | 1.66 | 0.81 | 0.81 | 1.70 | 1.00 | 1.48 | 0.944 |
| GZMK+CD8+_cytotoxic_T_cell\|CD4+_effector_T_cell | 1.63 | 1.74 | 1.16 | 0.82 | 1.25 | 1.25 | 1.25 | 1.35 | 1.33 | 1.18 | 0.00 | 1.53 | 1.58 | 1.69 | 0.67 | 1.01 | 1.70 | 0.84 | 1.96 | 0.55 |
| GZMK+CD8+_cytotoxic_T_cell\|CD4+_memory_T_cell | 1.63 | 1.74 | 1.16 | 0.78 | 1.25 | 1.25 | 1.25 | 1.26 | 1.24 | 1.09 | 1.27 | 1.55 | 1.58 | 1.67 | 0.70 | 1.01 | 1.70 | 0.84 | 1.99 | 0.649 |
| GZMK+CD8+_cytotoxic_T_cell\|CD4+_naÃ¯ve_T_cell | 1.63 | 1.74 | 1.16 | 0.78 | 1.25 | 1.25 | 1.25 | 1.12 | 1.11 | 1.01 | 1.27 | 1.52 | 1.58 | 1.66 | 0.72 | 1.01 | 1.70 | 0.79 | 2.09 | 0.537 |
| GZMK+CD8+_cytotoxic_T_cell\|CD56brightCD16-_NK | 1.65 | 1.85 | 1.18 | 0.88 | 1.96 | 1.51 | 1.32 | 1.30 | 1.28 | 1.13 | 0.00 | 1.51 | 1.59 | 1.69 | 0.66 | 1.04 | 1.74 | 0.90 | 1.91 | 0.697 |
| GZMK+CD8+_cytotoxic_T_cell\|CD56dimCD16+_NK | 1.67 | 1.75 | 1.17 | 0.90 | 1.42 | 1.39 | 1.29 | 1.46 | 1.44 | 1.15 | 1.27 | 1.53 | 1.64 | 1.72 | 0.66 | 0.95 | 1.81 | 0.82 | 1.70 | 0.307 |
| GZMK+CD8+_cytotoxic_T_cell\|CD8+_naÃ¯ve_T_cell | 1.64 | 1.75 | 1.16 | 0.82 | 1.31 | 1.31 | 1.27 | 1.29 | 1.28 | 1.08 | 1.27 | 1.58 | 1.60 | 1.69 | 0.71 | 1.06 | 1.71 | 0.83 | 2.03 | 0.514 |
| GZMK+CD8+_cytotoxic_T_cell\|GNLY+CD8+_cytotoxic_T_cell | 1.64 | 1.75 | 1.16 | 0.86 | 1.27 | 1.33 | 1.29 | 1.35 | 1.33 | 1.13 | 1.27 | 1.51 | 1.60 | 1.68 | 0.67 | 0.96 | 1.73 | 0.83 | 1.91 | 0.458 |
| GZMK+CD8+_cytotoxic_T_cell\|GZMK+CD8+_cytotoxic_T_cell | 1.63 | 1.76 | 1.16 | 0.83 | 1.28 | 1.27 | 1.28 | 1.28 | 1.27 | 1.09 | 1.27 | 1.54 | 1.58 | 1.68 | 0.67 | 0.99 | 1.70 | 0.83 | 1.99 | 0.567 |
| GZMK+CD8+_cytotoxic_T_cell\|MAI_T | 0.00 | 1.82 | 1.16 | 0.81 | 1.30 | 1.25 | 1.26 | 1.31 | 1.29 | 1.08 | 0.00 | 1.49 | 0.00 | 1.72 | 0.67 | 0.98 | 0.00 | 0.86 | 1.98 | 0.529 |
| GZMK+CD8+_cytotoxic_T_cell\|Memory_B_cell | 0.00 | 1.75 | 1.23 | 0.78 | 0.00 | 0.00 | 1.25 | 1.21 | 1.20 | 1.16 | 1.65 | 1.36 | 0.00 | 1.77 | 0.71 | 0.99 | 1.70 | 0.84 | 1.99 | 0.538 |
| GZMK+CD8+_cytotoxic_T_cell\|NC-Mono | 1.63 | 2.00 | 1.37 | 0.87 | 1.25 | 0.00 | 0.00 | 1.04 | 1.02 | 0.99 | 1.28 | 1.41 | 0.00 | 1.68 | 0.77 | 0.86 | 1.70 | 0.97 | 1.72 | 0.757 |
| GZMK+CD8+_cytotoxic_T_cell\|NKT | 1.65 | 1.74 | 1.17 | 0.90 | 1.39 | 1.39 | 1.30 | 1.42 | 1.41 | 1.19 | 0.00 | 1.61 | 1.62 | 1.72 | 0.67 | 1.01 | 1.77 | 0.84 | 1.85 | 0.446 |
| GZMK+CD8+_cytotoxic_T_cell\|NaÃ¯ve_B_cell | 0.00 | 1.74 | 1.19 | 0.78 | 1.25 | 1.25 | 1.25 | 1.11 | 1.09 | 1.08 | 1.80 | 1.34 | 1.58 | 1.82 | 0.75 | 0.89 | 1.69 | 0.80 | 2.07 | 0.379 |
| GZMK+CD8+_cytotoxic_T_cell\|Plasma | 0.00 | 1.74 | 0.00 | 0.79 | 1.26 | 0.00 | 0.00 | 0.71 | 0.69 | 1.06 | 1.29 | 1.36 | 0.00 | 1.72 | 0.67 | 1.15 | 0.00 | 0.86 | 1.62 | 0.524 |
| GZMK+CD8+_cytotoxic_T_cell\|Treg | 0.00 | 1.74 | 1.16 | 0.79 | 0.00 | 0.00 | 1.25 | 1.30 | 1.28 | 1.16 | 1.27 | 1.51 | 0.00 | 1.65 | 0.68 | 0.98 | 1.69 | 0.84 | 1.92 | 0.661 |
| GZMK+CD8+_cytotoxic_T_cell\|γδ_T | 0.00 | 1.76 | 1.17 | 0.84 | 1.59 | 1.28 | 1.27 | 1.30 | 1.28 | 1.12 | 0.00 | 1.54 | 1.59 | 1.69 | 0.68 | 0.96 | 1.73 | 0.83 | 2.00 | 0.533 |
| MAI_T\|C-Mono1 | 0.00 | 1.82 | 1.27 | 0.91 | 1.27 | 1.27 | 0.00 | 1.07 | 1.01 | 1.07 | 1.10 | 1.27 | 0.00 | 1.67 | 0.60 | 0.65 | 1.63 | 0.81 | 1.62 | 1.23 |
| MAI_T\|C-Mono2 | 1.63 | 1.78 | 1.22 | 0.89 | 1.27 | 1.27 | 1.27 | 0.99 | 0.93 | 0.87 | 1.10 | 1.27 | 1.53 | 1.65 | 0.58 | 0.58 | 1.63 | 0.74 | 1.44 | 1.217 |
| MAI_T\|C-Mono3 | 0.00 | 1.90 | 1.23 | 0.89 | 1.28 | 1.28 | 1.28 | 1.09 | 1.03 | 0.93 | 1.10 | 1.28 | 1.53 | 1.65 | 0.57 | 0.58 | 1.63 | 0.76 | 1.48 | 1.219 |
| MAI_T\|CD4+_effector_T_cell | 1.63 | 1.47 | 1.15 | 0.88 | 1.27 | 1.28 | 1.27 | 1.37 | 1.32 | 1.17 | 0.00 | 1.36 | 1.53 | 1.68 | 0.43 | 0.77 | 1.63 | 0.61 | 1.96 | 0.825 |
| MAI_T\|CD4+_memory_T_cell | 1.63 | 1.47 | 1.15 | 0.84 | 1.28 | 1.27 | 1.28 | 1.28 | 1.23 | 1.09 | 1.10 | 1.39 | 1.53 | 1.67 | 0.46 | 0.77 | 1.64 | 0.60 | 1.99 | 0.923 |
| MAI_T\|CD4+_naÃ¯ve_T_cell | 1.63 | 1.47 | 1.15 | 0.84 | 1.27 | 1.27 | 1.27 | 1.15 | 1.09 | 1.01 | 1.10 | 1.35 | 1.53 | 1.66 | 0.49 | 0.78 | 1.63 | 0.55 | 2.09 | 0.811 |
| MAI_T\|CD56brightCD16-_NK | 1.64 | 1.57 | 1.17 | 0.94 | 1.99 | 1.54 | 1.34 | 1.32 | 1.27 | 1.12 | 0.00 | 1.34 | 1.53 | 1.68 | 0.43 | 0.80 | 1.67 | 0.66 | 1.91 | 0.972 |
| MAI_T\|CD56dimCD16+_NK | 1.66 | 1.47 | 1.16 | 0.95 | 1.45 | 1.42 | 1.32 | 1.48 | 1.43 | 1.15 | 1.10 | 1.37 | 1.58 | 1.72 | 0.43 | 0.71 | 1.74 | 0.59 | 1.70 | 0.582 |
| MAI_T\|CD8+_naÃ¯ve_T_cell | 1.64 | 1.47 | 1.15 | 0.87 | 1.33 | 1.34 | 1.29 | 1.32 | 1.26 | 1.08 | 1.10 | 1.41 | 1.54 | 1.68 | 0.48 | 0.82 | 1.65 | 0.60 | 2.03 | 0.788 |
| MAI_T\|GNLY+CD8+_cytotoxic_T_cell | 1.63 | 1.48 | 1.15 | 0.91 | 1.29 | 1.36 | 1.31 | 1.37 | 1.32 | 1.13 | 1.10 | 1.35 | 1.54 | 1.68 | 0.43 | 0.73 | 1.67 | 0.59 | 1.91 | 0.732 |
| MAI_T\|GZMK+CD8+_cytotoxic_T_cell | 1.63 | 1.49 | 1.15 | 0.88 | 1.31 | 1.29 | 1.30 | 1.30 | 1.25 | 1.08 | 1.10 | 1.37 | 1.53 | 1.67 | 0.44 | 0.76 | 1.63 | 0.59 | 1.99 | 0.841 |
| MAI_T\|MAI_T | 0.00 | 1.55 | 1.15 | 0.86 | 1.33 | 1.28 | 1.28 | 1.33 | 1.28 | 1.08 | 0.00 | 1.32 | 0.00 | 1.71 | 0.43 | 0.74 | 0.00 | 0.62 | 1.98 | 0.804 |
| MAI_T\|Memory_B_cell | 0.00 | 1.47 | 1.22 | 0.83 | 0.00 | 0.00 | 1.27 | 1.24 | 1.18 | 1.16 | 1.48 | 1.19 | 0.00 | 1.77 | 0.47 | 0.75 | 1.63 | 0.60 | 1.99 | 0.812 |
| MAI_T\|NC-Mono | 1.63 | 1.72 | 1.36 | 0.92 | 1.28 | 0.00 | 0.00 | 1.06 | 1.00 | 0.99 | 1.11 | 1.24 | 0.00 | 1.67 | 0.54 | 0.63 | 1.63 | 0.74 | 1.72 | 1.032 |
| MAI_T\|NKT | 1.64 | 1.47 | 1.16 | 0.95 | 1.42 | 1.42 | 1.32 | 1.44 | 1.39 | 1.19 | 0.00 | 1.44 | 1.56 | 1.72 | 0.43 | 0.78 | 1.71 | 0.61 | 1.85 | 0.72 |
| MAI_T\|NaÃ¯ve_B_cell | 0.00 | 1.46 | 1.18 | 0.84 | 1.27 | 1.27 | 1.28 | 1.13 | 1.08 | 1.07 | 1.63 | 1.18 | 1.53 | 1.81 | 0.52 | 0.65 | 1.63 | 0.56 | 2.07 | 0.653 |
| MAI_T\|Plasma | 0.00 | 1.46 | 0.00 | 0.84 | 1.29 | 0.00 | 0.00 | 0.73 | 0.68 | 1.06 | 1.12 | 1.19 | 0.00 | 1.72 | 0.43 | 0.92 | 0.00 | 0.63 | 1.62 | 0.799 |
| MAI_T\|Treg | 0.00 | 1.46 | 1.15 | 0.84 | 0.00 | 0.00 | 1.27 | 1.32 | 1.26 | 1.16 | 1.10 | 1.35 | 0.00 | 1.65 | 0.45 | 0.75 | 1.63 | 0.61 | 1.92 | 0.935 |
| MAI_T\|γδ_T | 0.00 | 1.49 | 1.15 | 0.90 | 1.62 | 1.30 | 1.30 | 1.32 | 1.27 | 1.12 | 0.00 | 1.38 | 1.53 | 1.69 | 0.44 | 0.73 | 1.66 | 0.60 | 2.00 | 0.808 |
| Memory_B_cell\|C-Mono1 | 0.00 | 0.47 | 1.49 | 0.79 | 1.18 | 1.18 | 0.00 | 0.00 | 0.00 | 1.01 | 0.83 | 1.00 | 0.00 | 1.45 | 2.06 | 2.11 | 1.65 | 2.27 | 1.62 | 0.935 |
| Memory_B_cell\|C-Mono2 | 1.40 | 0.43 | 1.44 | 0.77 | 1.18 | 1.18 | 1.18 | 0.00 | 0.00 | 0.80 | 0.83 | 0.99 | 1.37 | 1.42 | 2.04 | 2.04 | 1.65 | 2.21 | 1.44 | 0.923 |
| Memory_B_cell\|C-Mono3 | 0.00 | 0.55 | 1.44 | 0.77 | 1.18 | 1.18 | 1.18 | 0.00 | 0.00 | 0.87 | 0.83 | 1.01 | 1.37 | 1.43 | 2.04 | 2.04 | 1.65 | 2.22 | 1.47 | 0.925 |
| Memory_B_cell\|CD4+_effector_T_cell | 1.40 | 0.11 | 1.37 | 0.76 | 1.18 | 1.18 | 1.18 | 0.00 | 0.00 | 1.11 | 0.00 | 1.09 | 1.37 | 1.45 | 1.90 | 2.24 | 1.65 | 2.07 | 1.95 | 0.53 |
| Memory_B_cell\|CD4+_memory_T_cell | 1.40 | 0.11 | 1.37 | 0.72 | 1.18 | 1.18 | 1.18 | 0.00 | 0.00 | 1.02 | 0.83 | 1.11 | 1.37 | 1.44 | 1.92 | 2.24 | 1.65 | 2.06 | 1.99 | 0.629 |
| Memory_B_cell\|CD4+_naÃ¯ve_T_cell | 1.40 | 0.11 | 1.36 | 0.72 | 1.18 | 1.18 | 1.18 | 0.00 | 0.00 | 0.94 | 0.83 | 1.08 | 1.37 | 1.43 | 1.95 | 2.24 | 1.65 | 2.01 | 2.09 | 0.517 |
| Memory_B_cell\|CD56brightCD16-_NK | 1.42 | 0.22 | 1.38 | 0.82 | 1.90 | 1.45 | 1.25 | 0.00 | 0.00 | 1.06 | 0.00 | 1.07 | 1.37 | 1.45 | 1.89 | 2.26 | 1.69 | 2.13 | 1.91 | 0.678 |
| Memory_B_cell\|CD56dimCD16+_NK | 1.44 | 0.12 | 1.38 | 0.84 | 1.35 | 1.33 | 1.22 | 0.00 | 0.00 | 1.08 | 0.82 | 1.09 | 1.42 | 1.49 | 1.89 | 2.18 | 1.76 | 2.05 | 1.70 | 0.288 |
| Memory_B_cell\|CD8+_naÃ¯ve_T_cell | 1.41 | 0.12 | 1.36 | 0.75 | 1.24 | 1.24 | 1.20 | 0.00 | 0.00 | 1.02 | 0.83 | 1.14 | 1.38 | 1.46 | 1.94 | 2.28 | 1.66 | 2.06 | 2.03 | 0.494 |
| Memory_B_cell\|GNLY+CD8+_cytotoxic_T_cell | 1.41 | 0.12 | 1.37 | 0.80 | 1.20 | 1.26 | 1.22 | 0.00 | 0.00 | 1.07 | 0.83 | 1.07 | 1.38 | 1.45 | 1.89 | 2.19 | 1.68 | 2.06 | 1.90 | 0.438 |
| Memory_B_cell\|GZMK+CD8+_cytotoxic_T_cell | 1.40 | 0.13 | 1.36 | 0.77 | 1.21 | 1.20 | 1.21 | 0.00 | 0.00 | 1.02 | 0.82 | 1.10 | 1.37 | 1.45 | 1.90 | 2.22 | 1.65 | 2.06 | 1.98 | 0.547 |
| Memory_B_cell\|MAI_T | 0.00 | 0.19 | 1.37 | 0.75 | 1.24 | 1.18 | 1.19 | 0.00 | 0.00 | 1.01 | 0.00 | 1.05 | 0.00 | 1.49 | 1.89 | 2.20 | 0.00 | 2.09 | 1.98 | 0.51 |
| Memory_B_cell\|Memory_B_cell | 0.00 | 0.12 | 1.43 | 0.72 | 0.00 | 0.00 | 1.18 | 0.00 | 0.00 | 1.10 | 1.21 | 0.92 | 0.00 | 1.54 | 1.94 | 2.22 | 1.65 | 2.06 | 1.99 | 0.518 |
| Memory_B_cell\|NC-Mono | 1.40 | 0.37 | 1.58 | 0.80 | 1.18 | 0.00 | 0.00 | 0.00 | 0.00 | 0.93 | 0.84 | 0.97 | 0.00 | 1.45 | 2.00 | 2.09 | 1.65 | 2.20 | 1.72 | 0.738 |
| Memory_B_cell\|NKT | 1.41 | 0.12 | 1.38 | 0.83 | 1.32 | 1.33 | 1.23 | 0.00 | 0.00 | 1.13 | 0.00 | 1.17 | 1.40 | 1.49 | 1.90 | 2.24 | 1.73 | 2.07 | 1.85 | 0.426 |
| Memory_B_cell\|NaÃ¯ve_B_cell | 0.00 | 0.11 | 1.40 | 0.72 | 1.18 | 1.18 | 1.18 | 0.00 | 0.00 | 1.01 | 1.36 | 0.90 | 1.37 | 1.59 | 1.98 | 2.12 | 1.65 | 2.02 | 2.07 | 0.359 |
| Memory_B_cell\|Plasma | 0.00 | 0.11 | 0.00 | 0.72 | 1.19 | 0.00 | 0.00 | 0.00 | 0.00 | 1.00 | 0.84 | 0.92 | 0.00 | 1.49 | 1.89 | 2.38 | 0.00 | 2.09 | 1.62 | 0.504 |
| Memory_B_cell\|Treg | 0.00 | 0.11 | 1.37 | 0.72 | 0.00 | 0.00 | 1.18 | 0.00 | 0.00 | 1.10 | 0.83 | 1.07 | 0.00 | 1.42 | 1.91 | 2.21 | 1.65 | 2.07 | 1.91 | 0.641 |
| Memory_B_cell\|γδ_T | 0.00 | 0.14 | 1.37 | 0.78 | 1.53 | 1.21 | 1.20 | 0.00 | 0.00 | 1.06 | 0.00 | 1.10 | 1.37 | 1.46 | 1.91 | 2.19 | 1.68 | 2.06 | 2.00 | 0.514 |
| NC-Mono\|C-Mono1 | 0.00 | 0.47 | 1.08 | 0.90 | 1.00 | 1.00 | 0.00 | 1.01 | 0.00 | 1.13 | 1.16 | 1.33 | 0.00 | 1.44 | 1.53 | 1.58 | 1.60 | 1.75 | 2.19 | 2.451 |
| NC-Mono\|C-Mono2 | 1.39 | 0.43 | 1.03 | 0.87 | 1.00 | 1.00 | 1.00 | 0.93 | 0.00 | 0.93 | 1.16 | 1.33 | 1.26 | 1.41 | 1.52 | 1.51 | 1.60 | 1.68 | 2.02 | 2.439 |
| NC-Mono\|C-Mono3 | 0.00 | 0.55 | 1.03 | 0.88 | 1.00 | 1.00 | 1.00 | 1.03 | 0.00 | 0.99 | 1.16 | 1.34 | 1.26 | 1.42 | 1.51 | 1.51 | 1.60 | 1.70 | 2.05 | 2.44 |
| NC-Mono\|CD4+_effector_T_cell | 1.39 | 0.12 | 0.95 | 0.87 | 1.00 | 1.00 | 1.00 | 1.32 | 0.00 | 1.23 | 0.00 | 1.42 | 1.26 | 1.45 | 1.37 | 1.71 | 1.60 | 1.54 | 2.53 | 2.046 |
| NC-Mono\|CD4+_memory_T_cell | 1.39 | 0.12 | 0.95 | 0.83 | 1.00 | 1.00 | 1.00 | 1.23 | 0.00 | 1.15 | 1.16 | 1.45 | 1.26 | 1.43 | 1.40 | 1.71 | 1.60 | 1.54 | 2.56 | 2.144 |
| NC-Mono\|CD4+_naÃ¯ve_T_cell | 1.39 | 0.11 | 0.95 | 0.83 | 1.00 | 1.00 | 1.00 | 1.09 | 0.00 | 1.07 | 1.16 | 1.41 | 1.26 | 1.42 | 1.43 | 1.71 | 1.60 | 1.49 | 2.66 | 2.032 |
| NC-Mono\|CD56brightCD16-_NK | 1.41 | 0.22 | 0.97 | 0.93 | 1.72 | 1.27 | 1.07 | 1.27 | 0.00 | 1.19 | 0.00 | 1.40 | 1.27 | 1.45 | 1.36 | 1.74 | 1.64 | 1.60 | 2.48 | 2.193 |
| NC-Mono\|CD56dimCD16+_NK | 1.43 | 0.12 | 0.97 | 0.94 | 1.17 | 1.15 | 1.05 | 1.43 | 0.00 | 1.21 | 1.16 | 1.43 | 1.31 | 1.48 | 1.37 | 1.65 | 1.71 | 1.52 | 2.27 | 1.803 |
| NC-Mono\|CD8+_naÃ¯ve_T_cell | 1.40 | 0.12 | 0.95 | 0.86 | 1.06 | 1.06 | 1.02 | 1.26 | 0.00 | 1.14 | 1.16 | 1.47 | 1.27 | 1.45 | 1.41 | 1.76 | 1.61 | 1.54 | 2.60 | 2.01 |
| NC-Mono\|GNLY+CD8+_cytotoxic_T_cell | 1.40 | 0.13 | 0.95 | 0.90 | 1.02 | 1.09 | 1.04 | 1.32 | 0.00 | 1.19 | 1.16 | 1.41 | 1.27 | 1.44 | 1.37 | 1.67 | 1.64 | 1.53 | 2.48 | 1.954 |
| NC-Mono\|GZMK+CD8+_cytotoxic_T_cell | 1.39 | 0.14 | 0.95 | 0.87 | 1.04 | 1.02 | 1.03 | 1.25 | 0.00 | 1.15 | 1.16 | 1.43 | 1.26 | 1.44 | 1.37 | 1.69 | 1.60 | 1.53 | 2.56 | 2.063 |
| NC-Mono\|MAI_T | 0.00 | 0.19 | 0.95 | 0.85 | 1.06 | 1.00 | 1.01 | 1.28 | 0.00 | 1.14 | 0.00 | 1.38 | 0.00 | 1.48 | 1.37 | 1.68 | 0.00 | 1.56 | 2.55 | 2.025 |
| NC-Mono\|Memory_B_cell | 0.00 | 0.12 | 1.02 | 0.82 | 0.00 | 0.00 | 1.00 | 1.18 | 0.00 | 1.22 | 1.54 | 1.25 | 0.00 | 1.53 | 1.41 | 1.69 | 1.60 | 1.54 | 2.56 | 2.034 |
| NC-Mono\|NC-Mono | 1.39 | 0.37 | 1.17 | 0.91 | 1.00 | 0.00 | 0.00 | 1.00 | 0.00 | 1.05 | 1.17 | 1.30 | 0.00 | 1.44 | 1.47 | 1.56 | 1.60 | 1.67 | 2.29 | 2.253 |
| NC-Mono\|NKT | 1.41 | 0.12 | 0.96 | 0.94 | 1.14 | 1.15 | 1.05 | 1.39 | 0.00 | 1.25 | 0.00 | 1.50 | 1.30 | 1.48 | 1.37 | 1.71 | 1.68 | 1.54 | 2.42 | 1.942 |
| NC-Mono\|NaÃ¯ve_B_cell | 0.00 | 0.11 | 0.99 | 0.83 | 1.00 | 1.00 | 1.00 | 1.07 | 0.00 | 1.14 | 1.69 | 1.24 | 1.26 | 1.58 | 1.45 | 1.59 | 1.60 | 1.50 | 2.65 | 1.875 |
| NC-Mono\|Plasma | 0.00 | 0.11 | 0.00 | 0.83 | 1.01 | 0.00 | 0.00 | 0.67 | 0.00 | 1.12 | 1.18 | 1.25 | 0.00 | 1.48 | 1.37 | 1.85 | 0.00 | 1.57 | 2.19 | 2.02 |
| NC-Mono\|Treg | 0.00 | 0.11 | 0.95 | 0.83 | 0.00 | 0.00 | 1.00 | 1.26 | 0.00 | 1.22 | 1.16 | 1.41 | 0.00 | 1.41 | 1.38 | 1.69 | 1.60 | 1.54 | 2.49 | 2.157 |
| NC-Mono\|γδ_T | 0.00 | 0.14 | 0.96 | 0.89 | 1.35 | 1.03 | 1.03 | 1.27 | 0.00 | 1.18 | 0.00 | 1.44 | 1.27 | 1.45 | 1.38 | 1.67 | 1.63 | 1.53 | 2.57 | 2.029 |
| NKT\|C-Mono1 | 0.00 | 2.14 | 1.22 | 0.86 | 1.39 | 1.39 | 0.00 | 1.15 | 1.16 | 1.17 | 1.36 | 1.53 | 0.00 | 1.88 | 0.70 | 0.75 | 1.85 | 0.91 | 1.61 | 1.802 |
| NKT\|C-Mono2 | 1.83 | 2.10 | 1.17 | 0.84 | 1.39 | 1.39 | 1.39 | 1.07 | 1.08 | 0.96 | 1.35 | 1.52 | 1.68 | 1.86 | 0.68 | 0.68 | 1.86 | 0.84 | 1.44 | 1.79 |
| NKT\|C-Mono3 | 0.00 | 2.23 | 1.17 | 0.84 | 1.39 | 1.39 | 1.39 | 1.17 | 1.18 | 1.03 | 1.36 | 1.53 | 1.69 | 1.86 | 0.68 | 0.68 | 1.86 | 0.86 | 1.47 | 1.791 |
| NKT\|CD4+_effector_T_cell | 1.83 | 1.79 | 1.10 | 0.83 | 1.39 | 1.39 | 1.39 | 1.46 | 1.46 | 1.27 | 0.00 | 1.61 | 1.69 | 1.89 | 0.54 | 0.87 | 1.86 | 0.71 | 1.95 | 1.397 |
| NKT\|CD4+_memory_T_cell | 1.83 | 1.79 | 1.10 | 0.79 | 1.39 | 1.39 | 1.39 | 1.37 | 1.37 | 1.19 | 1.35 | 1.64 | 1.69 | 1.88 | 0.56 | 0.87 | 1.86 | 0.70 | 1.99 | 1.496 |
| NKT\|CD4+_naÃ¯ve_T_cell | 1.83 | 1.79 | 1.09 | 0.79 | 1.39 | 1.39 | 1.39 | 1.23 | 1.24 | 1.10 | 1.35 | 1.60 | 1.68 | 1.86 | 0.59 | 0.88 | 1.86 | 0.65 | 2.08 | 1.384 |
| NKT\|CD56brightCD16-_NK | 1.85 | 1.89 | 1.11 | 0.89 | 2.10 | 1.65 | 1.46 | 1.41 | 1.41 | 1.22 | 0.00 | 1.59 | 1.69 | 1.89 | 0.53 | 0.90 | 1.90 | 0.76 | 1.90 | 1.545 |
| NKT\|CD56dimCD16+_NK | 1.87 | 1.79 | 1.11 | 0.91 | 1.56 | 1.53 | 1.43 | 1.57 | 1.57 | 1.24 | 1.35 | 1.62 | 1.74 | 1.92 | 0.53 | 0.82 | 1.97 | 0.69 | 1.70 | 1.155 |
| NKT\|CD8+_naÃ¯ve_T_cell | 1.84 | 1.79 | 1.10 | 0.82 | 1.45 | 1.45 | 1.41 | 1.40 | 1.41 | 1.18 | 1.35 | 1.67 | 1.70 | 1.89 | 0.58 | 0.92 | 1.87 | 0.70 | 2.03 | 1.361 |
| NKT\|GNLY+CD8+_cytotoxic_T_cell | 1.84 | 1.80 | 1.10 | 0.86 | 1.41 | 1.47 | 1.43 | 1.46 | 1.46 | 1.23 | 1.35 | 1.60 | 1.70 | 1.88 | 0.53 | 0.83 | 1.89 | 0.69 | 1.90 | 1.305 |
| NKT\|GZMK+CD8+_cytotoxic_T_cell | 1.83 | 1.81 | 1.09 | 0.84 | 1.42 | 1.41 | 1.42 | 1.39 | 1.39 | 1.18 | 1.35 | 1.62 | 1.68 | 1.88 | 0.54 | 0.86 | 1.86 | 0.69 | 1.98 | 1.414 |
| NKT\|MAI_T | 0.00 | 1.87 | 1.10 | 0.82 | 1.44 | 1.39 | 1.40 | 1.42 | 1.42 | 1.18 | 0.00 | 1.58 | 0.00 | 1.92 | 0.53 | 0.84 | 0.00 | 0.73 | 1.98 | 1.376 |
| NKT\|Memory_B_cell | 0.00 | 1.79 | 1.16 | 0.79 | 0.00 | 0.00 | 1.39 | 1.32 | 1.33 | 1.26 | 1.73 | 1.44 | 0.00 | 1.97 | 0.58 | 0.86 | 1.86 | 0.70 | 1.99 | 1.385 |
| NKT\|NC-Mono | 1.83 | 2.04 | 1.31 | 0.87 | 1.39 | 0.00 | 0.00 | 1.14 | 1.15 | 1.09 | 1.36 | 1.50 | 0.00 | 1.88 | 0.64 | 0.73 | 1.86 | 0.84 | 1.72 | 1.605 |
| NKT\|NKT | 1.85 | 1.79 | 1.11 | 0.90 | 1.53 | 1.53 | 1.44 | 1.53 | 1.53 | 1.29 | 0.00 | 1.70 | 1.72 | 1.93 | 0.53 | 0.88 | 1.94 | 0.71 | 1.85 | 1.293 |
| NKT\|NaÃ¯ve_B_cell | 0.00 | 1.79 | 1.13 | 0.79 | 1.39 | 1.39 | 1.39 | 1.22 | 1.22 | 1.17 | 1.88 | 1.43 | 1.69 | 2.02 | 0.62 | 0.76 | 1.85 | 0.66 | 2.07 | 1.226 |
| NKT\|Plasma | 0.00 | 1.79 | 0.00 | 0.79 | 1.40 | 0.00 | 0.00 | 0.82 | 0.82 | 1.16 | 1.37 | 1.45 | 0.00 | 1.93 | 0.53 | 1.02 | 0.00 | 0.73 | 1.61 | 1.371 |
| NKT\|Treg | 0.00 | 1.79 | 1.10 | 0.79 | 0.00 | 0.00 | 1.39 | 1.40 | 1.41 | 1.26 | 1.35 | 1.60 | 0.00 | 1.85 | 0.55 | 0.85 | 1.86 | 0.71 | 1.91 | 1.508 |
| NKT\|γδ_T | 0.00 | 1.81 | 1.10 | 0.85 | 1.73 | 1.42 | 1.41 | 1.41 | 1.41 | 1.22 | 0.00 | 1.63 | 1.69 | 1.89 | 0.54 | 0.83 | 1.89 | 0.70 | 1.99 | 1.381 |
| NaÃ¯ve_B_cell\|C-Mono1 | 0.00 | 0.45 | 1.48 | 0.72 | 1.07 | 1.07 | 0.00 | 1.01 | 1.01 | 0.99 | 0.86 | 1.03 | 0.00 | 1.20 | 2.13 | 2.18 | 1.41 | 2.35 | 1.62 | 0.934 |
| NaÃ¯ve_B_cell\|C-Mono2 | 1.15 | 0.40 | 1.43 | 0.70 | 1.07 | 1.07 | 1.07 | 0.93 | 0.93 | 0.79 | 0.86 | 1.02 | 1.12 | 1.18 | 2.12 | 2.11 | 1.41 | 2.28 | 1.44 | 0.922 |
| NaÃ¯ve_B_cell\|C-Mono3 | 0.00 | 0.53 | 1.44 | 0.71 | 1.07 | 1.07 | 1.07 | 1.03 | 1.03 | 0.85 | 0.86 | 1.04 | 1.12 | 1.18 | 2.11 | 2.11 | 1.41 | 2.30 | 1.48 | 0.924 |
| NaÃ¯ve_B_cell\|CD4+_effector_T_cell | 1.16 | 0.09 | 1.36 | 0.69 | 1.07 | 1.07 | 1.07 | 1.31 | 1.31 | 1.09 | 0.00 | 1.12 | 1.12 | 1.21 | 1.97 | 2.31 | 1.41 | 2.14 | 1.96 | 0.529 |
| NaÃ¯ve_B_cell\|CD4+_memory_T_cell | 1.16 | 0.09 | 1.36 | 0.65 | 1.08 | 1.07 | 1.08 | 1.23 | 1.23 | 1.01 | 0.86 | 1.14 | 1.12 | 1.20 | 2.00 | 2.31 | 1.41 | 2.14 | 1.99 | 0.628 |
| NaÃ¯ve_B_cell\|CD4+_naÃ¯ve_T_cell | 1.16 | 0.09 | 1.36 | 0.65 | 1.07 | 1.07 | 1.07 | 1.09 | 1.09 | 0.93 | 0.86 | 1.11 | 1.12 | 1.19 | 2.02 | 2.31 | 1.41 | 2.09 | 2.09 | 0.516 |
| NaÃ¯ve_B_cell\|CD56brightCD16-_NK | 1.17 | 0.19 | 1.38 | 0.75 | 1.79 | 1.34 | 1.14 | 1.27 | 1.27 | 1.04 | 0.00 | 1.10 | 1.13 | 1.21 | 1.96 | 2.34 | 1.45 | 2.20 | 1.91 | 0.677 |
| NaÃ¯ve_B_cell\|CD56dimCD16+_NK | 1.19 | 0.10 | 1.37 | 0.77 | 1.24 | 1.22 | 1.12 | 1.42 | 1.42 | 1.07 | 0.86 | 1.12 | 1.17 | 1.25 | 1.96 | 2.25 | 1.52 | 2.12 | 1.70 | 0.287 |
| NaÃ¯ve_B_cell\|CD8+_naÃ¯ve_T_cell | 1.16 | 0.09 | 1.36 | 0.69 | 1.13 | 1.14 | 1.09 | 1.26 | 1.26 | 1.00 | 0.86 | 1.17 | 1.13 | 1.21 | 2.01 | 2.36 | 1.43 | 2.14 | 2.03 | 0.493 |
| NaÃ¯ve_B_cell\|GNLY+CD8+_cytotoxic_T_cell | 1.16 | 0.10 | 1.36 | 0.73 | 1.09 | 1.16 | 1.11 | 1.32 | 1.32 | 1.05 | 0.86 | 1.10 | 1.13 | 1.20 | 1.97 | 2.27 | 1.45 | 2.13 | 1.91 | 0.437 |
| NaÃ¯ve_B_cell\|GZMK+CD8+_cytotoxic_T_cell | 1.16 | 0.11 | 1.36 | 0.70 | 1.11 | 1.09 | 1.10 | 1.25 | 1.25 | 1.00 | 0.86 | 1.13 | 1.12 | 1.20 | 1.97 | 2.29 | 1.41 | 2.13 | 1.99 | 0.546 |
| NaÃ¯ve_B_cell\|MAI_T | 0.00 | 0.17 | 1.36 | 0.68 | 1.13 | 1.08 | 1.08 | 1.27 | 1.27 | 1.00 | 0.00 | 1.08 | 0.00 | 1.24 | 1.97 | 2.28 | 0.00 | 2.16 | 1.98 | 0.508 |
| NaÃ¯ve_B_cell\|Memory_B_cell | 0.00 | 0.10 | 1.42 | 0.65 | 0.00 | 0.00 | 1.07 | 1.18 | 1.18 | 1.08 | 1.24 | 0.95 | 0.00 | 1.29 | 2.01 | 2.29 | 1.41 | 2.14 | 1.99 | 0.517 |
| NaÃ¯ve_B_cell\|NC-Mono | 1.16 | 0.35 | 1.57 | 0.74 | 1.07 | 0.00 | 0.00 | 1.00 | 1.00 | 0.91 | 0.87 | 1.00 | 0.00 | 1.20 | 2.07 | 2.16 | 1.41 | 2.27 | 1.72 | 0.737 |
| NaÃ¯ve_B_cell\|NKT | 1.17 | 0.09 | 1.37 | 0.77 | 1.22 | 1.22 | 1.12 | 1.39 | 1.39 | 1.11 | 0.00 | 1.20 | 1.16 | 1.25 | 1.97 | 2.31 | 1.49 | 2.14 | 1.85 | 0.425 |
| NaÃ¯ve_B_cell\|NaÃ¯ve_B_cell | 0.00 | 0.09 | 1.39 | 0.65 | 1.07 | 1.07 | 1.07 | 1.07 | 1.07 | 0.99 | 1.39 | 0.93 | 1.12 | 1.34 | 2.05 | 2.19 | 1.41 | 2.10 | 2.07 | 0.358 |
| NaÃ¯ve_B_cell\|Plasma | 0.00 | 0.09 | 0.00 | 0.66 | 1.09 | 0.00 | 0.00 | 0.67 | 0.67 | 0.98 | 0.88 | 0.95 | 0.00 | 1.25 | 1.97 | 2.45 | 0.00 | 2.17 | 1.62 | 0.503 |
| NaÃ¯ve_B_cell\|Treg | 0.00 | 0.09 | 1.36 | 0.66 | 0.00 | 0.00 | 1.07 | 1.26 | 1.26 | 1.08 | 0.86 | 1.11 | 0.00 | 1.17 | 1.98 | 2.29 | 1.41 | 2.14 | 1.92 | 0.64 |
| NaÃ¯ve_B_cell\|γδ_T | 0.00 | 0.11 | 1.36 | 0.71 | 1.42 | 1.10 | 1.10 | 1.26 | 1.27 | 1.04 | 0.00 | 1.13 | 1.13 | 1.22 | 1.98 | 2.27 | 1.44 | 2.13 | 2.00 | 0.513 |
| Plasma\|C-Mono1 | 0.00 | 0.57 | 0.66 | 0.50 | 0.67 | 0.67 | 0.00 | 1.02 | 0.00 | 1.08 | 0.46 | 0.63 | 0.00 | 1.14 | 0.94 | 0.99 | 1.38 | 1.15 | 0.00 | 1.03 |
| Plasma\|C-Mono2 | 1.10 | 0.52 | 0.61 | 0.47 | 0.67 | 0.67 | 0.67 | 0.94 | 0.00 | 0.87 | 0.45 | 0.62 | 1.17 | 1.12 | 0.92 | 0.92 | 1.38 | 1.08 | 0.00 | 1.017 |
| Plasma\|C-Mono3 | 0.00 | 0.65 | 0.62 | 0.48 | 0.67 | 0.67 | 0.67 | 1.04 | 0.00 | 0.94 | 0.46 | 0.63 | 1.17 | 1.12 | 0.92 | 0.92 | 1.38 | 1.10 | 0.00 | 1.019 |
| Plasma\|CD4+_effector_T_cell | 1.10 | 0.21 | 0.54 | 0.47 | 0.67 | 0.67 | 0.67 | 1.33 | 0.00 | 1.18 | 0.00 | 0.71 | 1.17 | 1.15 | 0.78 | 1.11 | 1.39 | 0.95 | 0.00 | 0.625 |
| Plasma\|CD4+_memory_T_cell | 1.10 | 0.21 | 0.54 | 0.42 | 0.68 | 0.67 | 0.67 | 1.24 | 0.00 | 1.09 | 0.45 | 0.74 | 1.17 | 1.14 | 0.80 | 1.11 | 1.39 | 0.94 | 0.00 | 0.723 |
| Plasma\|CD4+_naÃ¯ve_T_cell | 1.10 | 0.21 | 0.54 | 0.42 | 0.67 | 0.67 | 0.67 | 1.10 | 0.00 | 1.01 | 0.45 | 0.70 | 1.17 | 1.13 | 0.83 | 1.12 | 1.39 | 0.89 | 0.00 | 0.611 |
| Plasma\|CD56brightCD16-_NK | 1.11 | 0.31 | 0.56 | 0.53 | 1.39 | 0.94 | 0.74 | 1.28 | 0.00 | 1.13 | 0.00 | 0.69 | 1.18 | 1.15 | 0.77 | 1.14 | 1.43 | 1.00 | 0.00 | 0.772 |
| Plasma\|CD56dimCD16+_NK | 1.13 | 0.22 | 0.55 | 0.54 | 0.84 | 0.82 | 0.72 | 1.44 | 0.00 | 1.15 | 0.45 | 0.72 | 1.22 | 1.19 | 0.77 | 1.06 | 1.50 | 0.93 | 0.00 | 0.382 |
| Plasma\|CD8+_naÃ¯ve_T_cell | 1.10 | 0.21 | 0.54 | 0.46 | 0.73 | 0.74 | 0.69 | 1.27 | 0.00 | 1.09 | 0.45 | 0.77 | 1.18 | 1.15 | 0.82 | 1.16 | 1.40 | 0.94 | 0.00 | 0.588 |
| Plasma\|GNLY+CD8+_cytotoxic_T_cell | 1.10 | 0.22 | 0.54 | 0.50 | 0.69 | 0.76 | 0.71 | 1.33 | 0.00 | 1.14 | 0.45 | 0.70 | 1.18 | 1.15 | 0.77 | 1.07 | 1.42 | 0.93 | 0.00 | 0.532 |
| Plasma\|GZMK+CD8+_cytotoxic_T_cell | 1.10 | 0.23 | 0.54 | 0.47 | 0.71 | 0.69 | 0.70 | 1.26 | 0.00 | 1.09 | 0.45 | 0.72 | 1.17 | 1.14 | 0.78 | 1.10 | 1.39 | 0.93 | 0.00 | 0.641 |
| Plasma\|MAI_T | 0.00 | 0.29 | 0.54 | 0.45 | 0.73 | 0.68 | 0.68 | 1.29 | 0.00 | 1.09 | 0.00 | 0.68 | 0.00 | 1.18 | 0.77 | 1.08 | 0.00 | 0.97 | 0.00 | 0.604 |
| Plasma\|Memory_B_cell | 0.00 | 0.22 | 0.61 | 0.42 | 0.00 | 0.00 | 0.67 | 1.19 | 0.00 | 1.17 | 0.83 | 0.54 | 0.00 | 1.24 | 0.82 | 1.10 | 1.39 | 0.94 | 0.00 | 0.612 |
| Plasma\|NC-Mono | 1.10 | 0.47 | 0.76 | 0.51 | 0.67 | 0.00 | 0.00 | 1.01 | 0.00 | 1.00 | 0.46 | 0.60 | 0.00 | 1.14 | 0.88 | 0.97 | 1.39 | 1.08 | 0.00 | 0.832 |
| Plasma\|NKT | 1.11 | 0.21 | 0.55 | 0.54 | 0.82 | 0.82 | 0.72 | 1.40 | 0.00 | 1.20 | 0.00 | 0.80 | 1.21 | 1.19 | 0.77 | 1.12 | 1.46 | 0.95 | 0.00 | 0.52 |
| Plasma\|NaÃ¯ve_B_cell | 0.00 | 0.21 | 0.57 | 0.43 | 0.67 | 0.67 | 0.67 | 1.09 | 0.00 | 1.08 | 0.98 | 0.53 | 1.17 | 1.28 | 0.86 | 1.00 | 1.38 | 0.90 | 0.00 | 0.453 |
| Plasma\|Plasma | 0.00 | 0.21 | 0.00 | 0.43 | 0.68 | 0.00 | 0.00 | 0.68 | 0.00 | 1.07 | 0.47 | 0.55 | 0.00 | 1.19 | 0.77 | 1.26 | 0.00 | 0.97 | 0.00 | 0.598 |
| Plasma\|Treg | 0.00 | 0.21 | 0.54 | 0.43 | 0.00 | 0.00 | 0.67 | 1.27 | 0.00 | 1.17 | 0.45 | 0.70 | 0.00 | 1.12 | 0.79 | 1.09 | 1.38 | 0.95 | 0.00 | 0.735 |
| Plasma\|γδ_T | 0.00 | 0.23 | 0.55 | 0.48 | 1.02 | 0.70 | 0.70 | 1.28 | 0.00 | 1.13 | 0.00 | 0.73 | 1.18 | 1.16 | 0.78 | 1.07 | 1.42 | 0.94 | 0.00 | 0.608 |
| Treg\|C-Mono1 | 0.00 | 0.47 | 1.54 | 0.83 | 1.26 | 1.26 | 0.00 | 0.00 | 0.00 | 1.04 | 1.17 | 1.34 | 0.00 | 1.62 | 0.72 | 0.77 | 1.61 | 0.93 | 1.61 | 0.968 |
| Treg\|C-Mono2 | 1.57 | 0.43 | 1.49 | 0.81 | 1.26 | 1.26 | 1.26 | 0.00 | 0.00 | 0.84 | 1.17 | 1.33 | 1.59 | 1.60 | 0.70 | 0.70 | 1.61 | 0.86 | 1.44 | 0.956 |
| Treg\|C-Mono3 | 0.00 | 0.55 | 1.50 | 0.81 | 1.26 | 1.26 | 1.26 | 0.00 | 0.00 | 0.91 | 1.17 | 1.35 | 1.59 | 1.60 | 0.70 | 0.70 | 1.61 | 0.88 | 1.47 | 0.958 |
| Treg\|CD4+_effector_T_cell | 1.57 | 0.11 | 1.42 | 0.80 | 1.26 | 1.26 | 1.26 | 0.00 | 0.00 | 1.15 | 0.00 | 1.42 | 1.59 | 1.63 | 0.56 | 0.89 | 1.61 | 0.73 | 1.95 | 0.563 |
| Treg\|CD4+_memory_T_cell | 1.57 | 0.11 | 1.42 | 0.76 | 1.26 | 1.26 | 1.26 | 0.00 | 0.00 | 1.06 | 1.16 | 1.45 | 1.59 | 1.62 | 0.58 | 0.89 | 1.61 | 0.72 | 1.98 | 0.662 |
| Treg\|CD4+_naÃ¯ve_T_cell | 1.57 | 0.11 | 1.42 | 0.76 | 1.26 | 1.26 | 1.26 | 0.00 | 0.00 | 0.98 | 1.16 | 1.41 | 1.59 | 1.60 | 0.61 | 0.90 | 1.61 | 0.67 | 2.08 | 0.55 |
| Treg\|CD56brightCD16-_NK | 1.59 | 0.21 | 1.44 | 0.86 | 1.98 | 1.53 | 1.33 | 0.00 | 0.00 | 1.10 | 0.00 | 1.41 | 1.60 | 1.63 | 0.55 | 0.92 | 1.65 | 0.78 | 1.90 | 0.711 |
| Treg\|CD56dimCD16+_NK | 1.61 | 0.12 | 1.43 | 0.88 | 1.43 | 1.41 | 1.31 | 0.00 | 0.00 | 1.12 | 1.16 | 1.43 | 1.64 | 1.66 | 0.55 | 0.84 | 1.72 | 0.71 | 1.70 | 0.321 |
| Treg\|CD8+_naÃ¯ve_T_cell | 1.58 | 0.11 | 1.42 | 0.79 | 1.32 | 1.33 | 1.28 | 0.00 | 0.00 | 1.06 | 1.16 | 1.48 | 1.60 | 1.63 | 0.60 | 0.94 | 1.62 | 0.72 | 2.03 | 0.527 |
| Treg\|GNLY+CD8+_cytotoxic_T_cell | 1.58 | 0.12 | 1.42 | 0.83 | 1.28 | 1.35 | 1.30 | 0.00 | 0.00 | 1.10 | 1.16 | 1.41 | 1.60 | 1.62 | 0.55 | 0.85 | 1.65 | 0.71 | 1.90 | 0.471 |
| Treg\|GZMK+CD8+_cytotoxic_T_cell | 1.57 | 0.13 | 1.42 | 0.81 | 1.30 | 1.28 | 1.29 | 0.00 | 0.00 | 1.06 | 1.16 | 1.44 | 1.59 | 1.62 | 0.56 | 0.88 | 1.61 | 0.71 | 1.98 | 0.58 |
| Treg\|MAI_T | 0.00 | 0.19 | 1.42 | 0.79 | 1.32 | 1.26 | 1.27 | 0.00 | 0.00 | 1.05 | 0.00 | 1.39 | 0.00 | 1.66 | 0.55 | 0.86 | 0.00 | 0.75 | 1.97 | 0.543 |
| Treg\|Memory_B_cell | 0.00 | 0.12 | 1.49 | 0.76 | 0.00 | 0.00 | 1.26 | 0.00 | 0.00 | 1.14 | 1.55 | 1.26 | 0.00 | 1.71 | 0.60 | 0.88 | 1.61 | 0.72 | 1.99 | 0.551 |
| Treg\|NC-Mono | 1.57 | 0.37 | 1.63 | 0.84 | 1.26 | 0.00 | 0.00 | 0.00 | 0.00 | 0.97 | 1.17 | 1.31 | 0.00 | 1.62 | 0.66 | 0.75 | 1.61 | 0.86 | 1.71 | 0.771 |
| Treg\|NKT | 1.59 | 0.11 | 1.43 | 0.87 | 1.40 | 1.41 | 1.31 | 0.00 | 0.00 | 1.17 | 0.00 | 1.51 | 1.63 | 1.66 | 0.55 | 0.90 | 1.69 | 0.73 | 1.85 | 0.459 |
| Treg\|NaÃ¯ve_B_cell | 0.00 | 0.11 | 1.45 | 0.76 | 1.26 | 1.26 | 1.26 | 0.00 | 0.00 | 1.05 | 1.69 | 1.24 | 1.59 | 1.76 | 0.64 | 0.78 | 1.61 | 0.68 | 2.07 | 0.392 |
| Treg\|Plasma | 0.00 | 0.11 | 0.00 | 0.76 | 1.27 | 0.00 | 0.00 | 0.00 | 0.00 | 1.03 | 1.18 | 1.26 | 0.00 | 1.67 | 0.55 | 1.04 | 0.00 | 0.75 | 1.61 | 0.537 |
| Treg\|Treg | 0.00 | 0.11 | 1.42 | 0.76 | 0.00 | 0.00 | 1.26 | 0.00 | 0.00 | 1.14 | 1.16 | 1.41 | 0.00 | 1.59 | 0.57 | 0.87 | 1.61 | 0.73 | 1.91 | 0.674 |
| Treg\|γδ_T | 0.00 | 0.13 | 1.42 | 0.82 | 1.61 | 1.29 | 1.29 | 0.00 | 0.00 | 1.10 | 0.00 | 1.44 | 1.60 | 1.63 | 0.56 | 0.85 | 1.64 | 0.72 | 1.99 | 0.547 |
| γδ_T\|C-Mono1 | 0.00 | 2.13 | 1.38 | 0.84 | 1.27 | 1.26 | 0.00 | 1.36 | 1.04 | 1.08 | 1.31 | 1.48 | 0.00 | 1.70 | 0.79 | 0.84 | 1.67 | 1.01 | 1.62 | 1.298 |
| γδ_T\|C-Mono2 | 1.65 | 2.09 | 1.33 | 0.81 | 1.27 | 1.27 | 1.26 | 1.28 | 0.96 | 0.88 | 1.31 | 1.47 | 1.53 | 1.68 | 0.78 | 0.77 | 1.67 | 0.94 | 1.44 | 1.286 |
| γδ_T\|C-Mono3 | 0.00 | 2.21 | 1.33 | 0.82 | 1.27 | 1.27 | 1.27 | 1.38 | 1.06 | 0.95 | 1.31 | 1.49 | 1.53 | 1.68 | 0.77 | 0.78 | 1.67 | 0.96 | 1.48 | 1.287 |
| γδ_T\|CD4+_effector_T_cell | 1.65 | 1.77 | 1.25 | 0.81 | 1.27 | 1.27 | 1.27 | 1.66 | 1.34 | 1.19 | 0.00 | 1.57 | 1.53 | 1.71 | 0.63 | 0.97 | 1.67 | 0.81 | 1.96 | 0.893 |
| γδ_T\|CD4+_memory_T_cell | 1.65 | 1.77 | 1.26 | 0.76 | 1.27 | 1.26 | 1.27 | 1.57 | 1.26 | 1.10 | 1.31 | 1.59 | 1.53 | 1.70 | 0.66 | 0.97 | 1.67 | 0.80 | 1.99 | 0.992 |
| γδ_T\|CD4+_naÃ¯ve_T_cell | 1.65 | 1.77 | 1.25 | 0.76 | 1.27 | 1.26 | 1.26 | 1.44 | 1.12 | 1.02 | 1.31 | 1.56 | 1.53 | 1.68 | 0.69 | 0.98 | 1.67 | 0.75 | 2.09 | 0.88 |
| γδ_T\|CD56brightCD16-_NK | 1.67 | 1.88 | 1.27 | 0.87 | 1.98 | 1.53 | 1.34 | 1.61 | 1.30 | 1.14 | 0.00 | 1.55 | 1.54 | 1.71 | 0.63 | 1.00 | 1.71 | 0.86 | 1.91 | 1.04 |
| γδ_T\|CD56dimCD16+_NK | 1.69 | 1.78 | 1.27 | 0.88 | 1.44 | 1.41 | 1.31 | 1.77 | 1.45 | 1.16 | 1.31 | 1.57 | 1.59 | 1.74 | 0.63 | 0.91 | 1.78 | 0.78 | 1.70 | 0.65 |
| γδ_T\|CD8+_naÃ¯ve_T_cell | 1.66 | 1.78 | 1.25 | 0.80 | 1.32 | 1.33 | 1.28 | 1.61 | 1.29 | 1.09 | 1.31 | 1.62 | 1.55 | 1.71 | 0.68 | 1.02 | 1.68 | 0.80 | 2.03 | 0.857 |
| γδ_T\|GNLY+CD8+_cytotoxic_T_cell | 1.66 | 1.78 | 1.26 | 0.84 | 1.28 | 1.35 | 1.31 | 1.66 | 1.35 | 1.14 | 1.31 | 1.55 | 1.55 | 1.70 | 0.63 | 0.93 | 1.71 | 0.79 | 1.91 | 0.801 |
| γδ_T\|GZMK+CD8+_cytotoxic_T_cell | 1.65 | 1.79 | 1.25 | 0.81 | 1.30 | 1.28 | 1.29 | 1.59 | 1.28 | 1.10 | 1.31 | 1.58 | 1.53 | 1.70 | 0.63 | 0.96 | 1.67 | 0.79 | 1.99 | 0.91 |
| γδ_T\|MAI_T | 0.00 | 1.85 | 1.25 | 0.79 | 1.32 | 1.27 | 1.27 | 1.62 | 1.30 | 1.09 | 0.00 | 1.53 | 0.00 | 1.74 | 0.63 | 0.94 | 0.00 | 0.82 | 1.98 | 0.872 |
| γδ_T\|Memory_B_cell | 0.00 | 1.78 | 1.32 | 0.76 | 0.00 | 0.00 | 1.27 | 1.53 | 1.21 | 1.18 | 1.69 | 1.40 | 0.00 | 1.79 | 0.67 | 0.95 | 1.67 | 0.80 | 1.99 | 0.881 |
| γδ_T\|NC-Mono | 1.65 | 2.03 | 1.47 | 0.85 | 1.27 | 0.00 | 0.00 | 1.35 | 1.03 | 1.00 | 1.32 | 1.45 | 0.00 | 1.70 | 0.73 | 0.83 | 1.67 | 0.93 | 1.72 | 1.101 |
| γδ_T\|NKT | 1.67 | 1.78 | 1.26 | 0.88 | 1.41 | 1.41 | 1.31 | 1.73 | 1.42 | 1.20 | 0.00 | 1.65 | 1.57 | 1.75 | 0.63 | 0.97 | 1.75 | 0.80 | 1.85 | 0.789 |
| γδ_T\|NaÃ¯ve_B_cell | 0.00 | 1.77 | 1.29 | 0.77 | 1.26 | 1.27 | 1.27 | 1.42 | 1.10 | 1.09 | 1.84 | 1.38 | 1.53 | 1.84 | 0.72 | 0.85 | 1.67 | 0.76 | 2.07 | 0.722 |
| γδ_T\|Plasma | 0.00 | 1.77 | 0.00 | 0.77 | 1.28 | 0.00 | 0.00 | 1.02 | 0.70 | 1.07 | 1.33 | 1.40 | 0.00 | 1.75 | 0.63 | 1.12 | 0.00 | 0.83 | 1.62 | 0.867 |
| γδ_T\|Treg | 0.00 | 1.77 | 1.25 | 0.77 | 0.00 | 0.00 | 1.26 | 1.61 | 1.29 | 1.17 | 1.31 | 1.56 | 0.00 | 1.67 | 0.64 | 0.95 | 1.67 | 0.81 | 1.92 | 1.004 |
| γδ_T\|γδ_T | 0.00 | 1.80 | 1.26 | 0.82 | 1.61 | 1.29 | 1.29 | 1.61 | 1.29 | 1.13 | 0.00 | 1.59 | 1.54 | 1.71 | 0.64 | 0.93 | 1.70 | 0.80 | 2.00 | 0.877 |
