# Supplementary material for: Decoding the immune landscape following hip fracture in elderly patients: unveiling temporal dynamics through single-cell RNA sequencing
Source: Immun Ageing. 2023 Oct 17;20:54. doi: 10.1186/s12979-023-00380-6 (PMC10580557; doi:10.1186/s12979-023-00380-6)
Supplement: Supplementary file 16 — Supplementary Material 16 [file 12979_2023_380_MOESM16_ESM.docx]

**Supplementary Table 15.** Top 20 (p-value) interacting pair (7-d post-surgery)

| **interacting pair** | **CCL5_CCR5** | **CCL5_CCR1** | **TYROBP_CD44** | **HLA-C_KIR2DL3** | **HLA-C_FAM3C** | **CD94:NKG2A_HLA-E** | **CD94:NKG2C_HLA-E** | **CD74_MIF** | **CD74_COPA** | **CD74_APP** | **HLA-B_KIR3DL2** | **PTPRC_SEMA4D** | **PTPRC_CD22** | **CD48_CD244** | **HLA-E_KLRC1** | **HLA-E_KLRC2** | **HLA-E_KLRK1** | **C5AR1_RPS19** | **CD52_SIGLEC10** | **BSG_PPIA** |
| --- | --- | --- | --- | --- | --- | --- | --- | --- | --- | --- | --- | --- | --- | --- | --- | --- | --- | --- | --- | --- |
| C-Mono1\|C-Mono1 | 1.00 | 1.00 | 0.00 | 1.00 | 1.00 | 1.00 | 1.00 | 0.00 | 0.00 | 0.00 | 1.00 | 0.97 | 1.00 | 0.00 | 1.00 | 1.00 | 1.00 | 1.00 | 1.00 | 0.00 |
| C-Mono1\|C-Mono2 | 1.00 | 1.00 | 0.00 | 1.00 | 1.00 | 1.00 | 1.00 | 0.00 | 0.00 | 0.00 | 1.00 | 1.00 | 1.00 | 1.00 | 1.00 | 1.00 | 1.00 | 1.00 | 1.00 | 1.00 |
| C-Mono1\|C-Mono3 | 1.00 | 1.00 | 0.00 | 1.00 | 1.00 | 1.00 | 1.00 | 0.00 | 0.00 | 0.00 | 1.00 | 0.60 | 1.00 | 0.00 | 1.00 | 1.00 | 1.00 | 1.00 | 1.00 | 1.00 |
| C-Mono1\|CD4+_effector_T_cell | 1.00 | 1.00 | 0.00 | 1.00 | 1.00 | 1.00 | 1.00 | 0.00 | 0.00 | 1.00 | 1.00 | 0.00 | 1.00 | 1.00 | 1.00 | 1.00 | 1.00 | 0.00 | 1.00 | 0.00 |
| C-Mono1\|CD4+_memory_T_cell | 1.00 | 1.00 | 0.00 | 1.00 | 1.00 | 1.00 | 1.00 | 0.00 | 0.00 | 1.00 | 1.00 | 0.00 | 1.00 | 1.00 | 1.00 | 1.00 | 1.00 | 0.00 | 1.00 | 0.00 |
| C-Mono1\|CD4+_naÃ¯ve_T_cell | 1.00 | 1.00 | 0.00 | 1.00 | 1.00 | 1.00 | 1.00 | 0.00 | 0.00 | 0.00 | 1.00 | 0.00 | 1.00 | 1.00 | 1.00 | 1.00 | 1.00 | 0.00 | 1.00 | 0.00 |
| C-Mono1\|CD56brightCD16-_NK | 1.00 | 1.00 | 0.00 | 1.00 | 1.00 | 1.00 | 1.00 | 0.00 | 0.00 | 1.00 | 1.00 | 0.06 | 1.00 | 0.00 | 0.00 | 0.00 | 1.00 | 0.00 | 1.00 | 0.00 |
| C-Mono1\|CD56dimCD16+_NK | 1.00 | 1.00 | 0.00 | 1.00 | 1.00 | 1.00 | 1.00 | 0.00 | 0.00 | 1.00 | 0.00 | 0.00 | 1.00 | 0.00 | 0.00 | 1.00 | 1.00 | 0.00 | 1.00 | 0.00 |
| C-Mono1\|CD8+_naÃ¯ve_T_cell | 1.00 | 1.00 | 0.00 | 1.00 | 1.00 | 1.00 | 1.00 | 0.00 | 0.00 | 1.00 | 1.00 | 0.00 | 1.00 | 0.06 | 1.00 | 1.00 | 1.00 | 0.00 | 1.00 | 0.00 |
| C-Mono1\|GNLY+CD8+_cytotoxic_T_cell | 1.00 | 1.00 | 0.00 | 1.00 | 1.00 | 1.00 | 1.00 | 0.00 | 0.00 | 1.00 | 1.00 | 0.00 | 1.00 | 0.00 | 1.00 | 1.00 | 1.00 | 0.00 | 1.00 | 0.00 |
| C-Mono1\|GZMK+CD8+_cytotoxic_T_cell | 1.00 | 1.00 | 0.00 | 1.00 | 1.00 | 1.00 | 1.00 | 0.00 | 0.00 | 1.00 | 1.00 | 0.00 | 1.00 | 1.00 | 1.00 | 1.00 | 1.00 | 0.00 | 1.00 | 0.00 |
| C-Mono1\|MAI_T | 1.00 | 1.00 | 0.00 | 1.00 | 1.00 | 1.00 | 1.00 | 0.00 | 0.00 | 1.00 | 1.00 | 0.66 | 1.00 | 1.00 | 1.00 | 1.00 | 1.00 | 0.00 | 1.00 | 0.00 |
| C-Mono1\|Memory_B_cell | 1.00 | 1.00 | 0.00 | 1.00 | 1.00 | 1.00 | 1.00 | 0.00 | 0.00 | 1.00 | 1.00 | 1.00 | 0.00 | 1.00 | 1.00 | 1.00 | 1.00 | 0.00 | 1.00 | 0.00 |
| C-Mono1\|NC-Mono | 1.00 | 1.00 | 0.00 | 1.00 | 1.00 | 1.00 | 1.00 | 0.00 | 0.00 | 0.00 | 1.00 | 1.00 | 1.00 | 0.00 | 1.00 | 1.00 | 1.00 | 0.00 | 0.00 | 0.00 |
| C-Mono1\|NKT | 1.00 | 1.00 | 0.00 | 1.00 | 1.00 | 1.00 | 1.00 | 0.00 | 0.00 | 1.00 | 0.06 | 0.00 | 1.00 | 0.00 | 1.00 | 1.00 | 1.00 | 0.00 | 1.00 | 0.00 |
| C-Mono1\|NaÃ¯ve_B_cell | 1.00 | 1.00 | 0.00 | 1.00 | 1.00 | 1.00 | 1.00 | 0.00 | 0.00 | 0.00 | 1.00 | 1.00 | 0.00 | 1.00 | 1.00 | 1.00 | 1.00 | 0.00 | 1.00 | 0.00 |
| C-Mono1\|Plasma | 1.00 | 1.00 | 0.00 | 1.00 | 0.85 | 1.00 | 1.00 | 0.00 | 0.00 | 0.00 | 1.00 | 1.00 | 1.00 | 1.00 | 1.00 | 1.00 | 1.00 | 0.00 | 1.00 | 0.97 |
| C-Mono1\|Treg | 1.00 | 1.00 | 0.00 | 1.00 | 1.00 | 1.00 | 1.00 | 0.00 | 0.00 | 1.00 | 1.00 | 0.03 | 1.00 | 1.00 | 1.00 | 1.00 | 1.00 | 0.00 | 1.00 | 0.00 |
| C-Mono1\|γδ_T | 1.00 | 1.00 | 0.00 | 1.00 | 1.00 | 1.00 | 1.00 | 0.00 | 0.00 | 1.00 | 1.00 | 0.00 | 1.00 | 1.00 | 0.00 | 1.00 | 1.00 | 0.00 | 1.00 | 0.00 |
| C-Mono2\|C-Mono1 | 1.00 | 1.00 | 0.00 | 1.00 | 1.00 | 1.00 | 1.00 | 0.00 | 0.00 | 0.00 | 1.00 | 1.00 | 1.00 | 1.00 | 1.00 | 1.00 | 1.00 | 0.97 | 1.00 | 0.16 |
| C-Mono2\|C-Mono2 | 1.00 | 1.00 | 0.00 | 1.00 | 1.00 | 1.00 | 1.00 | 0.00 | 0.00 | 0.00 | 1.00 | 1.00 | 1.00 | 1.00 | 1.00 | 1.00 | 1.00 | 1.00 | 1.00 | 1.00 |
| C-Mono2\|C-Mono3 | 1.00 | 1.00 | 0.00 | 1.00 | 1.00 | 1.00 | 1.00 | 0.00 | 0.00 | 0.00 | 1.00 | 1.00 | 1.00 | 1.00 | 1.00 | 1.00 | 1.00 | 1.00 | 1.00 | 1.00 |
| C-Mono2\|CD4+_effector_T_cell | 1.00 | 1.00 | 0.00 | 1.00 | 1.00 | 1.00 | 1.00 | 0.00 | 0.00 | 1.00 | 1.00 | 0.19 | 1.00 | 1.00 | 1.00 | 1.00 | 1.00 | 0.00 | 1.00 | 0.00 |
| C-Mono2\|CD4+_memory_T_cell | 1.00 | 1.00 | 0.00 | 1.00 | 1.00 | 1.00 | 1.00 | 0.00 | 0.00 | 1.00 | 1.00 | 0.00 | 1.00 | 1.00 | 1.00 | 1.00 | 1.00 | 0.00 | 1.00 | 0.00 |
| C-Mono2\|CD4+_naÃ¯ve_T_cell | 1.00 | 1.00 | 0.00 | 1.00 | 1.00 | 1.00 | 1.00 | 0.00 | 0.00 | 0.00 | 1.00 | 0.00 | 1.00 | 1.00 | 1.00 | 1.00 | 1.00 | 0.00 | 1.00 | 0.88 |
| C-Mono2\|CD56brightCD16-_NK | 1.00 | 1.00 | 0.00 | 1.00 | 1.00 | 1.00 | 1.00 | 0.00 | 0.00 | 1.00 | 1.00 | 0.29 | 1.00 | 0.85 | 0.00 | 1.00 | 1.00 | 0.00 | 1.00 | 0.00 |
| C-Mono2\|CD56dimCD16+_NK | 1.00 | 1.00 | 0.00 | 1.00 | 1.00 | 1.00 | 1.00 | 0.00 | 0.00 | 1.00 | 1.00 | 0.00 | 1.00 | 0.70 | 1.00 | 1.00 | 1.00 | 0.00 | 1.00 | 0.00 |
| C-Mono2\|CD8+_naÃ¯ve_T_cell | 1.00 | 1.00 | 0.00 | 1.00 | 1.00 | 1.00 | 1.00 | 0.00 | 0.00 | 1.00 | 1.00 | 0.00 | 1.00 | 1.00 | 1.00 | 1.00 | 1.00 | 0.00 | 1.00 | 0.00 |
| C-Mono2\|GNLY+CD8+_cytotoxic_T_cell | 1.00 | 1.00 | 0.00 | 1.00 | 1.00 | 1.00 | 1.00 | 0.00 | 0.00 | 1.00 | 1.00 | 0.00 | 1.00 | 1.00 | 1.00 | 1.00 | 1.00 | 0.00 | 1.00 | 0.00 |
| C-Mono2\|GZMK+CD8+_cytotoxic_T_cell | 1.00 | 1.00 | 0.00 | 1.00 | 1.00 | 1.00 | 1.00 | 0.00 | 0.00 | 1.00 | 1.00 | 0.03 | 1.00 | 1.00 | 1.00 | 1.00 | 1.00 | 0.00 | 1.00 | 0.00 |
| C-Mono2\|MAI_T | 1.00 | 1.00 | 0.00 | 1.00 | 1.00 | 1.00 | 1.00 | 0.00 | 0.00 | 1.00 | 1.00 | 1.00 | 1.00 | 1.00 | 1.00 | 1.00 | 1.00 | 0.00 | 1.00 | 0.00 |
| C-Mono2\|Memory_B_cell | 1.00 | 1.00 | 0.00 | 1.00 | 1.00 | 1.00 | 1.00 | 0.00 | 0.00 | 1.00 | 1.00 | 1.00 | 0.00 | 1.00 | 1.00 | 1.00 | 1.00 | 0.00 | 1.00 | 0.00 |
| C-Mono2\|NC-Mono | 1.00 | 1.00 | 0.00 | 1.00 | 1.00 | 1.00 | 1.00 | 0.00 | 0.00 | 0.00 | 1.00 | 1.00 | 1.00 | 1.00 | 1.00 | 1.00 | 1.00 | 0.00 | 0.00 | 0.09 |
| C-Mono2\|NKT | 1.00 | 1.00 | 0.00 | 1.00 | 1.00 | 1.00 | 1.00 | 0.00 | 0.00 | 1.00 | 1.00 | 0.00 | 1.00 | 0.59 | 1.00 | 1.00 | 1.00 | 0.00 | 1.00 | 0.00 |
| C-Mono2\|NaÃ¯ve_B_cell | 1.00 | 1.00 | 0.00 | 1.00 | 1.00 | 1.00 | 1.00 | 0.00 | 0.00 | 0.00 | 1.00 | 1.00 | 0.00 | 1.00 | 1.00 | 1.00 | 1.00 | 0.00 | 1.00 | 0.00 |
| C-Mono2\|Plasma | 1.00 | 1.00 | 0.00 | 1.00 | 1.00 | 1.00 | 1.00 | 0.00 | 0.00 | 0.00 | 1.00 | 1.00 | 1.00 | 1.00 | 1.00 | 1.00 | 1.00 | 0.00 | 1.00 | 1.00 |
| C-Mono2\|Treg | 1.00 | 1.00 | 0.00 | 1.00 | 1.00 | 1.00 | 1.00 | 0.00 | 0.00 | 1.00 | 1.00 | 0.60 | 1.00 | 1.00 | 1.00 | 1.00 | 1.00 | 0.00 | 1.00 | 0.00 |
| C-Mono2\|γδ_T | 1.00 | 1.00 | 0.00 | 1.00 | 1.00 | 1.00 | 1.00 | 0.00 | 0.00 | 1.00 | 1.00 | 0.00 | 1.00 | 1.00 | 0.00 | 1.00 | 1.00 | 0.00 | 1.00 | 0.00 |
| C-Mono3\|C-Mono1 | 1.00 | 1.00 | 0.00 | 1.00 | 1.00 | 1.00 | 1.00 | 0.00 | 0.00 | 0.00 | 1.00 | 0.94 | 1.00 | 0.81 | 1.00 | 1.00 | 1.00 | 0.37 | 1.00 | 0.13 |
| C-Mono3\|C-Mono2 | 1.00 | 1.00 | 0.00 | 1.00 | 1.00 | 1.00 | 1.00 | 0.00 | 0.00 | 0.00 | 1.00 | 1.00 | 1.00 | 1.00 | 1.00 | 1.00 | 1.00 | 1.00 | 1.00 | 1.00 |
| C-Mono3\|C-Mono3 | 1.00 | 1.00 | 0.00 | 1.00 | 1.00 | 1.00 | 1.00 | 0.00 | 0.00 | 0.00 | 1.00 | 0.56 | 1.00 | 1.00 | 1.00 | 1.00 | 1.00 | 1.00 | 1.00 | 1.00 |
| C-Mono3\|CD4+_effector_T_cell | 1.00 | 1.00 | 0.00 | 1.00 | 1.00 | 1.00 | 1.00 | 0.00 | 0.00 | 1.00 | 1.00 | 0.00 | 1.00 | 1.00 | 1.00 | 1.00 | 1.00 | 0.00 | 1.00 | 0.00 |
| C-Mono3\|CD4+_memory_T_cell | 1.00 | 1.00 | 0.00 | 1.00 | 1.00 | 1.00 | 1.00 | 0.00 | 0.00 | 1.00 | 1.00 | 0.00 | 1.00 | 1.00 | 1.00 | 1.00 | 1.00 | 0.00 | 1.00 | 0.00 |
| C-Mono3\|CD4+_naÃ¯ve_T_cell | 1.00 | 1.00 | 0.00 | 1.00 | 1.00 | 1.00 | 1.00 | 0.00 | 0.00 | 0.00 | 1.00 | 0.00 | 1.00 | 1.00 | 1.00 | 1.00 | 1.00 | 0.00 | 1.00 | 0.43 |
| C-Mono3\|CD56brightCD16-_NK | 1.00 | 1.00 | 0.00 | 1.00 | 1.00 | 1.00 | 1.00 | 0.00 | 0.00 | 1.00 | 1.00 | 0.03 | 1.00 | 0.06 | 0.00 | 0.00 | 1.00 | 0.00 | 1.00 | 0.00 |
| C-Mono3\|CD56dimCD16+_NK | 1.00 | 1.00 | 0.00 | 1.00 | 1.00 | 1.00 | 1.00 | 0.00 | 0.00 | 1.00 | 0.00 | 0.00 | 1.00 | 0.00 | 0.00 | 0.00 | 1.00 | 0.00 | 1.00 | 0.00 |
| C-Mono3\|CD8+_naÃ¯ve_T_cell | 1.00 | 1.00 | 0.00 | 1.00 | 1.00 | 1.00 | 1.00 | 0.00 | 0.00 | 1.00 | 1.00 | 0.00 | 1.00 | 1.00 | 1.00 | 1.00 | 1.00 | 0.00 | 1.00 | 0.00 |
| C-Mono3\|GNLY+CD8+_cytotoxic_T_cell | 1.00 | 1.00 | 0.00 | 1.00 | 1.00 | 1.00 | 1.00 | 0.00 | 0.00 | 1.00 | 1.00 | 0.00 | 1.00 | 0.68 | 1.00 | 1.00 | 1.00 | 0.00 | 1.00 | 0.00 |
| C-Mono3\|GZMK+CD8+_cytotoxic_T_cell | 1.00 | 1.00 | 0.00 | 1.00 | 1.00 | 1.00 | 1.00 | 0.00 | 0.00 | 1.00 | 1.00 | 0.00 | 1.00 | 1.00 | 1.00 | 1.00 | 1.00 | 0.00 | 1.00 | 0.00 |
| C-Mono3\|MAI_T | 1.00 | 1.00 | 0.00 | 1.00 | 1.00 | 1.00 | 1.00 | 0.00 | 0.00 | 1.00 | 1.00 | 0.56 | 1.00 | 1.00 | 1.00 | 1.00 | 1.00 | 0.00 | 1.00 | 0.00 |
| C-Mono3\|Memory_B_cell | 1.00 | 1.00 | 0.00 | 1.00 | 1.00 | 1.00 | 1.00 | 0.00 | 0.00 | 1.00 | 1.00 | 1.00 | 0.00 | 1.00 | 1.00 | 1.00 | 1.00 | 0.00 | 1.00 | 0.00 |
| C-Mono3\|NC-Mono | 1.00 | 1.00 | 0.00 | 1.00 | 1.00 | 1.00 | 1.00 | 0.00 | 0.00 | 0.00 | 1.00 | 1.00 | 1.00 | 0.47 | 1.00 | 1.00 | 1.00 | 0.00 | 0.78 | 0.12 |
| C-Mono3\|NKT | 1.00 | 1.00 | 0.00 | 1.00 | 1.00 | 1.00 | 1.00 | 0.00 | 0.00 | 1.00 | 0.00 | 0.00 | 1.00 | 0.00 | 0.00 | 1.00 | 1.00 | 0.00 | 1.00 | 0.00 |
| C-Mono3\|NaÃ¯ve_B_cell | 1.00 | 1.00 | 0.00 | 1.00 | 1.00 | 1.00 | 1.00 | 0.00 | 0.00 | 0.00 | 1.00 | 1.00 | 0.00 | 1.00 | 1.00 | 1.00 | 1.00 | 0.00 | 1.00 | 0.00 |
| C-Mono3\|Plasma | 1.00 | 1.00 | 0.00 | 1.00 | 0.94 | 1.00 | 1.00 | 0.00 | 0.00 | 0.00 | 1.00 | 1.00 | 1.00 | 1.00 | 1.00 | 1.00 | 1.00 | 0.00 | 1.00 | 1.00 |
| C-Mono3\|Treg | 1.00 | 1.00 | 0.00 | 1.00 | 1.00 | 1.00 | 1.00 | 0.00 | 0.00 | 1.00 | 1.00 | 0.06 | 1.00 | 1.00 | 1.00 | 1.00 | 1.00 | 0.00 | 1.00 | 0.00 |
| C-Mono3\|γδ_T | 1.00 | 1.00 | 0.00 | 1.00 | 1.00 | 1.00 | 1.00 | 0.00 | 0.00 | 1.00 | 1.00 | 0.00 | 1.00 | 1.00 | 0.00 | 1.00 | 1.00 | 0.00 | 1.00 | 0.00 |
| CD4+_effector_T_cell\|C-Mono1 | 1.00 | 0.00 | 0.94 | 1.00 | 0.00 | 1.00 | 1.00 | 1.00 | 0.00 | 0.00 | 1.00 | 0.00 | 1.00 | 0.00 | 1.00 | 1.00 | 1.00 | 1.00 | 0.00 | 0.59 |
| CD4+_effector_T_cell\|C-Mono2 | 1.00 | 0.00 | 1.00 | 1.00 | 1.00 | 1.00 | 1.00 | 1.00 | 0.00 | 0.00 | 1.00 | 0.00 | 1.00 | 1.00 | 1.00 | 1.00 | 1.00 | 1.00 | 0.00 | 1.00 |
| CD4+_effector_T_cell\|C-Mono3 | 1.00 | 0.00 | 0.90 | 1.00 | 1.00 | 1.00 | 1.00 | 1.00 | 0.00 | 0.00 | 1.00 | 0.00 | 1.00 | 0.00 | 1.00 | 1.00 | 1.00 | 1.00 | 0.00 | 1.00 |
| CD4+_effector_T_cell\|CD4+_effector_T_cell | 1.00 | 1.00 | 1.00 | 1.00 | 1.00 | 1.00 | 1.00 | 0.68 | 1.00 | 1.00 | 1.00 | 0.00 | 1.00 | 1.00 | 1.00 | 1.00 | 1.00 | 1.00 | 1.00 | 0.00 |
| CD4+_effector_T_cell\|CD4+_memory_T_cell | 1.00 | 1.00 | 1.00 | 1.00 | 1.00 | 1.00 | 1.00 | 0.00 | 1.00 | 1.00 | 1.00 | 0.00 | 1.00 | 1.00 | 1.00 | 1.00 | 1.00 | 1.00 | 1.00 | 0.03 |
| CD4+_effector_T_cell\|CD4+_naÃ¯ve_T_cell | 1.00 | 1.00 | 1.00 | 1.00 | 1.00 | 1.00 | 1.00 | 0.28 | 1.00 | 0.97 | 1.00 | 0.00 | 1.00 | 1.00 | 1.00 | 1.00 | 1.00 | 1.00 | 1.00 | 1.00 |
| CD4+_effector_T_cell\|CD56brightCD16-_NK | 1.00 | 0.00 | 1.00 | 1.00 | 0.00 | 1.00 | 1.00 | 0.00 | 0.94 | 1.00 | 1.00 | 0.00 | 1.00 | 0.00 | 0.00 | 0.00 | 0.00 | 1.00 | 1.00 | 0.00 |
| CD4+_effector_T_cell\|CD56dimCD16+_NK | 1.00 | 1.00 | 1.00 | 1.00 | 0.00 | 1.00 | 1.00 | 0.74 | 0.97 | 1.00 | 0.00 | 0.00 | 1.00 | 0.00 | 0.00 | 0.00 | 1.00 | 1.00 | 1.00 | 0.00 |
| CD4+_effector_T_cell\|CD8+_naÃ¯ve_T_cell | 1.00 | 1.00 | 1.00 | 1.00 | 0.00 | 1.00 | 1.00 | 0.22 | 0.75 | 1.00 | 1.00 | 0.00 | 1.00 | 0.00 | 0.00 | 1.00 | 1.00 | 1.00 | 1.00 | 0.00 |
| CD4+_effector_T_cell\|GNLY+CD8+_cytotoxic_T_cell | 1.00 | 1.00 | 1.00 | 1.00 | 1.00 | 1.00 | 1.00 | 1.00 | 1.00 | 1.00 | 1.00 | 0.00 | 1.00 | 0.00 | 1.00 | 1.00 | 1.00 | 1.00 | 1.00 | 0.00 |
| CD4+_effector_T_cell\|GZMK+CD8+_cytotoxic_T_cell | 0.00 | 1.00 | 1.00 | 1.00 | 1.00 | 1.00 | 1.00 | 0.15 | 1.00 | 1.00 | 1.00 | 0.00 | 1.00 | 1.00 | 1.00 | 1.00 | 1.00 | 1.00 | 1.00 | 0.00 |
| CD4+_effector_T_cell\|MAI_T | 0.00 | 0.00 | 1.00 | 1.00 | 0.00 | 1.00 | 1.00 | 0.00 | 1.00 | 1.00 | 1.00 | 0.00 | 1.00 | 1.00 | 1.00 | 1.00 | 1.00 | 1.00 | 1.00 | 0.06 |
| CD4+_effector_T_cell\|Memory_B_cell | 1.00 | 1.00 | 1.00 | 1.00 | 0.00 | 1.00 | 1.00 | 0.40 | 1.00 | 1.00 | 1.00 | 0.00 | 0.00 | 1.00 | 1.00 | 1.00 | 1.00 | 1.00 | 0.00 | 0.00 |
| CD4+_effector_T_cell\|NC-Mono | 1.00 | 0.00 | 1.00 | 1.00 | 1.00 | 1.00 | 1.00 | 1.00 | 0.06 | 0.94 | 1.00 | 0.00 | 1.00 | 0.00 | 1.00 | 1.00 | 1.00 | 1.00 | 0.00 | 0.72 |
| CD4+_effector_T_cell\|NKT | 1.00 | 1.00 | 1.00 | 1.00 | 0.00 | 1.00 | 1.00 | 0.03 | 0.28 | 1.00 | 0.00 | 0.00 | 1.00 | 0.00 | 0.00 | 0.00 | 0.00 | 1.00 | 1.00 | 0.00 |
| CD4+_effector_T_cell\|NaÃ¯ve_B_cell | 1.00 | 1.00 | 1.00 | 1.00 | 0.00 | 1.00 | 1.00 | 1.00 | 1.00 | 0.69 | 1.00 | 1.00 | 0.00 | 1.00 | 1.00 | 1.00 | 1.00 | 1.00 | 1.00 | 0.00 |
| CD4+_effector_T_cell\|Plasma | 1.00 | 1.00 | 1.00 | 1.00 | 0.00 | 1.00 | 1.00 | 0.00 | 0.09 | 1.00 | 1.00 | 0.06 | 1.00 | 1.00 | 1.00 | 1.00 | 1.00 | 1.00 | 1.00 | 1.00 |
| CD4+_effector_T_cell\|Treg | 1.00 | 1.00 | 1.00 | 1.00 | 1.00 | 1.00 | 1.00 | 0.06 | 1.00 | 1.00 | 1.00 | 0.00 | 1.00 | 1.00 | 1.00 | 1.00 | 1.00 | 1.00 | 1.00 | 0.00 |
| CD4+_effector_T_cell\|γδ_T | 0.00 | 1.00 | 1.00 | 1.00 | 1.00 | 1.00 | 1.00 | 0.00 | 1.00 | 1.00 | 1.00 | 0.00 | 1.00 | 1.00 | 0.00 | 1.00 | 1.00 | 1.00 | 1.00 | 0.00 |
| CD4+_memory_T_cell\|C-Mono1 | 1.00 | 0.00 | 1.00 | 1.00 | 0.00 | 1.00 | 1.00 | 1.00 | 0.97 | 1.00 | 1.00 | 1.00 | 1.00 | 0.00 | 1.00 | 1.00 | 1.00 | 1.00 | 0.00 | 1.00 |
| CD4+_memory_T_cell\|C-Mono2 | 1.00 | 0.00 | 1.00 | 1.00 | 1.00 | 1.00 | 1.00 | 1.00 | 1.00 | 1.00 | 1.00 | 1.00 | 1.00 | 1.00 | 1.00 | 1.00 | 1.00 | 1.00 | 0.00 | 1.00 |
| CD4+_memory_T_cell\|C-Mono3 | 1.00 | 0.00 | 1.00 | 1.00 | 1.00 | 1.00 | 1.00 | 1.00 | 1.00 | 1.00 | 1.00 | 0.69 | 1.00 | 0.03 | 1.00 | 1.00 | 1.00 | 1.00 | 0.00 | 1.00 |
| CD4+_memory_T_cell\|CD4+_effector_T_cell | 1.00 | 1.00 | 1.00 | 1.00 | 1.00 | 1.00 | 1.00 | 1.00 | 1.00 | 1.00 | 1.00 | 0.00 | 1.00 | 1.00 | 1.00 | 1.00 | 1.00 | 1.00 | 1.00 | 0.00 |
| CD4+_memory_T_cell\|CD4+_memory_T_cell | 1.00 | 1.00 | 1.00 | 1.00 | 1.00 | 1.00 | 1.00 | 1.00 | 1.00 | 1.00 | 1.00 | 0.00 | 1.00 | 1.00 | 1.00 | 1.00 | 1.00 | 1.00 | 1.00 | 0.75 |
| CD4+_memory_T_cell\|CD4+_naÃ¯ve_T_cell | 1.00 | 1.00 | 1.00 | 1.00 | 1.00 | 1.00 | 1.00 | 1.00 | 1.00 | 1.00 | 1.00 | 0.00 | 1.00 | 1.00 | 1.00 | 1.00 | 1.00 | 1.00 | 1.00 | 1.00 |
| CD4+_memory_T_cell\|CD56brightCD16-_NK | 1.00 | 1.00 | 1.00 | 1.00 | 0.00 | 1.00 | 1.00 | 1.00 | 1.00 | 1.00 | 1.00 | 0.03 | 1.00 | 0.00 | 0.00 | 0.00 | 0.00 | 1.00 | 1.00 | 0.00 |
| CD4+_memory_T_cell\|CD56dimCD16+_NK | 1.00 | 1.00 | 1.00 | 1.00 | 0.00 | 1.00 | 1.00 | 1.00 | 1.00 | 1.00 | 0.00 | 0.00 | 1.00 | 0.00 | 0.00 | 0.00 | 1.00 | 1.00 | 1.00 | 0.00 |
| CD4+_memory_T_cell\|CD8+_naÃ¯ve_T_cell | 1.00 | 1.00 | 1.00 | 1.00 | 0.00 | 1.00 | 1.00 | 1.00 | 1.00 | 1.00 | 1.00 | 0.00 | 1.00 | 0.06 | 0.00 | 1.00 | 1.00 | 1.00 | 1.00 | 0.03 |
| CD4+_memory_T_cell\|GNLY+CD8+_cytotoxic_T_cell | 1.00 | 1.00 | 1.00 | 1.00 | 1.00 | 1.00 | 1.00 | 1.00 | 1.00 | 1.00 | 1.00 | 0.00 | 1.00 | 0.00 | 1.00 | 1.00 | 1.00 | 1.00 | 1.00 | 0.00 |
| CD4+_memory_T_cell\|GZMK+CD8+_cytotoxic_T_cell | 1.00 | 1.00 | 1.00 | 1.00 | 1.00 | 1.00 | 1.00 | 1.00 | 1.00 | 1.00 | 1.00 | 0.00 | 1.00 | 1.00 | 1.00 | 1.00 | 1.00 | 1.00 | 1.00 | 0.00 |
| CD4+_memory_T_cell\|MAI_T | 0.91 | 1.00 | 1.00 | 1.00 | 0.00 | 1.00 | 1.00 | 0.97 | 1.00 | 1.00 | 1.00 | 0.62 | 1.00 | 1.00 | 1.00 | 1.00 | 1.00 | 1.00 | 1.00 | 0.22 |
| CD4+_memory_T_cell\|Memory_B_cell | 1.00 | 1.00 | 1.00 | 1.00 | 0.00 | 1.00 | 1.00 | 1.00 | 1.00 | 1.00 | 1.00 | 1.00 | 0.00 | 1.00 | 1.00 | 1.00 | 1.00 | 1.00 | 0.00 | 0.00 |
| CD4+_memory_T_cell\|NC-Mono | 1.00 | 1.00 | 1.00 | 1.00 | 1.00 | 1.00 | 1.00 | 1.00 | 1.00 | 1.00 | 1.00 | 1.00 | 1.00 | 0.00 | 1.00 | 1.00 | 1.00 | 1.00 | 0.00 | 1.00 |
| CD4+_memory_T_cell\|NKT | 1.00 | 1.00 | 1.00 | 1.00 | 0.00 | 1.00 | 1.00 | 1.00 | 1.00 | 1.00 | 0.00 | 0.00 | 1.00 | 0.00 | 0.00 | 0.00 | 0.00 | 1.00 | 1.00 | 0.00 |
| CD4+_memory_T_cell\|NaÃ¯ve_B_cell | 1.00 | 1.00 | 1.00 | 1.00 | 0.00 | 1.00 | 1.00 | 1.00 | 1.00 | 1.00 | 1.00 | 1.00 | 0.00 | 1.00 | 1.00 | 1.00 | 1.00 | 1.00 | 1.00 | 0.81 |
| CD4+_memory_T_cell\|Plasma | 1.00 | 1.00 | 1.00 | 1.00 | 0.00 | 1.00 | 1.00 | 0.94 | 1.00 | 1.00 | 1.00 | 1.00 | 1.00 | 1.00 | 1.00 | 1.00 | 1.00 | 1.00 | 1.00 | 1.00 |
| CD4+_memory_T_cell\|Treg | 1.00 | 1.00 | 1.00 | 1.00 | 1.00 | 1.00 | 1.00 | 1.00 | 1.00 | 1.00 | 1.00 | 0.09 | 1.00 | 1.00 | 1.00 | 1.00 | 1.00 | 1.00 | 1.00 | 0.16 |
| CD4+_memory_T_cell\|γδ_T | 1.00 | 1.00 | 1.00 | 1.00 | 1.00 | 1.00 | 1.00 | 1.00 | 1.00 | 1.00 | 1.00 | 0.00 | 1.00 | 1.00 | 0.00 | 1.00 | 1.00 | 1.00 | 1.00 | 0.00 |
| CD4+_naÃ¯ve_T_cell\|C-Mono1 | 1.00 | 1.00 | 1.00 | 1.00 | 1.00 | 1.00 | 1.00 | 1.00 | 1.00 | 1.00 | 1.00 | 1.00 | 1.00 | 0.00 | 1.00 | 1.00 | 1.00 | 1.00 | 0.00 | 1.00 |
| CD4+_naÃ¯ve_T_cell\|C-Mono2 | 1.00 | 1.00 | 1.00 | 1.00 | 1.00 | 1.00 | 1.00 | 1.00 | 1.00 | 1.00 | 1.00 | 1.00 | 1.00 | 1.00 | 1.00 | 1.00 | 1.00 | 1.00 | 0.00 | 1.00 |
| CD4+_naÃ¯ve_T_cell\|C-Mono3 | 1.00 | 1.00 | 1.00 | 1.00 | 1.00 | 1.00 | 1.00 | 1.00 | 1.00 | 1.00 | 1.00 | 1.00 | 1.00 | 0.00 | 1.00 | 1.00 | 1.00 | 1.00 | 0.00 | 1.00 |
| CD4+_naÃ¯ve_T_cell\|CD4+_effector_T_cell | 1.00 | 1.00 | 1.00 | 1.00 | 1.00 | 1.00 | 1.00 | 1.00 | 1.00 | 1.00 | 1.00 | 1.00 | 1.00 | 1.00 | 1.00 | 1.00 | 1.00 | 1.00 | 1.00 | 0.00 |
| CD4+_naÃ¯ve_T_cell\|CD4+_memory_T_cell | 1.00 | 1.00 | 1.00 | 1.00 | 1.00 | 1.00 | 1.00 | 1.00 | 1.00 | 1.00 | 1.00 | 0.90 | 1.00 | 1.00 | 1.00 | 1.00 | 1.00 | 1.00 | 1.00 | 1.00 |
| CD4+_naÃ¯ve_T_cell\|CD4+_naÃ¯ve_T_cell | 1.00 | 1.00 | 1.00 | 1.00 | 1.00 | 1.00 | 1.00 | 1.00 | 1.00 | 1.00 | 1.00 | 1.00 | 1.00 | 1.00 | 1.00 | 1.00 | 1.00 | 1.00 | 1.00 | 1.00 |
| CD4+_naÃ¯ve_T_cell\|CD56brightCD16-_NK | 1.00 | 1.00 | 1.00 | 1.00 | 1.00 | 1.00 | 1.00 | 1.00 | 1.00 | 1.00 | 1.00 | 1.00 | 1.00 | 0.00 | 0.00 | 0.00 | 1.00 | 1.00 | 1.00 | 0.19 |
| CD4+_naÃ¯ve_T_cell\|CD56dimCD16+_NK | 1.00 | 1.00 | 1.00 | 1.00 | 1.00 | 1.00 | 1.00 | 1.00 | 1.00 | 1.00 | 1.00 | 1.00 | 1.00 | 0.00 | 0.00 | 0.00 | 1.00 | 1.00 | 1.00 | 0.00 |
| CD4+_naÃ¯ve_T_cell\|CD8+_naÃ¯ve_T_cell | 1.00 | 1.00 | 1.00 | 1.00 | 1.00 | 1.00 | 1.00 | 1.00 | 1.00 | 1.00 | 1.00 | 0.94 | 1.00 | 0.00 | 1.00 | 1.00 | 1.00 | 1.00 | 1.00 | 1.00 |
| CD4+_naÃ¯ve_T_cell\|GNLY+CD8+_cytotoxic_T_cell | 1.00 | 1.00 | 1.00 | 1.00 | 1.00 | 1.00 | 1.00 | 1.00 | 1.00 | 1.00 | 1.00 | 1.00 | 1.00 | 0.00 | 1.00 | 1.00 | 1.00 | 1.00 | 1.00 | 0.00 |
| CD4+_naÃ¯ve_T_cell\|GZMK+CD8+_cytotoxic_T_cell | 1.00 | 1.00 | 1.00 | 1.00 | 1.00 | 1.00 | 1.00 | 1.00 | 1.00 | 1.00 | 1.00 | 1.00 | 1.00 | 1.00 | 1.00 | 1.00 | 1.00 | 1.00 | 1.00 | 0.72 |
| CD4+_naÃ¯ve_T_cell\|MAI_T | 1.00 | 1.00 | 1.00 | 1.00 | 1.00 | 1.00 | 1.00 | 1.00 | 1.00 | 1.00 | 1.00 | 1.00 | 1.00 | 1.00 | 1.00 | 1.00 | 1.00 | 1.00 | 1.00 | 0.97 |
| CD4+_naÃ¯ve_T_cell\|Memory_B_cell | 1.00 | 1.00 | 1.00 | 1.00 | 1.00 | 1.00 | 1.00 | 1.00 | 1.00 | 1.00 | 1.00 | 1.00 | 0.00 | 1.00 | 1.00 | 1.00 | 1.00 | 1.00 | 0.00 | 0.00 |
| CD4+_naÃ¯ve_T_cell\|NC-Mono | 1.00 | 1.00 | 1.00 | 1.00 | 1.00 | 1.00 | 1.00 | 1.00 | 1.00 | 1.00 | 1.00 | 1.00 | 1.00 | 0.00 | 1.00 | 1.00 | 1.00 | 1.00 | 0.00 | 1.00 |
| CD4+_naÃ¯ve_T_cell\|NKT | 1.00 | 1.00 | 1.00 | 1.00 | 1.00 | 1.00 | 1.00 | 1.00 | 1.00 | 1.00 | 1.00 | 0.51 | 1.00 | 0.00 | 0.00 | 1.00 | 1.00 | 1.00 | 1.00 | 0.00 |
| CD4+_naÃ¯ve_T_cell\|NaÃ¯ve_B_cell | 1.00 | 1.00 | 1.00 | 1.00 | 0.91 | 1.00 | 1.00 | 1.00 | 1.00 | 1.00 | 1.00 | 1.00 | 0.00 | 1.00 | 1.00 | 1.00 | 1.00 | 1.00 | 1.00 | 1.00 |
| CD4+_naÃ¯ve_T_cell\|Plasma | 1.00 | 1.00 | 1.00 | 1.00 | 0.06 | 1.00 | 1.00 | 1.00 | 1.00 | 1.00 | 1.00 | 1.00 | 1.00 | 1.00 | 1.00 | 1.00 | 1.00 | 1.00 | 1.00 | 1.00 |
| CD4+_naÃ¯ve_T_cell\|Treg | 1.00 | 1.00 | 1.00 | 1.00 | 1.00 | 1.00 | 1.00 | 1.00 | 1.00 | 1.00 | 1.00 | 1.00 | 1.00 | 1.00 | 1.00 | 1.00 | 1.00 | 1.00 | 1.00 | 1.00 |
| CD4+_naÃ¯ve_T_cell\|γδ_T | 1.00 | 1.00 | 1.00 | 1.00 | 1.00 | 1.00 | 1.00 | 1.00 | 1.00 | 1.00 | 1.00 | 1.00 | 1.00 | 1.00 | 0.00 | 1.00 | 1.00 | 1.00 | 1.00 | 0.81 |
| CD56brightCD16-_NK\|C-Mono1 | 1.00 | 0.00 | 0.00 | 1.00 | 0.00 | 0.00 | 0.00 | 0.97 | 0.00 | 0.00 | 1.00 | 0.72 | 1.00 | 1.00 | 1.00 | 1.00 | 1.00 | 1.00 | 1.00 | 0.03 |
| CD56brightCD16-_NK\|C-Mono2 | 1.00 | 0.00 | 0.00 | 1.00 | 1.00 | 0.00 | 1.00 | 1.00 | 0.00 | 0.00 | 1.00 | 0.94 | 1.00 | 1.00 | 1.00 | 1.00 | 1.00 | 1.00 | 1.00 | 1.00 |
| CD56brightCD16-_NK\|C-Mono3 | 1.00 | 0.00 | 0.00 | 1.00 | 1.00 | 0.00 | 0.00 | 1.00 | 0.00 | 0.00 | 1.00 | 0.41 | 1.00 | 1.00 | 1.00 | 1.00 | 1.00 | 1.00 | 1.00 | 1.00 |
| CD56brightCD16-_NK\|CD4+_effector_T_cell | 1.00 | 1.00 | 0.00 | 1.00 | 1.00 | 0.00 | 0.00 | 0.38 | 0.69 | 1.00 | 1.00 | 0.03 | 1.00 | 1.00 | 1.00 | 1.00 | 1.00 | 1.00 | 1.00 | 0.00 |
| CD56brightCD16-_NK\|CD4+_memory_T_cell | 1.00 | 1.00 | 0.00 | 1.00 | 1.00 | 0.00 | 0.00 | 0.03 | 0.84 | 1.00 | 1.00 | 0.00 | 1.00 | 1.00 | 1.00 | 1.00 | 1.00 | 1.00 | 1.00 | 0.03 |
| CD56brightCD16-_NK\|CD4+_naÃ¯ve_T_cell | 1.00 | 1.00 | 0.00 | 1.00 | 1.00 | 0.00 | 0.00 | 0.25 | 1.00 | 0.57 | 1.00 | 0.00 | 1.00 | 1.00 | 1.00 | 1.00 | 1.00 | 1.00 | 1.00 | 0.19 |
| CD56brightCD16-_NK\|CD56brightCD16-_NK | 1.00 | 0.00 | 0.00 | 1.00 | 0.00 | 0.00 | 0.00 | 0.00 | 0.66 | 1.00 | 1.00 | 0.03 | 1.00 | 0.85 | 0.00 | 0.00 | 0.00 | 1.00 | 1.00 | 0.00 |
| CD56brightCD16-_NK\|CD56dimCD16+_NK | 1.00 | 1.00 | 0.00 | 1.00 | 0.00 | 0.00 | 0.00 | 0.38 | 0.69 | 1.00 | 0.00 | 0.00 | 1.00 | 0.81 | 0.00 | 0.00 | 1.00 | 1.00 | 1.00 | 0.00 |
| CD56brightCD16-_NK\|CD8+_naÃ¯ve_T_cell | 1.00 | 1.00 | 0.00 | 1.00 | 0.00 | 0.00 | 0.00 | 0.16 | 0.38 | 1.00 | 1.00 | 0.00 | 1.00 | 1.00 | 0.00 | 1.00 | 1.00 | 1.00 | 1.00 | 0.00 |
| CD56brightCD16-_NK\|GNLY+CD8+_cytotoxic_T_cell | 1.00 | 1.00 | 0.00 | 1.00 | 1.00 | 0.00 | 0.00 | 0.72 | 0.66 | 1.00 | 1.00 | 0.00 | 1.00 | 1.00 | 1.00 | 1.00 | 1.00 | 1.00 | 1.00 | 0.00 |
| CD56brightCD16-_NK\|GZMK+CD8+_cytotoxic_T_cell | 0.00 | 1.00 | 0.00 | 1.00 | 1.00 | 0.00 | 0.00 | 0.19 | 0.69 | 1.00 | 1.00 | 0.00 | 1.00 | 1.00 | 1.00 | 1.00 | 1.00 | 1.00 | 1.00 | 0.00 |
| CD56brightCD16-_NK\|MAI_T | 0.00 | 0.00 | 0.00 | 1.00 | 0.00 | 0.00 | 0.00 | 0.00 | 0.81 | 1.00 | 1.00 | 0.38 | 1.00 | 1.00 | 1.00 | 1.00 | 1.00 | 1.00 | 1.00 | 0.00 |
| CD56brightCD16-_NK\|Memory_B_cell | 1.00 | 1.00 | 0.00 | 1.00 | 0.00 | 0.00 | 0.00 | 0.25 | 0.78 | 1.00 | 1.00 | 1.00 | 0.00 | 1.00 | 1.00 | 1.00 | 1.00 | 1.00 | 1.00 | 0.00 |
| CD56brightCD16-_NK\|NC-Mono | 1.00 | 0.00 | 0.00 | 1.00 | 1.00 | 0.00 | 0.00 | 1.00 | 0.10 | 0.47 | 1.00 | 0.88 | 1.00 | 1.00 | 1.00 | 1.00 | 1.00 | 1.00 | 0.00 | 0.13 |
| CD56brightCD16-_NK\|NKT | 1.00 | 1.00 | 0.00 | 1.00 | 0.00 | 0.00 | 0.00 | 0.06 | 0.31 | 1.00 | 0.00 | 0.00 | 1.00 | 0.78 | 0.00 | 0.00 | 0.00 | 1.00 | 1.00 | 0.00 |
| CD56brightCD16-_NK\|NaÃ¯ve_B_cell | 1.00 | 1.00 | 0.00 | 1.00 | 0.00 | 0.00 | 0.00 | 0.90 | 1.00 | 0.35 | 1.00 | 1.00 | 0.00 | 1.00 | 1.00 | 1.00 | 1.00 | 1.00 | 1.00 | 0.00 |
| CD56brightCD16-_NK\|Plasma | 1.00 | 1.00 | 0.00 | 1.00 | 0.00 | 0.00 | 1.00 | 0.00 | 0.10 | 0.75 | 1.00 | 1.00 | 1.00 | 1.00 | 1.00 | 1.00 | 1.00 | 1.00 | 1.00 | 0.97 |
| CD56brightCD16-_NK\|Treg | 1.00 | 1.00 | 0.00 | 1.00 | 1.00 | 0.00 | 0.00 | 0.03 | 0.75 | 1.00 | 1.00 | 0.10 | 1.00 | 1.00 | 1.00 | 1.00 | 1.00 | 1.00 | 1.00 | 0.00 |
| CD56brightCD16-_NK\|γδ_T | 0.00 | 1.00 | 0.00 | 1.00 | 1.00 | 0.00 | 0.00 | 0.00 | 0.75 | 1.00 | 1.00 | 0.00 | 1.00 | 1.00 | 0.00 | 1.00 | 1.00 | 1.00 | 1.00 | 0.00 |
| CD56dimCD16+_NK\|C-Mono1 | 1.00 | 0.00 | 0.00 | 1.00 | 0.00 | 0.00 | 1.00 | 1.00 | 0.85 | 1.00 | 1.00 | 0.00 | 1.00 | 1.00 | 1.00 | 1.00 | 1.00 | 1.00 | 1.00 | 0.00 |
| CD56dimCD16+_NK\|C-Mono2 | 1.00 | 0.00 | 0.00 | 1.00 | 1.00 | 1.00 | 1.00 | 1.00 | 1.00 | 1.00 | 1.00 | 0.35 | 1.00 | 1.00 | 1.00 | 1.00 | 1.00 | 1.00 | 1.00 | 1.00 |
| CD56dimCD16+_NK\|C-Mono3 | 1.00 | 0.00 | 0.00 | 1.00 | 1.00 | 0.00 | 0.00 | 1.00 | 1.00 | 1.00 | 1.00 | 0.00 | 1.00 | 1.00 | 1.00 | 1.00 | 1.00 | 1.00 | 1.00 | 1.00 |
| CD56dimCD16+_NK\|CD4+_effector_T_cell | 1.00 | 1.00 | 0.00 | 1.00 | 1.00 | 0.00 | 0.00 | 1.00 | 1.00 | 1.00 | 1.00 | 0.00 | 1.00 | 1.00 | 1.00 | 1.00 | 1.00 | 1.00 | 1.00 | 0.00 |
| CD56dimCD16+_NK\|CD4+_memory_T_cell | 1.00 | 1.00 | 0.00 | 1.00 | 1.00 | 0.00 | 0.00 | 1.00 | 1.00 | 1.00 | 1.00 | 0.00 | 1.00 | 1.00 | 1.00 | 1.00 | 1.00 | 1.00 | 1.00 | 0.00 |
| CD56dimCD16+_NK\|CD4+_naÃ¯ve_T_cell | 1.00 | 1.00 | 0.00 | 1.00 | 1.00 | 0.00 | 0.00 | 1.00 | 1.00 | 1.00 | 1.00 | 0.00 | 1.00 | 1.00 | 1.00 | 1.00 | 1.00 | 1.00 | 1.00 | 0.00 |
| CD56dimCD16+_NK\|CD56brightCD16-_NK | 1.00 | 0.00 | 0.00 | 1.00 | 0.00 | 0.00 | 0.00 | 1.00 | 1.00 | 1.00 | 1.00 | 0.00 | 1.00 | 0.47 | 0.00 | 0.00 | 0.00 | 1.00 | 1.00 | 0.00 |
| CD56dimCD16+_NK\|CD56dimCD16+_NK | 1.00 | 1.00 | 0.00 | 1.00 | 0.00 | 0.00 | 0.00 | 1.00 | 1.00 | 1.00 | 0.00 | 0.00 | 1.00 | 0.13 | 0.00 | 0.00 | 1.00 | 1.00 | 1.00 | 0.00 |
| CD56dimCD16+_NK\|CD8+_naÃ¯ve_T_cell | 1.00 | 1.00 | 0.00 | 1.00 | 0.00 | 0.00 | 0.00 | 1.00 | 1.00 | 1.00 | 1.00 | 0.00 | 1.00 | 1.00 | 0.00 | 1.00 | 1.00 | 1.00 | 1.00 | 0.00 |
| CD56dimCD16+_NK\|GNLY+CD8+_cytotoxic_T_cell | 1.00 | 1.00 | 0.00 | 1.00 | 1.00 | 0.00 | 0.00 | 1.00 | 1.00 | 1.00 | 1.00 | 0.00 | 1.00 | 1.00 | 1.00 | 1.00 | 1.00 | 1.00 | 1.00 | 0.00 |
| CD56dimCD16+_NK\|GZMK+CD8+_cytotoxic_T_cell | 0.00 | 1.00 | 0.00 | 1.00 | 1.00 | 0.00 | 0.00 | 1.00 | 1.00 | 1.00 | 1.00 | 0.00 | 1.00 | 1.00 | 1.00 | 1.00 | 1.00 | 1.00 | 1.00 | 0.00 |
| CD56dimCD16+_NK\|MAI_T | 0.00 | 0.00 | 0.00 | 1.00 | 0.00 | 0.00 | 0.00 | 0.97 | 1.00 | 1.00 | 1.00 | 0.00 | 1.00 | 1.00 | 1.00 | 1.00 | 1.00 | 1.00 | 1.00 | 0.00 |
| CD56dimCD16+_NK\|Memory_B_cell | 1.00 | 1.00 | 0.00 | 1.00 | 0.00 | 0.00 | 0.00 | 1.00 | 1.00 | 1.00 | 1.00 | 1.00 | 0.00 | 1.00 | 1.00 | 1.00 | 1.00 | 1.00 | 1.00 | 0.00 |
| CD56dimCD16+_NK\|NC-Mono | 1.00 | 0.00 | 0.00 | 1.00 | 1.00 | 0.00 | 0.00 | 1.00 | 1.00 | 1.00 | 1.00 | 0.41 | 1.00 | 1.00 | 1.00 | 1.00 | 1.00 | 1.00 | 0.00 | 0.00 |
| CD56dimCD16+_NK\|NKT | 1.00 | 1.00 | 0.00 | 1.00 | 0.00 | 0.00 | 0.00 | 1.00 | 1.00 | 1.00 | 0.00 | 0.00 | 1.00 | 0.15 | 0.00 | 0.00 | 0.00 | 1.00 | 1.00 | 0.00 |
| CD56dimCD16+_NK\|NaÃ¯ve_B_cell | 1.00 | 1.00 | 0.00 | 1.00 | 0.00 | 0.00 | 0.97 | 1.00 | 1.00 | 1.00 | 1.00 | 1.00 | 0.00 | 1.00 | 1.00 | 1.00 | 1.00 | 1.00 | 1.00 | 0.00 |
| CD56dimCD16+_NK\|Plasma | 1.00 | 1.00 | 0.00 | 1.00 | 0.00 | 1.00 | 1.00 | 0.97 | 1.00 | 1.00 | 1.00 | 1.00 | 1.00 | 1.00 | 1.00 | 1.00 | 1.00 | 1.00 | 1.00 | 0.63 |
| CD56dimCD16+_NK\|Treg | 1.00 | 1.00 | 0.00 | 1.00 | 1.00 | 0.00 | 0.00 | 1.00 | 1.00 | 1.00 | 1.00 | 0.00 | 1.00 | 1.00 | 1.00 | 1.00 | 1.00 | 1.00 | 1.00 | 0.00 |
| CD56dimCD16+_NK\|γδ_T | 0.00 | 1.00 | 0.00 | 1.00 | 1.00 | 0.00 | 0.00 | 1.00 | 1.00 | 1.00 | 1.00 | 0.00 | 1.00 | 1.00 | 0.00 | 1.00 | 1.00 | 1.00 | 1.00 | 0.00 |
| CD8+_naÃ¯ve_T_cell\|C-Mono1 | 1.00 | 0.00 | 0.00 | 1.00 | 0.38 | 1.00 | 1.00 | 1.00 | 1.00 | 1.00 | 1.00 | 0.85 | 1.00 | 0.00 | 1.00 | 1.00 | 1.00 | 1.00 | 0.00 | 0.81 |
| CD8+_naÃ¯ve_T_cell\|C-Mono2 | 1.00 | 0.00 | 0.00 | 1.00 | 1.00 | 1.00 | 1.00 | 1.00 | 1.00 | 1.00 | 1.00 | 1.00 | 1.00 | 1.00 | 1.00 | 1.00 | 1.00 | 1.00 | 0.00 | 1.00 |
| CD8+_naÃ¯ve_T_cell\|C-Mono3 | 1.00 | 0.00 | 0.00 | 1.00 | 1.00 | 1.00 | 1.00 | 1.00 | 1.00 | 1.00 | 1.00 | 0.47 | 1.00 | 0.00 | 1.00 | 1.00 | 1.00 | 1.00 | 0.00 | 1.00 |
| CD8+_naÃ¯ve_T_cell\|CD4+_effector_T_cell | 1.00 | 1.00 | 1.00 | 1.00 | 1.00 | 0.00 | 1.00 | 1.00 | 1.00 | 1.00 | 1.00 | 0.03 | 1.00 | 1.00 | 1.00 | 1.00 | 1.00 | 1.00 | 1.00 | 0.00 |
| CD8+_naÃ¯ve_T_cell\|CD4+_memory_T_cell | 1.00 | 1.00 | 1.00 | 1.00 | 1.00 | 0.00 | 1.00 | 1.00 | 1.00 | 1.00 | 1.00 | 0.00 | 1.00 | 1.00 | 1.00 | 1.00 | 1.00 | 1.00 | 1.00 | 0.13 |
| CD8+_naÃ¯ve_T_cell\|CD4+_naÃ¯ve_T_cell | 1.00 | 1.00 | 1.00 | 1.00 | 1.00 | 1.00 | 1.00 | 1.00 | 1.00 | 1.00 | 1.00 | 0.00 | 1.00 | 1.00 | 1.00 | 1.00 | 1.00 | 1.00 | 1.00 | 1.00 |
| CD8+_naÃ¯ve_T_cell\|CD56brightCD16-_NK | 1.00 | 0.00 | 0.97 | 1.00 | 0.13 | 0.00 | 1.00 | 1.00 | 1.00 | 1.00 | 1.00 | 0.06 | 1.00 | 0.00 | 0.00 | 0.00 | 0.00 | 1.00 | 1.00 | 0.00 |
| CD8+_naÃ¯ve_T_cell\|CD56dimCD16+_NK | 1.00 | 1.00 | 1.00 | 1.00 | 0.00 | 0.00 | 1.00 | 1.00 | 1.00 | 1.00 | 0.00 | 0.00 | 1.00 | 0.00 | 0.00 | 0.00 | 1.00 | 1.00 | 1.00 | 0.00 |
| CD8+_naÃ¯ve_T_cell\|CD8+_naÃ¯ve_T_cell | 1.00 | 1.00 | 1.00 | 1.00 | 0.26 | 0.00 | 1.00 | 1.00 | 1.00 | 1.00 | 1.00 | 0.00 | 1.00 | 0.00 | 0.00 | 1.00 | 1.00 | 1.00 | 1.00 | 0.00 |
| CD8+_naÃ¯ve_T_cell\|GNLY+CD8+_cytotoxic_T_cell | 1.00 | 1.00 | 1.00 | 1.00 | 1.00 | 0.00 | 1.00 | 1.00 | 1.00 | 1.00 | 1.00 | 0.00 | 1.00 | 0.00 | 1.00 | 1.00 | 1.00 | 1.00 | 1.00 | 0.00 |
| CD8+_naÃ¯ve_T_cell\|GZMK+CD8+_cytotoxic_T_cell | 0.00 | 1.00 | 1.00 | 1.00 | 1.00 | 0.00 | 1.00 | 1.00 | 1.00 | 1.00 | 1.00 | 0.00 | 1.00 | 1.00 | 1.00 | 1.00 | 1.00 | 1.00 | 1.00 | 0.00 |
| CD8+_naÃ¯ve_T_cell\|MAI_T | 0.00 | 0.00 | 1.00 | 1.00 | 0.13 | 0.00 | 1.00 | 1.00 | 1.00 | 1.00 | 1.00 | 0.47 | 1.00 | 1.00 | 1.00 | 1.00 | 1.00 | 1.00 | 1.00 | 0.13 |
| CD8+_naÃ¯ve_T_cell\|Memory_B_cell | 1.00 | 1.00 | 1.00 | 1.00 | 0.00 | 0.03 | 1.00 | 1.00 | 1.00 | 1.00 | 1.00 | 1.00 | 0.00 | 1.00 | 1.00 | 1.00 | 1.00 | 1.00 | 0.00 | 0.00 |
| CD8+_naÃ¯ve_T_cell\|NC-Mono | 1.00 | 0.00 | 0.90 | 1.00 | 1.00 | 0.00 | 1.00 | 1.00 | 1.00 | 1.00 | 1.00 | 1.00 | 1.00 | 0.00 | 1.00 | 1.00 | 1.00 | 1.00 | 0.00 | 0.82 |
| CD8+_naÃ¯ve_T_cell\|NKT | 1.00 | 1.00 | 1.00 | 1.00 | 0.03 | 0.00 | 1.00 | 1.00 | 1.00 | 1.00 | 0.00 | 0.00 | 1.00 | 0.00 | 0.00 | 0.00 | 0.00 | 1.00 | 1.00 | 0.00 |
| CD8+_naÃ¯ve_T_cell\|NaÃ¯ve_B_cell | 1.00 | 1.00 | 1.00 | 1.00 | 0.00 | 1.00 | 1.00 | 1.00 | 1.00 | 1.00 | 1.00 | 1.00 | 0.00 | 1.00 | 1.00 | 1.00 | 1.00 | 1.00 | 1.00 | 0.22 |
| CD8+_naÃ¯ve_T_cell\|Plasma | 1.00 | 1.00 | 1.00 | 1.00 | 0.00 | 1.00 | 1.00 | 1.00 | 1.00 | 1.00 | 1.00 | 1.00 | 1.00 | 1.00 | 1.00 | 1.00 | 1.00 | 1.00 | 1.00 | 1.00 |
| CD8+_naÃ¯ve_T_cell\|Treg | 1.00 | 1.00 | 1.00 | 1.00 | 1.00 | 0.00 | 1.00 | 1.00 | 1.00 | 1.00 | 1.00 | 0.03 | 1.00 | 1.00 | 1.00 | 1.00 | 1.00 | 1.00 | 1.00 | 0.00 |
| CD8+_naÃ¯ve_T_cell\|γδ_T | 0.00 | 1.00 | 1.00 | 1.00 | 1.00 | 0.00 | 1.00 | 1.00 | 1.00 | 1.00 | 1.00 | 0.00 | 1.00 | 1.00 | 0.00 | 1.00 | 1.00 | 1.00 | 1.00 | 0.00 |
| GNLY+CD8+_cytotoxic_T_cell\|C-Mono1 | 1.00 | 0.00 | 0.00 | 1.00 | 0.00 | 1.00 | 1.00 | 1.00 | 0.00 | 0.00 | 1.00 | 0.00 | 1.00 | 0.03 | 1.00 | 1.00 | 1.00 | 1.00 | 0.00 | 0.19 |
| GNLY+CD8+_cytotoxic_T_cell\|C-Mono2 | 1.00 | 0.00 | 0.00 | 1.00 | 1.00 | 1.00 | 1.00 | 1.00 | 0.00 | 0.31 | 1.00 | 0.00 | 1.00 | 1.00 | 1.00 | 1.00 | 1.00 | 1.00 | 0.00 | 1.00 |
| GNLY+CD8+_cytotoxic_T_cell\|C-Mono3 | 1.00 | 0.00 | 0.00 | 1.00 | 1.00 | 1.00 | 1.00 | 1.00 | 0.00 | 0.06 | 1.00 | 0.00 | 1.00 | 0.60 | 1.00 | 1.00 | 1.00 | 1.00 | 0.00 | 1.00 |
| GNLY+CD8+_cytotoxic_T_cell\|CD4+_effector_T_cell | 1.00 | 1.00 | 1.00 | 1.00 | 1.00 | 1.00 | 1.00 | 1.00 | 1.00 | 1.00 | 1.00 | 0.00 | 1.00 | 1.00 | 1.00 | 1.00 | 1.00 | 1.00 | 1.00 | 0.00 |
| GNLY+CD8+_cytotoxic_T_cell\|CD4+_memory_T_cell | 1.00 | 1.00 | 1.00 | 1.00 | 1.00 | 1.00 | 1.00 | 0.79 | 1.00 | 1.00 | 1.00 | 0.00 | 1.00 | 1.00 | 1.00 | 1.00 | 1.00 | 1.00 | 1.00 | 0.00 |
| GNLY+CD8+_cytotoxic_T_cell\|CD4+_naÃ¯ve_T_cell | 1.00 | 1.00 | 1.00 | 1.00 | 1.00 | 1.00 | 1.00 | 1.00 | 1.00 | 1.00 | 1.00 | 0.00 | 1.00 | 1.00 | 1.00 | 1.00 | 1.00 | 1.00 | 1.00 | 1.00 |
| GNLY+CD8+_cytotoxic_T_cell\|CD56brightCD16-_NK | 1.00 | 0.00 | 1.00 | 1.00 | 0.00 | 1.00 | 1.00 | 0.35 | 1.00 | 1.00 | 1.00 | 0.00 | 1.00 | 0.00 | 0.00 | 0.00 | 0.00 | 1.00 | 1.00 | 0.00 |
| GNLY+CD8+_cytotoxic_T_cell\|CD56dimCD16+_NK | 1.00 | 1.00 | 1.00 | 1.00 | 0.00 | 1.00 | 1.00 | 1.00 | 1.00 | 1.00 | 0.00 | 0.00 | 1.00 | 0.00 | 0.00 | 0.00 | 1.00 | 1.00 | 1.00 | 0.00 |
| GNLY+CD8+_cytotoxic_T_cell\|CD8+_naÃ¯ve_T_cell | 1.00 | 1.00 | 1.00 | 1.00 | 0.00 | 1.00 | 1.00 | 1.00 | 1.00 | 1.00 | 1.00 | 0.00 | 1.00 | 0.78 | 0.00 | 1.00 | 1.00 | 1.00 | 1.00 | 0.00 |
| GNLY+CD8+_cytotoxic_T_cell\|GNLY+CD8+_cytotoxic_T_cell | 1.00 | 1.00 | 1.00 | 1.00 | 1.00 | 1.00 | 1.00 | 1.00 | 1.00 | 1.00 | 1.00 | 0.00 | 1.00 | 0.00 | 1.00 | 1.00 | 1.00 | 1.00 | 1.00 | 0.00 |
| GNLY+CD8+_cytotoxic_T_cell\|GZMK+CD8+_cytotoxic_T_cell | 0.00 | 1.00 | 1.00 | 1.00 | 1.00 | 1.00 | 1.00 | 1.00 | 1.00 | 1.00 | 1.00 | 0.00 | 1.00 | 1.00 | 1.00 | 1.00 | 1.00 | 1.00 | 1.00 | 0.00 |
| GNLY+CD8+_cytotoxic_T_cell\|MAI_T | 0.00 | 0.00 | 1.00 | 1.00 | 0.00 | 1.00 | 1.00 | 0.03 | 1.00 | 1.00 | 1.00 | 0.00 | 1.00 | 1.00 | 1.00 | 1.00 | 1.00 | 1.00 | 1.00 | 0.03 |
| GNLY+CD8+_cytotoxic_T_cell\|Memory_B_cell | 1.00 | 1.00 | 1.00 | 1.00 | 0.00 | 1.00 | 1.00 | 1.00 | 1.00 | 1.00 | 1.00 | 0.00 | 0.00 | 1.00 | 1.00 | 1.00 | 1.00 | 1.00 | 0.00 | 0.00 |
| GNLY+CD8+_cytotoxic_T_cell\|NC-Mono | 1.00 | 0.00 | 1.00 | 1.00 | 1.00 | 1.00 | 1.00 | 1.00 | 0.97 | 1.00 | 1.00 | 0.00 | 1.00 | 0.00 | 1.00 | 1.00 | 1.00 | 1.00 | 0.00 | 0.31 |
| GNLY+CD8+_cytotoxic_T_cell\|NKT | 1.00 | 1.00 | 1.00 | 1.00 | 0.00 | 1.00 | 1.00 | 1.00 | 1.00 | 1.00 | 0.00 | 0.00 | 1.00 | 0.00 | 0.00 | 0.00 | 0.00 | 1.00 | 1.00 | 0.00 |
| GNLY+CD8+_cytotoxic_T_cell\|NaÃ¯ve_B_cell | 1.00 | 1.00 | 1.00 | 1.00 | 0.00 | 1.00 | 1.00 | 1.00 | 1.00 | 1.00 | 1.00 | 1.00 | 0.00 | 1.00 | 1.00 | 1.00 | 1.00 | 1.00 | 1.00 | 0.00 |
| GNLY+CD8+_cytotoxic_T_cell\|Plasma | 1.00 | 1.00 | 1.00 | 1.00 | 0.00 | 1.00 | 1.00 | 0.00 | 0.97 | 1.00 | 1.00 | 0.00 | 1.00 | 1.00 | 1.00 | 1.00 | 1.00 | 1.00 | 1.00 | 1.00 |
| GNLY+CD8+_cytotoxic_T_cell\|Treg | 1.00 | 1.00 | 1.00 | 1.00 | 1.00 | 1.00 | 1.00 | 1.00 | 1.00 | 1.00 | 1.00 | 0.00 | 1.00 | 1.00 | 1.00 | 1.00 | 1.00 | 1.00 | 1.00 | 0.00 |
| GNLY+CD8+_cytotoxic_T_cell\|γδ_T | 0.00 | 1.00 | 1.00 | 1.00 | 1.00 | 1.00 | 1.00 | 0.44 | 1.00 | 1.00 | 1.00 | 0.00 | 1.00 | 1.00 | 0.00 | 1.00 | 1.00 | 1.00 | 1.00 | 0.00 |
| GZMK+CD8+_cytotoxic_T_cell\|C-Mono1 | 1.00 | 0.00 | 1.00 | 1.00 | 0.00 | 1.00 | 1.00 | 1.00 | 0.00 | 0.00 | 1.00 | 0.00 | 1.00 | 0.00 | 1.00 | 1.00 | 1.00 | 1.00 | 0.00 | 0.94 |
| GZMK+CD8+_cytotoxic_T_cell\|C-Mono2 | 1.00 | 0.00 | 1.00 | 1.00 | 1.00 | 1.00 | 1.00 | 1.00 | 0.00 | 0.00 | 1.00 | 0.00 | 1.00 | 1.00 | 1.00 | 1.00 | 1.00 | 1.00 | 0.00 | 1.00 |
| GZMK+CD8+_cytotoxic_T_cell\|C-Mono3 | 1.00 | 0.00 | 1.00 | 1.00 | 1.00 | 1.00 | 1.00 | 1.00 | 0.00 | 0.00 | 1.00 | 0.00 | 1.00 | 0.00 | 1.00 | 1.00 | 1.00 | 1.00 | 0.00 | 1.00 |
| GZMK+CD8+_cytotoxic_T_cell\|CD4+_effector_T_cell | 1.00 | 1.00 | 1.00 | 1.00 | 1.00 | 1.00 | 1.00 | 0.00 | 0.26 | 1.00 | 1.00 | 0.00 | 1.00 | 1.00 | 1.00 | 1.00 | 1.00 | 1.00 | 1.00 | 0.00 |
| GZMK+CD8+_cytotoxic_T_cell\|CD4+_memory_T_cell | 1.00 | 1.00 | 1.00 | 1.00 | 1.00 | 1.00 | 1.00 | 0.00 | 0.84 | 1.00 | 1.00 | 0.00 | 1.00 | 1.00 | 1.00 | 1.00 | 1.00 | 1.00 | 1.00 | 0.25 |
| GZMK+CD8+_cytotoxic_T_cell\|CD4+_naÃ¯ve_T_cell | 1.00 | 1.00 | 1.00 | 1.00 | 1.00 | 1.00 | 1.00 | 0.00 | 0.97 | 0.06 | 1.00 | 0.00 | 1.00 | 1.00 | 1.00 | 1.00 | 1.00 | 1.00 | 1.00 | 1.00 |
| GZMK+CD8+_cytotoxic_T_cell\|CD56brightCD16-_NK | 1.00 | 0.00 | 1.00 | 1.00 | 0.00 | 1.00 | 1.00 | 0.00 | 0.38 | 1.00 | 1.00 | 0.00 | 1.00 | 0.00 | 0.00 | 0.00 | 0.00 | 1.00 | 1.00 | 0.00 |
| GZMK+CD8+_cytotoxic_T_cell\|CD56dimCD16+_NK | 1.00 | 1.00 | 1.00 | 1.00 | 0.00 | 1.00 | 1.00 | 0.00 | 0.22 | 1.00 | 0.00 | 0.00 | 1.00 | 0.00 | 0.00 | 0.00 | 1.00 | 1.00 | 1.00 | 0.00 |
| GZMK+CD8+_cytotoxic_T_cell\|CD8+_naÃ¯ve_T_cell | 1.00 | 1.00 | 1.00 | 1.00 | 0.00 | 1.00 | 1.00 | 0.00 | 0.00 | 1.00 | 1.00 | 0.00 | 1.00 | 0.00 | 0.00 | 1.00 | 1.00 | 1.00 | 1.00 | 0.00 |
| GZMK+CD8+_cytotoxic_T_cell\|GNLY+CD8+_cytotoxic_T_cell | 1.00 | 1.00 | 1.00 | 1.00 | 1.00 | 1.00 | 1.00 | 0.25 | 0.13 | 1.00 | 1.00 | 0.00 | 1.00 | 0.00 | 1.00 | 1.00 | 1.00 | 1.00 | 1.00 | 0.00 |
| GZMK+CD8+_cytotoxic_T_cell\|GZMK+CD8+_cytotoxic_T_cell | 0.00 | 1.00 | 1.00 | 1.00 | 1.00 | 1.00 | 1.00 | 0.00 | 0.19 | 1.00 | 1.00 | 0.00 | 1.00 | 1.00 | 1.00 | 1.00 | 1.00 | 1.00 | 1.00 | 0.00 |
| GZMK+CD8+_cytotoxic_T_cell\|MAI_T | 0.00 | 0.00 | 1.00 | 1.00 | 0.00 | 1.00 | 1.00 | 0.00 | 0.69 | 1.00 | 1.00 | 0.00 | 1.00 | 1.00 | 1.00 | 1.00 | 1.00 | 1.00 | 1.00 | 0.12 |
| GZMK+CD8+_cytotoxic_T_cell\|Memory_B_cell | 1.00 | 1.00 | 1.00 | 1.00 | 0.00 | 1.00 | 1.00 | 0.00 | 0.49 | 1.00 | 1.00 | 0.15 | 0.00 | 1.00 | 1.00 | 1.00 | 1.00 | 1.00 | 0.00 | 0.00 |
| GZMK+CD8+_cytotoxic_T_cell\|NC-Mono | 1.00 | 0.00 | 1.00 | 1.00 | 1.00 | 1.00 | 1.00 | 1.00 | 0.00 | 0.00 | 1.00 | 0.00 | 1.00 | 0.00 | 1.00 | 1.00 | 1.00 | 1.00 | 0.00 | 1.00 |
| GZMK+CD8+_cytotoxic_T_cell\|NKT | 1.00 | 1.00 | 1.00 | 1.00 | 0.00 | 1.00 | 1.00 | 0.00 | 0.00 | 1.00 | 0.00 | 0.00 | 1.00 | 0.00 | 0.00 | 0.00 | 0.00 | 1.00 | 1.00 | 0.00 |
| GZMK+CD8+_cytotoxic_T_cell\|NaÃ¯ve_B_cell | 1.00 | 1.00 | 1.00 | 1.00 | 0.00 | 1.00 | 1.00 | 0.94 | 1.00 | 0.00 | 1.00 | 1.00 | 0.00 | 1.00 | 1.00 | 1.00 | 1.00 | 1.00 | 1.00 | 0.41 |
| GZMK+CD8+_cytotoxic_T_cell\|Plasma | 1.00 | 1.00 | 1.00 | 1.00 | 0.00 | 1.00 | 1.00 | 0.00 | 0.00 | 0.41 | 1.00 | 0.35 | 1.00 | 1.00 | 1.00 | 1.00 | 1.00 | 1.00 | 1.00 | 1.00 |
| GZMK+CD8+_cytotoxic_T_cell\|Treg | 1.00 | 1.00 | 1.00 | 1.00 | 1.00 | 1.00 | 1.00 | 0.00 | 0.47 | 1.00 | 1.00 | 0.00 | 1.00 | 1.00 | 1.00 | 1.00 | 1.00 | 1.00 | 1.00 | 0.03 |
| GZMK+CD8+_cytotoxic_T_cell\|γδ_T | 0.00 | 1.00 | 1.00 | 1.00 | 1.00 | 1.00 | 1.00 | 0.00 | 0.28 | 1.00 | 1.00 | 0.00 | 1.00 | 1.00 | 0.00 | 1.00 | 1.00 | 1.00 | 1.00 | 0.00 |
| MAI_T\|C-Mono1 | 1.00 | 0.00 | 0.00 | 1.00 | 0.00 | 1.00 | 1.00 | 1.00 | 0.88 | 1.00 | 1.00 | 1.00 | 1.00 | 0.00 | 1.00 | 1.00 | 1.00 | 1.00 | 0.00 | 0.66 |
| MAI_T\|C-Mono2 | 1.00 | 0.00 | 0.00 | 1.00 | 1.00 | 1.00 | 1.00 | 1.00 | 1.00 | 1.00 | 1.00 | 1.00 | 1.00 | 1.00 | 1.00 | 1.00 | 1.00 | 1.00 | 0.00 | 1.00 |
| MAI_T\|C-Mono3 | 1.00 | 0.00 | 0.00 | 1.00 | 1.00 | 1.00 | 1.00 | 1.00 | 1.00 | 1.00 | 1.00 | 1.00 | 1.00 | 0.00 | 1.00 | 1.00 | 1.00 | 1.00 | 0.00 | 1.00 |
| MAI_T\|CD4+_effector_T_cell | 1.00 | 1.00 | 0.94 | 1.00 | 1.00 | 1.00 | 1.00 | 1.00 | 1.00 | 1.00 | 1.00 | 1.00 | 1.00 | 1.00 | 1.00 | 1.00 | 1.00 | 1.00 | 1.00 | 0.00 |
| MAI_T\|CD4+_memory_T_cell | 1.00 | 1.00 | 0.12 | 1.00 | 1.00 | 1.00 | 1.00 | 1.00 | 1.00 | 1.00 | 1.00 | 0.53 | 1.00 | 1.00 | 1.00 | 1.00 | 1.00 | 1.00 | 1.00 | 0.10 |
| MAI_T\|CD4+_naÃ¯ve_T_cell | 1.00 | 1.00 | 0.63 | 1.00 | 1.00 | 1.00 | 1.00 | 1.00 | 1.00 | 1.00 | 1.00 | 1.00 | 1.00 | 1.00 | 1.00 | 1.00 | 1.00 | 1.00 | 1.00 | 0.90 |
| MAI_T\|CD56brightCD16-_NK | 1.00 | 0.00 | 0.00 | 1.00 | 0.00 | 1.00 | 1.00 | 1.00 | 1.00 | 1.00 | 1.00 | 0.97 | 1.00 | 0.00 | 0.00 | 0.00 | 0.00 | 1.00 | 1.00 | 0.00 |
| MAI_T\|CD56dimCD16+_NK | 1.00 | 1.00 | 1.00 | 1.00 | 0.00 | 1.00 | 1.00 | 1.00 | 1.00 | 1.00 | 0.00 | 0.94 | 1.00 | 0.00 | 0.00 | 0.00 | 1.00 | 1.00 | 1.00 | 0.00 |
| MAI_T\|CD8+_naÃ¯ve_T_cell | 1.00 | 1.00 | 0.50 | 1.00 | 0.00 | 1.00 | 1.00 | 1.00 | 1.00 | 1.00 | 1.00 | 0.56 | 1.00 | 0.00 | 0.00 | 1.00 | 1.00 | 1.00 | 1.00 | 0.00 |
| MAI_T\|GNLY+CD8+_cytotoxic_T_cell | 1.00 | 1.00 | 1.00 | 1.00 | 1.00 | 1.00 | 1.00 | 1.00 | 1.00 | 1.00 | 1.00 | 1.00 | 1.00 | 0.00 | 1.00 | 1.00 | 1.00 | 1.00 | 1.00 | 0.00 |
| MAI_T\|GZMK+CD8+_cytotoxic_T_cell | 0.00 | 1.00 | 0.66 | 1.00 | 1.00 | 1.00 | 1.00 | 1.00 | 1.00 | 1.00 | 1.00 | 1.00 | 1.00 | 1.00 | 1.00 | 1.00 | 1.00 | 1.00 | 1.00 | 0.00 |
| MAI_T\|MAI_T | 0.00 | 0.00 | 0.72 | 1.00 | 0.00 | 1.00 | 1.00 | 0.97 | 1.00 | 1.00 | 1.00 | 1.00 | 1.00 | 1.00 | 1.00 | 1.00 | 1.00 | 1.00 | 1.00 | 0.15 |
| MAI_T\|Memory_B_cell | 1.00 | 1.00 | 0.97 | 1.00 | 0.00 | 1.00 | 1.00 | 1.00 | 1.00 | 1.00 | 1.00 | 1.00 | 0.00 | 1.00 | 1.00 | 1.00 | 1.00 | 1.00 | 0.00 | 0.00 |
| MAI_T\|NC-Mono | 1.00 | 0.00 | 0.00 | 1.00 | 1.00 | 1.00 | 1.00 | 1.00 | 1.00 | 1.00 | 1.00 | 1.00 | 1.00 | 0.00 | 1.00 | 1.00 | 1.00 | 1.00 | 0.00 | 0.66 |
| MAI_T\|NKT | 1.00 | 1.00 | 1.00 | 1.00 | 0.00 | 1.00 | 1.00 | 1.00 | 1.00 | 1.00 | 0.00 | 0.47 | 1.00 | 0.00 | 0.00 | 0.00 | 0.00 | 1.00 | 1.00 | 0.00 |
| MAI_T\|NaÃ¯ve_B_cell | 1.00 | 1.00 | 1.00 | 1.00 | 0.00 | 1.00 | 1.00 | 1.00 | 1.00 | 1.00 | 1.00 | 1.00 | 0.00 | 1.00 | 1.00 | 1.00 | 1.00 | 1.00 | 1.00 | 0.13 |
| MAI_T\|Plasma | 1.00 | 1.00 | 1.00 | 1.00 | 0.00 | 1.00 | 1.00 | 0.91 | 1.00 | 1.00 | 1.00 | 1.00 | 1.00 | 1.00 | 1.00 | 1.00 | 1.00 | 1.00 | 1.00 | 1.00 |
| MAI_T\|Treg | 1.00 | 1.00 | 0.00 | 1.00 | 1.00 | 1.00 | 1.00 | 1.00 | 1.00 | 1.00 | 1.00 | 1.00 | 1.00 | 1.00 | 1.00 | 1.00 | 1.00 | 1.00 | 1.00 | 0.00 |
| MAI_T\|γδ_T | 0.00 | 1.00 | 0.79 | 1.00 | 1.00 | 1.00 | 1.00 | 1.00 | 1.00 | 1.00 | 1.00 | 1.00 | 1.00 | 1.00 | 0.00 | 1.00 | 1.00 | 1.00 | 1.00 | 0.00 |
| Memory_B_cell\|C-Mono1 | 1.00 | 1.00 | 1.00 | 1.00 | 1.00 | 1.00 | 1.00 | 0.00 | 0.00 | 0.00 | 1.00 | 1.00 | 1.00 | 1.00 | 1.00 | 1.00 | 1.00 | 1.00 | 0.00 | 1.00 |
| Memory_B_cell\|C-Mono2 | 1.00 | 1.00 | 1.00 | 1.00 | 1.00 | 1.00 | 1.00 | 0.00 | 0.00 | 0.00 | 1.00 | 1.00 | 1.00 | 1.00 | 1.00 | 1.00 | 1.00 | 1.00 | 0.00 | 1.00 |
| Memory_B_cell\|C-Mono3 | 1.00 | 1.00 | 1.00 | 1.00 | 1.00 | 1.00 | 1.00 | 0.00 | 0.00 | 0.00 | 1.00 | 1.00 | 1.00 | 1.00 | 1.00 | 1.00 | 1.00 | 1.00 | 0.00 | 1.00 |
| Memory_B_cell\|CD4+_effector_T_cell | 1.00 | 1.00 | 1.00 | 1.00 | 1.00 | 1.00 | 1.00 | 0.00 | 0.00 | 1.00 | 1.00 | 1.00 | 1.00 | 1.00 | 1.00 | 1.00 | 1.00 | 1.00 | 1.00 | 0.00 |
| Memory_B_cell\|CD4+_memory_T_cell | 1.00 | 1.00 | 1.00 | 1.00 | 1.00 | 1.00 | 1.00 | 0.00 | 0.00 | 1.00 | 1.00 | 1.00 | 1.00 | 1.00 | 1.00 | 1.00 | 1.00 | 1.00 | 1.00 | 1.00 |
| Memory_B_cell\|CD4+_naÃ¯ve_T_cell | 1.00 | 1.00 | 1.00 | 1.00 | 1.00 | 1.00 | 1.00 | 0.00 | 0.00 | 0.00 | 1.00 | 1.00 | 1.00 | 1.00 | 1.00 | 1.00 | 1.00 | 1.00 | 1.00 | 1.00 |
| Memory_B_cell\|CD56brightCD16-_NK | 1.00 | 1.00 | 1.00 | 1.00 | 1.00 | 1.00 | 1.00 | 0.00 | 0.00 | 1.00 | 1.00 | 1.00 | 1.00 | 1.00 | 0.00 | 0.00 | 0.00 | 1.00 | 1.00 | 0.03 |
| Memory_B_cell\|CD56dimCD16+_NK | 1.00 | 1.00 | 1.00 | 1.00 | 1.00 | 1.00 | 1.00 | 0.00 | 0.00 | 1.00 | 0.00 | 1.00 | 1.00 | 0.97 | 0.00 | 0.00 | 1.00 | 1.00 | 1.00 | 0.00 |
| Memory_B_cell\|CD8+_naÃ¯ve_T_cell | 1.00 | 1.00 | 1.00 | 1.00 | 1.00 | 1.00 | 1.00 | 0.00 | 0.00 | 1.00 | 1.00 | 1.00 | 1.00 | 1.00 | 0.03 | 1.00 | 1.00 | 1.00 | 1.00 | 0.38 |
| Memory_B_cell\|GNLY+CD8+_cytotoxic_T_cell | 1.00 | 1.00 | 1.00 | 1.00 | 1.00 | 1.00 | 1.00 | 0.00 | 0.00 | 1.00 | 1.00 | 1.00 | 1.00 | 1.00 | 1.00 | 1.00 | 1.00 | 1.00 | 1.00 | 0.00 |
| Memory_B_cell\|GZMK+CD8+_cytotoxic_T_cell | 1.00 | 1.00 | 1.00 | 1.00 | 1.00 | 1.00 | 1.00 | 0.00 | 0.00 | 1.00 | 1.00 | 1.00 | 1.00 | 1.00 | 1.00 | 1.00 | 1.00 | 1.00 | 1.00 | 0.03 |
| Memory_B_cell\|MAI_T | 1.00 | 1.00 | 1.00 | 1.00 | 1.00 | 1.00 | 1.00 | 0.00 | 0.00 | 1.00 | 1.00 | 1.00 | 1.00 | 1.00 | 1.00 | 1.00 | 1.00 | 1.00 | 1.00 | 0.66 |
| Memory_B_cell\|Memory_B_cell | 1.00 | 1.00 | 1.00 | 1.00 | 0.13 | 1.00 | 1.00 | 0.00 | 0.00 | 1.00 | 1.00 | 1.00 | 1.00 | 1.00 | 1.00 | 1.00 | 1.00 | 1.00 | 0.00 | 0.00 |
| Memory_B_cell\|NC-Mono | 1.00 | 1.00 | 1.00 | 1.00 | 1.00 | 1.00 | 1.00 | 0.00 | 0.00 | 0.00 | 1.00 | 1.00 | 1.00 | 1.00 | 1.00 | 1.00 | 1.00 | 1.00 | 0.00 | 1.00 |
| Memory_B_cell\|NKT | 1.00 | 1.00 | 1.00 | 1.00 | 1.00 | 1.00 | 1.00 | 0.00 | 0.00 | 1.00 | 0.00 | 1.00 | 1.00 | 1.00 | 0.00 | 0.00 | 0.00 | 1.00 | 1.00 | 0.00 |
| Memory_B_cell\|NaÃ¯ve_B_cell | 1.00 | 1.00 | 1.00 | 1.00 | 0.00 | 1.00 | 1.00 | 0.00 | 0.00 | 0.00 | 1.00 | 1.00 | 0.00 | 1.00 | 1.00 | 1.00 | 1.00 | 1.00 | 1.00 | 1.00 |
| Memory_B_cell\|Plasma | 1.00 | 1.00 | 1.00 | 1.00 | 0.00 | 1.00 | 1.00 | 0.00 | 0.00 | 0.00 | 1.00 | 1.00 | 1.00 | 1.00 | 1.00 | 1.00 | 1.00 | 1.00 | 1.00 | 1.00 |
| Memory_B_cell\|Treg | 1.00 | 1.00 | 1.00 | 1.00 | 1.00 | 1.00 | 1.00 | 0.00 | 0.00 | 1.00 | 1.00 | 1.00 | 1.00 | 1.00 | 1.00 | 1.00 | 1.00 | 1.00 | 1.00 | 0.72 |
| Memory_B_cell\|γδ_T | 1.00 | 1.00 | 1.00 | 1.00 | 1.00 | 1.00 | 1.00 | 0.00 | 0.00 | 1.00 | 1.00 | 1.00 | 1.00 | 1.00 | 0.00 | 1.00 | 1.00 | 1.00 | 1.00 | 0.03 |
| NC-Mono\|C-Mono1 | 1.00 | 1.00 | 0.00 | 1.00 | 0.78 | 1.00 | 1.00 | 0.00 | 0.00 | 0.00 | 1.00 | 0.00 | 1.00 | 0.00 | 1.00 | 1.00 | 1.00 | 0.00 | 0.15 | 0.00 |
| NC-Mono\|C-Mono2 | 1.00 | 1.00 | 0.00 | 1.00 | 1.00 | 1.00 | 1.00 | 0.00 | 0.00 | 0.00 | 1.00 | 0.00 | 1.00 | 1.00 | 1.00 | 1.00 | 1.00 | 0.00 | 1.00 | 1.00 |
| NC-Mono\|C-Mono3 | 1.00 | 1.00 | 0.00 | 1.00 | 1.00 | 1.00 | 1.00 | 0.00 | 0.00 | 0.00 | 1.00 | 0.00 | 1.00 | 0.00 | 1.00 | 1.00 | 1.00 | 0.00 | 1.00 | 1.00 |
| NC-Mono\|CD4+_effector_T_cell | 1.00 | 1.00 | 0.00 | 1.00 | 1.00 | 1.00 | 1.00 | 0.00 | 0.00 | 1.00 | 1.00 | 0.00 | 1.00 | 1.00 | 1.00 | 1.00 | 1.00 | 0.00 | 1.00 | 0.00 |
| NC-Mono\|CD4+_memory_T_cell | 1.00 | 1.00 | 0.00 | 1.00 | 1.00 | 1.00 | 1.00 | 0.00 | 0.00 | 1.00 | 1.00 | 0.00 | 1.00 | 1.00 | 1.00 | 1.00 | 1.00 | 0.00 | 1.00 | 0.00 |
| NC-Mono\|CD4+_naÃ¯ve_T_cell | 1.00 | 1.00 | 0.00 | 1.00 | 1.00 | 1.00 | 1.00 | 0.00 | 0.00 | 0.00 | 1.00 | 0.00 | 1.00 | 1.00 | 1.00 | 1.00 | 1.00 | 0.00 | 1.00 | 0.03 |
| NC-Mono\|CD56brightCD16-_NK | 1.00 | 1.00 | 0.00 | 1.00 | 0.28 | 1.00 | 1.00 | 0.00 | 0.00 | 1.00 | 1.00 | 0.00 | 1.00 | 0.00 | 0.00 | 0.00 | 0.00 | 0.00 | 1.00 | 0.00 |
| NC-Mono\|CD56dimCD16+_NK | 1.00 | 1.00 | 0.00 | 1.00 | 0.06 | 1.00 | 1.00 | 0.00 | 0.00 | 1.00 | 0.00 | 0.00 | 1.00 | 0.00 | 0.00 | 0.00 | 1.00 | 0.00 | 1.00 | 0.00 |
| NC-Mono\|CD8+_naÃ¯ve_T_cell | 1.00 | 1.00 | 0.00 | 1.00 | 0.68 | 1.00 | 1.00 | 0.00 | 0.00 | 1.00 | 1.00 | 0.00 | 1.00 | 0.00 | 0.00 | 1.00 | 1.00 | 0.00 | 1.00 | 0.00 |
| NC-Mono\|GNLY+CD8+_cytotoxic_T_cell | 1.00 | 1.00 | 0.00 | 1.00 | 1.00 | 1.00 | 1.00 | 0.00 | 0.00 | 1.00 | 1.00 | 0.00 | 1.00 | 0.00 | 1.00 | 1.00 | 1.00 | 0.00 | 1.00 | 0.00 |
| NC-Mono\|GZMK+CD8+_cytotoxic_T_cell | 1.00 | 1.00 | 0.00 | 1.00 | 1.00 | 1.00 | 1.00 | 0.00 | 0.00 | 1.00 | 1.00 | 0.00 | 1.00 | 1.00 | 1.00 | 1.00 | 1.00 | 0.00 | 1.00 | 0.00 |
| NC-Mono\|MAI_T | 1.00 | 1.00 | 0.00 | 1.00 | 0.25 | 1.00 | 1.00 | 0.00 | 0.00 | 1.00 | 1.00 | 0.00 | 1.00 | 1.00 | 1.00 | 1.00 | 1.00 | 0.00 | 1.00 | 0.00 |
| NC-Mono\|Memory_B_cell | 1.00 | 1.00 | 0.00 | 1.00 | 0.00 | 1.00 | 1.00 | 0.00 | 0.00 | 1.00 | 1.00 | 0.00 | 0.00 | 1.00 | 1.00 | 1.00 | 1.00 | 0.00 | 1.00 | 0.00 |
| NC-Mono\|NC-Mono | 1.00 | 1.00 | 0.00 | 1.00 | 1.00 | 1.00 | 1.00 | 0.00 | 0.00 | 0.00 | 1.00 | 0.00 | 1.00 | 0.00 | 1.00 | 1.00 | 1.00 | 0.00 | 0.00 | 0.00 |
| NC-Mono\|NKT | 1.00 | 1.00 | 0.00 | 1.00 | 0.22 | 1.00 | 1.00 | 0.00 | 0.00 | 1.00 | 0.00 | 0.00 | 1.00 | 0.00 | 0.00 | 0.00 | 0.00 | 0.00 | 1.00 | 0.00 |
| NC-Mono\|NaÃ¯ve_B_cell | 1.00 | 1.00 | 0.00 | 1.00 | 0.00 | 1.00 | 1.00 | 0.00 | 0.00 | 0.00 | 1.00 | 1.00 | 0.00 | 1.00 | 1.00 | 1.00 | 1.00 | 0.00 | 1.00 | 0.00 |
| NC-Mono\|Plasma | 1.00 | 1.00 | 0.00 | 1.00 | 0.00 | 1.00 | 1.00 | 0.00 | 0.00 | 0.00 | 1.00 | 0.00 | 1.00 | 1.00 | 1.00 | 1.00 | 1.00 | 0.00 | 1.00 | 0.94 |
| NC-Mono\|Treg | 1.00 | 1.00 | 0.00 | 1.00 | 1.00 | 1.00 | 1.00 | 0.00 | 0.00 | 1.00 | 1.00 | 0.00 | 1.00 | 1.00 | 1.00 | 1.00 | 1.00 | 0.00 | 1.00 | 0.00 |
| NC-Mono\|γδ_T | 1.00 | 1.00 | 0.00 | 1.00 | 1.00 | 1.00 | 1.00 | 0.00 | 0.00 | 1.00 | 1.00 | 0.00 | 1.00 | 1.00 | 0.00 | 1.00 | 1.00 | 0.00 | 1.00 | 0.00 |
| NKT\|C-Mono1 | 1.00 | 0.00 | 0.00 | 1.00 | 0.00 | 1.00 | 1.00 | 1.00 | 0.00 | 0.10 | 1.00 | 0.00 | 1.00 | 0.09 | 1.00 | 1.00 | 1.00 | 1.00 | 0.00 | 0.00 |
| NKT\|C-Mono2 | 1.00 | 0.00 | 0.00 | 1.00 | 1.00 | 1.00 | 1.00 | 1.00 | 0.13 | 0.25 | 1.00 | 0.00 | 1.00 | 1.00 | 1.00 | 1.00 | 1.00 | 1.00 | 0.00 | 1.00 |
| NKT\|C-Mono3 | 1.00 | 0.00 | 0.00 | 1.00 | 1.00 | 0.00 | 1.00 | 1.00 | 0.06 | 0.13 | 1.00 | 0.00 | 1.00 | 0.31 | 1.00 | 1.00 | 1.00 | 1.00 | 0.00 | 1.00 |
| NKT\|CD4+_effector_T_cell | 1.00 | 1.00 | 0.00 | 1.00 | 1.00 | 0.00 | 0.00 | 0.97 | 1.00 | 1.00 | 1.00 | 0.00 | 1.00 | 1.00 | 1.00 | 1.00 | 1.00 | 1.00 | 1.00 | 0.00 |
| NKT\|CD4+_memory_T_cell | 1.00 | 1.00 | 0.00 | 1.00 | 1.00 | 0.00 | 0.00 | 0.40 | 1.00 | 1.00 | 1.00 | 0.00 | 1.00 | 1.00 | 1.00 | 1.00 | 1.00 | 1.00 | 1.00 | 0.00 |
| NKT\|CD4+_naÃ¯ve_T_cell | 1.00 | 1.00 | 0.00 | 1.00 | 1.00 | 0.00 | 1.00 | 0.81 | 1.00 | 1.00 | 1.00 | 0.00 | 1.00 | 1.00 | 1.00 | 1.00 | 1.00 | 1.00 | 1.00 | 0.00 |
| NKT\|CD56brightCD16-_NK | 1.00 | 0.00 | 0.00 | 1.00 | 0.00 | 0.00 | 0.00 | 0.25 | 1.00 | 1.00 | 1.00 | 0.00 | 1.00 | 0.00 | 0.00 | 0.00 | 0.00 | 1.00 | 1.00 | 0.00 |
| NKT\|CD56dimCD16+_NK | 1.00 | 1.00 | 0.06 | 1.00 | 0.00 | 0.00 | 0.00 | 0.94 | 1.00 | 1.00 | 0.00 | 0.00 | 1.00 | 0.00 | 0.00 | 0.00 | 1.00 | 1.00 | 1.00 | 0.00 |
| NKT\|CD8+_naÃ¯ve_T_cell | 1.00 | 1.00 | 0.00 | 1.00 | 0.00 | 0.00 | 0.00 | 0.69 | 0.94 | 1.00 | 1.00 | 0.00 | 1.00 | 0.37 | 0.00 | 1.00 | 1.00 | 1.00 | 1.00 | 0.00 |
| NKT\|GNLY+CD8+_cytotoxic_T_cell | 1.00 | 1.00 | 0.00 | 1.00 | 1.00 | 0.00 | 0.00 | 1.00 | 1.00 | 1.00 | 1.00 | 0.00 | 1.00 | 0.06 | 1.00 | 1.00 | 1.00 | 1.00 | 1.00 | 0.00 |
| NKT\|GZMK+CD8+_cytotoxic_T_cell | 0.00 | 1.00 | 0.00 | 1.00 | 1.00 | 0.00 | 0.00 | 0.72 | 1.00 | 1.00 | 1.00 | 0.00 | 1.00 | 1.00 | 1.00 | 1.00 | 1.00 | 1.00 | 1.00 | 0.00 |
| NKT\|MAI_T | 0.00 | 0.00 | 0.00 | 1.00 | 0.00 | 0.00 | 0.00 | 0.06 | 1.00 | 1.00 | 1.00 | 0.00 | 1.00 | 1.00 | 1.00 | 1.00 | 1.00 | 1.00 | 1.00 | 0.00 |
| NKT\|Memory_B_cell | 1.00 | 1.00 | 0.00 | 1.00 | 0.00 | 0.00 | 0.00 | 0.84 | 1.00 | 1.00 | 1.00 | 0.06 | 0.00 | 1.00 | 1.00 | 1.00 | 1.00 | 1.00 | 0.00 | 0.00 |
| NKT\|NC-Mono | 1.00 | 0.00 | 0.00 | 1.00 | 1.00 | 0.00 | 0.00 | 1.00 | 0.56 | 0.94 | 1.00 | 0.00 | 1.00 | 0.03 | 1.00 | 1.00 | 1.00 | 1.00 | 0.00 | 0.00 |
| NKT\|NKT | 1.00 | 1.00 | 0.00 | 1.00 | 0.00 | 0.00 | 0.00 | 0.59 | 0.72 | 1.00 | 0.00 | 0.00 | 1.00 | 0.00 | 0.00 | 0.00 | 0.00 | 1.00 | 1.00 | 0.00 |
| NKT\|NaÃ¯ve_B_cell | 1.00 | 1.00 | 0.00 | 1.00 | 0.00 | 0.88 | 1.00 | 1.00 | 1.00 | 0.90 | 1.00 | 1.00 | 0.00 | 1.00 | 1.00 | 1.00 | 1.00 | 1.00 | 1.00 | 0.00 |
| NKT\|Plasma | 1.00 | 1.00 | 0.00 | 1.00 | 0.00 | 1.00 | 1.00 | 0.03 | 0.53 | 1.00 | 1.00 | 0.10 | 1.00 | 1.00 | 1.00 | 1.00 | 1.00 | 1.00 | 1.00 | 0.38 |
| NKT\|Treg | 1.00 | 1.00 | 0.00 | 1.00 | 1.00 | 0.00 | 0.00 | 0.47 | 1.00 | 1.00 | 1.00 | 0.00 | 1.00 | 1.00 | 1.00 | 1.00 | 1.00 | 1.00 | 1.00 | 0.00 |
| NKT\|γδ_T | 0.00 | 1.00 | 0.00 | 1.00 | 1.00 | 0.00 | 0.00 | 0.22 | 1.00 | 1.00 | 1.00 | 0.00 | 1.00 | 1.00 | 0.00 | 1.00 | 1.00 | 1.00 | 1.00 | 0.00 |
| NaÃ¯ve_B_cell\|C-Mono1 | 1.00 | 1.00 | 1.00 | 1.00 | 1.00 | 1.00 | 1.00 | 0.00 | 0.00 | 0.00 | 1.00 | 1.00 | 1.00 | 1.00 | 1.00 | 1.00 | 1.00 | 1.00 | 0.00 | 1.00 |
| NaÃ¯ve_B_cell\|C-Mono2 | 1.00 | 1.00 | 1.00 | 1.00 | 1.00 | 1.00 | 1.00 | 0.00 | 0.00 | 0.00 | 1.00 | 1.00 | 1.00 | 1.00 | 1.00 | 1.00 | 1.00 | 1.00 | 0.00 | 1.00 |
| NaÃ¯ve_B_cell\|C-Mono3 | 1.00 | 1.00 | 1.00 | 1.00 | 1.00 | 1.00 | 1.00 | 0.00 | 0.00 | 0.00 | 1.00 | 1.00 | 1.00 | 1.00 | 1.00 | 1.00 | 1.00 | 1.00 | 0.00 | 1.00 |
| NaÃ¯ve_B_cell\|CD4+_effector_T_cell | 1.00 | 1.00 | 1.00 | 1.00 | 1.00 | 1.00 | 1.00 | 0.00 | 0.00 | 1.00 | 1.00 | 1.00 | 1.00 | 1.00 | 1.00 | 1.00 | 1.00 | 1.00 | 1.00 | 0.03 |
| NaÃ¯ve_B_cell\|CD4+_memory_T_cell | 1.00 | 1.00 | 1.00 | 1.00 | 1.00 | 1.00 | 1.00 | 0.00 | 0.00 | 1.00 | 1.00 | 1.00 | 1.00 | 1.00 | 1.00 | 1.00 | 1.00 | 1.00 | 1.00 | 1.00 |
| NaÃ¯ve_B_cell\|CD4+_naÃ¯ve_T_cell | 1.00 | 1.00 | 1.00 | 1.00 | 1.00 | 1.00 | 1.00 | 0.00 | 0.00 | 0.00 | 1.00 | 1.00 | 1.00 | 1.00 | 1.00 | 1.00 | 1.00 | 1.00 | 1.00 | 1.00 |
| NaÃ¯ve_B_cell\|CD56brightCD16-_NK | 1.00 | 1.00 | 1.00 | 1.00 | 1.00 | 1.00 | 1.00 | 0.00 | 0.00 | 1.00 | 1.00 | 1.00 | 1.00 | 1.00 | 0.00 | 0.00 | 1.00 | 1.00 | 1.00 | 0.47 |
| NaÃ¯ve_B_cell\|CD56dimCD16+_NK | 1.00 | 1.00 | 1.00 | 1.00 | 1.00 | 1.00 | 1.00 | 0.00 | 0.00 | 1.00 | 1.00 | 1.00 | 1.00 | 1.00 | 0.00 | 0.97 | 1.00 | 1.00 | 1.00 | 0.00 |
| NaÃ¯ve_B_cell\|CD8+_naÃ¯ve_T_cell | 1.00 | 1.00 | 1.00 | 1.00 | 1.00 | 1.00 | 1.00 | 0.00 | 0.00 | 1.00 | 1.00 | 1.00 | 1.00 | 1.00 | 1.00 | 1.00 | 1.00 | 1.00 | 1.00 | 1.00 |
| NaÃ¯ve_B_cell\|GNLY+CD8+_cytotoxic_T_cell | 1.00 | 1.00 | 1.00 | 1.00 | 1.00 | 1.00 | 1.00 | 0.00 | 0.00 | 1.00 | 1.00 | 1.00 | 1.00 | 1.00 | 1.00 | 1.00 | 1.00 | 1.00 | 1.00 | 0.34 |
| NaÃ¯ve_B_cell\|GZMK+CD8+_cytotoxic_T_cell | 1.00 | 1.00 | 1.00 | 1.00 | 1.00 | 1.00 | 1.00 | 0.00 | 0.00 | 1.00 | 1.00 | 1.00 | 1.00 | 1.00 | 1.00 | 1.00 | 1.00 | 1.00 | 1.00 | 1.00 |
| NaÃ¯ve_B_cell\|MAI_T | 1.00 | 1.00 | 1.00 | 1.00 | 1.00 | 1.00 | 1.00 | 0.00 | 0.00 | 1.00 | 1.00 | 1.00 | 1.00 | 1.00 | 1.00 | 1.00 | 1.00 | 1.00 | 1.00 | 1.00 |
| NaÃ¯ve_B_cell\|Memory_B_cell | 1.00 | 1.00 | 1.00 | 1.00 | 1.00 | 1.00 | 1.00 | 0.00 | 0.00 | 1.00 | 1.00 | 1.00 | 0.32 | 1.00 | 1.00 | 1.00 | 1.00 | 1.00 | 0.00 | 0.06 |
| NaÃ¯ve_B_cell\|NC-Mono | 1.00 | 1.00 | 1.00 | 1.00 | 1.00 | 1.00 | 1.00 | 0.00 | 0.00 | 0.00 | 1.00 | 1.00 | 1.00 | 1.00 | 1.00 | 1.00 | 1.00 | 1.00 | 0.00 | 1.00 |
| NaÃ¯ve_B_cell\|NKT | 1.00 | 1.00 | 1.00 | 1.00 | 1.00 | 1.00 | 1.00 | 0.00 | 0.00 | 1.00 | 1.00 | 1.00 | 1.00 | 1.00 | 0.88 | 1.00 | 1.00 | 1.00 | 1.00 | 0.00 |
| NaÃ¯ve_B_cell\|NaÃ¯ve_B_cell | 1.00 | 1.00 | 1.00 | 1.00 | 1.00 | 1.00 | 1.00 | 0.00 | 0.00 | 0.00 | 1.00 | 1.00 | 0.00 | 1.00 | 1.00 | 1.00 | 1.00 | 1.00 | 1.00 | 1.00 |
| NaÃ¯ve_B_cell\|Plasma | 1.00 | 1.00 | 1.00 | 1.00 | 1.00 | 1.00 | 1.00 | 0.00 | 0.00 | 0.00 | 1.00 | 1.00 | 1.00 | 1.00 | 1.00 | 1.00 | 1.00 | 1.00 | 1.00 | 1.00 |
| NaÃ¯ve_B_cell\|Treg | 1.00 | 1.00 | 1.00 | 1.00 | 1.00 | 1.00 | 1.00 | 0.00 | 0.00 | 1.00 | 1.00 | 1.00 | 1.00 | 1.00 | 1.00 | 1.00 | 1.00 | 1.00 | 1.00 | 1.00 |
| NaÃ¯ve_B_cell\|γδ_T | 1.00 | 1.00 | 1.00 | 1.00 | 1.00 | 1.00 | 1.00 | 0.00 | 0.00 | 1.00 | 1.00 | 1.00 | 1.00 | 1.00 | 0.00 | 1.00 | 1.00 | 1.00 | 1.00 | 1.00 |
| Plasma\|C-Mono1 | 1.00 | 1.00 | 0.79 | 1.00 | 0.62 | 1.00 | 1.00 | 0.00 | 0.00 | 0.00 | 1.00 | 1.00 | 1.00 | 1.00 | 1.00 | 1.00 | 1.00 | 1.00 | 1.00 | 0.00 |
| Plasma\|C-Mono2 | 1.00 | 1.00 | 0.97 | 1.00 | 1.00 | 1.00 | 1.00 | 0.00 | 0.00 | 0.00 | 1.00 | 1.00 | 1.00 | 1.00 | 1.00 | 1.00 | 1.00 | 1.00 | 1.00 | 1.00 |
| Plasma\|C-Mono3 | 1.00 | 1.00 | 0.72 | 1.00 | 1.00 | 1.00 | 1.00 | 0.16 | 0.00 | 0.00 | 1.00 | 1.00 | 1.00 | 1.00 | 1.00 | 1.00 | 1.00 | 1.00 | 1.00 | 1.00 |
| Plasma\|CD4+_effector_T_cell | 1.00 | 1.00 | 1.00 | 1.00 | 1.00 | 1.00 | 1.00 | 0.00 | 0.00 | 1.00 | 1.00 | 1.00 | 1.00 | 1.00 | 1.00 | 1.00 | 1.00 | 1.00 | 1.00 | 0.00 |
| Plasma\|CD4+_memory_T_cell | 1.00 | 1.00 | 1.00 | 1.00 | 1.00 | 1.00 | 1.00 | 0.00 | 0.00 | 1.00 | 1.00 | 1.00 | 1.00 | 1.00 | 1.00 | 1.00 | 1.00 | 1.00 | 1.00 | 0.00 |
| Plasma\|CD4+_naÃ¯ve_T_cell | 1.00 | 1.00 | 1.00 | 1.00 | 1.00 | 1.00 | 1.00 | 0.00 | 0.00 | 0.00 | 1.00 | 1.00 | 1.00 | 1.00 | 1.00 | 1.00 | 1.00 | 1.00 | 1.00 | 0.00 |
| Plasma\|CD56brightCD16-_NK | 1.00 | 1.00 | 1.00 | 1.00 | 0.34 | 1.00 | 1.00 | 0.00 | 0.00 | 1.00 | 1.00 | 1.00 | 1.00 | 1.00 | 0.00 | 1.00 | 1.00 | 1.00 | 1.00 | 0.00 |
| Plasma\|CD56dimCD16+_NK | 1.00 | 1.00 | 1.00 | 1.00 | 0.09 | 1.00 | 1.00 | 0.00 | 0.00 | 1.00 | 0.22 | 1.00 | 1.00 | 1.00 | 1.00 | 1.00 | 1.00 | 1.00 | 1.00 | 0.00 |
| Plasma\|CD8+_naÃ¯ve_T_cell | 1.00 | 1.00 | 1.00 | 1.00 | 0.53 | 1.00 | 1.00 | 0.00 | 0.00 | 1.00 | 1.00 | 1.00 | 1.00 | 1.00 | 1.00 | 1.00 | 1.00 | 1.00 | 1.00 | 0.00 |
| Plasma\|GNLY+CD8+_cytotoxic_T_cell | 1.00 | 1.00 | 1.00 | 1.00 | 1.00 | 1.00 | 1.00 | 0.00 | 0.00 | 1.00 | 1.00 | 1.00 | 1.00 | 1.00 | 1.00 | 1.00 | 1.00 | 1.00 | 1.00 | 0.00 |
| Plasma\|GZMK+CD8+_cytotoxic_T_cell | 1.00 | 1.00 | 1.00 | 1.00 | 1.00 | 1.00 | 1.00 | 0.00 | 0.00 | 1.00 | 1.00 | 1.00 | 1.00 | 1.00 | 1.00 | 1.00 | 1.00 | 1.00 | 1.00 | 0.00 |
| Plasma\|MAI_T | 1.00 | 1.00 | 1.00 | 1.00 | 0.28 | 1.00 | 1.00 | 0.00 | 0.00 | 1.00 | 1.00 | 1.00 | 1.00 | 1.00 | 1.00 | 1.00 | 1.00 | 1.00 | 1.00 | 0.00 |
| Plasma\|Memory_B_cell | 1.00 | 1.00 | 1.00 | 1.00 | 0.00 | 1.00 | 1.00 | 0.00 | 0.00 | 1.00 | 1.00 | 1.00 | 1.00 | 1.00 | 1.00 | 1.00 | 1.00 | 1.00 | 1.00 | 0.00 |
| Plasma\|NC-Mono | 1.00 | 1.00 | 1.00 | 1.00 | 1.00 | 1.00 | 1.00 | 0.00 | 0.00 | 0.00 | 1.00 | 1.00 | 1.00 | 1.00 | 1.00 | 1.00 | 1.00 | 1.00 | 1.00 | 0.00 |
| Plasma\|NKT | 1.00 | 1.00 | 1.00 | 1.00 | 0.15 | 1.00 | 1.00 | 0.00 | 0.00 | 1.00 | 0.71 | 1.00 | 1.00 | 1.00 | 1.00 | 1.00 | 1.00 | 1.00 | 1.00 | 0.00 |
| Plasma\|NaÃ¯ve_B_cell | 1.00 | 1.00 | 1.00 | 1.00 | 0.00 | 1.00 | 1.00 | 0.00 | 0.00 | 0.00 | 1.00 | 1.00 | 1.00 | 1.00 | 1.00 | 1.00 | 1.00 | 1.00 | 1.00 | 0.00 |
| Plasma\|Plasma | 1.00 | 1.00 | 1.00 | 1.00 | 0.00 | 1.00 | 1.00 | 0.00 | 0.00 | 0.00 | 1.00 | 1.00 | 1.00 | 1.00 | 1.00 | 1.00 | 1.00 | 1.00 | 1.00 | 0.60 |
| Plasma\|Treg | 1.00 | 1.00 | 1.00 | 1.00 | 1.00 | 1.00 | 1.00 | 0.00 | 0.00 | 1.00 | 1.00 | 1.00 | 1.00 | 1.00 | 1.00 | 1.00 | 1.00 | 1.00 | 1.00 | 0.00 |
| Plasma\|γδ_T | 1.00 | 1.00 | 1.00 | 1.00 | 1.00 | 1.00 | 1.00 | 0.00 | 0.00 | 1.00 | 1.00 | 1.00 | 1.00 | 1.00 | 0.94 | 1.00 | 1.00 | 1.00 | 1.00 | 0.00 |
| Treg\|C-Mono1 | 1.00 | 1.00 | 0.91 | 1.00 | 0.31 | 1.00 | 1.00 | 1.00 | 1.00 | 1.00 | 1.00 | 0.81 | 1.00 | 0.53 | 1.00 | 1.00 | 1.00 | 1.00 | 0.00 | 0.97 |
| Treg\|C-Mono2 | 1.00 | 1.00 | 1.00 | 1.00 | 1.00 | 1.00 | 1.00 | 1.00 | 1.00 | 1.00 | 1.00 | 1.00 | 1.00 | 1.00 | 1.00 | 1.00 | 1.00 | 1.00 | 0.00 | 1.00 |
| Treg\|C-Mono3 | 1.00 | 1.00 | 0.87 | 1.00 | 1.00 | 1.00 | 1.00 | 1.00 | 1.00 | 1.00 | 1.00 | 0.25 | 1.00 | 0.91 | 1.00 | 1.00 | 1.00 | 1.00 | 0.00 | 1.00 |
| Treg\|CD4+_effector_T_cell | 1.00 | 1.00 | 1.00 | 1.00 | 1.00 | 1.00 | 1.00 | 1.00 | 1.00 | 1.00 | 1.00 | 0.00 | 1.00 | 1.00 | 1.00 | 1.00 | 1.00 | 1.00 | 1.00 | 0.00 |
| Treg\|CD4+_memory_T_cell | 1.00 | 1.00 | 1.00 | 1.00 | 1.00 | 1.00 | 1.00 | 1.00 | 1.00 | 1.00 | 1.00 | 0.00 | 1.00 | 1.00 | 1.00 | 1.00 | 1.00 | 1.00 | 1.00 | 0.70 |
| Treg\|CD4+_naÃ¯ve_T_cell | 1.00 | 1.00 | 1.00 | 1.00 | 1.00 | 1.00 | 1.00 | 1.00 | 1.00 | 1.00 | 1.00 | 0.00 | 1.00 | 1.00 | 1.00 | 1.00 | 1.00 | 1.00 | 1.00 | 1.00 |
| Treg\|CD56brightCD16-_NK | 1.00 | 1.00 | 1.00 | 1.00 | 0.19 | 1.00 | 1.00 | 1.00 | 1.00 | 1.00 | 1.00 | 0.00 | 1.00 | 0.03 | 0.00 | 0.00 | 0.00 | 1.00 | 1.00 | 0.00 |
| Treg\|CD56dimCD16+_NK | 1.00 | 1.00 | 1.00 | 1.00 | 0.00 | 1.00 | 1.00 | 1.00 | 1.00 | 1.00 | 0.88 | 0.00 | 1.00 | 0.00 | 0.00 | 0.00 | 1.00 | 1.00 | 1.00 | 0.00 |
| Treg\|CD8+_naÃ¯ve_T_cell | 1.00 | 1.00 | 1.00 | 1.00 | 0.31 | 1.00 | 1.00 | 1.00 | 1.00 | 1.00 | 1.00 | 0.00 | 1.00 | 0.97 | 0.00 | 1.00 | 1.00 | 1.00 | 1.00 | 0.03 |
| Treg\|GNLY+CD8+_cytotoxic_T_cell | 1.00 | 1.00 | 1.00 | 1.00 | 1.00 | 1.00 | 1.00 | 1.00 | 1.00 | 1.00 | 1.00 | 0.00 | 1.00 | 0.41 | 1.00 | 1.00 | 1.00 | 1.00 | 1.00 | 0.00 |
| Treg\|GZMK+CD8+_cytotoxic_T_cell | 1.00 | 1.00 | 1.00 | 1.00 | 1.00 | 1.00 | 1.00 | 1.00 | 1.00 | 1.00 | 1.00 | 0.00 | 1.00 | 1.00 | 1.00 | 1.00 | 1.00 | 1.00 | 1.00 | 0.00 |
| Treg\|MAI_T | 1.00 | 1.00 | 1.00 | 1.00 | 0.13 | 1.00 | 1.00 | 1.00 | 1.00 | 1.00 | 1.00 | 0.40 | 1.00 | 1.00 | 1.00 | 1.00 | 1.00 | 1.00 | 1.00 | 0.19 |
| Treg\|Memory_B_cell | 1.00 | 1.00 | 1.00 | 1.00 | 0.00 | 1.00 | 1.00 | 1.00 | 1.00 | 1.00 | 1.00 | 1.00 | 0.00 | 1.00 | 1.00 | 1.00 | 1.00 | 1.00 | 0.00 | 0.00 |
| Treg\|NC-Mono | 1.00 | 1.00 | 1.00 | 1.00 | 1.00 | 1.00 | 1.00 | 1.00 | 1.00 | 1.00 | 1.00 | 1.00 | 1.00 | 0.19 | 1.00 | 1.00 | 1.00 | 1.00 | 0.00 | 1.00 |
| Treg\|NKT | 1.00 | 1.00 | 1.00 | 1.00 | 0.09 | 1.00 | 1.00 | 1.00 | 1.00 | 1.00 | 1.00 | 0.00 | 1.00 | 0.03 | 0.00 | 0.00 | 0.00 | 1.00 | 1.00 | 0.00 |
| Treg\|NaÃ¯ve_B_cell | 1.00 | 1.00 | 1.00 | 1.00 | 0.00 | 1.00 | 1.00 | 1.00 | 1.00 | 1.00 | 1.00 | 1.00 | 0.00 | 1.00 | 1.00 | 1.00 | 1.00 | 1.00 | 1.00 | 0.63 |
| Treg\|Plasma | 1.00 | 1.00 | 1.00 | 1.00 | 0.00 | 1.00 | 1.00 | 1.00 | 1.00 | 1.00 | 1.00 | 1.00 | 1.00 | 1.00 | 1.00 | 1.00 | 1.00 | 1.00 | 1.00 | 1.00 |
| Treg\|Treg | 1.00 | 1.00 | 1.00 | 1.00 | 1.00 | 1.00 | 1.00 | 1.00 | 1.00 | 1.00 | 1.00 | 0.00 | 1.00 | 1.00 | 1.00 | 1.00 | 1.00 | 1.00 | 1.00 | 0.19 |
| Treg\|γδ_T | 1.00 | 1.00 | 1.00 | 1.00 | 1.00 | 1.00 | 1.00 | 1.00 | 1.00 | 1.00 | 1.00 | 0.00 | 1.00 | 1.00 | 0.00 | 1.00 | 1.00 | 1.00 | 1.00 | 0.00 |
| γδ_T\|C-Mono1 | 1.00 | 0.00 | 0.00 | 1.00 | 0.00 | 0.00 | 1.00 | 1.00 | 0.00 | 0.00 | 1.00 | 0.00 | 1.00 | 0.37 | 1.00 | 1.00 | 1.00 | 1.00 | 0.00 | 0.94 |
| γδ_T\|C-Mono2 | 1.00 | 0.00 | 0.00 | 1.00 | 1.00 | 0.00 | 1.00 | 1.00 | 0.00 | 0.00 | 1.00 | 0.03 | 1.00 | 1.00 | 1.00 | 1.00 | 1.00 | 1.00 | 0.00 | 1.00 |
| γδ_T\|C-Mono3 | 1.00 | 0.00 | 0.00 | 1.00 | 1.00 | 0.00 | 1.00 | 1.00 | 0.00 | 0.00 | 1.00 | 0.00 | 1.00 | 0.81 | 1.00 | 1.00 | 1.00 | 1.00 | 0.00 | 1.00 |
| γδ_T\|CD4+_effector_T_cell | 1.00 | 1.00 | 1.00 | 1.00 | 1.00 | 0.00 | 1.00 | 0.25 | 0.91 | 1.00 | 1.00 | 0.00 | 1.00 | 1.00 | 1.00 | 1.00 | 1.00 | 1.00 | 1.00 | 0.00 |
| γδ_T\|CD4+_memory_T_cell | 1.00 | 1.00 | 1.00 | 1.00 | 1.00 | 0.00 | 1.00 | 0.00 | 1.00 | 1.00 | 1.00 | 0.00 | 1.00 | 1.00 | 1.00 | 1.00 | 1.00 | 1.00 | 1.00 | 0.29 |
| γδ_T\|CD4+_naÃ¯ve_T_cell | 1.00 | 1.00 | 1.00 | 1.00 | 1.00 | 0.00 | 1.00 | 0.06 | 1.00 | 0.72 | 1.00 | 0.00 | 1.00 | 1.00 | 1.00 | 1.00 | 1.00 | 1.00 | 1.00 | 1.00 |
| γδ_T\|CD56brightCD16-_NK | 1.00 | 0.00 | 0.87 | 1.00 | 0.00 | 0.00 | 1.00 | 0.00 | 0.88 | 1.00 | 1.00 | 0.00 | 1.00 | 0.00 | 0.00 | 0.00 | 0.00 | 1.00 | 1.00 | 0.00 |
| γδ_T\|CD56dimCD16+_NK | 1.00 | 1.00 | 1.00 | 1.00 | 0.00 | 0.00 | 1.00 | 0.28 | 0.81 | 1.00 | 0.00 | 0.00 | 1.00 | 0.00 | 0.00 | 0.00 | 1.00 | 1.00 | 1.00 | 0.00 |
| γδ_T\|CD8+_naÃ¯ve_T_cell | 1.00 | 1.00 | 1.00 | 1.00 | 0.00 | 0.00 | 1.00 | 0.00 | 0.34 | 1.00 | 1.00 | 0.00 | 1.00 | 0.91 | 0.00 | 1.00 | 1.00 | 1.00 | 1.00 | 0.00 |
| γδ_T\|GNLY+CD8+_cytotoxic_T_cell | 1.00 | 1.00 | 1.00 | 1.00 | 1.00 | 0.00 | 1.00 | 0.91 | 0.88 | 1.00 | 1.00 | 0.00 | 1.00 | 0.19 | 1.00 | 1.00 | 1.00 | 1.00 | 1.00 | 0.00 |
| γδ_T\|GZMK+CD8+_cytotoxic_T_cell | 0.00 | 1.00 | 1.00 | 1.00 | 1.00 | 0.00 | 1.00 | 0.00 | 0.88 | 1.00 | 1.00 | 0.00 | 1.00 | 1.00 | 1.00 | 1.00 | 1.00 | 1.00 | 1.00 | 0.00 |
| γδ_T\|MAI_T | 0.00 | 0.00 | 1.00 | 1.00 | 0.00 | 0.00 | 1.00 | 0.00 | 0.94 | 1.00 | 1.00 | 0.00 | 1.00 | 1.00 | 1.00 | 1.00 | 1.00 | 1.00 | 1.00 | 0.15 |
| γδ_T\|Memory_B_cell | 1.00 | 1.00 | 1.00 | 1.00 | 0.00 | 0.00 | 1.00 | 0.03 | 0.91 | 1.00 | 1.00 | 0.94 | 0.00 | 1.00 | 1.00 | 1.00 | 1.00 | 1.00 | 0.00 | 0.00 |
| γδ_T\|NC-Mono | 1.00 | 0.00 | 0.75 | 1.00 | 1.00 | 0.00 | 1.00 | 1.00 | 0.00 | 0.41 | 1.00 | 0.03 | 1.00 | 0.00 | 1.00 | 1.00 | 1.00 | 1.00 | 0.00 | 0.97 |
| γδ_T\|NKT | 1.00 | 1.00 | 1.00 | 1.00 | 0.00 | 0.00 | 1.00 | 0.00 | 0.18 | 1.00 | 0.00 | 0.00 | 1.00 | 0.00 | 0.00 | 0.00 | 0.00 | 1.00 | 1.00 | 0.00 |
| γδ_T\|NaÃ¯ve_B_cell | 1.00 | 1.00 | 1.00 | 1.00 | 0.00 | 0.00 | 1.00 | 1.00 | 1.00 | 0.22 | 1.00 | 1.00 | 0.00 | 1.00 | 1.00 | 1.00 | 1.00 | 1.00 | 1.00 | 0.38 |
| γδ_T\|Plasma | 1.00 | 1.00 | 1.00 | 1.00 | 0.00 | 0.94 | 1.00 | 0.00 | 0.00 | 0.94 | 1.00 | 1.00 | 1.00 | 1.00 | 1.00 | 1.00 | 1.00 | 1.00 | 1.00 | 1.00 |
| γδ_T\|Treg | 1.00 | 1.00 | 1.00 | 1.00 | 1.00 | 0.00 | 1.00 | 0.00 | 0.97 | 1.00 | 1.00 | 0.00 | 1.00 | 1.00 | 1.00 | 1.00 | 1.00 | 1.00 | 1.00 | 0.00 |
| γδ_T\|γδ_T | 0.00 | 1.00 | 1.00 | 1.00 | 1.00 | 0.00 | 1.00 | 0.00 | 0.85 | 1.00 | 1.00 | 0.00 | 1.00 | 1.00 | 0.00 | 1.00 | 1.00 | 1.00 | 1.00 | 0.00 |
